# Whole Genome Sequence Analysis of *Brucella abortus* Isolates from Various Regions of South Africa

**DOI:** 10.3390/microorganisms9030570

**Published:** 2021-03-11

**Authors:** Maphuti Betty Ledwaba, Barbara Akorfa Glover, Itumeleng Matle, Giuseppe Profiti, Pier Luigi Martelli, Rita Casadio, Katiuscia Zilli, Anna Janowicz, Francesca Marotta, Giuliano Garofolo, Henriette van Heerden

**Affiliations:** 1 Department of Veterinary Tropical Diseases, Faculty of Veterinary Science, University of Pretoria, Onderstepoort, Pretoria 0110, South Africa; realrhema@gmail.com; 2 Bacteriology Division, ARC-Onderstepoort Veterinary Research, Onderstepoort, Pretoria 0110, South Africa; MatleI@arc.agric.za; 3 Bologna Biocomputing Group, University of Bologna, I-40126 Bologna, Italy; giuseppe.profiti2@unibo.it (G.P.); gigi@biocomp.unibo.it (P.L.M.); rita.casadio@unibo.it (R.C.); 4 National and OIE Reference Laboratory for Brucellosis, Experimental Zooprophylactic Institute of Abruzzo and Molise Giuseppe Caporale, 64100 Teramo, Italy; k.zilli@izs.it (K.Z.); a.janowicz@izs.it (A.J.); f.marotta@izs.it (F.M.); g.garofolo@izs.it (G.G.)

**Keywords:** bovine brucellosis, *Brucella abortus*, whole genome sequence, single nucleotide polymorphisms, comparative analysis

## Abstract

The availability of whole genome sequences in public databases permits genome-wide comparative studies of various bacterial species. Whole genome sequence-single nucleotide polymorphisms (WGS-SNP) analysis has been used in recent studies and allows the discrimination of various *Brucella* species and strains. In the present study, 13 *Brucella* spp. strains from cattle of various locations in provinces of South Africa were typed and discriminated. WGS-SNP analysis indicated a maximum pairwise distance ranging from 4 to 77 single nucleotide polymorphisms (SNPs) between the South African *Brucella abortus* virulent field strains. Moreover, it was shown that the South African *B. abortus* strains grouped closely to *B. abortus* strains from Mozambique and Zimbabwe, as well as other Eurasian countries, such as Portugal and India. WGS-SNP analysis of South African *B. abortus* strains demonstrated that the same genotype circulated in one farm (Farm 1), whereas another farm (Farm 2) in the same province had two different genotypes. This indicated that brucellosis in South Africa spreads within the herd on some farms, whereas the introduction of infected animals is the mode of transmission on other farms. Three *B. abortus* vaccine S19 strains isolated from tissue and aborted material were identical, even though they originated from different herds and regions of South Africa. This might be due to the incorrect vaccination of animals older than the recommended age of 4–8 months or might be a problem associated with vaccine production.

## 1. Introduction

The genus *Brucella* is a highly monomorphic genus comprised of gram-negative pathogenic species [1] affecting a wide range of hosts, including humans. Insufficient progress has been achieved in the identification of species within this genus, since their homogeneity has compromised discrimination, especially when using molecular typing tests. The introduction of newly described atypical *Brucella* species extended the level of diversity within the genus [2,3,4,5,6]. For instance, *Brucella* spp. isolated from amphibians are reported to be motile, despite the fact that the genus historically consists of non-motile species [6,7,8]. Species from this genus cause varying disease spectra with the collective name brucellosis. Although the infection has been eradicated in most developed and industrialized countries, it is still widely endemic elsewhere and contributes to massive economic losses, as well as veterinary and public health distress in many countries, especially those located in Africa, Central Asia, and the Middle East and some countries of the European Mediterranean area [9,10]. *Brucella abortus*, which is the causal agent of bovine brucellosis, affects the *Bovidae* family and its natural reservoir hosts include cattle (*Bos taurus*), African buffalo (*Syncerus caffer*), water buffalo (*Bubalus bubalis*), elk (*Cervus canadensis*), and American bison (*Bison bison*) [11]. Regardless of the host preference within the genus, it is evident that species can also affect hosts other than their preferred ones, especially if there is a close interaction between animals [12,13,14]. Therefore, it is crucial to accurately understand the inter-species relationships of the genus *Brucella*, in order to comprehend the complete epidemiology of the disease [15]. Even though there is no assay that can solely diagnose brucellosis accurately, except for the gold standard (culturing), a number of molecular assays that allow the differentiation of *Brucella* spp. and their biovars, as well as tracing back the source of the outbreak, have been reported [16,17,18,19,20,21].

Brucellosis is a notifiable disease in South Africa (SA) and is controlled according to the Animal Disease Act 35 of 1984. Bovine brucellosis is controlled based on the bovine brucellosis scheme (R.2483 of 9 Dec 1988) established under Section 10 of Act 35. The scheme involves the vaccination of heifer aged between 4 and 8 months with a 5 × 10^10^ organisms of *Brucella abortus* S19 vaccine and serological testing of high-risk herds that are suspected of or have been confirmed as being infected. The government continuously encourages farmers to participate in the scheme; however, it is voluntary. Moreover, the scheme is under review and several amendments, which include the compulsory participation of all animal owners and livestock, reporting of any abortion cases within the herd, etc., have been suggested [22]. This document also emphasizes that “to date, there are limited recent empirical data or published reports on the existence or prevalence of brucellosis in the livestock industry in South Africa”. Furthermore, it indicates that farmers buying replacement stock may be at risk of introducing infected animals to their herd since most farmers are not complying with the bovine brucellosis regulations. Ninety percent (90%) of bovine brucellosis infections in South Africa are reported [23] to be caused by *B. abortus* bv. 1, while 10% are due to *B. abortus* bv. 2. In addition, the 2015–2016 Gauteng Province Veterinary Services Annual Report [24] showed a sero-prevalence of 1.27% (30/2359) in the province when testing bovine brucellosis with the rose Bengal test (RBT) and complement fixation test (CFT). Therefore, initial steps that may help in the eradication of brucellosis include the identification of strains occurring in the country, host species, and sources of infection. Moreover, it will be essential to determine whether the same strains are circulating within the source of infection regions to avoid misinterpretation of the situation.

Whole genome sequence-single nucleotide polymorphisms (WGS-SNP) analysis was used in the present study, based on previous reports demonstrating its efficacy in the discrimination of various conserved *Brucella* strains and biovars [25,26,27]. In addition, a single nucleotide polymorphism (SNP) microarray was previously used to establish the evolutionary lineage of *Brucella* spp. by distinguishing the major clades of *B. abortus, B. melitensis,* and *B. suis* and assigning the strains to their designated lineages [28]. The analysis of raw data generated from sequencing with different platforms has improved due to the available bioinformatics tools, which have helped in the achievement of novel discoveries and clarification of most biological processes, leading to accurate results [29,30]. Variant calling tools used during the WGS-SNP analysis must include parameters that will allow for errors encountered during the data preparation, which include amplification biases and machine and software errors encountered during sequencing and sequence mapping/alignment [31], but the choice of the variant tool essentially depends on the study aims and objectives. Moreover, the reference genome chosen for use in WGS-SNP analysis should be considered as it can crucially influence the output number of SNPs, which in turn will affect the accuracy of the overall phylogenetic relationships [32]. Several studies have reported the use of SNP analysis in successfully discriminating between *B. melitensis* strains, as well as determining their geographic and global distribution [26,33,34]. The above authors [33] also reported that the WGS-SNP-based analysis used in their study to determine the phylogenetic relatedness of *B. melitensis* has a better resolution in grouping the strains into their specific genotypes based on their geographic distribution. Therefore, the current study also used WGS-SNP analysis to determine the relationship and genetic variation of *B. abortus* strains that exist within a single herd (farm) and between various herds in SA. Strains from two farms (Farm 1 and Farm 2) and some of those submitted to the Agricultural Research Council-Onderstepoort Veterinary Research (ARC-OVR) for routine screening were used and compared with global sequences available online. Strains from ARC-OVR are grouped under the name “other” in the generated trees since they are isolated from samples submitted at the national reference laboratory from different regions throughout SA.

## 2. Materials and Methods

### 2.1. Samples and Sample Area

Milk samples were collected opportunistically by animal health technicians during routine brucellosis eradication visits at the identified farms. Farm 1 and Farm 2 were considered brucellosis-infected herds because they had a history of brucellosis following repeated sampling by Gauteng Department of Agriculture and Rural Development (GDARD) animal health technicians and screening with serum agglutination test (SAT), RBT, and CFT at ARC-OVR. Complete random sampling of animals aged 18 months and above was applied to avoid false positive reactions that might result from vaccination since heifers at the farms are vaccinated at 4–8 months of age with the *B. abortus* S19 vaccine. Some of the samples were collected from previously identified (C-branded) seropositive animals, which are those that tested positive for RBT and CFT during a previous state veterinary surveillance and control program and were to be culled. Approximately 20 mL of milk was collected from each teat in a sterile tube and transported on ice to the Faculty of Veterinary Sciences, Department of Veterinary Tropical Diseases’ bacteriology laboratory for culturing. *Brucella* cultures from Farms 1 and 2 were submitted to ARC-OVR for biotyping [35] using standard microbiology procedures [36]. More strains were isolated and biotyped at ARC-OVR from tissue samples submitted for routine screening from different provinces and regions of South Africa. Briefly, isolates were biotyped based on their colony morphology; agglutination on anti-*Brucella* mono-specific sera *abortus* and *melitensis*; reaction to oxidase, urease, and catalase tests; production of hydrogen sulphide; growth in the absence of carbon dioxide; growth in the presence of basic fuchsin and thionin dyes; lysis by different phages (Tbilisi; Weybridge; Izatnagar strain 1; Berkeley strain 2); and inhibition by erythritol (1000 µg) and antibiotics (Streptomycin 10 µg, Penicillin G 10 units, and Rifampicin 30 µg) (35,36). A *Brucella abortus* str. 544 culture was used as a control in all the phenotypic tests.

### 2.2. Bacterial Strains, DNA Extraction, Quantification, and Molecular Identification

Milk samples collected from infected bovine herds in different provinces of South Africa were cultured with modified-Agrifood Research and Technology Center of Aragon (M-CITA) [35] for bacterial isolation. DNA was extracted with the High Pure PCR Template Preparation Kit (Roche Products Pty (Ltd.), Midrand, South Africa) from positive culture plates according to the manufacturer’s instructions. It was then quantified with multi-volume plate nanodrop analysed with the Take3 Session (BioTek Instruments, Thermo Fisher Scientific, Centurion, South Africa). A representative that consisted of 13 *B. abortus* strains was randomly selected and sequenced (Table 1 and Table 2). From the total strains isolated and selected for sequencing, three strains were isolated from samples collected at Farm 1 and two from Farm 2, while the others were isolated from samples submitted to ARC-OVR for routine screening from different provinces and regions of SA (Table 2). Moreover, they are dairy farms in Gauteng province, identified as brucellosis-infected farms by the animal health technician and state veterinarian working in the area. The farms have no record of trade between each other. All isolates used in the study were differentiated with species-specific abortus-melitensis-ovis-suis (AMOS) [16] and Bruce-ladder [18] multiplex polymerse chain reaction (PCR) assays, following the protocol described elsewhere. SA-UK6 was isolated in a later stage in the study and identified as *Brucella* spp. using the *Brucella* genus-specific 16 S–23 S rRNA interspace (ITS) PCR [37]. It was included in the sequencing list after the biotyping results at ARC-OVR indicated that it was *B. abortus* S19.

### 2.3. Whole-Genome Sequencing

Isolates were sequenced with the Illumina NextSeq 500 platform with 150 bp paired-end chemistry. The genome libraries were prepared with the Nextera XT Library Prep kit (Separations, Randburg, South Africa), according to the manufacturer’s recommendations. Isolates were sequenced with Illumina’s recommended dual index barcoding protocol, with the aim of obtaining a read coverage ranging from 18 to 356-fold, with an average of 155-fold. Reads with a quality score of <20 and a length shorter than 70 bp, as well as a Phred mean quality of less than 24, were trimmed at the 5′-end and 3′-end and poor quality reads were then discarded.

### 2.4. WGS-SNP Data Analysis

The sequences were assigned taxonomic identities using Kraken [38], which is a sequence classifier that queries the database of *k*-mers for exact-matches. Sequences were deposited in GenBank and assigned accession numbers, as shown in Table 1. In silico genotyper (ISG) version 0.16.10-3 [39] was used to identify the SNPs among the genomes. The ISG is an open source tool that identifies and annotates variants from nucleotide sequences. The current pipeline uses BWA-MEM (version 0.712-r1039) [40] for genome alignment and GATK version 3.9 [41] to call the SNPs. For comparative WGS-SNP, we retrieved 175 currently available *B. abortus* genomes from GenBank [42] and selected *B. melitensis* 16M (Accession numbers: AE008917; AE008918) as the outgroup strain. Moreover, *B. abortus* strain 2308 (Accession numbers: NC_007618; NC_007624) was used as the reference for the SNPs calling alignment. For the SNPs calling process, default parameters were used to filter and remove duplicates, with the minimum quality set at Phred 30. From the generated output, clean unique variants were used to further analyze the sequences. Default parameters of the PHYLOViZ software version 2.0 [43] were used to generate maximum spanning trees (MST) with the SNP data of the South African strains, in order to determine the geographic distribution of the strains and possible connections between the localities. Furthermore, a phylogenetic tree was constructed with maximum parsimony in Phylogenetic Analysis Using Parsimony (PAUP) Software version 4.0b [44], using 600 bootstrap replicates to assess the branch support. Since the tree was bigger and unclear to read, smaller sections were snipped from the tree for clear visibility.

## 3. Results and Discussion

**Figure 1:**
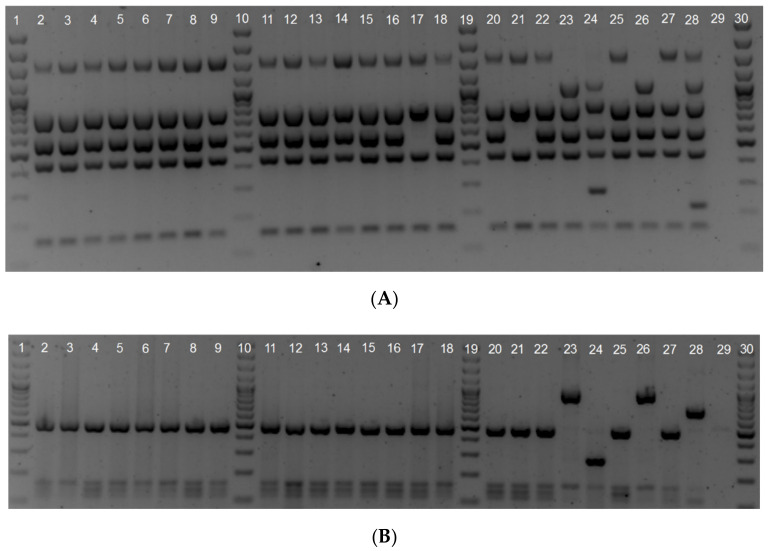
Gel images of Bruce-ladder (**A**) and AMOS (**B**) multiplex PCR assays. Descriptive information is given in Table S1.

The isolates were speciated and characterized with a Bruce-ladder [18] (Figure 1A) and AMOS [16] (Figure 1B) multiplex assay alongside thirteen others that were not sequenced but tested with molecular assays. All of the isolates were biotyped at the ARC-OVR, with most identified as *B. abortus* bv. 1, two as *B. abortus* bv.2, and two as *B*. *abortus* S19, as shown on Table 2 and previously in Ledwaba et al. [35].

The *Brucella* strains sequenced in the present study were all classified as *B. abortus* (Table 2) by the Kraken taxonomic classifier. The tool only indicated that the sequences were *B. abortus* since its sensitivity is up to the genus level; thus, it cannot differentiate between the biovars and it cannot distinguish the vaccine strains from the virulent ones. Nonetheless, Wood and Salzberg [38] indicated that although Kraken is only genus-specific, it is quicker than most classifiers and its accuracy, which is capacitated by the growing number of sequences, is comparable to that obtained with BLAST.

A comparative analysis of the thirteen South African and 175 *B. abortus* genome assemblies retrieved from the National Centre for Biotechnology Information (NCBI) resulted in a total of 22,307 variants, of which 17,379 are clean unique variants (Table S2), which were used to generate the maximum spanning trees (MST). To determine the possible connection between the South African farms, the MST tree was generated with clean unique variants from only SA strains.

**Figure 2:**
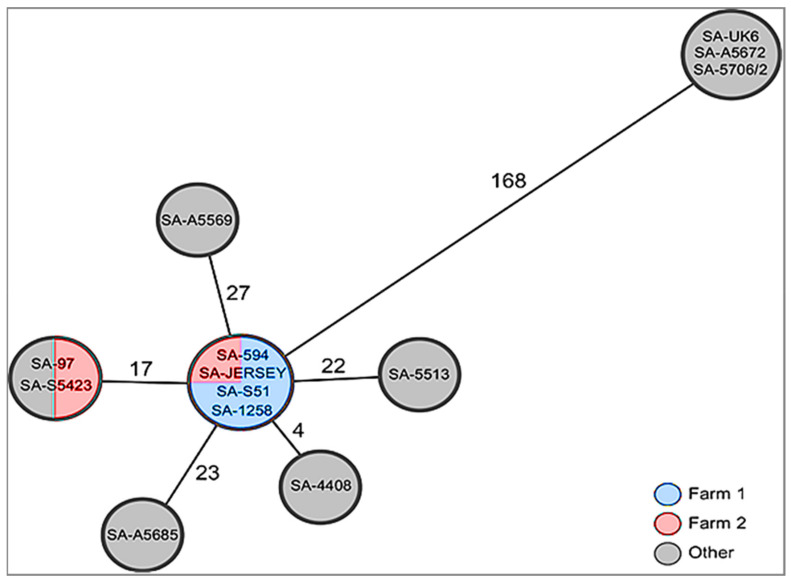
Minimum spanning tree of 13 South Africa (SA) strains depicting the connection between Farm 1 (shown with a blue background), Farm 2 (shown with a pink background), and isolates from the Agricultural Research Council-Onderstepoort Veterinary Research (ARC-OVR) (shown with a gray background). It also shows significant variation (168 single nucleotide polymorphisms (SNPs)) between the field and vaccine strains.

Figure 2 depicts a maximum pairwise distance ranging from 4 to 27 SNPs between all the South African virulent field strains from Farm 1, Farm 2, and ARC-OVR. Several genotypes were observed between the South African field strains (Figure 2). However, the three *B. abortus* S19 vaccine strains isolated from tissue and aborted material showed that they are identical but different to the virulent strains, with a significant variation of 168 SNPs (Figure 2).

**Figure 3:**
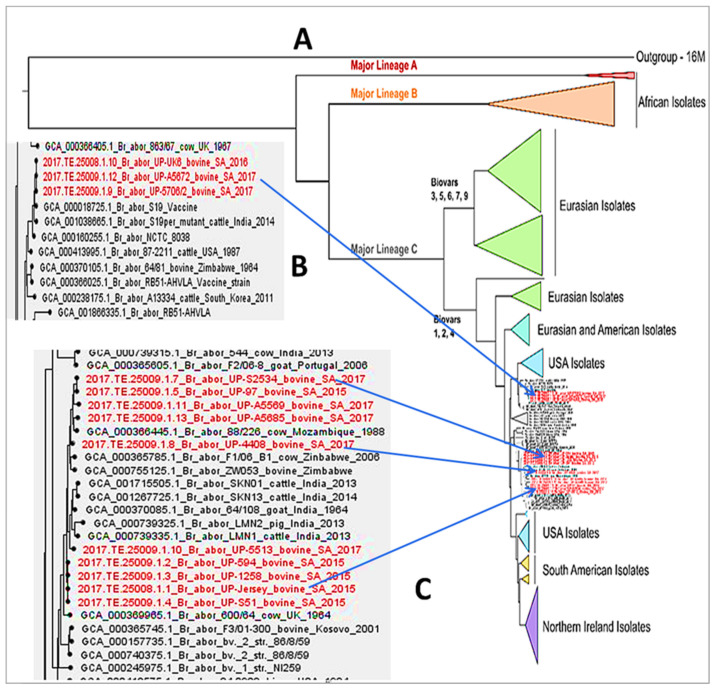
(**A**) A phylogenetic tree based on WGS-SNP depicting the relationship between 13 South African strains and 175 *Brucella abortus* genomes available online. The tree was generated with single nucleotide polymorphism (SNP) analysis using *B. abortus* bv. 1 strain 2308 as a reference and *Brucella melitensis* bv. 1 strain 16M as an outgroup. The labels in red on the tree (**A**) represent the South African strains. (**B**) A section cut from the original tree indicating the South African *B. abortus* S19 and its closely related strains. (**C**) A section also cut from the original tree illustrating South African *B. abortus* virulent strains and their closely related ones. NB: Strain S5423 (**C**) was incorrectly labelled as S2534.

The minimum spanning and phylogenetic trees (Figure 2 and Figure 3) generated with the WGS-SNP data respectively showed that all *B. abortus* bv. 1 strains from Farm 1 clustered together with one of the strains from Farm 2, whereas the SA-97 strain from Farm 2 clustered with the SA-5423 strain, although in one main group with the others (Figure 2 and Figure 3C). This indicates that there was one clonal genotype circulating within Farm 1, while multiple genotypes were found within Farm 2. These farms are approximately 80 km from each other, with Farm 1 located in Springs and Farm 2 located in Bronkhorstspruit in Gauteng province.

Moreover, previous farm records showed that both farms were disease-free in the past years; thus, the isolated strains might have been introduced on the farms through the replacement stock brought in without knowledge of their brucellosis status. The presence of the same strain on both farms indicates that they may have introduced cattle from the same infected source. Additionally, the clustering of SA-97 from Farm 2 with SA-5423, which is one of the ARC-OVR strains, points out that this strain might be occurring and circulating on various farms around South Africa, since the institution (ARC-OVR) is a national reference laboratory and thus receives samples from all provinces.

Moreover, South African sequences did not cluster within the major lineages A and B, despite the fact that most African isolates cluster to these lineages. However, they clustered with a few strains from African countries (Mozambique and Zimbabwe), as well as many strains from Eurasia (including Portugal and India). This was also previously shown in several molecular analysis studies that provided insight into the heterogeneity of *B. abortus* strains from Africa, Europe, Maghreb, and Sub-Saharan countries [45,46,47]. Additionally, it suggests that *B. abortus* might have spread into/out of South Africa as a result of socio-economic, migration, or colonization links between the countries in the past. Previous studies have also demonstrated that *B. abortus* isolates from sub-Saharan countries and those from Europe cluster together, even though heterogeneity within the clusters exists [48,49]. This was also shown in a study by Khames et al. [46], who indicated that Algerian *B. abortus* isolates used in their study clustered with those from Morocco, Zimbabwe, France, Germany, Portugal, and Italy.

*Brucella abortus* S19 vaccine strains isolated in this study grouped with *B. abortus* S19 per_mutant from India, as well as the *B. abortus* S19 reference strain (Figure 3B). Moreover, Figure 2 and Figure 3B clearly show that the three SA *B. abortus* S19 strains are identical. This was unanticipated since they were isolated from tissue and aborted material in different provinces of South Africa (Gauteng and Western Cape provinces). This suggests that the vaccine used for immunization might be from the same batch, which had production problems, or that the cows were vaccinated, with a full dose given to heifers of 4–8 months while pregnant or at a later stage than recommended by the manufacturer. Moreover, it has been shown that 70% of the cattle vaccinated with the *B. abortus* S19 live vaccine get protection against the virulent wild-type strains; however, the vaccine can also induce abortions if full doses are given to pregnant animals [50,51]. The isolation of clonal vaccine strains from livestock was also reported in a previous study in which two *B. melitensis* Rev. 1 isolates from sheep and two *B. abortus* RB51 isolates from bovine shared the same genotype and clustered with other available vaccine sequences of their type [52]. Previous studies [53,54] indicated that the vaccination of pregnant cows with a dose of 5 × 10^10^ of the attenuated *B. abortus* S19 vaccine has induced abortions in a significant number of vaccinated cows. Animals, mostly those vaccinated as adults, show persistent serological reactions against the antigenic O-chain of the lipopolysaccharide of the smooth *Brucella* strains [55]. In addition, a recent study showed that pregnant heifers vaccinated with a high dose of the *B. abortus* S19 vaccine displayed an increased body temperature within the first two days post vaccination [56]. Nevertheless, it was previously demonstrated that [57] the subcutaneous or conjunctival vaccination of animals with a reduced dose ranging from 3 ×10^8^ to 5 × 10^9^ can decrease the serological antibody response. In another study of Poester et al. [58], the low dose of 3 × 10^9^ was used in adult animals and resulted in a no antibody response from all of the animals when tested with RBT nine months after vaccination.

WGS-SNP analysis has been previously used to discriminate between various homologous *Brucella* strains [24,25]. It can be effective in the discrimination and comparative analysis of closely related *Brucella* spp., provided there is enough data available for comparison [25,59]. SNP calling requires well-processed data and a robust calling tool that is user-friendly and easier to apply. Even though there are far fewer genomes available for SNP-based comparison studies than multi-locus variable numbers of tandem repeat multi locus variable number of tandem repeat anlysis (MLVA) allele profiles, WGS-SNP analysis can still provide a better resolution since polymorphism can be established from either the coding or non-coding regions [34]. The phylogenetic analysis of bacterial strains provides information that could help in the development of rapid diagnostic assays for epidemiology, as well as understanding the structure, genetic diversity, and evolutionary history of the strains in question [60]. Currently, MLVA and multilocus sequence typing (MLST) are used in the typing of *Brucella* species [19,46,61], and the availability of WGS in public repositories makes it possible to conduct genome-wide comparative studies of various bacterial species [34,62]. However, it is still complicated to perform phylogeographic categorization because of the speculative information about the pathogen’s origin and the possible migration route [34,62]. Zaki and co-workers [63] reported that WGS-SNP-based analysis has proven to be a rapid tool for epidemiological studies and tracing outbreaks since it efficiently discriminated between 33 *Brucella* isolates by determining their native geographical origin based on spatial clustering. The reliability and reproducibility of SNP analysis were further shown in a study by Janowicz et al. (2018) [32], who indicated that it performed better than MLVA-16 and core genome multilocus sequence typing (cgMLST), even though there was little variation.

In the present study, the SNP analysis showed that it is a powerful tool to use in differentiating homogenous species. WGS-SNP analysis depicted that there is one clonal genotype circulating within Farm 1, but varying genotypes are present on Farm 2. The occurrence of one genotype on both farms indicates that both farms may have unknowingly introduced the infection from the same source, where they might have bought replacement stock. Comparatively, the presence of multiple genotypes on Farm 2 indicates that there may have been multiple introductions of the replacement stock from different herds. The unmonitored movement of animals from one herd to another poses a serious problem of disease transmission in most countries. In addition, WGS-SNP comparative analysis was able to discriminate the *B. abortus* S19 strains from the virulent strains, as well as illustrating that the vaccine strains are identical, despite their varying origin. Farmer’s days and workshops to discuss the effects of the incorrect use of vaccines and the unapproved movement of animals from one herd to another are necessary measures that can supplement the available precautions and the brucellosis control and eradication programs, especially with the emerging small-scale farmers.

## Figures and Tables

**Table 1:** A list indicating the old sample names, new assigned sample names, and accession numbers of South African *Brucella abortus* strains characterized in this study.

Current Sample Name ^a^	Old Sample Name ^b^	Accession Numbers
*B. abor*_UP-Jersey	SA-JERSEY	SAMN15685093
*B. abor*_UP-594	SA-594	SAMN15685094
*B. abor*_UP-1258	SA-1258	SAMN15685095
*B. abor*_UP-S51	SA-S51	SAMN15685096
*B. abor*_UP-97	SA-97	SAMN15685097
*B. abor*_UP-4408	SA-4408	SAMN15685098
*B. abor*_UP-5423	SA-S5423	SAMN15685099
*B. abor*_UP-5706/2	SA-5706/2	SAMN15685100
*B. abor*_UP-5513	SA-5513	SAMN15685101
*B. abor*_UP-A5569	SA-A5569	SAMN15685102
*B. abor*_UP-A5672	SA-A5672	SAMN15685103
*B. abor*_UP-A5685	SA-A5685	SAMN15685104
*B. abor*_UP-UK6	SA-UK6	SAMN15685105

^a^ The old sample names used in Figure 1. ^b^ The new assigned names used in Figure 2.

**Table 2:** *Brucella* isolates sequenced and their identity, as determined by phenotyping identification and PCR assays (AMOS and Bruce-ladder) taxonomically classified with Kraken.

Sample Name	Sample ID	Host	Sample Source	Country of Origin	Sample ID (Assay Used)	Kraken ID
SA-JERSEY	2017.TE.25009.1.1	cattle	Milk	SA (F1 ^a^)	*B. abortus*: AMOS, Bruceladder; *B. abortus* bv. 1: biotyping	*B. abortus*
SA-594	2017.TE.25009.1.2	cattle	Milk	SA (F1 ^a^)	*B. abortus*: AMOS, Bruceladder; *B. abortus* bv. 1: biotyping	*B. abortus*
SA-1258	2017.TE.25009.1.3	cattle	Milk	SA (F1 ^a^)	*B. abortus*: AMOS, Bruceladder; *B. abortus* bv. 1: biotyping	*B. abortus*
SA-S51	2017.TE.25009.1.4	cattle	Milk	SA (F2 ^b^)	*B. abortus*: AMOS, Bruceladder; *B. abortus* bv. 1: biotyping	*B. abortus*
SA-97	2017.TE.25009.1.5	cattle	Milk	SA (F2 ^b^)	*B. abortus*: AMOS, Bruceladder; *B. abortus* bv. 1: biotyping	*B. abortus*
SA-4408	2017.TE.25009.1.7	cattle	Tissue	SA (ARC-OVR)	*B. abortus*: AMOS, Bruceladder; *B. abortus* bv. 1: biotyping	*B. abortus*
SA- 5423 ^c^	2017.TE.25009.1.8	cattle	Tissue	SA (ARC-OVR)	*B. abortus*: AMOS, Bruceladder; *B. abortus* bv. 1: biotyping	*B. abortus*
SA-5706/2	2017.TE.25009.1.9	cattle	Tissue	SA (ARC-OVR)	*B. abortus* S19 (AMOS; Bruceladder, biotyping)	*B. abortus*
SA-5513	2017.TE.25009.1.10	cattle	Abomasal fluid	SA (ARC-OVR)	*B. abortus*: AMOS, Bruceladder; *B. abortus* bv. 2: biotyping	*B. abortus*
SA-A5569	2017.TE.25009.1.11	cattle	Tissue	SA (ARC-OVR)	*B. abortus*: AMOS, Bruceladder; *B. abortus* bv. 2: biotyping	*B. abortus*
SA-A5672 ^d^	2017.TE.25009.1.12	cattle	Tissue	SA (ARC-OVR)	*B. abortus* S19 (AMOS; Bruceladder, biotyping)	*B. abortus*
SA-A5685	2017.TE.25009.1.13	cattle	Tissue	SA (ARC-OVR)	*B. abortus*: AMOS, Bruceladder; *B. abortus* bv. 1: biotyping	*B. abortus*
SA-UK6 ^c^^,f^	2017.TE.25008.1.10	cattle	Aborted fetus	SA (ARC-OVR)	*Brucella* spp. (ITS) ^e^;	*B. abortus*
					*B. abortus* S19 (biotyping)	

^a^ F1 = Farm 1 in Gauteng province; ^b^ F2 = Farm 2 in Gauteng province; ^c^ submitted from Western Cape province; ^d^ submitted from Free State province; ^e^ 16–23 S ribosomal DNA interspacer PCR. ^f^ Molecularly identified (ITS PCR) and biotyped at a later stage in the study.

## Data Availability

All relevant data are within the paper and its supplementary information files.

## Supplementary Materials

Available online at https://www.mdpi.com/2076-2607/9/3/570/s1: Table S1: Sample order used in Bruce-ladder (A) and AMOS (B) multiplex PCR assays and the descriptive information of the gel images, Table S2: Clean unique variants of the South African strains (refer to Table 2 for the sample names (in column 1) and sample ID (in column 2)).
