# Supplementary material for: Whole Genome Sequence Analysis of Brucella abortus Isolates from Various Regions of South Africa
Source: Microorganisms. 2021 Mar 11;9(3):570. doi: 10.3390/microorganisms9030570 (PMC7998772; doi:10.3390/microorganisms9030570)
Supplement: Supplementary file 1 [file microorganisms-09-00570-s001.pdf]

Table S1: Samples order used in Bruce-ladder **(A)** and AMOS **(B)** multiplex PCR assays and the descriptive information of the gel images.

LANES	SAMPLES	SPECIES	BRUCE-LADDER[a]	AMOS[a]
**1**	**100 bp+ DNA marker:** 3000; 2000; 1500; 1000; 900; 800; 700; 600; 500; 400; 300; 200; 100			
**2**	SA-JERSEY	*Brucella abortus*	1682, 794, 587, 450 & 152	498
**3**	SA-594	*Brucella abortus*	1682, 794, 587, 450 & 152	498
**4**	SA- 1258	*Brucella abortus*	1682, 794, 587, 450 & 152	498
**5**	SA-97	*Brucella abortus*	1682, 794, 587, 450 & 152	498
**6**	SA-S51	*Brucella abortus*	1682, 794, 587, 450 & 152	498
**7**	SA-5423[#]	*Brucella abortus*	1682, 794, 587, 450 & 152	498
**8**	SA-BA34	*Brucella abortus*	1682, 794, 587, 450 & 152	498
**9**	SA-BA41	*Brucella abortus*	1682, 794, 587, 450 & 152	498
**10**	**100 bp+ DNA marker**			
**11**	SA-4408	*Brucella abortus*	1682, 794, 587, 450 & 152	498
**12**	SA-5423/1	*Brucella abortus*	1682, 794, 587, 450 & 152	498
**13**	SA-5423/2	*Brucella abortus*	1682, 794, 587, 450 & 152	498
**14**	SA-5423/3	*Brucella abortus*	1682, 794, 587, 450 & 152	498
**15**	SA-5513	*Brucella abortus*	1682, 794, 587, 450 & 152	498
**16**	SA-5569	*Brucella abortus*	1682, 794, 587, 450 & 152	498
**17**	SA-5672	*Brucella abortus* S19	1682, 794, 450 & 152	498
**18**	SA-5685	*Brucella abortus*	1682, 794, 587, 450 & 152	498
**19**	**100 bp+ DNA marker**			
**20**	SA-5706/1	*Brucella abortus*	1682, 794, 587, 450 & 152	498
**21**	SA-5706/2	*Brucella abortus* S19	1682, 794, 450 & 152	498
**22**	SA-5706/3	*Brucella abortus*	1682, 794, 587, 450 & 152	498
**23**	SA-RC48[b]	*Brucella ovis*	1071, 794, 587, 450 & 152	976
**24**	ZW011 [b]	*Brucella suis*	1071, 794, 587, 450, 272 & 152	285
**25**	86/6/59 [b]	*Brucella abortus*	1682, 794, 587, 450 & 152	498
**26**	63/290 [b]	*Brucella ovis*		976
**27**	544 [b]	*Brucella abortus*	1682, 794, 587, 450 & 152	498
**28**	Rev1 [b]	*Brucella melitensis* Rev1	1682, 1071, 794, 587, 450, 218 & 152	731
**29**	NEGATIVE	Control	N/A	N/A
**30**	**100 bp+ DNA marker**			

[a]Assay fragment sizes in bp; [b]Positive controls; # mistakenly listed as SA-2534 instead of SA-5423

Table S2: Clean unique variants of the South African strains

>2017.TE.25009.1.5

CACGCCCCCCTCCCGGCCAGCCCGCCCCCCGCCCCAGCGTCGTACCCGGCGTCGCGACTCCCCGCCGTCGAGAGGCCTTTGCCGGGGCCCCGGCTAGCC
CGGGGCGAGCGGACCGCCGCGCGCGGGGGGTGTCACCCGGGGCACACCGCGCCAGCCTCCCGGGCATGCCACGCCGGGTTCCGCCCCCGCTGCTCCAA
TTTGGGGGGCCCCTAAGATCCCGTTCCCCAGGCCAGCCGCGCCCGCGGGCCAACATTGCCACACCGTGTCGCCCCCCCGCCTGCCGCACTGACCGTCTTG
CGGGCCCTGCTCCCTGGCCCGAGCACACCTCCGGGGACCCCCCTCGCTCGGCCGGGGGAGCCTTAAGCGCGGCACCGAGTAGGGGGTCGCGTGTTGCCG
CCGCGCTGACGCCCCGGCTGGGACCCCTTTCAGGTCGAGACGCCGGGGTGGCCATGCGGGTCCAGTCGCACGATGGCGCGGCGATGTGGCGCCGACTGC
CCCGCCTGTCTTCTCCGTGCCAGCCCCTCGCCACCATGCCGGTCCGGCCCGCACGGGGGTAGGAGGTGCTGCCGCGGCGGGAGGCGTAGGACGCCATAT
CCGAACCGGCGGGTCCCCCCGGCCACCGAGTCGACAGGACCATAGGCACCGCGTCCGGGCGGGCGCCCTTAACGCCGCCCCTTACTGGAGAAGCAGAG
CTCTGCCGAGGCACCCTGCCTAGCTGGCTGCGTCCCGCACCCCGCGATCCCCCTGGTCCACTTACCCAGCGGGATGCGGGGATGTTCGCAGCTATCACAC
GGTCGTGGCCTGCCTCCCTGGGCCGGCGAGAGCACCGCCACCTTCGTGTTCCTTGCCGCCGGGCGACGTGTCCGGGAAAGGCTTCGCCGCGCGCCCGCGC
GGAGCGCCGGGGAGTCCCGTGAGCGGCCGCAGGAACGTCACGGACTCCGGCTTGGTGCGGTCCCCCTCAGTGGTGCCCGACCAGAGACCGCGGAAAGA
CTCCAGGAAACGCCTCGGGGGGAAAGCAGGGCCGCGCACTTCCGCTCCACGCTAGCTGGGGGGACCACGACGGCAATACTGAACACCTGGCTCTTTGGC
AGCCCGGCCTATGAGGGGCCCTCTCGTCCCGGCCAAAGGCTATTTGCCTTCCTGTCCGGGCACCCTCGACCGCACCCGGGCGGGCGCAGGCTTCATTGCG
GCGCGACTCGCCAAAAGTCCCCGCGATCTCAGCGAGTACCCTGAGCGCTGGCCCTAGTCCGCCTGCCCCCTTAATGTCGCTCAGGTCTGCAATCTCTTAC
GACCGGCTCAGGTAGTGGCCGCACGGTGCGTAGGTCCGCTGCGGGCCGGGCCACTCGCGCCGATCGTGCCGTGCGGCGGCCCTGACACCGTATGCGTGC
GCGCTCCGCAGGCTCGCGAAAATCCTTCGCAGGCACTTGGCCCCGTTGCTAGACGTGGTGTCTCCTAACTGTGGCTCACGCACGGCGTTTAGGCCACGGT
AGCCCGCTGTTCGGTCCTTACGACTCGGCACACGCCCCCGTGCACGCCCTCGGGTTCCGCAGGCCAACGCCTGGCCCTAGGGTTGCGTGCCGGCCTGCCC
GCGATGCCACTTGCCCCACGTCCCCCACCTGGCCGGCGCAGACGGGTTCAGTGGGTGTCGAACGCCATTCAGTCCCCTGGGACTCCCCCGACCCCTCCCA
TGGCACCCGCCGCCTGGCCTTGGAGCTCCTGCGCGTTGGACCTTACTGCGGTCGTCAGCTTCTCGGCGCGCCCCGGACTAGTCCTGGGCGGCCAGCTCGC
GGGACCCTAGCGCGCACGCGTGCCCGATTGCCGTATCCCCTCGACGGCGCCCGGGCATAGGGTTCGATCCCGCTGCTATTGCGGGTGCGTCCAGACCTGC
CGCCGTTTGGCGATCCGAGTGTGCGAAGGGAGCCAGCCAGCGGGCCGCCTGGCCGCACTCCAGGATCCTGGCGCCAAGGGAGAGCCCTGCCAGCGCTA
CGCGAATACTCGACCTGTCCGCGCCCCGCCTCGGGCTTGCGAGATGGCCACTAGACGCTATCGGGTCGTCACCCCGTCGGCCCCACAGGGGCCCGGCTTC
GCGTGGCTTTCTTGTCGAAGGCCCCGAAATCCGGCAGGCCCCGACACCTTCACCGTGAGCGAGGACGCGCGGTGCGGGATGTCCAGTTCATGCACGCCC

CTCCACGGCCGCCTTCGCGCGGACAGCGTCGGGCGTGCCCAGGACGAGAACAGACCCTGGGCCAGCCTCAGGGCCCGGCCTAGGCGACCCTGGTGACC

AGTTTCCACTCGGCACCCTCGGGCTTCGTGGCAGTGCAGTCATGCCCCCGGGTGCTCCCGCGCGACCGCGCACCTCCCACCCAAGGTTCTTGGTCAACGC

GCCGGATCTGACGTCGCGGTCCGGCCCGCGCCGTAGGCAAAAATGGGTTCGGCTCCCGCCACTCCGCCAGTTCGAGGGCGGTAAAGATCTCAGCCGCCG

TGTTTCGGGCTATCTCCCCGCGATCCCGGGCCACAATCGCAGGTGGCTGGGGCGTCGGCCAGACCCGTCTCGTCGGCCTTTCTGCGCACATTACCTCCGAG

CAAGGCAACCGCTCCGCCCCATTACAGGGGGTTGTCTGGTACCTCCGTCACGTACCCCCGCATCGCTGGCGGGCTGACCCAAGCCCCCTCCCATCCCGAC

CCTCACGCGCTCACGCCCCTTGCCGGGCGGGGTCGTGGCTGCCCAGGGTCCAGGGCCCGCCCCGGGGGCCGGTTGGTTCCAGGAACTGAGGGGGGGTCG

CCCAGCTGACCGCTCCTCTCGCGGGGTCTATTCGGCGTGGCCATCCGCAGGTGCGACAATGCCGGCTCCCATTGCCGGGCCGACCCTGGTCCCTGGCGCT

TCGGACTCCCGGGGAGCGCCTCCCTTCATCGGCGGCCCCTGCCCCCGTCATGACCCCCCTTACGCGCCGGTGGTAGCCGCGCGTGGTGGCGTGCCCCTCT

CCATTATTACGCTCCACAGGAGTGGCCACCGGTATGGGCGCGGTGCAGTCGTACATGTGTGCGGCCCTGTTTTGCGAACCCTTCCCGCGGTGACCTCCGCC

CCAGCGGCCTTCTTCGACCGTTAACGCCGGCCAGCACCCGAAGGGGGTCACACAAAGCCCTGCAAGGATGCGACGCTGCGTAGCCGCAGGTTAGCCGCT

TCCTTCCCAAAACAGTGTCAGAGCTGTCCGGCGGAACGTTAACGCCGCCGGGAGGATCTGCGGCTACCCTCAGGCCCGTCCCGTGGGAGTGGCCGGCGT

CGATGGGGGTACGCACCCCACTAGACGCGGATTTTGGCCAACGGGCACGGCCGCTTCCGAGTTCCCCTGCGGTCTGAGTGTTGGTACGTTCAGCCGGCCG

TTCCGTGGGGCAGAGCGCCGCACGCATGTCGCAGGAGCGCGCCCTCCGCGTCTTGCGGGCGGGGTCCTAACGAAGCCCCCTGGGCTTGGAGGTAGGGGG

CGCGGGGGAGCGATGGGAGCGCAAGCGTGCCATCGGGTCCGGCCCATAAGTGTGTATGGCGCACCGCGCTGCCACGATGTCCGGTGCGCGGGCGCCGCT

GGCTAGCTCACTCTCCCCCGACTCCGCCGCCGCGCCCCGAGACGTATGGAACAGCTGGCGCGCGTCCCCCGCCGCGGGCGGCAACGAACTAGATCCACC

GCCCTCACGAGGAAGCGCGCCGCCACCACGTCTCCCCGGACGACGCGGTCCCACCATCCGCTGTGCCGGCGCGCGAGCAGCCTGGCCCTCGGGGCGGTC

GCGCCAGCGCAGACACCCTGTCACGCGCACGGGAGCCCTGGCCACCGCGTCGACGCCCGGGCTAGCCCCGACTATCAGTACTGACCAGAGGGGCGCCG

GTTGTCCCGACTGGTTCCTCCCGAAACGCTGGCGGTGGGCCCGCACGATTGGTGCAGGCCCCCAAGAGGATACGGCTATTCGCGGCTGCCCGAGGCGAG

TCGGGCCAGACCGGTCGCGCGTGTGTGTGTTAGCCCTCCGTCGCGGCCAAGCCGGCGACACCGGACTGGGCGACAGTAGCGTTCTTCTGTCACTAGTCAC

TCTAACGGACAAATGGAAAAATGGCACCTATCGCTACCGACGGGGTTTTCCTCCAGGATAAGCCTCCGCGCCCCCCACTGGACGCCAGCCATCCTTCCCC

GAACGGCTCGCCTGTGCCGGCTCCAACCCCGCGGGACCTCCCTTCGCGGCCCCGGCGGGGATCGGTGGATCGTCGCCGCCGGCGCCTACCGATCGCGCC

GCCACGGTCGTGCACCAACCAAGCACTGCTACACGCCTGTGCGGCCGAGGCCGCCCCATCCAGCAACGGGGCACCGTTGACCCGTCCCCCATGATGCCG

GACATGGTAGCCCCCACACATGCATCTTCGGGTAACCCGCGCTAGGTCCCCCCCCGCGCCGCCGCCGGCTTTCCCCCCGCTGGTGCTAAGCGGCAGATGC

ACCACTGACGGGACACACTCGCCCCCGATCCAGTGCGCTGCCGCCTGCGGCCCCCCACCGGACTGGTGCCTGGCGCTACACGGAGCCGGCCGCGCACTG

TGACGTATGGATGCCCGAGGGGACATCCGCCCCGGACGCGTGCGCAAAAGACCACTCGCTCGACGCCACGACGTGTGCGGCCGGGGTGATGAACGTCG

GCCGGTGTGCCAGTGAGGCTTGCACCTACACTCCCTGGTCCACCGGGCCGTTCGAGCGGCACCGGGCGAAGAACCCGCAGCCTCCCGGCTTACGCACCG

GCCTCGCGTCAACAGGCAACCACGACCGCAGGGCCGGCAGTGTGTGTCGCTGTGGAAAGGTGCGCCGCAGGCGACCCCCACGTCAGCCCACTACAAAG
CGTGGCAGCATGCTGCGCAGCGACCGCACGCCCCGACACAGCCCAATGCACAGCATCGGGGGGGCGGAATCGCCCCACGCCCGTAACATCCCGCGGGT
CGACCGCCTTACACGTCGACTGCCACACCCTGGTGCCGCAGTGGTATCCCGAGACCGCATGACCCGCCGCGAGCCCGCTGACCACCTGTCCTTGATCGCA
GTCGCCACGGGGCCGGAAGGTGTACCGCATACCGCACGTGCTGCGCGTGTGCAGCACGGGGGCACCCTGCATGTATGGATCCCTCTAAGCCGACCTGCC
ACCCCAGGCCAGAGGCTAAAGTCTGCCGCCTAGGCCCCCCCCCGTGGTCGCCAGCCCCGGTGGCGGCCCACCCTCGGGGCGACTGACCGGCCACCATCC
AGGGTAGAGAGGTTTGGCCCTAAATACGGGCGTGCCGAGGAGGTGTTTGGGGCGAGCGGCAGCAGGTTCGGCGGATCCTTGAGGGTGGGGCCTGGGGTG
GGACGGGGGCGGCCGAAACGCGAGCATAGGCCCCTGCCTGGCCCCTTTGCAGTCTGTACTTTTATTGACATGATACTAATACCGTGTTCCGCCCTGAGAT
CGCACGCGGACCTACCGTGCCTCTCTCTGCATAGTGCTTCAAGAGCCCAGCGCGACTGGGGACCGGCTCCCGCAGCCGGCTCGGGGCGTAACGCTCGGT
GGAAGGGTCACCCGGATAGCTCCCGGTTAGTTTCGTACAGGGTGTCGGGGGCGCCCCCAGCGAGTCTCCTGGAGGTGCAGGGACGGCGGTGGTCCTCGA
GGGGGCGGACGTTGGCCGCATAACGGGTAGTCTCCAGGGTCACCTATGGTCTGGCTCGCCGTCAGGGTCTGTGAGCGTCGTGCTCACGCCTCCGCAACAG
CCGGCGACCCCGGCTCGCAACGGCCAGCCGTGGGCGGGGGGGCGACCGGACGCAGTTCCGAGTCTAGGCATGGTGCCACCGCCCTCTCGTTGGGTTAGT
CTCGAATGGCTGTTTCGGGGGTCAGCGCGGCGGGGCCGCCGCTGGGAGCCCGCATAACCGTGGATACACTTGCTGGGTGCGCGCTCGAGGGCGGCCCCC
GAGGGCTGGAAAACCATTTCGGGATCGCCCCCGCGCTCCCGTCCGCACTGCGGGGTCCAGCCCGACGGGGGCGGGTTTCGTTGGGGCAGTACTCACGCC
CACGGCGAGGCCCATCCAGAGAGTGAGGGTGTCGCTGGGGGGGCCTACCGGGCGGAACGCGCCCCGACTTAGGGCGCCGCAATGCACGACCAGTCACC
GTCTGGGCGTCGCCGCGCCGTTGAGAACCCCAACACAGAAAGAGCTTGCCGCCTCTGGGGGAGCCAGGCCGAGAAGCGTGGGCGAGCGGCCACGCTGC
GGGCCCTCCGCGGACCGTGCGGCGCTCTCGGGTGACAAACCTAGTCCCCGTCCCAGGCCATGGGCGAGCAGCTGCGCCGGGACTGTGCGACGCCTATCC
GGCACATTCGGTATTATTGGCCTGGGACGACAGCCGGCGACAGGGCGGACGAGGCGGCGTGGACTCGGGGTGCAGGGACGACGTCCACCCGTACTGGT
CAGTAGCCCAACGCGGAAGCCTGTCTGGGGCTTTGGCCCCTAGACATCGGTCCGGGGCGGGGCGCGTCATCGCTACGTTAGTGAGGGGGAGTCTGCACC
GATAGGGTGTGGTGGTGTTGCTTAGATCGTTGACGGGCCGTGAGGTGGCGCTGCTGTTTTCCGGCTTACGGCTTACGACCTACCGCCCGCCGCGCCGGCCC
GAAGGCGGCGCCCCCGCACATCGCCCTTGGGTGGTAAAACCCGCCCTGCCGCCGCTACCCCCCCCCCAGGGGTGGCCGGAACGGCGAGGGCAGGACAC
GGTCGACGCACGCAGCGCCGTGACGCCCGGCCCGCGCTAGGGTTGGCACCCGTGGGACCTCGACAAACTTTCCGTACGATTGGGGGGCGCATACAGCGG
ACGGGGAACCCGCCCGACGCACGCGCGGTCCCGAGGGGTGGGGGACCTGCAGCTGCGTCGCGCGGTCCGCGAGGGCGTCTTGCTAGACCATGGGCGTT
GCAATAGTGGGAAGGTGCCTCCCAGGCTGCGGGGCGCCGGACGCCCGACACCAAAGCTGTGCGAGGACAGAGAGGCTAGGCACGGATGGGTACAAGC
GAGCTGACAGCCTCCATCCTTGAGTGAGCACGCCGCGGCGAAATGCCGGCCGAAATGGGAGGGGCGCGACACCGGATCGTGATCGAGCGCGTGCGCTT
CGCCCACACGCCCTGACCCGCTGAGTGTCTCGGGTTTGAACCCCGTGCCCGGGGGCGCAGTGGGATAGGCACGACGGGCAGAGTTAGGGGCCTGGGCAT
AACGGCCCTGTCCGCGGCCGAGCGGGCCCGGGTCTTCTCGACCAGGGTGCCGGCCCCGGCGGGCCCCCCGCCCACGGACGGGGACCTGGAGTTGAGCC

AGGCCACAGGCGTGACCGGAATTGGAGCGCCAGGGGACAGCGGCCGCCCGCGCCAGGCCACGCCGATGGGGGCGTTCTGCCCAAGCGTCGCGCAATGG
GCGGACCGGTCTACGCGACACCGCCCGCCCGGCCTGGAACTCCCCCCGAGCACATTCGATATGGTCCGCATCAAGGCGGGAGGCATCGCCGGGGGCCG
CGCGGACCACCACCTTCGCGGGGGACGGTGCGGGTCGCGACGAGGCGGCGGCATGCGCGCGGGTGCCGTGAGCCCTAGAGTCAGCGGTTGCTGGTATG
GGCGGACGGGCGCCCCCGACCTGCCGTGGACGCAGGCTCGCGGACTAGTCGACCCGCCATGGGTGCTGTGCGTGACGTTGGCCTTGGTCCAGGGCGCGA
CGCGCAATACCGCGCGGGCTGAGTATCGCGTCCACGGACAGGCGGCTGAGCCGCCCGGCGTAGCTGGTACACGGTCGGGCCGAAGTGGCAAAGAGCCG
GCTTGCGCCCCCAGCGCCGTTGCAGCAGGTCTGGACAGGCAGTACGGAGCGAACGCGGCGAGACACCCGGCTCCGGGCGTAGTCGGATCCGCCCGCCA
GCGGGCGCGCAGCGGTGTCGCGGGCTTGATGCGCCGGTCAGGGAGGTCGTCGCGAATGAGCCGGGTGGAGGGTGCAAGAAGCCAGACGATGGAGCAC
CGAGTTGTCCGCGTGCGATTGCCGTCCCGGCGAAAGCGCCAGGCCGCGGCGCGTGTGGCAACGGCAAGTACCCGGAAACACGGCACGGGTCTCGTGGC
GCCGCGAGGCGGCGGGTGGCGCGCTCCGGGGGGCTGCGCAGCCCCTCAGCGCCGCTGCTAGTGCCAGACGCTTGCCCTTCTTGAGCGCGGCGGCGGGAA
CTCGGTCGCGTGCGTTCAGGGCCCTCATCCGCAGACGTGAGGGGTGCGGGCCAGGGGGCGCAGAGGCGCCTTCCGAAACCCGTAAGGGATGTTCTGGCG
TTGGGGGGCCGCCGTCTTAGGGGGGTAGCGCCGCCTCAACTCCAGTATCGCCGCAAGGCCGGTGCCAGGTTCGATCCGGGGGCGGCGGCGTACCGCGTC
CACGCGTCTCCCAGAGGAGGGTCAAGGTGCATACGGGAGGGATCATACGGCAATCGGCGGCCGGGTTCCGCCATCTAACGTGGCGATCGCAGCGTACC
AGAGCGAGGCCTATGGGCCATATCCCTGGCGGGAACGGGCTGCTTGCGCCCCGTCCTCGCCGACCCTCCGTACCGGTACGGGGCGAGGATCCCGACACA
CAGCACGGGGGCGGCGCGCAGCACCCACCCCGTCCTCGGCCCAACAGACAAAGACCCCGCGGCGGGCTGGGGCACCCCCTTTCCGGCTACGTCCACCG
ATTCTGTACAGGACAGCCGTTGCGATGGGCACCGGCGATCGGAGAGAAGGACGCCACAGATCAGCAGACGACTCGGGGAAACCACGGGACCGCGCGTC
GCGTCAACTGCCGCGGGAGTGAGAAGGGAGCGACAGGAGCGCGGAGGTAAGGCCAATGGGGCCAGTTGAAGCCGCCCACAGAGGCTGTGCGGGGCCC
CGCCGGGTTTAGGAGCTGTTCTTGGAGCCTGTCGTTAGGGGCGGGGGGGGGCTTACACGGCGCTGGGGGACCCACGAGGCGCAATCCGCCGCGGACTGC
TGGAGGGCTACCTGGGGCAGCACCCCAGGGGCGGCCATGGCGCTTGTTGACGGGGGCGCTAGGACCTTGTCCTAGGGCGCGCCGCTTTCGCCCACAGGG
CTTCGGTGGGCAGTCTATCTGTGCGGTTTCAGTCTCGTACGCACGTGATGCGGTGTAGCCTGAGCCCGGCAGTTGTCTAGAGTAACTAGCGCGCGGTTATG
GCTTCGTCTGGAAACGGGCTGCAATGTTCCGGGTGTTCATCACACCACGTTCCGCTTTGCAGCTCACCCCCCGACCACACCCGGATTCGGACCGGTGGTC
CTTCCTCTGAGGTACTCGCCGCTCATACAACCGCGGTTCCCGCTGTGGTCGGCACGGCCCGGGTTGCAGGGGCGTGCCTTCCCGGGTAGTGTGCCTGCGC
GGCGTGCGGCTTCCGCAGTCCACGGACCGTTCCGGGGGTGAACGATGGAGGTGAGGGGACGAGTCCCTCCGAGATTAATGCCCCGCGGCAAGCCCGATC
AGCGCTGCGGTCCTGGGCTGTCAGCGCGCGCGGAGGAGCGCCCGATCGGGCGCGGGGGAATGAGAGTGTGGAGGATCATAATAGAGAGATCGCCCCGG
AGACTGCGAGGGGCATGGACGCGGAGGGACGGGGCGGGGGGTCGGCACGCCCGTGCGGCCTGTCGTCATATCCCACGCTGTCACTCGGCTGCGTCCGGC
ACTCCGGGCGGCCGGCGAGCAAAGCCGCCCTGCGCGGCCCGATCGAGGGTGCGGCGGCGCGACGTCGTGGCGTGGGAAATGCGGCGAGGGGCGGGGT
GGGGGACGTCATTGAACCTGCAGCGCGCACGGTGAGTACCGCGGCCCGTGGCGGGGCTGTACTGCGGGACCCCGGAGCGGTAGCGGGGGTTAGACTGG

GTGAGGGTAACCTGCTGCCCCGCGCACCACGTTCTTGGCCAGAGGCCCTGTGGGTGTTGGAGCAGGGTCGAGTATCCCTCCGTGCTTGCCTGTGTGGAGT
GGTTCGCCATGCAGTTGGACTCTTGGCGTCAGCTGGGGCCAGGCGGCGCCTCCGAGGTCGTAAAGGTCAACAGCGCTGTGAGATGGGCGCAGGAAGAGC
GCCAAAGACCTCCTGCGCCCCGATGTCCGCCTCGGGCGGACGTTCGGCCGCCTCGGGACTAGTTAAAGTGCGGCTTGGCTGGGGGCGCGGGACTCGGAA
GTCCGGGTGGAAGATGCGGGGAAGCTGCCCGTCGCCGGTACTGGGGGACTGTAGCTGTGATCCGGGACCACGAGGCATGCACCCGTCGTGGTCCAGCCG
GCCTGGCGCGGGGTCACAGAGCGAAACGGCGGACGCTAGGAACATTCGCTACCACCCCCCGGCGGCACCTTGCAGGGTCTACCCCAGTAAGGACCGGC
CCCAGCAGTGAGTGCGCCCGGGGGCTGGCAGCGGTAGATTGGGTCGCACGAGCTCACGTGGGCCGTTGACCGGAACCGGCAACGCCTGGGACTGGAGC
GCAAACTGGGGTACACTCTTCACGCCCGCTGCTCGTCATCGACCCCGGCGGGCGCCCGGGACCGGACCGGGGGCGACCCACCGGGGGACGGCGCGGCC
CCTGGATGGCCCTGCCCTCGGTCACGAAGCGGCCGAATGGTTTGGGGGGCCTCGGGACGCGGCCGTGGAGGTGCGGGGGGCCTGAAGTCCCTGCCTGGC
CCTCCTCCTAACGGCTTCACGCCCCTCAGGGCAGCTAGGGATTAACGTGGCCGGGTCACACGACCCGCCCAGCGCGGTACGCTGGGCCGCAGTGATTGC
GCGCGCCTGGCGGGTCCTCACCCCCCCACACACATCCGGGTCGGGGCGGAACTGGGTCCCGCCGTTCCCTCAGATCTGCCCCCTGACTTGAACATGCCCA
AAAACCGAAACCGGGCCGGCGCCCAGACCCCCGTGCTAAGGTTCCCACCGCCGACCCTAGCCCCCCCGGAGGTCCCCTGTACTCCGCACGCGACGTCGT
ATCAGGTCCCAGAGCCCGACAATGCATGCGCGTCCCGCTCTGCGCCATGCCCATTGCCCGACCAGCCCCGCCCAGCGCAGAGGCCCCGACTGCTGGCGC
CCTCTGTGGCGCTCGGCCGCTCGAGAGCACCTGCCGCCCGACCGCAGTCTGGCTACCTGCTCCACGGGGCGGCGCTCGTGGTCTCCCGTACTCGGAACGC
CACGCCCCGGGCTCGCGCGTCTCGCGGTCCGACGCATCGTGGTCTTACCTCCGACCCTTTCCTGGGGTCGAGCGCGGGGGGGCAGAGCGCGCTCATCCAG
CCCCGCGCGGCAGCACCCGCCCGCCCAGGGTGGTACCGCTCTCGAGAGTCCGGACGGCCGAACGCCAGGGCAACGGGCCGTGTGAAGACTCACCGTTT
CGGCCGACCGCCATCCTAGCTGGGGCGAGCCCCCGCGCGCGGCCGGATGCGTATGACGTGCGCGCGCTCGGTGCGATGGCGCACGTCGACTGGAATAAC
CCCTTGCCAACCCCAGCGCCGAGAGTCCCTCTTCGGGTCTCCCGTCTGCCTCCGATGGGTGGCCCCTGCCGCTACTTGTTAGGTCCTGGCTCGCCGGGCGC
CTCGTCACCTCGGCAGCCTGGATCGCTTGTCGCCGCCGCAAACGTGCGCGCGTTTCGCCACGGGCCGCCCCGCGAAAAGCGGCGGGAACGTGGACCACG
CGCGCGCCCGCCATCGGCTCGCCGGGATCCCCACCGCGGCAGCGCCCCGCCACAGCTCAGGACGCGGCCGATCGACAAGCACATCTCCCGACTACCGG
GCCCCGCGGCGTGAACGTGGTCATGGAAAACCCGAGCGTGTGCGTTCCTGTCACGGGATCCCGCCGTGGGCAGTTCGCGGCAGGGGCCTACCCGTCGAG
GGGCTACACCGGACCGCCCCTCCAGCTGCATCGTGCAGCCCTAGTGCTGCAGCGAACTTGCCCCGGCCATCCCGTCCGCCCGCAAGCGGCTGGGGACAG
GTTCTCCGCCCATGCGCAGCCAGTGGCCTCATTCATGAGGGCGTGGCGGGCCGGGCCGCGTTGGGCCATCGCCGGTTGGGGGCCCCCACGCCGTCTACCT
GAGTGGACGCGCCGGGCGAGGCTCCCACCACCCCAGTCCTGGGGTCCCAGACGGCCTCCTGGGCGGAGTGTTCTACCCCGCGGCCACCCCCAGCCCGTC
TGCGGCCGAGGCCCTACTGGAGACTGCATTCCCCGCGGCCGGGGAACCGGCCACACCGGCAACGGCGCTCCTGACCCCACCCGCCTGCCACACTCCCCG
CGGCGCGGAGCCCCACCCATACTAAGGCCCCTACGCGCGGCCGACGACATGTTGATGGCTTCGCTGGGGGGGCGGTGACACGTCCACGAGGCGCCTCGC
CGCCGTCGGCCCTGCCCCAGCACGTTCCCCCGTCCCCCGGGGGTGCGGCGTCCTGCTCGCCCCCCGTGTAGGAACGCCGACCGGGCTGCGTCGTCGATGA

GGCAGTGGCCCGCTACGCGGCTCCTCTTCGCACGTGCTTTTGGCTGAAGAACGGGCACCCCCACCATTACGCAGTTGGGACGGCGCGCGGCTGCGAGGG
CCCAAATCTCTCCCATGCGAGTCGTGGTCGCGCCCGTGGACCTCTGAGGGGCAAGGCCGCTCGTCTGCTGAGCGGGGATGTTGCGCCCAGCAAATCGAG
GAAGGTTTGCTCGTAACTTACGGGTACCGAGACTCCTCGCTTGGCTCACCCATTGCGGCAGCCGCGGCCGCCGCCGGGACCAGTAACGGCGTGTCGACC
GCCCCTCGACAACGCCTGGTAGGACGCTCCCCTCCCGGCCCGGACACTAACCCCCGCAACAGCGAATTCCAGGGGCGTCTTCCGCCCAACAAAGACCCA
TCGTGCACGAATTGGGCTGGCGGGCAAGCATGTGCATCACGGACTAGGGTACCGCCTGCCTCCCCGCTGCCTCCGGGGCCCTGGCGCTCCGTCCGATTTT
ATCCCGCTCTGCTGCGATACTCAACCAACCGTTTCGCTTTATTCAGGCCGCCTCTGGGGCCCGGCCGAACACTTTCTCCCGACAAGTGGCGTGACGGACCT
GCCTTCGCGCCGCCCCGAAACAGGCGGGCCGGCCTCGCACCCCTGGGGAGCGGTCCGCCGCAGGCGGGCCTCCTGAGCGCGCGGGTGGCCGGCCAGCG
CGCATACCCCCTCGTCGCTGCCGCCCCCCCCGCCCCGATCCCCGGCCCCGCTCGGCAAGACAGTGGAACCCGCCGCGCCCTTCACTCGCCCGGCTGGCCG
TATTTCGTGGGTCCTCACCTCAGTCGCGTCTGACAGATTAGACCTCGCCGCTGATGCCTAGGGTGGGACACCACCTTCCCGCCCGCCACCACCCTGTGGA
GCGTGAGCGCACTTAAGTCTCCCTACCTCCCCAACTCAGCAGCGGCCCCAGGGCATCCGCGAGGCTCCGTTGACGCACGTGCCGTCGTCCCGCGCCCACT
GTGGCACCTCGGCGGCGACCCTCCGCTGCGGGATGCCTGGAGGAGCGGTCCAGCCTGGGCGCTCTGCCGGTCACCAACGGCGGCCGGGCGGGGGCCCTC
GCGGTCGGACCGAACTTCGGAGGGGTCCCCGGCAGCTGAGTCGAGCGCTGCTAGTGACCTCCACCTCGGCCTAGTCACACTTGCCCGGCTCCCAACCGCT
AAGACATAGGGGGCGTCGCGACGAGGGCAATGCGGGGGCAACCTTGCCACCCCCAAGCGACGTCGCCTCATGTAGGTTGGAGGGGCTGTCTTGTACTCC
ACCGAGGGCGACACGTAGCGTGCGGCGGCTCGACTCGAATCTTGCGCCTCGGTACGCGCCACGCGACCGATGACCTTTGCAGGCGACTCTGCGAGAATG
GGGCCCGCGTCCTGGGGCGTGCTAATTCACGACCGCCGCCGCTGGCCTCCAGGAGGTCCCAGGGGGCAGCACACGCCATTCGCGTCGGTGGTCTAGCCT
CGCGGCACCTCGACGAGTCCCAGGACGCGTCGGGCCCGGTTCTTCCTCTTCCTGTGTGGCAGTCCGGCCGGGTGACGCTTCCGCCGCTGGCTAATTCAGA
CCGCCGTCAGTCGACTGGGCTGCCCTAACCAGCCAGGGCACTCGTGGACTTCCGGCGCCGTCCGACTCCACGCGGCGCGCACAGTCGCCTCCCTCCTTCG
TACCATTCGGCCCTCCTCACCCCAGGGGGGGCTTGCCGCCATTCCCTACGCTGCCATTCGACGTCCCGCGCTACTCACCCATAGTGCCAATTGCGGCGTCG
CGGACACCGGCCTGGCGCAGCGCCAGAGCGCCACCTGTGACGTCGCTCACCGCGGGGGGAGCCCAGCCTCGACGCCCCAGATGGCTCGCGGGGGGGGA
GGTCCCGAGGCCACCCTTCCAGGTGGCACAGATGCCCCTCCCTGGAGGGGGTGGAAAGCGCGCGGCGGGCCCAACACCGTGGGCGGCAGCTGGGCATG
CGCTCGGATAAGAGCGCCAGGATGGCAGGAGGGCCGATCTGGCGGGTGGTGGCCAGTGCGGGGCGGGGGGCGAAACTGTCTGGGGGTACCTGGAGCCC
AGGGTTCAGGGTCCGTGCCATGAGGCTGTCAGGCAAGCCAAGCCGTTAACGGCGGCCAGCGAGTGGGCAGGGAAGGTGACTAGCGCGAGCTACATATT
GCCGGCCGCCGCGCGGGGCGGCCAGCCGTGAGGAGCAATCTGAGGGACGGCCCACCGCCGGAGGGCCTGCACCATGAGGTGAGGCGGCGGGTCCCCG
CCACGCCGCGAGACGGTACGACCCGTTCCGGATGTGCACCACCGATGAAGAAGGCGCGCACATGCCGTTCGCTTTCGGCGTATGGTCTTCGCAGCCTCCG
GTGGGAGGCGCAGCCCGGAGGGTGCCCGCGCCACACGGATCAATGGGTTGGAGGGGCGGACTCGTGGTTTCGACCATTGGTGCAGCTAGAAACCGTCCC
AAGGCTCCACTCGATCGCACTGCCGTCGATCTCGGTTCTGACTCCCACCTATGCCAACACGGGAGTGCGCTATGAAACGCGGTTTTATAGTCGGTAATCG

CGACCGAACGCGGAGCGCCGTCTCGAGGCTTGTAACGCTCCGCGGTCGTTGTGGAGCGGACCGGGTAGTCAGCGTGACGTATTTCTAGCCCCCAGTGTGC
CGCCCGGCGAGAAAGTCCTCGCGGGAGCCCGTCGGCTGCCGCTGCGTACGCGGGGGAAAGCGGCCGAGGTACGTCTTCCTGGTGGCTTGCTGCCGCCAA
CCACCGATCGGCCCGGCTCGATGACAGCCGGCGAGAATGGGTTGGGGCCTACTTGGCCGGGTTGGCTGATCGTACGCAAGCAGCGTGCTGCGGGCCCAC
GCAAAGGGGAATGGCGGGGGAGCGCCGGCTGCCAATTCCCCCCGGCTGCACAGGCATGATCCGGTGTCAAGAACGGTCTCCGGCCCACGCGGCCGCGC
CTTGGCCCGAGTACGGCTGACTCAGAAGCCATTGGGTGCGAGGGCGTGGGGTGGTGGCGCATAGGGAGCCAGACCCGACGGAGCCAGGATGGTTCGAG
CAGTGTCACGGCCCGGGGTCATGTGGGGCCCGCCCGCACGGATGACCCGCGTCTCGATAATCGGTTGCAGGCGTGCCGTTCCCACGGGGCGCGCCGGTT
ATCGAACGGGTGCGCGCGTAGACTCGCTTCCGGGCCCCGGACCTGGGCCAGGCAGAAGCGGGGATTACCCGTCTTGATCGTAGACAGCCCGGTGCCCCC
GGGGGCCGACGTACAGCTGGCAAGCACCGGTCCGGCGATTACCCGACCTGGGTTGGCAGGTACACGTTACAGGGGTGGCGTGCCCCCCGGCGCTTGGCG
CCGGCTTGGAGGGACGGGGGGGGATCTAGCTCAGATGGCCAGCGAGCGTAGCTTCGACGGTGGCAAGAGCGAGCGGCGGCGTGCTTGGGGAGGCCGCG
GGCGGGTGCGACGTCCAGTTCGTCACACGCATGAGGGGTTTCCGGGGAGGAATCACCGCCTCGCGCAGGCGTGTCCGACACGACCCGTGTAGCGGGTGT
GAGACATTTAGGCGGAGGCCAGAACCCCTTGCCGGAGGGCTTGTAGTATATTGTCAATGGGGCCGACACCACGTGCGGATCGGGACTGAGAGCGCGTAG
AGCCTTGGCCCTTGCTATATTTGTGGAGCGGACACTGTGCTTCCCACACGTCACGCTGGCGTAAGCCGATGCCCGTCACCGCCCGCGCCCCAGGCCGAGT
GCGGGGGGCCGCAAGGTCCTGAAGCCGATCTACGATGGGCTGGGTGGGCGACGTCGTGGGCTAAGCTCGGCGGCCCCACCGTGAGGCGGACGTGGGGG
CCTGTACTAGAGATGGAGCGACGGTCGTCGATACAGTTCTTGCCAGGAGACCGAGCATTTTGCTAGGCACGGGCTGCATGGGCAATTCATTAACGGCGG
CGGGCCCACCGCGGCCAGCGGTGGCGGGGCGTAGTACCGGTCGGCAGGGATACGACCCCGGGCCGAAATTCGGGGCCGGCGGCCAGGGGGCGGCGCG
CCTGCGCGCCGTGACGCTGACGCCCTCCCGGGGTGCCGCGGGCTGGCCTGAGGCCCAAGGAGGCGCCTGGGGGTCGAGCGCGGGCCCGGGCGCACGCT
GATCCCGAGCCGACGAGCTTACGGATTGACCAAGAGCACCGCGAGCGTCCGAGCGCTGCGGGCGTGGTAACAACTACATCCCGGGTCGAGTTTGCGGCT
GGGACGTGGGATTGGCCGTGCAGACGCGCTGGGGGGGGAGCGGGGCTGGCCCCTCGCTCCGTATTCCCCACTCCACATGGTCCCCGGGCGTACGGGCGT
CCCCGACGGTTCGGGACGCGGGAATCGCGCAGGTGCCCCTCGGTGCGTGGCGGGCTGCGTGTAAGGCGCTTTCGCTGCATGCGGCCGTCTCGCGGACGT
GGCGTACGGGGGCGACCAGGGCGAATCCAAGGTGCGCCCAACGGGCCGAGGCGCTACAGATGGGGAGCCCCGGGCGGCCGTACGCAGGAGTCCAGTG
GCGTATGGAGGGAGGCCTGGGGCTCCGCAACGGCCGAGGCTATGCCCAGACCCCATGCCATTGTAGGCCGAACGCGATAGGTCACGCCGGTGATCCTGC
CGGGGTCGCGGGCCGGATCCACAAGGCCAGACCCATAGCGTAGTCTATAGGCGTCCGGGAAGGGTCGTGGGGGGGCTAA

>2017.TE.25009.1.4

CACGCCCCCTCCCGGCCAGCCCGCCCCCCGCCCCAGCGTCGTACCCGGCGTCGCGACTCCCCGCCGTCGAGAGGCCTTTGCCGGGGCCCCGGCTAGCC
CGGGGCGAGCGGACCGCCGCGCGCGGGGGGTGTCACCCGGGGCACACCGCGCCAGCCTCCCGGGCATGCCACGCCGGGTTCCGCCCCCGCTGCTCCAA
TTTGGGGGGCCCCTAAGATCCCGTTCCCCAGGCCAGCCGCGCCCGCGGGCCAACATTGCCACACCGTGTCGCCCCCCCGCCTGCCGCACTGACCGTCTTG
CGGGCCCTGCTCCCTGGCCCGAGCACACCTCCGGGGACCCCCCTCGCTCGGCCGGGGGAGCCTTAAGCGCGGCACCGAGTAGGGGGTCGCGTGTTGCCG
CCGCGCTGACGCCCCGGCTGGGACCCCTTTCAGGTCGAGACGCCGGGGTGGCCATGCGGGTCCAGTCGCACGATGGCGCGGCGATGTGGCGCCGACTGC
CCCGCCTGTCTTCTCCGTGCCAGCCCCTCGCCACCATGCCGGTCCGGCCCGCACGGGGGTAGGAGGTGCTGCCGCGGCGGGAGGCGTAGGACGCCATAT
CCGAACCGGCGGGTCCCCCCGGCCACCGAGTCGACAGGACCATAGGCACCGCGTCCGGGCGGGCGCCCTTAACGCCGCCCCTTACTGGAGAAGCAGAG
CTCTGCCGAGGCACCCTGCCTAGCTGGCTGCGTCCCGCACCCCGCGATCCCCCTGGTCCACTTACCCAGCGGGATGCGGGGATGTTCGCAGCTATCACAC
GGTCGTGGCCTGCCTCCCTGGGCCGGCGAGAGCACCGCCACCTTCGTGTTCCTTGCCGCCGGGCGACGTGTCCGGGAAAGGCTTCGCCGCGCGCCCGCGC
GGAGCGCCGGGGAGTCCCGTGAGCGGCCGCAGGAACGTCACGGACCCCGGCTTGGTGCGGTCCCCCTCAGTGGTGCCCGACCAGAGACCGCGGAAAGA
CTCCAGGAAACGCCTCGGGGGGAAAGCAGGGCCGCGCACTTCCGCTCCACGCTAGCTGGGGGGACCACGACGGCAATACTGAACACCTGGCTCTTCGG
CAGCCCGGCCTATGAGGGGCCCTCTCGTCCCGGCCAAAGGCTATTTGCCTTCCTGTCCGGGCACCCTCGACCGCACCCGGGCGGGCGCAGGCTTCATTGC
GGCGCGACTCGCCAAAAGTCCCCGCGATCTCAGCGAGTACCCTGAGCGCTGGCCCTAGTCCGCCTGCCCCCTTAATGTCGCTCAGGTCTGCAATCTCTTA
CGACCGGCTCAGGTAGTGGCCGCACGGTGCGTAGGTCCGCTGCGGGCCGGGCCACTCGCGCCGATCGTGCCGTGCGGCGGCCCTGACACCGTATGCGTG
CGCGCTCCGCAGGCTCGCGAAAATCCTTCGCAGGCACTTGGCCCCGTTGCTAGACGTGGTGTCTCCTAACTGTGGCTCACGCACGGCGTTTAGGCCACGG
TAGCCCGCTGTTCGGTCCTTACGACTCGGCACACGCCCCCGTGCACGCCCTCGGGTTCCGCAGGCCAACGCCTGGCCCTAGGGTTGCGTGCCGGCCTGCC
CGCGATGCCACTTGCCCCACGTCCCCCACCTGGCCGGCGCAGACGGGTTCAGTGGGTGTCGAACGCCATTCAGTCCCCTGGGACTCCCCCGACCCCTCCC
ATGGCACCCGCCGCCTGGCCTTGGAGCTCCTGCGCGTTGGACCTTACTGCGGTCGTCAGCTTCTCGGCGCGCCCCGGACTAGTCCTGGGCGGCCAGCTCG
CGGGACCCTAGCGCGCACGCGTGCCCGATTGCCGTATCCCCTCGACGGCGCCCGGGCATAGGGTTCGATCCCGCTGCTATTGCGGGTGCGTCCAGACCTG
CCGCCGTTTGGCGATCCGAGTGTGCGAAGGGAGCCAGCCAGCGGGCCGCCTGGCCGCACTCCAGGATCCTGGCGCCAAGGGAGAGCCCTGCCAGCGCT
ACGCGAATACTCGACCTGTCCGCGCCCCGCCTCGGGCTTGCGAGATGGCCACTAGACGCTATCGGGTCGTCACCCCGTCGGCCCCACAGGGGCCCGGCTT
CGCGTGGCTTTCTTGTCGAAGGCCCCGAAATCCGGCAGGCCCCGACACCTTCACCGTGAGCGAGGACGCGCGGTGCGGGATGTCCAGTTCATGCACGCC
CCTCCACGGCCGCCTTCGCGCGGACAGCGTCGGGCGTGCCCAGGACGAGAACAGACCCTGGGCCAGCCTCAGGGCCCGGCCTAGGCGACCCTGGTGAC
CAGTTTCCACTCGGCACCCTCGGGCTTCGTGGCAGTGCAGTCATGCCCCCGGGTGCTCCCGCGCGACCGCGCACCTCCCACCCAAGGTTCTTGGTCAACG
CGCCGGATCTGACGTCGCGGTCCGGCCCGCGCCGTAGGCAAAAATGGGTTCGGCTCCCGCCACTCCGCCAGTTCGAGGGCGGTAAAGATCTCAGCCGCC
GTGTTTCGGGCTATCTCCCCGCGATCCCGGGCCACAATCGCAGGTGGCTGGGGCGTCGGCCAGACCCGTCTCGTCGGCCTTTCTGCGCACATTACCTCCGA

GCAAGGCAACCGCTCCGCCCCATTACAGGGGGTTGTCTGGTACCTCCGTCACGTACCCCCGCATCGCTGGCGGGCTGACCCAAGCCCCCTCCCATCCCGA

CCCTCACGCGCTCACGCCCCTTGCCGGGCGGGGTCGTGGCTGCCCAGGGTCCAGGGCCCGCCCCGGGGGCCGGTTGGTTCCAGGAACTGAGGGGGGGTC

GCCCAGCTGACCGCTCCTCTCGCGGGGTCTATTCGGCGTGGCCATCCGCAGGTGCGACAATGCCGGCTCCCATTGCCGGGCCGACCCTGGTCCCTGGCGC

TTCGGACTCCCGGGGAGCGCCTCCCTTCATCGGCGGCCCCTGCCCCCGTCATGACCCCCCTTACGCGCCGGTGGTAGCCGCGCGTGGTGGCGTGCCCCTCT

CCATCATTACGCTCCACAGGAGTGGCCACCGGTATGGGCGCGGTGCAGTCGTACATGTGTGCGGCCCTGTTTTGCGAACCCTTCCCGCGGTGACCTCCGC

CCCAGCGGCCTTCTTCGACCGTTAACGCCGGCCAGCACCCGAAGGGGGTCACACAAAGCCCTGCAAGGATGCGACGCTGCGTAGCCGCAGGTTAGCCGC

TTCCTTCCCAAAACAGTGTCAGAGCTGTCCGGCGGAACGTTAACGCCGCCGGGAGGATCTGCGGCTACCCTCAGGCCCGTCCCGTGGGAGTGGCCGGCG

TCGATGGGGGTACGCACCCCACTAGACGCGGATTTTGGCCAACGGGCACGGCCGCTTCCGAGTTCCCCTGCGGTCTGAGTGTTGGTACGTTCAGCCGGCC

GTTCCGTGGGGCAGAGCGCCGCACGCATGTCGCAGGAGCGCGCCCTCCGCGTCTTGCGGGCGGGGTCCTAACGAAGCCCCCTGGGCTTGGAGGTAGGGG

GCGCGGGGGAGCGATGGGAGCGCAAGCGTGCCATCGGGTCCGGCCCATAAGTGTGTATGGCGCACCGCGCTGCCACGATGTCCGGTGCGCGGGCGCCG

CTGGCTAGCTCACTCTCCCCCGACTCCGCCGCCGCGCCCCGAGACGTATGGAACAGCTGGCGCGCGTCCCCCGCCGCGGGCGGCAACGAACTAGATCCA

CCGCCCTCACGAGGAAGCGCGCCGCCACCACGTCTCCCCGGCCGACGCGGTCCCACCATCCGCTGTGCCGGCGCGCGAGCAGCCTGGCCCTCGGGGCGG

TCGCGCCAGCGCAGACACCCTGTCACGCGCACGGGAGCCCTGGCCACCGCGTCGACGCCCGGGCTAGCCCCGACTATCAGTACTGACCAGAGGGGCGC

CGGTTGTCCCGACTGGTTCCTCCCGAAACGCTGGCGGTGGGCCCGCACGATTGGTGCAGGCCCCCAAGAGGATACGGCTATTCGCGGCTGCCCGAGGCG

AGTCGGGCCAGACCGGTCGCGCGTGTGTGTGTTAGCCCTCCGTCGCGGCCAAGCCGGCGGCACCGGACTGGGCGACAGTAGCGTTCCTCTGTCACTAGTC

ACTCTAACGGACAAATGGAAAAATGGCACCTATCGCTACCGACGGGGTTTTCCTCCAGGATAAGCCTCCGCGCCCCCCACTGGACGCCAGCCATCCTTCC

CCGAACGGCTCGCCTGTGCCGGCTCCAACCCCGCGGGACCTCCCTTCGCGGCCCCGGCGGGGATCGGTGGATCGTCGCCGCCGGCGCCTACCGATCGCG

CCGCCACGGTCGTGCACCAACCAAGCACTGCTACACGCCTGTGCGGCCGAGGCCGCCCCATCCAGCAACGGGGCACCGTTGACCCGTCCCCCATGATGC

CGGACATGGTAGCCCCCACACATGCATCTTCGGGTAACCCGCGCCAGGTCCCCCCCCGCGCCGCCGCCGGCTTTCCCCCCGCTGGTGCTAAGCGGCAGAT

GCACCACTGACGGGACACACTCGCCCCCGATCCAGTGCGCTGCCGCCTGCGGCCCCCCACCGGACTGGTGCCTGGCGCTACACGGAGCCGGCCGCGCAC

TGTGACGTATGGATGCCCGAGGGGACATCCGCCCCGGACGCGTGCGCAAAAGACCACTCGCTCGACGCCACGACGTGTGCGGCCGGGGTGATGAACGT

CGGCCGGTGTGCCAGTGAGGCTTGCACCTACACTCCCTGGTCCACCGGGCCGTTCGAGCGGCACCGGGCGAAGAACCCGCAGCCTCCCGGCTTACGCAC

CGGCCTCGCGTCAACAGGCAACCACGACCGCAGGGCCGGCAGTGTGTGTCGCTGTGGAAAGGTGCGCCGCAGGCGACCCCCACGTCAGCCCACTACAA

AGCGTGGCAGCATGCTGCGCAGCGACCGCACGCCCCGACACAGCCCAATGCACAGCATCGGGGGGGCGGAATCGCCCCACGCCCGTAACATCCCGCGG

GTCGACCGCCTTACACGTCGACTGCCACACCCTGGTGCCGCAGTGGTATCCCGAGACCGCATGACCCGCCGCGAGCCCGCTGACCACCTGTCCTTGATCG

CAGTCGCCACGGGGCCGGAAGGTGTACCGCATACCGCACGTGCTGCGCGTGTGCAGCACGGGGGCACCCTGCATGTATGGATCCCTCTAAGCCGACCTG

CCACCCCAGGCCAGAGGCTAGAGTCTGCCGCCTAGGCCCCCCCCGTGGTCGCCAGCCCCGGTGGCGGCCCACCCTCGGGGCGACTGACCGGCCACCAT
CCAGGGTAGAGAGGTTTGGCCCTAAATACGGGCGTGCCGAGGAGGTGTTTGGGGCGAGCGGCAGCAGGTTCGGCGGATCCTTGAGGGTGGGGCCTGGG
GTGGGACGGGGGCGGCCGAAACGCGAGCATAGGCCCCTGCCTGGCCCCTTTGCAGTCTGTACTTTTATTGACATGATACTAATACCGTGTTCCGCCCTGA
GATCGCACGCGGACCTACCGTGCCTCTCTCTGCATAGTGCTTCAAGAGCCCAGCGCGACTGGGGACCGGCTCCCGCAGCCGGCTCGGGGCGTAACGCTC
GGTGGAAGGGTCACCCGGATAGCTCCCGGTTAGTTTCGTACAGGGTGTCGGGGGCGCCCCCAGCGAGTCTCCTGGAGGTGCAGGGACGGCGGTGGTCCT
CGAGGGGGCGGACGTTGGCCGCATAACGGGTAGTCTCCAGGGTCACCTATGGTCTGGCTCGCCGTCAGGGTCTGTGAGCGTCGTGCTCACGCCTCCGCAA
CAGCCGGCGACCCCGGCTCGCAACGGCCAGCCGTGGGCGGGGGGGCGACCGGACGCAGTTCCGAGTCTAGGCATGGTGCCACCGCCCTCTCGTTGGGTT
AGTCTCGAATGGCTGTTTCGGGGGTCAGCGCGGCGGGGCCGCCGCTGGGAGCCCGCATAACCGTGGATACACTTGCTGGGTGCGCGCTCGAGGGCGGCC
CCCGAGGGCTGGAAAACCATTTCGGGATCGCCCCCGCGCTCCCGTCCGCACTGCGGGGTCCAGCCCGACGGGGGCGGGTTTCGTTGGGGCAGTACTCAC
GCCCACGGCGAGGCCCATCCAGAGAGTGAGGGTGTCGCTGGGGGGGCCTACCGGGCGGAACGCGCCCCGACTTAGGGCGCCGCAATGCACGACCAGTC
ACCGTCTGGGCGTCGCCGCGCCGTTGAGAACCCCAACACAGAAAGAGCTTGCCGCCTCTGGGGGAGCCAGGCCGAGAAGCGTGGGCGAGCGGCCACGC
TGCGGGCCCTCCGCGGACCGTGCGGCGCTCTCGGGTGACAAACCTAGTCCCCGTCCCAGGCCATGGGCGAGCAGCTGCGCCGGGACTGTGCGACGCCTA
TCCGGCACATTCGGTATTATTGGCCTGGGACGACAGCCGGCGACAGGGCGGACGAGGCGGCGTGGACTCGGGGTGCAGGGACGACGTCCACCCGTACT
GGTCAGTAGCCCAACGCGGAAGCCTGTCTGGGGCTTTGGCCCCTAGACATCGGTCCGGGGCGGGGCGCGTCATCGCTACGTTAGTGAGGGGGAGTCTGC
ACCGATAGGGTGTGGTGGTGTTGCTTAGATCGTTGACGGGCCGTGAGGTGGCGCTGCTGTTTTCCGGCTTACGGCTTACGACCTACCGCCCGCCGCGCCGG
CCCGAAGGCGGCGCCCCCGCACATCGCCCTTGGGTGGTAAAACCCGCCCTGCCGCCGCTACCCCCCCCCCAGGGGTGGCCGGAACGGCGAGGGCAGGA
CACGGTCGACGCACGCAGCGCCGTGACGCCCGGCCCGCGCTAGGGTTGGCACCCGTGGGACCTCGACAAACTTTCCGTACGATTGGGGGGCGCATACAG
CGGACGGGGAACCCGCCCGACGCACGCGCGGTCCCGAGGGGTGGGGGACCTGCAGCTGCGTCGCGCGGTCCGCGAGGGCGTCTTGCTAGACCATGGGC
GTTGCAATAGTGGGAAGGTGCCTCCCAGGCTGCGGGGCGCCGGACGCCCGACACCAAAGCTGTGCGAGGACAGAGAGGCTAGGCACGGATGGGTACAA
GCGAGCTGACAGCCTCCATCCTTGAGTGAGCACGCCGCGGCGAAATGCCGGCCGAAATGGGAGGGGCGCGACACCGGATCGTGATCGAGCGCGTGCGC
TTCGCCCACACGCCCTGACCCGCTGAGTGTCTCGGGTTTGAACCCCGTGCCCGGGGGCGCAGTGGGATAGGCACGACGGGCAGAGTTAGGGGCCTGGGC
ATAACGGCCCTGTCCGCGGCCGAGCGGGCCCGGGTCTTCTCGACCAGGGTGCCGGCCCCGGCGGGCCCCCCGCCCACGGACGGGGACCTGGAGTTGAGC
CAGGCCACAGGCGTGACCGGAATTGGAGCGCCAGGGGACAGCGGCCGCCCGCGCCAGGCCACGCCGATGGGGGCGTTCTGCCCAGGCGTCGCGCAATG
GGCGGACCGGTCTACGCGACACCGCCCGCCCGGCCTGGAACTCCCCCCGAGCACATTCGATATGGTCCGCATCAAGGCGGGAGGCATCGCCGGGGGCC
GCGCGGACCACCACCTTCGCGGGGGACGGTGCGGGTCGCGACGAGGCGGCGGCATGCGCGCGGGTGCCGTGAGCCCTAGAGTCAGCGGTTGCTGGTAT
GGGCGGACGGGCGCCCCCGACCTGCCGTGGACGCAGGCTCGCGGACTAGTCGACCCGCCATGGGTGCTGTGCGTGACGTTGGCCTTGGTCCAGGGCGCG

ACGCGCAATACCGCGCGGGCTGAGTATCGCGTCCACGGACAGGCGGCTGAGCCGCCCGGCGTAGCTGGTACACGGTCGGGCCGAAGTGGCAAAGAGCC
GGCTTGCGCCCCCAGCGCCGTTGCAGCAGGTCTGGACAGGCAGTACGGAGCGAACGCGGCGAGACACCCGGCTCCGGGCGTAGTCGGATCCGCCCGCC
AGCGGGCGCGCAGCGGTGTCGCGGGCTTGATGCGCCGGTCAGGGAGGTCGTCGCGAATGAGCCGGGTGGAGGGTGCAAGAAGCCAGACGATGGGAGC
ACCGAGTTGTCCGCGTGCGATTGCCGTCCCGGCGAAAGCGCCAGGCCGCGGCGCGTGTGGCAACGGCAAGTACCCGGAAACACGGCACGGGTCTCGTG
GCGCCGCGAGGCGGCGGGTGGCGCGCTCCGGGGGGCTGCGCAGCCCCTCAGCGCCGCTGCTAGTGCCAGACGCTTGCCCTTCTTGAGCGCGGCGGCGGG
AACTCGGTCGCGTGCGTTCAGGGCCCTCATCCGCAGACGTGAGGGGTGCGGGCCAGGGGGCGCAGAGGCGCCTTCCGAAACCCGTAAGGGATGTTCTGG
CGTTGGGGGGCCGCCGTCTTAGGGGGGTAGCGCCGCCTCAACTCCAGTATCGCCGCAAGGCCGGTGCCAGGTTCGATCCGGGGGCGGCGGCGTACCGCG
TCCACGCGTCTCCCAGAGGAGGGTCAAGGTGCATACGGGAGGGATCATACGGCAATCGGCGGCCGGGTTCCGCCATCTAACGTGGCGATCGCAGCGTAC
CAGAGCGAGGCCTATGGGCCATATCCCTGGCGGGAACGGGCTGCTTGCGCCCCGTCCTCGCCGACCCTCCGTACCGGTACGGGGCGAGGATCCCGACAC
ACAGCACGGGGGCGGCGCGCAGCACCCACCCCGTCCTCGGCCCAACAGACAAAGACCCCGCGGCGGGCTGGGGCACCCCCTTTCCGGCTACGTCCACC
GATTCTGTACAGGACAGCCGTTGCGATGGGCACCGGCGATCGGAGAGAAGGACGCCACAGATCAGCAGACGACTCGGGGAAACCACGGGACCGCGCG
TCGCGTCAACTGCCGCGGGAGTGAGAAGGGAGCGACAGGAGCGCGGAGGTAAGGCCAATGGGGCCAGTTGAAGCCGCCCACAGAGGCTGTGCGGGGC
CCCGCCGGGTTTAGGAGCTGTTCTTGGAGCCTGTCGTTAGGGGCGGGGGGGGGCTTACACGGCGCTGGGGGACCCACGAGGCGCAATCCGCCGCGGACT
GCTGGAGGGCTACCTGGGGCAGCACCCCAGGGGCGGCCATGGCGCTTGTTGACGGGGGCGCTAGGACCTTGTCCTAGGGCGCGCCGCTTTCGCCCACAG
GGCTTCGGTGGGCAGTCTATCTGTGCGGTTTCAGTCTCGTACGCACGTGATGCGGTGTAGCCTGAGCCCGGCAGTTGTCTAGAGTAACTAGCGCGCGGTTA
TGGCTTCGTCTGGAAACGGGCTGCAATGTTCCGGGTGTTCATCACACCACGTTCCGCTTTGCAGCTCACCCCCCGACCACACCCGGATTCGGACCGGTGGT
CCTTCCTCTGAGGTACTCGCCGCTCATACAACCGCGGTTCCCGCTGTGGTCGGCACGGCCCGGGTTGCAGGGGCGTGCCTTCCCGGGTAGTGTGCCTGCGC
GGCGTGCGGCTTCCGCAGTCCACGGACCGTTCCGGGGGTGAACGATGGAGGTGAGGGGACGAGTCCCTCCGAGATTAATGCCCCGCGGCAAGCCCGATC
AGCGCTGCGGTCCTGGGCTGTCAGCGCGCGCGGAGGAGCGCCCGATCGGGCGCGGGGGAATGAGAGTGTGGAGGATCATAATAGAGAGATCGCCCCGG
AGACTGCGAGGGGCATGGACGCGGAGGGACGGGGCGGGGGGTCGGCACGCCCGTGCGGCCTGTCGTCATATCCCACGCTGTCACTCGGCTGCGTCCGGC
ACTCCGGGCGGCCGGCGAGCAAAGCCGCCCTGCGCGGCCCGATCGAGGGTGCGGCGGCGCGACGTCGTGGCGTGGGAAATGCGGCGAGGGGCGGGGT
GGGGGACGTCATTGAACCTGCAGCGCGCACGGTGAGTACCGCGGCCCGTGGCGGGGCTGTACTGCGGGACCCCGGAGCGGTAGCGGGGGTTAGACTGG
GTGAGGGTAACCTGCTGCCCCGCGCACCACGTTCTTGGCCAGAGGCCCTGTGGGTGTTGGAGCAGGGTCGAGTATCCCTCCGTGCTTGCCTGTGTGGAGT
GGTTCGCCATGCAGTTGGACTCTTGGCGTCAGCTGGGGCCAGGCGGCGCCTCCGAGGTCGTAAAGGTCAACAGCGCTGTGAGATGGGCGCAGGAAGAGC
GCCAAAGACCTCCTGCGCCCCGATGTCCGCCTCGGGCGGACGTTCGGCCGCCTCGGGACTAGTTAAAGTGCGGCTTGGCTGGGGGCGCGGGACTCGGAA
GTCCGGGTGGAAGATGCGGGGAAGCTGCCCGTCGCCGGTACTGGGGGACTGTAGCTGTGATCCGGGACCACGAGGCATGCACCCGTCGTGGTCCAGCCG

GCCTGGCGCGGGGTCACAGAGCGAAACGGCGGACGCTAGGAACATTCGCTACCACCCCCCGGCGGCACCTTGCAGGGTCTACCCCAGTAAGGACCGGC
CCCAGCAGTGAGTGCGCCCGGGGGCTGGCAGCGGTAGATTGGGTCGCACGAGCTCACGTGGGCCGTTGACCGGAACCGGCAACGCCTGGGACTGGAGC
GCAAACTGGGGTACACTCTTCACGCCCGCTGCTCGTCATCGACCCCGGCGGGCGCCCGGGACCGGACCGGGGGCGACCCACCGGGGGACGGCGCGGCC
CCTGGATGGCCCTGCCCTCGGTCACGAAGCGGCCGAATGGTTTGGGGGGCCTCGGGACGCGGCCGTGGAGGTGCGGGGGGCCTGAAGTCCCTGCCTGGC
CCTCCTCCTAACGGCTTCACGCCCCTCAGGGCAGCTAGGGATTAACGTGGCCGGGTCACACGACCCGCCCAGCGCGGTACGCTGGGCCGCAGTGATTGC
GCGCGCCTGGCGGGTCCTCACCCCCCCACACACATCCGGGTCGGGGCGGAACTGGGTCCCGCCGTTCCCTCAGATCTGCCCCCTGACTTGAACATGCCCA
AAAACCGAAACCGGGCCGGCGCCCAGACCCCCGTGCTAAGGTTCCCACCGCCGACCCTAGCCCCCCCGGAGGTCCCCTGTACTCCGCACGCGACGTCGT
ATCAGGTCCCAGAGCCCGACAATGCATGCGCGTCCCGCTCTGCGCCATGCCCATTGCCCGACCAGCCCCGCCCAGCGCAGAGGCCCCGACTGCTGGCGC
CCTCTGTGGCGCTCGGCCGCTCGAGAGCACCTGCCGCCCGACCGCAGTCTGGCTACCTGCTCCACGGGGCGGCGCTCGTGGTCTCCCGTACTCGGAACGC
CACGCCCCGGGCTCGCGCGTCTCGCGGTCCGACGCATCGTGGTCTTACCTCCGACCCTTTCCTGGGGTCGAGCGCGGGGGGGCAGAGCGCGCTCATCCAG
CCCCGCGCGGCAGCACCCGCCCGCCCAGGGTGGTACCGCTCTCGAGAGTCCGGACGGCCGAACGCCAGGGCAACGGGCCGTGTGAAGACTCACCGTTT
CGGCCGACCGCCATCCTAGCTGGGGCGAGCCCCCGCGCGCGGCCGGATGCGTATGACGTGCGCGCGCTCGGTGCGATGGCGCACGTCGACTGGAATAAC
CCCTTGCCAACCCCAGCGCCGAGAGTCCCTCTTCGGGTCTCCCGTCTGCCTCCGATGGGTGGCCCCTGCCGGTACTTGTTAGGTCCTGGCTCGCCGGGCGC
CTCGTCACCTCGGCAGCCTGGATCGCTTGTCGCCGCCGCAAACGTGCGCGCGTTTCGCCACGGGCCGCCCCGCGAAAAGCGGCGGGAACGTGGACCACG
CGCGCGCCCGCCATCGGCTCGCCGGGATCCCCACCGCGGCAGCGCCCCGCCACAGCTCAGGACGCGGCCGATCGACAAGCACATCTCCCGACTACCGG
GCCCCGCGGCGTGAACGTGGTCATGGAAAACCCGAGCGTGTGCGTTCCTGTCACGGGATCCCGCCGTGGGCAGTTCGCGGCAGGGGCCTACCCGTCGAG
GGGCTACACCGGACCGCCCCTCCAGCTGCATCGTGCAGCCCTAGTGCTGCAGCGAACTTGCCCCGGCCATCCCGTCCGCCCGCAAGCGGCTGGGGACAG
GTTCTCCGCCCATGCGCAGCCAGTGGCCTCATTCATGAGGGCGTGGCGGGCCGGGCCGCGTTGGGCCATCGCCGGTTGGGGGCCCCCACGCCGTCTACCT
GAGTGGACGCGCCGGGCGAGGCTCCCACCACCCCAGTCCTGGGGTCCCAGACGGCCTCCTGGGCGGAGTGTTCTACCCCGCGGCCACCCCCAGCCCGTC
TGCGGCCGAGGCCCCACTGGAGACTGCATTCCCCGCGGCCGGGGAACCGGCCACACCGGCAACGGCGCTCCTGACCCCACCCGCCTGCCACACTCCCCG
CGGCGCGGAGCCCCACCCATACTAAGGCCCCTACGCGCGGCCGACGACATGTTGATGGCTTCGCTGGGGGGGCGGTGACACGTCCACGAGGCGCCTCGC
CGCCGTCGGCCCTGCCCCAGCACGTTCCCCCGTCCCCCGGGGGTGCGGCGTCCTGCTCGCCCCCCGTGTAGGAACGCCGACCGGGCTGCGTCGTCGATGA
GGCAGTGGCCCGCTACGCGGCTCCTCTTCGCACGTGCTTTTGGCTGAAGAACGGGCACCCCCACCATTACGCAGTTGGGACGGCGCGCGGCTGCGAGGG
CCCAAATCTCTCCCATGCGAGTCGTGGTCGCGCCCGTGGACCTCTGAGGGGCAAGGCCGCTCGTCTGCTGAGCGGGGATGTTGCGCCCAGCAAATCGAG
GAAGGTTTGCTCGTAACTTACGGGTACCGAGACTCCTCGCTTGGCTCACCCATTGCGGCAGCCGCGGCCGCCGCCGGGACCAGTAACGGCGTGTCGACC
GCCCCTCGACAACGCCTGGTAGGACGCTCCCCTCCCGGCCCGGACACTAACCCCCGCAACAGCGAATTCCAGGGGCGTCTTCCGCCCAACAAAGACCCA

TCGTGCACGAATTGGGCTGGCGGGCAAGCATGTGCATCACGGACTAGGGTACCGCCTGCCTCCCCGCTGCCTCCGGGGCCCTGGCGCTCCGTCCGATTTT

ATCCCGCTCTGCTGCGATACTCAACCAACCGTTTCGCTTTATTCAGGCCGCCTCTGGGGCCCGGCCGAACACTTTCTCCCGACAAGTGGCGTGACGGACCT

GCCTTCGCGCCGCCCCGAAACAGGCGGGCCGGCCTCGCACCCCTGGGGAGCGGTCCGCCGCAGGCGGGCCTCCTGAGCGCGCGGGTGGCCGGCCAGCG

CGCATACCCCCTCGTCGCTGCCGCCCCCCCCGCCCCGATCCCCGGCCCCGCTCGGCAAGACAGTGGAACCCGCCGCGCCCTTCACTCGCCCGGCTGGCCG

TATTTCGTGGGTCCTCACCTCAGTCGCGTCTGACAGATTAGACCTCGCCGCTGATGCCTAGGGTGGGACACCACCTTCCCGCCCGCCACCACCCTGTGGA

GCGTGAGCGCACTTAAGTCTCCCTACCTCCCCAACTCAGCAGCGGCCCCAGGGCATCCGCGAGGCTCCGTTGACGCACGTGCCGTCGTCCCGCGCCCACT

GTGGCACCTCGGCGGCGACCCTCCGCTGCGGGATGCCTGGAGGAGCGGTCCAGCCTGGGCGCTCTGCCGGTCACCAACGGCGGCCGGGCGGGGGCCCTC

GCGGTCGGACCGAACTTCGGAGGGGTCCCCGGCAGCTGAGTCGAGCGCTGCTAGTGACCTCCACCTCGGCCTAGTCACACTTGCCCGGCTCCCAACCGCT

AAGACATAGGGGGCGTCGCGACGAGGGCAATGCGGGGGCAACCTTGCCACCCCCAAGCGACGTCGCCTCATGTAGGTTGGAGGGGCTGTCTTGTACTCC

ACCGAGGGCGACACGTAGCGTGCGGCGGCTCGACTCGAATCTTGCGCCTCGGTACGCGCCACGCGACCGATGACCTTTGCAGGCGACTCTGCGAGAATG

GGGCCCGCGTCCTGGGGCGTGCTAAATCACGACCGCCGCCGCTGGCCTCCAGGAGGTCCCAGGGGGCAGCACACGCCATTCGCGTCGGTGGTCTAGCCT

CGCGGCACCTCGACGAGTCCCAGGACGCGTCGGGCCCGGTTCTTCCTCTTCCTGTGTGGCAGTCCGGCCGGGTGACGCTTCCGCCGCTGGCTAATTCAGA

CCGCCGTCAGTCGACTGGGCTGCCCTAACCAGCCAGGGCACTCGTGGACTTCCGGCGCCGTCCGACTCCACGCGGCGCGCACAGTCGCCTCCCTCCTTCG

TACCATTCGGCCCTCCTCACCCCAGGGGGGGCTTGCCGCCATTCCCTACGCTGCCATTCGACGTCCCGCGCTACTCACCCATAGTGCCAATTGCGGCGTCG

CGGACACCGGCCTGGCGCAGCGCCAGAGCGCCACCTGTGACGTCGCTCACCGCGGGGGGAGCCCAGCCTCGACGCCCCAGATGGCTCGCGGGGGGGA

GGTCCCGAGGCCACCCTTCCAGGTGGCACAGATGCCCCTCCCTGGAGGGGGTGGAAAGCGCGCGGCGGGCCCAACACCGTGGGCGGCAGCTGGGCATG

CGCTCGGATAAGAGCGCCAGGATGGCAGGAGGGCCGATCTGGCGGGTGGTGGCCAGTGCGGGGCGGGGGGCGAAACTGTCTGGGGGTACCTGGAGCCC

AGGGTTCAGGGTCCGTGCCATGAGGCTGTCAGGCAAGCCAAGCCGTTAACGGCGGCCAGCGAGTGGGCAGGGAAGGTGACTAGCGCGAGCTACATATT

GCCGGCCGCCGCGCGGGGCGGCCAGCCGTGAGGAGCAATCTGAGGGACGGCCCACCGCCGGAGGGCCTGCACCATGAGGTGAGGCGGCGGGTCCCCG

CCACGCCGCGAGACGGTACGACCCGTTCCGGATGTGCACCACCGATGAAGAAGGCGCGCACATGCCGTTCGCTTTCGGCGTATGGTCTTCGCAGCCTCCG

GTGGGAGGCGCAGCCCGGAGGGTGCCCGCGCCACACGGATCAATGGGTTGGAGGGGCGGACTCGTGGTTTCGACCATTGGTGCAGCTAGAAACCGTCCC

AAGGCTCCACTCGATCGCACTGCCGTCGATCTCGGTTCTGACTCCCACCTATGCCAACACGGGAGTGCGCTATGAAACGCGGTTTTATAGTCGGTAATCG

CGACCGAACGCGGAGCGCCGTCTCGAGGCTTGTAACGCTCCGCGGTCGTTGTGGAGCGGACCGGGTAGTCAGCGTGACGTATTTCTAGCCCCCAGTGTGC

CGCCCGGCGAGAAAGTCCTCGCGGGAGCCCGTCGGCTGCCGCTGCGTACGCGGGGGAAAGCGGCCGAGGTACGTCTTCCTGGTGGCTTGCTGCCGCCAA

CCACCGATCGGCCCGGCTCGATGACAGCCGGCGAGAATGGGTTGGGGCCTACTTGGCCGGGTTGGCTGATCGTACGCAAGCAGCGTGCTGCGGGCCCAC

GCAAAGGGGAATGGCGGGGGAGCGCCGGCTGCCAATTCCCCCCGGCTGCACAGGCATGATCCGGTGTCAAGAACGGTCTCCGGCCCACGCGGCCGCGC

CTTGGCCCGAGTACGGCTGACTCAGAAGCCGTTGGGTGCGAGGGCGTGGGGTGGTGGCGCATAGGGAGCCAGACCCGACGGAGCCAGGATGGTTCGAG
CAGTGTCACGGCCCGGGGTCATGTGGGGCCCGCCCGCACGGATGACCCGCGTCTCGATAATCGGTTGCAGGCGTGCCGTTCCCACGGGGCGCGCCGGTT
ATCGAACGGGTGCGCGCGTAGACTCGCTTCCGGGCCCCGGACCTGGGCCAGGCAGAAGCGGGGATTACCCGTCTTGATCGCAGACAGCCCGGTGCCCCC
GGGGGCCGACGTACAGCTGGCAAGCACCGGTCCGGCGATTACCCGACCTGGGTTGGCAGGTACACGTTACAGGGGTGGCGTGCCCCCCGGCGCTTGGCG
CCGGCTTGGAGGGACGGGGGGGGATCTAGCTCAGATGGCCAGCGAGCGTAGCTTCGACGGTGGCAAGAGCGAGCGGCGGCGTGCTTGGGGAGGCCGCG
GGCGGGTGCGACGTCCAGTTCGTCACACGCATGAGGGGTTTCCGGGGAGGAATCACCGCCTCGCGCAGGCGTGTCCGACACGACCCGTGTAGCGGGTGT
GAGACATTTAGGCGGAGGCCAGAACCCCTTGCCGGAGGGCTTGTAGTATATTGTCAATGGGGCCGACACCACGTGCGGATCGGGACTGAGAGCGCGTAG
AGCCTTGGCCCTTGCTATATTTGTGGAGCGGACACTGTGCTTCCCACACGTCACGCTGGCGTAAGCCGATGCCCGTCACCGCCCGCGCCCCAGGCCGAGT
GCGGGGGGCCGCAAGGTCCTGAAGCCGATCTACGATGGGCTGGGTGGGCGACGTCGTGGGCTAAGCTCGGCGGCCCCACCGTGAGGCGGACGTGGGGG
CCTGTACTAGAGATGGAGCGACGGTCGTCGATACAGTTCTTGCCAGGAGACCGAGCATTTTGCTAGGCACGGGCTGCATGGGCAATTCATTAACGGCGG
CGGGCCCACCGCGGCCAGCGGTGGCGGGGCGTAGTACCGGTCGGCAGGGATACGACCCCGGGCCGAAATTCGGGGCCGGCGGCCAGGGGGCGGCGCG
CCTGCGCGCCGTGACGCTGACGCCCTCCCGGGGTGCCGCGGGCTGGCCTGAGGCCCAAGGAGGCGCCTGGGGGTCGAGCGCGGGCCCGGGCGCACGCT
GATCCCGAGCCGACGAGCTTACGGATTGACCAAGAGCACCGCGAGCGTCCGAGCGCTGCGGGCGTGGTAACAACTACATCCCGGGTCGAGTTTGCGGCT
GGGACGTGGGATTGGCCGTGCAGACGCGCTGGGGGGGGAGCGGGGCTGGCCCCTCGCTCCGTATTCCCCACTCCACATGGTCCCCGGGCGTACGGGCGT
CCCCGACGGTTCGGGACGCGGGAATCGCGCAGGTGCCCCTCGGTGCGTGGCGGGCTGCGTGTAAGGCGCTTTCGCTGCATGCGGCCGTCTCGCGGACAT
GGCGTACGGGGGCGACCAGGGCGAATCCAAGGTGCGCCCAACGGGCCGAGGCGCTACAGATGGGGAGCCCCGGGCGGCCGTACGCAGGAGTCCAGTG
GCGTATGGAGGGAGGCCTGGGGCTCCGCAACGGCCGAGGCTATGCCCAGACCCCATGCCATTGTAGGCCGAACGCGATAGGTCACGCCGGTGATCCTGC
CGGGGTCGCGGGCCGGATCCACAAGGCCAGACCCATAGCGTAGTCTATAGGCGTCCGGGAAGGGTCGTGGGGGGGCTAA

>2017.TE.25009.1.7

CACGCCCCCCTCCCGGCCAGCCCGCCCCCCGCCCCAGCGTCGTACCCGGCGTCGCGACTCCCCGCCGTCGAGAGGCCTTTGCCGGGGCCCCGGCTAGCC
CGGGGCGAGCGGACCGCCGCGCGCGGGGGGTGTCACCCGGGGCACACCGCGCCAGCCTCCCGGGCATGCCACGCCGGGTTCCGCCCCCGCTGCTCCAA
TTTGGGGGGCCCCTAAGATCCCGTTCCCCAGGCCAGCCGCGCCCGCGGGCCAACATTGCCACACCGTGTCGCCCCCCCGCCTGCCGCACTGACCGTCTTG
CGGGCCCTGCTCCCTGGCCCGAGCACACCTCCGGGGACCCCCCTCGCTCGGCCGGGGGAGCCTTAAGCGCGGCACCGAGTAGGGGGTCGCGTGTTGCCG
CCGCGCTGACGCCCCGGCTGGGACCCCTTTCAGGTCGAGACGCCGGGGTGGCCATGCGGGTCCAGTCGCACGATGGCGCGGCGATGTGGCGCCGACTGC

CCCGCCTGTCTTCTCCGTGCCAGCCCCTCGCCACCATGCCGGTCCGGCCCGCACGGGGGTAGGAGGTGCTGCCGCGGCGGGAGGCGTAGGACGCCATAT

CCGAACCGGCGGGTCCCCCCGGCCACCGAGTCGACAGGACCATAGGCACCGCGTCCGGGCGGGCGCCCTTAACGCCGCCCCTTACTGGAGAAGCAGAG

CTCTGCCGAGGCACCCTGCCTAGCTGGCTGCGTCCCGCACCCCGCGATCCCCCTGGTCCACTTACCCAGCGGGATGCGGGGATGTTCGCAGCTATCACAC

GGTCGTGGCCTGCCTCCCTGGGCCGGCGAGAGCACCGCCACCTTCGTGTTCCTTGCCGCCGGGCGACGTGTCCGGGAAAGGCTTCGCCGCGCGCCCGCGC

GGAGCGCCGGGGAGTCCCGTGAGCGGCCGCAGGAACGTCACGGACTCCGGCTTGGTGCGGTCCCCCTCAGTGGTGCCCGACCAGAGACCGCGGAAAGA

CTCCAGGAAACGCCTCGGGGGGAAAGCAGGGCCGCGCACTTCCGCTCCACGCTAGCTGGGGGGACCACGACGGCAATACTGAACACCTGGCTCTTTGGC

AGCCCGGCCTATGAGGGGCCCTCTCGTCCCGGCCAAAGGCTATTTGCCTTCCTGTCCGGGCACCCTCGACCGCACCCGGGCGGGCGCAGGCTTCATTGCG

GCGCGACTCGCCAAAAGTCCCCGCGATCTCAGCGAGTACCCTGAGCGCTGGCCCTAGTCCGCCTGCCCCCTTAATGTCGCTCAGGTCTGCAATCTCTTAC

GACCGGCTCAGGTAGTGGCCGCACGGTGCGTAGGTCCGCTGCGGGCCGGGCCACTCGCGCCGATCGTGCCGTGCGGCGGCCCTGACACCGTATGCGTGC

GCGCTCCGCAGGCTCGCGAAAATCCTTCGCAGGCACTTGGCCCCGTTGCTAGACGTGGTGTCTCCTAACTGTGGCTCACGCACGGCGTTTAGGCCACGGT

AGCCCGCTGTTCGGTCCTTACGACTCGGCACACGCCCCCGTGCACGCCCTCGGGTTCCGCAGGCCAACGCCTGGCCCTAGGGTTGCGTGCCGGCCTGCCC

GCGATGCCACTTGCCCCACGTCCCCCACCTGGCCGGCGCAGACGGGTTCAGTGGGTGTCGAACGCCATTCAGTCCCCTGGGACTCCCCCGACCCCTCCCA

TGGCACCCGCCGCCTGGCCTTGGAGCTCCTGCGCGTTGGACCTTACTGCGGTCGTCAGCTTCTCGGCGCGCCCCGGACTAGTCCTGGGCGGCCAGCTCGC

GGGACCCTAGCGCGCACGCGTGCCCGATTGCCGTATCCCCTCGACGGCGCCCGGGCATAGGGTTCGATCCCGCTGCTATTGCGGGTGCGTCCAGACCTGC

CGCCGTTTGGCGATCCGAGTGTGCGAAGGGAGCCAGCCAGCGGGCCGCCTGGCCGCACTCCAGGATCCTGGCGCCAAGGGAGAGCCCTGCCAGCGCTA

CGCGAATACTCGACCTGTCCGCGCCCCGCCTCGGGCTTGCGAGATGGCCACTAGACGCTATCGGGTCGTCACCCCGTCGGCCCCACAGGGGCCCGGCTTC

GCGTGGCTTTCTTGTCGAAGGCCCCGAAATCCGGCAGGCCCCGACACCTTCACCGTGAGCGAGGACGCGCGGTGCGGGATGTCCAGTTCATGCACGCCC

CTCCACGGCCGCCTTCGCGCGGACAGCGTCGGGCGTGCCCAGGACGAGAACAGACCCTGGGCCAGCCTCAGGGCCCGGCCTAGGCGACCCTGGTGACC

AGTTTCCACTCGGCACCCTCGGGCTTCGTGGCAGTGCAGTCATGCCCCCGGGTGCTCCCGCGCGACCGCGCACCTCCCACCCAAGGTTCTTGGTCAACGC

GCCGGATCTGACGTCGCGGTCCGGCCCGCGCCGTAGGCAAAAATGGGTTCGGCTCCCGCCACTCCGCCAGTTCGAGGGCGGTAAAGATCTCAGCCGCCG

TGTTTCGGGCTATCTCCCCGCGATCCCGGGCCACAATCGCAGGTGGCTGGGGCGTCGGCCAGACCCGTCTCGTCGGCCTTTCTGCGCACATTACCTCCGAG

CAAGGCAACCGCTCCGCCCCATTACAGGGGGTTGTCTGGTACCTCCGTCACGTACCCCCGCATCGCTGGCGGGCTGACCCAAGCCCCCTCCCATCCCGAC

CCTCACGCGCTCACGCCCCTTGCCGGGCGGGGTCGTGGCTGCCCAGGGTCCAGGGCCCGCCCCGGGGGCCGGTTGGTTCCAGGAACTGAGGGGGGGTCG

CCCAGCTGACCGCTCCTCTCGCGGGGTCTATTCGGCGTGGCCATCCGCAGGTGCGACAATGCCGGCTCCCATTGCCGGGCCGACCCTGGTCCCTGGCGCT

TCGGACTCCCGGGGAGCGCCTCCCTTCATCGGCGGCCCCTGCCCCCGTCATGACCCCCCTTACGCGCCGGTGGTAGCCGCGCGTGGTGGCGTGCCCCTCT

CCATTATTACGCTCCACAGGAGTGGCCACCGGTATGGGCGCGGTGCAGTCGTACATGTGTGCGGCCCTGTTTTGCGAACCCTTCCCGCGGTGACCTCCGCC

CCAGCGGCCTTCTTCGACCGTTAACGCCGGCCAGCACCCGAAGGGGGTCACACAAAGCCCTGCAAGGATGCGACGCTGCGTAGCCGCAGGTTAGCCGCT
TCCTTCCCAAAACAGTGTCAGAGCTGTCCGGCGGAACGTTAACGCCGCCGGGAGGATCTGCGGCTACCCTCAGGCCCGTCCCGTGGGAGTGGCCGGCGT
CGATGGGGGTACGCACCCCACTAGACGCGGATTTTGGCCAACGGGCACGGCCGCTTCCGAGTTCCCCTGCGGTCTGAGTGTTGGTACGTTCAGCCGGCCG
TTCCGTGGGGCAGAGCGCCGCACGCATGTCGCAGGAGCGCGCCCTCCGCGTCTTGCGGGCGGGGTCCTAACGAAGCCCCCTGGGCTTGGAGGTAGGGGG
CGCGGGGGAGCGATGGGAGCGCAAGCGTGCCATCGGGTCCGGCCCATAAGTGTGTATGGCGCACCGCGCTGCCACGATGTCCGGTGCGCGGGCGCCGCT
GGCTAGCTCACTCTCCCCCGACTCCGCCGCCGCGCCCCGAGACGTATGGAACAGCTGGCGCGCGTCCCCCGCCGCGGGCGGCAACGAACTAGATCCACC
GCCCTCACGAGGAAGCGCGCCGCCACCACGTCTCCCCGGACGACGCGGTCCCACCATCCGCTGTGCCGGCGCGCGAGCAGCCTGGCCCTCGGGGCGGTC
GCGCCAGCGCAGACACCCTGTCACGCGCACGGGAGCCCTGGCCACCGCGTCGACGCCCGGGCTAGCCCCGACTATCAGTACTGACCAGAGGGGCGCCG
GTTGTCCCGACTGGTTCCTCCCGAAACGCTGGCGGTGGGCCCGCACGATTGGTGCAGGCCCCCAAGAGGATACGGCTATTCGCGGCTGCCCGAGGCGAG
TCGGGCCAGACCGGTCGCGCGTGTGTGTGTTAGCCCTCCGTCGCGGCCAAGCCGGCGACACCGGACTGGGCGACAGTAGCGTTCTTCTGTCACTAGTCAC
TCTAACGGACAAATGGAAAAATGGCACCTATCGCTACCGACGGGGTTTTCCTCCAGGATAAGCCTCCGCGCCCCCCACTGGACGCCAGCCATCCTTCCCC
GAACGGCTCGCCTGTGCCGGCTCCAACCCCGCGGGACCTCCCTTCGCGGCCCCGGCGGGGATCGGTGGATCGTCGCCGCCGGCGCCTACCGATCGCGCC
GCCACGGTCGTGCACCAACCAAGCACTGCTACACGCCTGTGCGGCCGAGGCCGCCCCATCCAGCAACGGGGCACCGTTGACCCGTCCCCCATGATGCCG
GACATGGTAGCCCCCACACATGCATCTTCGGGTAACCCGCGCTAGGTCCCCCCCCGCGCCGCCGCCGGCTTTCCCCCCGCTGGTGCTAAGCGGCAGATGC
ACCACTGACGGGACACACTCGCCCCCGATCCAGTGCGCTGCCGCCTGCGGCCCCCCACCGGACTGGTGCCTGGCGCTACACGGAGCCGGCCGCGCACTG
TGACGTATGGATGCCCGAGGGGACATCCGCCCCGGACGCGTGCGCAAAAGACCACTCGCTCGACGCCACGACGTGTGCGGCCGGGGTGATGAACGTCG
GCCGGTGTGCCAGTGAGGCTTGCACCTACACTCCCTGGTCCACCGGGCCGTTCGAGCGGCACCGGGCGAAGAACCCGCAGCCTCCCGGCTTACGCACCG
GCCTCGCGTCAACAGGCAACCACGACCGCAGGGCCGGCAGTGTGTGTCGCTGTGGAAAGGTGCGCCGCAGGCGACCCCCACGTCAGCCCACTACAAAG
CGTGGCAGCATGCTGCGCAGCGACCGCACGCCCCGACACAGCCCAATGCACAGCATCGGGGGGGCGGAATCGCCCCACGCCCGTAACATCCCGCGGGT
CGACCGCCTTACACGTCGACTGCCACACCCTGGTGCCGCAGTGGTATCCCGAGACCGCATGACCCGCCGCGAGCCCGCTGACCACCTGTCCTTGATCGCA
GTCGCCACGGGGCCGGAAGGTGTACCGCATACCGCACGTGCTGCGCGTGTGCAGCACGGGGGCACCCTGCATGTATGGATCCCTCTAAGCCGACCTGCC
ACCCCAGGCCAGAGGCTAAAGTCTGCCGCCTAGGCCCCCCCCCGTGGTCGCCAGCCCCGGTGGCGGCCCACCCTCGGGGCGACTGACCGGCCACCATCC
AGGGTAGAGAGGTTTGGCCCTAAATACGGGCGTGCCGAGGAGGTGTTTGGGGCGAGCGGCAGCAGGTTCGGCGGATCCTTGAGGGTGGGGCCTGGGGTG
GGACGGGGGCGGCCGAAACGCGAGCATAGGCCCCTGCCTGGCCCCTTTGCAGTCTGTACTTTTATTGACATGATACTAATACCGTGTTCCGCCCTGAGAT
CGCACGCGGACCTACCGTGCCTCTCTCTGCATAGTGCTTCAAGAGCCCAGCGCGACTGGGGACCGGCTCCCGCAGCCGGCTCGGGGCGTAACGCTCGGT
GGAAGGGTCACCCGGATAGCTCCCGGTTAGTTTCGTACAGGGTGTCGGGGGCGCCCCCAGCGAGTCTCCTGGAGGTGCAGGGACGGCGGTGGTCCTCGA

GGGGGCGGACGTTGGCCGCATAACGGGTAGTCTCCAGGGTCACCTATGGTCTGGCTCGCCGTCAGGGTCTGTGAGCGTCGTGCTCACGCCTCCGCAACAG
CCGGCGACCCCGGCTCGCAACGGCCAGCCGTGGGCGGGGGGGCGACCGGACGCAGTTCCGAGTCTAGGCATGGTGCCACCGCCCTCTCGTTGGGTTAGT
CTCGAATGGCTGTTTCGGGGGTCAGCGCGGCGGGGCCGCCGCTGGGAGCCCGCATAACCGTGGATACACTTGCTGGGTGCGCGCTCGAGGGCGGCCCCC
GAGGGCTGGAAAACCATTTCGGGATCGCCCCCGCGCTCCCGTCCGCACTGCGGGGTCCAGCCCGACGGGGGCGGGTTTCGTTGGGGCAGTACTCACGCC
CACGGCGAGGCCCATCCAGAGAGTGAGGGTGTCGCTGGGGGGGCCTACCGGGCGGAACGCGCCCCGACTTAGGGCGCCGCAATGCACGACCAGTCACC
GTCTGGGCGTCGCCGCGCCGTTGAGAACCCCAACACAGAAAGAGCTTGCCGCCTCTGGGGGAGCCAGGCCGAGAAGCGTGGGCGAGCGGCCACGCTGC
GGGCCCTCCGCGGACCGTGCGGCGCTCTCGGGTGACAAACCTAGTCCCCGTCCCAGGCCATGGGCGAGCAGCTGCGCCGGGACTGTGCGACGCCTATCC
GGCACATTCGGTATTATTGGCCTGGGACGACAGCCGGCGACAGGGCGGACGAGGCGGCGTGGACTCGGGGTGCAGGGACGACGTCCACCCGTACTGGT
CAGTAGCCCAACGCGGAAGCCTGTCTGGGGCTTTGGCCCCTAGACATCGGTCCGGGGCGGGGCGCGTCATCGCTACGTTAGTGAGGGGGAGTCTGCACC
GATAGGGTGTGGTGGTGTTGCTTAGATCGTTGACGGGCCGTGAGGTGGCGCTGCTGTTTTCCGGCTTACGGCTTACGACCTACCGCCCGCCGCGCCGGCCC
GAAGGCGGCGCCCCCGCACATCGCCCTTGGGTGGTAAAACCCGCCCTGCCGCCGCTACCCCCCCCCAGGGGTGGCCGGAACGGCGAGGGCAGGACAC
GGTCGACGCACGCAGCGCCGTGACGCCCGGCCCGCGCTAGGGTTGGCACCCGTGGGACCTCGACAAACTTTCCGTACGATTGGGGGGCGCATACAGCGG
ACGGGGAACCCGCCCGACGCACGCGCGGTCCCGAGGGGTGGGGGACCTGCAGCTGCGTCGCGCGGTCCGCGAGGGCGTCTTGCTAGACCATGGGCGTT
GCAATAGTGGGAAGGTGCCTCCCAGGCTGCGGGGCGCCGGACGCCCGACACCAAAGCTGTGCGAGGACAGAGAGGCTAGGCACGGATGGGTACAAGC
GAGCTGACAGCCTCCATCCTTGAGTGAGCACGCCGCGGCGAAATGCCGGCCGAAATGGGAGGGGCGCGACACCGGATCGTGATCGAGCGCGTGCGCTT
CGCCCACACGCCCTGACCCGCTGAGTGTCTCGGGTTTGAACCCCGTGCCCGGGGGCGCAGTGGGATAGGCACGACGGGCAGAGTTAGGGGCCTGGGCAT
AACGGCCCTGTCCGCGGCCGAGCGGGCCCGGGTCTTCTCGACCAGGGTGCCGGCCCCGGCGGGCCCCCCGCCCACGGACGGGGACCTGGAGTTGAGCC
AGGCCACAGGCGTGACCGGAATTGGAGCGCCAGGGGACAGCGGCCGCCCGCGCCAGGCCACGCCGATGGGGGCGTTCTGCCCAAGCGTCGCGCAATGG
GCGGACCGGTCTACGCGACACCGCCCGCCCGGCCTGGAACTCCCCCCGAGCACATTCGATATGGTCCGCATCAAGGCGGGAGGCATCGCCGGGGGCCG
CGCGGACCACCACCTTCGCGGGGGACGGTGCGGGTCGCGACGAGGCGGCGGCATGCGCGCGGGTGCCGTGAGCCCTAGAGTCAGCGGTTGCTGGTATG
GGCGGACGGGCGCCCCCGACCTGCCGTGGACGCAGGCTCGCGGACTAGTCGACCCGCCATGGGTGCTGTGCGTGACGTTGGCCTTGGTCCAGGGCGCGA
CGCGCAATACCGCGCGGGCTGAGTATCGCGTCCACGGACAGGCGGCTGAGCCGCCCGGCGTAGCTGGTACACGGTCGGGCCGAAGTGGCAAAGAGCCG
GCTTGCGCCCCCAGCGCCGTTGCAGCAGGTCTGGACAGGCAGTACGGAGCGAACGCGGCGAGACACCCGGCTCCGGGCGTAGTCGGATCCGCCCGCCA
GCGGGCGCGCAGCGGTGTCGCGGGCTTGATGCGCCGGTCAGGGAGGTCGTCGCGAATGAGCCGGGTGGAGGGTGCAAGAAGCCAGACGATGGGAGCAC
CGAGTTGTCCGCGTGCGATTGCCGTCCCGGCGAAAGCGCCAGGCCGCGGCGCGTGTGGCAACGGCAAGTACCCGGAAACACGGCACGGGTCTCGTGGC
GCCGCGAGGCGGCGGGTGGCGCGCTCCGGGGGGCTGCGCAGCCCCTCAGCGCCGCTGCTAGTGCCAGACGCTTGCCCTTCTTGAGCGCGGCGGCGGGAA

CTCGGTCGCGTGCGTTCAGGGCCCTCATCCGCAGACGTGAGGGGTGCGGGCCAGGGGGCGCAGAGGCGCCTTCCGAAACCCGTAAGGGATGTTCTGGCG
TTGGGGGGCCGCCGTCTTAGGGGGGTAGCGCCGCCTCAACTCCAGTATCGCCGCAAGGCCGGTGCCAGGTTCGATCCGGGGGCGGCGGCGTACCGCGTC
CACGCGTCTCCCAGAGGAGGGTCAAGGTGCATACGGGAGGGATCATACGGCAATCGGCGGCCGGGTTCCGCCATCTAACGTGGCGATCGCAGCGTACC
AGAGCGAGGCCTATGGGCCATATCCCTGGCGGGAACGGGCTGCTTGCGCCCCGTCCTCGCCGACCCTCCGTACCGGTACGGGGCGAGGATCCCGACACA
CAGCACGGGGGCGGCGCGCAGCACCCACCCCGTCCTCGGCCCAACAGACAAAGACCCCGCGGCGGGCTGGGGCACCCCCTTTCCGGCTACGTCCACCG
ATTCTGTACAGGACAGCCGTTGCGATGGGCACCGGCGATCGGAGAGAAGGACGCCACAGATCAGCAGACGACTCGGGGAAACCACGGGACCGCGCGTC
GCGTCAACTGCCGCGGGAGTGAGAAGGGAGCGACAGGAGCGCGGAGGTAAGGCCAATGGGGCCAGTTGAAGCCGCCCACAGAGGCTGTGCGGGGCCC
CGCCGGGTTTAGGAGCTGTTCTTGGAGCCTGTCGTTAGGGGCGGGGGGGGGCTTACACGGCGCTGGGGGACCCACGAGGCGCAATCCGCCGCGGACTGC
TGGAGGGCTACCTGGGGCAGCACCCCAGGGGCGGCCATGGCGCTTGTTGACGGGGGCGCTAGGACCTTGTCCTAGGGCGCGCCGCTTTCGCCCACAGGG
CTTCGGTGGGCAGTCTATCTGTGCGGTTTCAGTCTCGTACGCACGTGATGCGGTGTAGCCTGAGCCCGGCAGTTGTCTAGAGTAACTAGCGCGCGGTTATG
GCTTCGTCTGGAAACGGGCTGCAATGTTCCGGGTGTTCATCACACCACGTTCCGCTTTGCAGCTCACCCCCCGACCACACCCGGATTCGGACCGGTGGTC
CTTCCTCTGAGGTACTCGCCGCTCATACAACCGCGGTTCCCGCTGTGGTCGGCACGGCCCGGGTTGCAGGGGCGTGCCTTCCCGGGTAGTGTGCCTGCGC
GGCGTGCGGCTTCCGCAGTCCACGGACCGTTCCGGGGGTGAACGATGGAGGTGAGGGGACGAGTCCCTCCGAGATTAATGCCCCGCGGCAAGCCCGATC
AGCGCTGCGGTCCTGGGCTGTCAGCGCGCGCGGAGGAGCGCCCGATCGGGCGCGGGGGAATGAGAGTGTGGAGGATCATAATAGAGAGATCGCCCCGG
AGACTGCGAGGGGCATGGACGCGGAGGGACGGGGCGGGGGGTCGGCACGCCCGTGCGGCCTGTCGTCATATCCCACGCTGTCACTCGGCTGCGTCCGGC
ACTCCGGGCGGCCGGCGAGCAAAGCCGCCCTGCGCGGCCCGATCGAGGGTGCGGCGGCGCGACGTCGTGGCGTGGGAAATGCGGCGAGGGGCGGGGT
GGGGGACGTCATTGAACCTGCAGCGCGCACGGTGAGTACCGCGGCCCGTGGCGGGGCTGTACTGCGGGACCCCGGAGCGGTAGCGGGGGTTAGACTGG
GTGAGGGTAACCTGCTGCCCCGCGCACCACGTTCTTGGCCAGAGGCCCTGTGGGTGTTGGAGCAGGGTCGAGTATCCCTCCGTGCTTGCCTGTGTGGAGT
GGTTCGCCATGCAGTTGGACTCTTGGCGTCAGCTGGGGCCAGGCGGCGCCTCCGAGGTCGTAAAGGTCAACAGCGCTGTGAGATGGGCGCAGGAAGAGC
GCCAAAGACCTCCTGCGCCCCGATGTCCGCCTCGGGCGGACGTTCGGCCGCCTCGGGACTAGTTAAAGTGCGGCTTGGCTGGGGGCGCGGGACTCGGAA
GTCCGGGTGGAAGATGCGGGGAAGCTGCCCGTCGCCGGTACTGGGGGACTGTAGCTGTGATCCGGGACCACGAGGCATGCACCCGTCGTGGTCCAGCCG
GCCTGGCGCGGGGTCACAGAGCGAAACGGCGGACGCTAGGAACATTCGCTACCACCCCCCGGCGGCACCTTGCAGGGTCTACCCCAGTAAGGACCGGC
CCCAGCAGTGAGTGCGCCCGGGGGCTGGCAGCGGTAGATTGGGTCGCACGAGCTCACGTGGGCCGTTGACCGGAACCGGCAACGCCTGGGACTGGAGC
GCAAACTGGGGTACACTCTTCACGCCCGCTGCTCGTCATCGACCCCGGCGGGCGCCCGGGACCGGACCGGGGGCGACCCACCGGGGGACGGCGCGGCC
CCTGGATGGCCCTGCCCTCGGTCACGAAGCGGCCGAATGGTTTGGGGGGCCTCGGGACGCGGCCGTGGAGGTGCGGGGGGCCTGAAGTCCCTGCCTGGC
CCTCCTCCTAACGGCTTCACGCCCCTCAGGGCAGCTAGGGATTAACGTGGCCGGGTCACACGACCCGCCCAGCGCGGTACGCTGGGCCGCAGTGATTGC

GCGCGCCTGGCGGGTCCTCACCCCCCCACACACATCCGGGTCGGGGCGGAACTGGGTCCCGCCGTTCCCTCAGATCTGCCCCCTGACTTGAACATGCCCA
AAAACCGAAACCGGGCCGGCGCCCAGACCCCCGTGCTAAGGTTCCCACCGCCGACCCTAGCCCCCCCGGAGGTCCCCTGTACTCCGCACGCGACGTCGT
ATCAGGTCCCAGAGCCCGACAATGCATGCGCGTCCCGCTCTGCGCCATGCCCATTGCCCGACCAGCCCCGCCCAGCGCAGAGGCCCCGACTGCTGGCGC
CCTCTGTGGCGCTCGGCCGCTCGAGAGCACCTGCCGCCCGACCGCAGTCTGGCTACCTGCTCCACGGGGCGGCGCTCGTGGTCTCCCGTACTCGGAACGC
CACGCCCCGGGCTCGCGCGTCTCGCGGTCCGACGCATCGTGGTCTTACCTCCGACCCTTTCCTGGGGTCGAGCGCGGGGGGGCAGAGCGCGCTCATCCAG
CCCCGCGCGGCAGCACCCGCCCGCCCAGGGTGGTACCGCTCTCGAGAGTCCGGACGGCCGAACGCCAGGGCAACGGGCCGTGTGAAGACTCACCGTTT
CGGCCGACCGCCATCCTAGCTGGGGCGAGCCCCCGCGCGCGGCCGGATGCGTATGACGTGCGCGCGCTCGGTGCGATGGCGCACGTCGACTGGAATAAC
CCCTTGCCAACCCCAGCGCCGAGAGTCCCTCTTCGGGTCTCCCGTCTGCCTCCGATGGGTGGCCCCTGCCGCTACTTGTTAGGTCCTGGCTCGCCGGGCGC
CTCGTCACCTCGGCAGCCTGGATCGCTTGTCGCCGCCGCAAACGTGCGCGCGTTTCGCCACGGGCCGCCCCGCGAAAAGCGGCGGGAACGTGGACCACG
CGCGCGCCCGCCATCGGCTCGCCGGGATCCCCACCGCGGCAGCGCCCCGCCACAGCTCAGGACGCGGCCGATCGACAAGCACATCTCCCGACTACCGG
GCCCCGCGGCGTGAACGTGGTCATGGAAAACCCGAGCGTGTGCGTTCCTGTCACGGGATCCCGCCGTGGGCAGTTCGCGGCAGGGGCCTACCCGTCGAG
GGGCTACACCGGACCGCCCCTCCAGCTGCATCGTGCAGCCCTAGTGCTGCAGCGAACTTGCCCCGGCCATCCCGTCCGCCCGCAAGCGGCTGGGGACAG
GTTCTCCGCCCATGCGCAGCCAGTGGCCTCATTCATGAGGGCGTGGCGGGCCGGGCCGCGTTGGGCCATCGCCGGTTGGGGGCCCCCACGCCGTCTACCT
GAGTGGACGCGCCGGGCGAGGCTCCCACCACCCCAGTCCTGGGGTCCCAGACGGCCTCCTGGGCGGAGTGTTCTACCCCGCGGCCACCCCCAGCCCGTC
TGCGGCCGAGGCCCTACTGGAGACTGCATTCCCCGCGGCCGGGGAACCGGCCACACCGGCAACGGCGCTCCTGACCCCACCCGCCTGCCACACTCCCCG
CGGCGCGGAGCCCCACCCATACTAAGGCCCCTACGCGCGGCCGACGACATGTTGATGGCTTCGCTGGGGGGGCGGTGACACGTCCACGAGGCGCCTCGC
CGCCGTCGGCCCTGCCCCAGCACGTTCCCCCGTCCCCCGGGGGTGCGGCGTCCTGCTCGCCCCCCGTGTAGGAACGCCGACCGGGCTGCGTCGTCGATGA
GGCAGTGGCCCGCTACGCGGCTCCTCTTCGCACGTGCTTTTGGCTGAAGAACGGGCACCCCCACCATTACGCAGTTGGGACGGCGCGCGGCTGCGAGGG
CCCAAATCTCTCCCATGCGAGTCGTGGTCGCGCCCGTGGACCTCTGAGGGGCAAGGCCGCTCGTCTGCTGAGCGGGGATGTTGCGCCCAGCAAATCGAG
GAAGGTTTGCTCGTAACTTACGGGTACCGAGACTCCTCGCTTGGCTCACCCATTGCGGCAGCCGCGGCCGCCGCCGGGACCAGTAACGGCGTGTCGACC
GCCCCTCGACAACGCCTGGTAGGACGCTCCCCTCCCGGCCCGGACACTAACCCCCGCAACAGCGAATTCCAGGGGCGTCTTCCGCCCAACAAAGACCCA
TCGTGCACGAATTGGGCTGGCGGGCAAGCATGTGCATCACGGACTAGGGTACCGCCTGCCTCCCCGCTGCCTCCGGGGCCCTGGCGCTCCGTCCGATTTT
ATCCCGCTCTGCTGCGATACTCAACCAACCGTTTCGCTTTATTCAGGCCGCCTCTGGGGCCCGGCCGAACACTTTCTCCCGACAAGTGGCGTGACGGACCT
GCCTTCGCGCCGCCCCGAAACAGGCGGGCCGGCCTCGCACCCCTGGGGAGCGGTCCGCCGCAGGCGGGCCTCCTGAGCGCGCGGGTGGCCGGCCAGCG
CGCATACCCCCTCGTCGCTGCCGCCCCCCCGCCCCGATCCCCGGCCCCGCTCGGCAAGACAGTGGAACCCGCCGCGCCCTTCACTCGCCCGGCTGGCCG
TATTTCGTGGGTCCTCACCTCAGTCGCGTCTGACAGATTAGACCTCGCCGCTGATGCCTAGGGTGGACACCACCTTCCCGCCCGCCACCACCCTGTGGA

GCGTGAGCGCACTTAAGTCTCCCTACCTCCCCAACTCAGCAGCGGCCCCAGGGCATCCGCGAGGCTCCGTTGACGCACGTGCCGTCGTCCCGCGCCCACT
GTGGCACCTCGGCGGCGACCCTCCGCTGCGGGATGCCTGGAGGAGCGGTCCAGCCTGGGCGCTCTGCCGGTCACCAACGGCGGCCGGGCGGGGGCCCTC
GCGGTCGGACCGAACTTCGGAGGGGTCCCCGGCAGCTGAGTCGAGCGCTGCTAGTGACCTCCACCTCGGCCTAGTCACACTTGCCCGGCTCCCAACCGCT
AAGACATAGGGGGCGTCGCGACGAGGGCAATGCGGGGGCAACCTTGCCACCCCCAAGCGACGTCGCCTCATGTAGGTTGGAGGGGCTGTCTTGTACTCC
ACCGAGGGCGACACGTAGCGTGCGGCGGCTCGACTCGAATCTTGCGCCTCGGTACGCGCCACGCGACCGATGACCTTTGCAGGCGACTCTGCGAGAATG
GGGCCCGCGTCCTGGGGCGTGCTAATTCACGACCGCCGCCGCTGGCCTCCAGGAGGTCCCAGGGGGCAGCACACGCCATTCGCGTCGGTGGTCTAGCCT
CGCGGCACCTCGACGAGTCCCAGGACGCGTCGGGCCCGGTTCTTCCTCTTCCTGTGTGGCAGTCCGGCCGGGTGACGCTTCCGCCGCTGGCTAATTCAGA
CCGCCGTCAGTCGACTGGGCTGCCCTAACCAGCCAGGGCACTCGTGGACTTCCGGCGCCGTCCGACTCCACGCGGCGCGCACAGTCGCCTCCCTCCTTCG
TACCATTCGGCCCTCCTCACCCCAGGGGGGGCTTGCCGCCATTCCCTACGCTGCCATTCGACGTCCCGCGCTACTCACCCATAGTGCCAATTGCGGCGTCG
CGGACACCGGCCTGGCGCAGCGCCAGAGCGCCACCTGTGACGTCGCTCACCGCGGGGGGAGCCCAGCCTCGACGCCCCAGATGGCTCGCGGGGGGGA
GGTCCCGAGGCCACCCTTCCAGGTGGCACAGATGCCCCTCCCTGGAGGGGGTGGAAAGCGCGCGGCGGGCCCAACACCGTGGGCGGCAGCTGGGCATG
CGCTCGGATAAGAGCGCCAGGATGGCAGGAGGGCCGATCTGGCGGGTGGTGGCCAGTGCGGGGCGGGGGGCGAAACTGTCTGGGGGTACCTGGAGCCC
AGGGTTCAGGGTCCGTGCCATGAGGCTGTCAGGCAAGCCAAGCCGTTAACGGCGGCCAGCGAGTGGGCAGGGAAGGTGACTAGCGCGAGCTACATATT
GCCGGCCGCCGCGCGGGGCGGCCAGCCGTGAGGAGCAATCTGAGGGACGGCCCACCGCCGGAGGGCCTGCACCATGAGGTGAGGCGGCGGGTCCCCG
CCACGCCGCGAGACGGTACGACCCGTTCCGGATGTGCACCACCGATGAAGAAGGCGCGCACATGCCGTTCGCTTTCGGCGTATGGTCTTCGCAGCCTCCG
GTGGGAGGCGCAGCCCGGAGGGTGCCCGCGCCACACGGATCAATGGGTTGGAGGGGCGGACTCGTGGTTTCGACCATTGGTGCAGCTAGAAACCGTCCC
AAGGCTCCACTCGATCGCACTGCCGTCGATCTCGGTTCTGACTCCCACCTATGCCAACACGGGAGTGCGCTATGAAACGCGGTTTTATAGTCGGTAATCG
CGACCGAACGCGGAGCGCCGTCTCGAGGCTTGTAACGCTCCGCGGTCGTTGTGGAGCGGACCGGGTAGTCAGCGTGACGTATTTCTAGCCCCCAGTGTGC
CGCCCGGCGAGAAAGTCCTCGCGGGAGCCCGTCGGCTGCCGCTGCGTACGCGGGGGAAAGCGGCCGAGGTACGTCTTCCTGGTGGCTTGCTGCCGCCAA
CCACCGATCGGCCCGGCTCGATGACAGCCGGCGAGAATGGGTTGGGGCCTACTTGGCCGGGTTGGCTGATCGTACGCAAGCAGCGTGCTGCGGGCCCAC
GCAAAGGGGAATGGCGGGGGAGCGCCGGCTGCCAATTCCCCCCGGCTGCACAGGCATGATCCGGTGTCAAGAACGGTCTCCGGCCCACGCGGCCGCGC
CTTGGCCCGAGTACGGCTGACTCAGAAGCCATTGGGTGCGAGGGCGTGGGGTGGTGGCGCATAGGGAGCCAGACCCGACGGAGCCAGGATGGTTCGAG
CAGTGTCACGGCCCGGGGTCATGTGGGGCCCGCCCGCACGGATGACCCGCGTCTCGATAATCGGTTGCAGGCGTGCCGTTCCCACGGGGCGCGCCGGTT
ATCGAACGGGTGCGCGCGTAGACTCGCTTCCGGGCCCCGGACCTGGGCCAGGCAGAAGCGGGGATTACCCGTCTTGATCGTAGACAGCCCGGTGCCCCC
GGGGGCCGACGTACAGCTGGCAAGCACCGGTCCGGCGATTACCCGACCTGGGTTGGCAGGTACACGTTACAGGGGTGGCGTGCCCCCCGGCGCTTGGCG
CCGGCTTGGAGGGACGGGGGGGGATCTAGCTCAGATGGCCAGCGAGCGTAGCTTCGACGGTGGCAAGAGCGAGCGGCGGCGTGCTTGGGGAGGCCGCG

GGCGGGTGCGACGTCCAGTTCGTCACACGCATGAGGGGTTTCCGGGGAGGAATCACCGCCTCGCGCAGGCGTGTCCGACACGACCCGTGTAGCGGGTGT

GAGACATTTAGGCGGAGGCCAGAACCCCTTGCCGGAGGGCTTGTAGTATATTGTCAATGGGGCCGACACCACGTGCGGATCGGGACTGAGAGCGCGTAG

AGCCTTGGCCCTTGCTATATTTGTGGAGCGGACACTGTGCTTCCCACACGTCACGCTGGCGTAAGCCGATGCCCGTCACCGCCCGCGCCCCAGGCCGAGT

GCGGGGGGCCGCAAGGTCCTGAAGCCGATCTACGATGGGCTGGGTGGGCGACGTCGTGGGCTAAGCTCGGCGGCCCCACCGTGAGGCGGACGTGGGGG

CCTGTACTAGAGATGGAGCGACGGTCGTCGATACAGTTCTTGCCAGGAGACCGAGCATTTTGCTAGGCACGGGCTGCATGGGCAATTCATTAACGGCGG

CGGGCCCACCGCGGCCAGCGGTGGCGGGGCGTAGTACCGGTCGGCAGGGATACGACCCCGGGCCGAAATTCGGGGCCGGCGGCCAGGGGGCGGCGCG

CCTGCGCGCCGTGACGCTGACGCCCTCCCGGGGTGCCGCGGGCTGGCCTGAGGCCCAAGGAGGCGCCTGGGGGTCGAGCGCGGGCCCGGGCGCACGCT

GATCCCGAGCCGACGAGCTTACGGATTGACCAAGAGCACCGCGAGCGTCCGAGCGCTGCGGGCGTGGTAACAACTACATCCCGGGTCGAGTTTGCGGCT

GGGACGTGGGATTGGCCGTGCAGACGCGCTGGGGGGGGAGCGGGGCTGGCCCCTCGCTCCGTATTCCCCACTCCACATGGTCCCCGGGCGTACGGGCGT

CCCCGACGGTTCGGGACGCGGGAATCGCGCAGGTGCCCCTCGGTGCGTGGCGGGCTGCGTGTAAGGCGCTTTCGCTGCATGCGGCCGTCTCGCGGACGT

GGCGTACGGGGGCGACCAGGGCGAATCCAAGGTGCGCCCAACGGGCCGAGGCGCTACAGATGGGGAGCCCCGGGCGGCCGTACGCAGGAGTCCAGTG

GCGTATGGAGGGAGGCCTGGGGCTCCGCAACGGCCGAGGCTATGCCCAGACCCCATGCCATTGTAGGCCGAACGCGATAGGTCACGCCGGTGATCCTGC

CGGGGTCGCGGGCCGGATCCACAAGGCCAGACCCATAGCGTAGTCTATAGGCGTCCGGGAAGGGTCGTGGGGGGGCTAA

>2017.TE.25009.1.1

CACGCCCCCCTCCCGGCCAGCCCGCCCCCCGCCCCAGCGTCGTACCCGGCGTCGCGACTCCCCGCCGTCGAGAGGCCTTTGCCGGGGCCCCGGCTAGCC

CGGGGCGAGCGGACCGCCGCGCGCGGGGGGTGTCACCCGGGGCACACCGCGCCAGCCTCCCGGGCATGCCACGCCGGGTTCCGCCCCCGCTGCTCCAA

TTTGGGGGGCCCCTAAGATCCCGTTCCCCAGGCCAGCCGCGCCCGCGGGCCAACATTGCCACACCGTGTCGCCCCCCCGCCTGCCGCACTGACCGTCTTG

CGGGCCCTGCTCCCTGGCCCGAGCACACCTCCGGGGACCCCCCTCGCTCGGCCGGGGGAGCCTTAAGCGCGGCACCGAGTAGGGGGTCGCGTGTTGCCG

CCGCGCTGACGCCCCGGCTGGGACCCCTTTCAGGTCGAGACGCCGGGGTGGCCATGCGGGTCCAGTCGCACGATGGCGCGGCGATGTGGCGCCGACTGC

CCCGCCTGTCTTCTCCGTGCCAGCCCCTCGCCACCATGCCGGTCCGGCCCGCACGGGGGTAGGAGGTGCTGCCGCGGCGGGAGGCGTAGGACGCCATAT

CCGAACCGGCGGGTCCCCCCGGCCACCGAGTCGACAGGACCATAGGCACCGCGTCCGGGCGGGCGCCCTTAACGCCGCCCCTTACTGGAGAAGCAGAG

CTCTGCCGAGGCACCCTGCCTAGCTGGCTGCGTCCCGCACCCCGCGATCCCCCTGGTCCACTTACCCAGCGGGATGCGGGGATGTTCGCAGCTATCACAC

GGTCGTGGCCTGCCTCCCTGGGCCGGCGAGAGCACCGCCACCTTCGTGTTCCTTGCCGCCGGGCGACGTGTCCGGGAAAGGCTTCGCCGCGCGCCCGCGC

GGAGCGCCGGGGAGTCCCGTGAGCGGCCGCAGGAACGTCACGGACCCCGGCTTGGTGCGGTCCCCCTCAGTGGTGCCCGACCAGAGACCGCGGAAAGA
CTCCAGGAAACGCCTCGGGGGGAAAGCAGGGCCGCGCACTTCCGCTCCACGCTAGCTGGGGGGACCACGACGGCAATACTGAACACCTGGCTCTTCGG
CAGCCCGGCCTATGAGGGGCCCTCTCGTCCCGGCCAAAGGCTATTTGCCTTCCTGTCCGGGCACCCTCGACCGCACCCGGGCGGGCGCAGGCTTCATTGC
GGCGCGACTCGCCAAAAGTCCCCGCGATCTCAGCGAGTACCCTGAGCGCTGGCCCTAGTCCGCCTGCCCCCTTAATGTCGCTCAGGTCTGCAATCTCTTA
CGACCGGCTCAGGTAGTGGCCGCACGGTGCGTAGGTCCGCTGCGGGCCGGGCCACTCGCGCCGATCGTGCCGTGCGGCGGCCCTGACACCGTATGCGTG
CGCGCTCCGCAGGCTCGCGAAAATCCTTCGCAGGCACTTGGCCCCGTTGCTAGACGTGGTGTCTCCTAACTGTGGCTCACGCACGGCGTTTAGGCCACGG
TAGCCCGCTGTTCGGTCCTTACGACTCGGCACACGCCCCCGTGCACGCCCTCGGGTTCCGCAGGCCAACGCCTGGCCCTAGGGTTGCGTGCCGGCCTGCC
CGCGATGCCACTTGCCCCACGTCCCCCACCTGGCCGGCGCAGACGGGTTCAGTGGGTGTCGAACGCCATTCAGTCCCCTGGGACTCCCCCGACCCCTCCC
ATGGCACCCGCCGCCTGGCCTTGGAGCTCCTGCGCGTTGGACCTTACTGCGGTCGTCAGCTTCTCGGCGCGCCCCGGACTAGTCCTGGGCGGCCAGCTCG
CGGGACCCTAGCGCGCACGCGTGCCCGATTGCCGTATCCCCTCGACGGCGCCCGGGCATAGGGTTCGATCCCGCTGCTATTGCGGGTGCGTCCAGACCTG
CCGCCGTTTGGCGATCCGAGTGTGCGAAGGGAGCCAGCCAGCGGGCCGCCTGGCCGCACTCCAGGATCCTGGCGCCAAGGGAGAGCCCTGCCAGCGCT
ACGCGAATACTCGACCTGTCCGCGCCCCGCCTCGGGCTTGCGAGATGGCCACTAGACGCTATCGGGTCGTCACCCCGTCGGCCCCACAGGGGCCCGGCTT
CGCGTGGCTTTCTTGTCGAAGGCCCCGAAATCCGGCAGGCCCCGACACCTTCACCGTGAGCGAGGACGCGCGGTGCGGGATGTCCAGTTCATGCACGCC
CCTCCACGGCCGCCTTCGCGCGGACAGCGTCGGGCGTGCCCAGGACGAGAACAGACCCTGGGCCAGCCTCAGGGCCCGGCCTAGGCGACCCTGGTGAC
CAGTTTCCACTCGGCACCCTCGGGCTTCGTGGCAGTGCAGTCATGCCCCCGGGTGCTCCCGCGCGACCGCGCACCTCCCACCCAAGGTTCTTGGTCAACG
CGCCGGATCTGACGTCGCGGTCCGGCCCGCGCCGTAGGCAAAAATGGGTTCGGCTCCCGCCACTCCGCCAGTTCGAGGGCGGTAAAGATCTCAGCCGCC
GTGTTTCGGGCTATCTCCCCGCGATCCCGGGCCACAATCGCAGGTGGCTGGGGCGTCGGCCAGACCCGTCTCGTCGGCCTTTCTGCGCACATTACCTCCGA
GCAAGGCAACCGCTCCGCCCCATTACAGGGGGTTGTCTGGTACCTCCGTCACGTACCCCCGCATCGCTGGCGGGCTGACCCAAGCCCCCTCCCATCCCGA
CCCTCACGCGCTCACGCCCCTTGCCGGGCGGGGTCGTGGCTGCCCAGGGTCCAGGGCCCGCCCCGGGGGCCGGTTGGTTCCAGGAACTGAGGGGGGGTC
GCCCAGCTGACCGCTCCTCTCGCGGGGTCTATTCGGCGTGGCCATCCGCAGGTGCGACAATGCCGGCTCCCATTGCCGGGCCGACCCTGGTCCCTGGCGC
TTCGGACTCCCGGGGAGCGCCTCCCTTCATCGGCGGCCCCTGCCCCCGTCATGACCCCCCTTACGCGCCGGTGGTAGCCGCGCGTGGTGGCGTGCCCCTCT
CCATCATTACGCTCCACAGGAGTGGCCACCGGTATGGGCGCGGTGCAGTCGTACATGTGTGCGGCCCTGTTTTGCGAACCCTTCCCGCGGTGACCTCCGC
CCCAGCGGCCTTCTTCGACCGTTAACGCCGGCCAGCACCCGAAGGGGGTCACACAAAGCCCTGCAAGGATGCGACGCTGCGTAGCCGCAGGTTAGCCGC
TTCCTTCCCAAAACAGTGTCAGAGCTGTCCGGCGGAACGTTAACGCCGCCGGGAGGATCTGCGGCTACCCTCAGGCCCGTCCCGTGGGAGTGGCCGGCG
TCGATGGGGGTACGCACCCCACTAGACGCGGATTTTGGCCAACGGGCACGGCCGCTTCCGAGTTCCCCTGCGGTCTGAGTGTTGGTACGTTCAGCCGGCC
GTTCCGTGGGGCAGAGCGCCGCACGCATGTCGCAGGAGCGCGCCCTCCGCGTCTTGCGGGCGGGGTCCTAACGAAGCCCCCTGGGCTTGGAGGTAGGGG

GCGCGGGGGAGCGATGGGAGCGCAAGCGTGCCATCGGGTCCGGCCCATAAGTGTGTATGGCGCACCGCGCTGCCACGATGTCCGGTGCGCGGGCGCCG
CTGGCTAGCTCACTCTCCCCCGACTCCGCCGCCGCGCCCCGAGACGTATGGAACAGCTGGCGCGCGTCCCCCGCCGCGGGCGGCAACGAACTAGATCCA
CCGCCCTCACGAGGAAGCGCGCCGCCACCACGTCTCCCCGGCCGACGCGGTCCCACCATCCGCTGTGCCGGCGCGCGAGCAGCCTGGCCCTCGGGGCGG
TCGCGCCAGCGCAGACACCCTGTCACGCGCACGGGAGCCCTGGCCACCGCGTCGACGCCCGGGCTAGCCCCGACTATCAGTACTGACCAGAGGGGCGC
CGGTTGTCCCGACTGGTTCCTCCCGAAACGCTGGCGGTGGGCCCGCACGATTGGTGCAGGCCCCCAAGAGGATACGGCTATTCGCGGCTGCCCGAGGCG
AGTCGGGCCAGACCGGTCGCGCGTGTGTGTGTTAGCCCTCCGTCGCGGCCAAGCCGGCGGCACCGGACTGGGCGACAGTAGCGTTCCTCTGTCACTAGTC
ACTCTAACGGACAAATGGAAAAATGGCACCTATCGCTACCGACGGGGTTTTCCTCCAGGATAAGCCTCCGCGCCCCCCACTGGACGCCAGCCATCCTTCC
CCGAACGGCTCGCCTGTGCCGGCTCCAACCCCGCGGGACCTCCCTTCGCGGCCCCGGCGGGGATCGGTGGATCGTCGCCGCCGGCGCCTACCGATCGCG
CCGCCACGGTCGTGCACCAACCAAGCACTGCTACACGCCTGTGCGGCCGAGGCCGCCCCATCCAGCAACGGGGCACCGTTGACCCGTCCCCCATGATGC
CGGACATGGTAGCCCCCACACATGCATCTTCGGGTAACCCGCGCCAGGTCCCCCCCCGCGCCGCCGCCGGCTTTCCCCCCGCTGGTGCTAAGCGGCAGAT
GCACCACTGACGGGACACACTCGCCCCCGATCCAGTGCGCTGCCGCCTGCGGCCCCCCACCGGACTGGTGCCTGGCGCTACACGGAGCCGGCCGCGCAC
TGTGACGTATGGATGCCCGAGGGGACATCCGCCCCGGACGCGTGCGCAAAAGACCACTCGCTCGACGCCACGACGTGTGCGGCCGGGGTGATGAACGT
CGGCCGGTGTGCCAGTGAGGCTTGCACCTACACTCCCTGGTCCACCGGGCCGTTCGAGCGGCACCGGGCGAAGAACCCGCAGCCTCCCGGCTTACGCAC
CGGCCTCGCGTCAACAGGCAACCACGACCGCAGGGCCGGCAGTGTGTGTCGCTGTGGAAAGGTGCGCCGCAGGCGACCCCCACGTCAGCCCACTACAA
AGCGTGGCAGCATGCTGCGCAGCGACCGCACGCCCCGACACAGCCCAATGCACAGCATCGGGGGGGCGGAATCGCCCCACGCCCGTAACATCCCGCGG
GTCGACCGCCTTACACGTCGACTGCCACACCCTGGTGCCGCAGTGGTATCCCGAGACCGCATGACCCGCCGCGAGCCCGCTGACCACCTGTCCTTGATCG
CAGTCGCCACGGGGCCGGAAGGTGTACCGCATACCGCACGTGCTGCGCGTGTGCAGCACGGGGGCACCCTGCATGTATGGATCCCTCTAAGCCGACCTG
CCACCCCAGGCCAGAGGCTAGAGTCTGCCGCCTAGGCCCCCCCCCGTGGTCGCCAGCCCCGGTGGCGGCCCACCCTCGGGGCGACTGACCGGCCACCAT
CCAGGGTAGAGAGGTTTGGCCCTAAATACGGGCGTGCCGAGGAGGTGTTTGGGGCGAGCGGCAGCAGGTTCGGCGGATCCTTGAGGGTGGGGCCTGGG
GTGGGACGGGGGCGGCCGAAACGCGAGCATAGGCCCCTGCCTGGCCCCTTTGCAGTCTGTACTTTTATTGACATGATACTAATACCGTGTTCCGCCCTGA
GATCGCACGCGGACCTACCGTGCCTCTCTCTGCATAGTGCTTCAAGAGCCCAGCGCGACTGGGGACCGGCTCCCGCAGCCGGCTCGGGGCGTAACGCTC
GGTGGAAGGGTCACCCGGATAGCTCCCGGTTAGTTTCGTACAGGGTGTCGGGGGCGCCCCCAGCGAGTCTCCTGGAGGTGCAGGGACGGCGGTGGTCCT
CGAGGGGGCGGACGTTGGCCGCATAACGGGTAGTCTCCAGGGTCACCTATGGTCTGGCTCGCCGTCAGGGTCTGTGAGCGTCGTGCTCACGCCTCCGCAA
CAGCCGGCGACCCCGGCTCGCAACGGCCAGCCGTGGGCGGGGGGGCGACCGGACGCAGTTCCGAGTCTAGGCATGGTGCCACCGCCCTCTCGTTGGGTT
AGTCTCGAATGGCTGTTTCGGGGGTCAGCGCGGCGGGGCCGCCGCTGGGAGCCCGCATAACCGTGGATACACTTGCTGGGTGCGCGCTCGAGGGCGGCC
CCCGAGGGCTGGAAAACCATTTCGGGATCGCCCCCGCGCTCCCGTCCGCACTGCGGGGTCCAGCCCGACGGGGGCGGGTTTCGTTGGGGCAGTACTCAC

GCCCACGGCGAGGCCCATCCAGAGAGTGAGGGTGTCGCTGGGGGGGCCTACCGGGCGGAACGCGCCCCGACTTAGGGCGCCGCAATGCACGACCAGTC

ACCGTCTGGGCGTCGCCGCGCCGTTGAGAACCCCAACACAGAAAGAGCTTGCCGCCTCTGGGGGAGCCAGGCCGAGAAGCGTGGGCGAGCGGCCACGC

TGCGGGCCCTCCGCGGACCGTGCGGCGCTCTCGGGTGACAAACCTAGTCCCCGTCCCAGGCCATGGGCGAGCAGCTGCGCCGGGACTGTGCGACGCCTA

TCCGGCACATTCGGTATTATTGGCCTGGGACGACAGCCGGCGACAGGGCGGACGAGGCGGCGTGGACTCGGGGTGCAGGGACGACGTCCACCCGTACT

GGTCAGTAGCCCAACGCGGAAGCCTGTCTGGGGCTTTGGCCCCTAGACATCGGTCCGGGGCGGGGCGCGTCATCGCTACGTTAGTGAGGGGGAGTCTGC

ACCGATAGGGTGTGGTGGTGTTGCTTAGATCGTTGACGGGCCGTGAGGTGGCGCTGCTGTTTTCCGGCTTACGGCTTACGACCTACCGCCCGCCGCGCCGG

CCCGAAGGCGGCGCCCCCGCACATCGCCCTTGGGTGGTAAAACCCGCCCTGCCGCCGCTACCCCCCCCCCAGGGGTGGCCGGAACGGCGAGGGCAGGA

CACGGTCGACGCACGCAGCGCCGTGACGCCCGGCCCGCGCTAGGGTTGGCACCCGTGGGACCTCGACAAACTTTCCGTACGATTGGGGGGCGCATACAG

CGGACGGGGAACCCGCCCGACGCACGCGCGGTCCCGAGGGGTGGGGGACCTGCAGCTGCGTCGCGCGGTCCGCGAGGGCGTCTTGCTAGACCATGGGC

GTTGCAATAGTGGGAAGGTGCCTCCCAGGCTGCGGGGCGCCGGACGCCCGACACCAAAGCTGTGCGAGGACAGAGAGGCTAGGCACGGATGGGTACAA

GCGAGCTGACAGCCTCCATCCTTGAGTGAGCACGCCGCGGCGAAATGCCGGCCGAAATGGGAGGGGCGCGACACCGGATCGTGATCGAGCGCGTGCGC

TTCGCCCACACGCCCTGACCCGCTGAGTGTCTCGGGTTTGAACCCCGTGCCCGGGGGCGCAGTGGGATAGGCACGACGGGCAGAGTTAGGGGCCTGGGC

ATAACGGCCCTGTCCGCGGCCGAGCGGGCCCGGGTCTTCTCGACCAGGGTGCCGGCCCCGGCGGGCCCCCCGCCCACGGACGGGGACCTGGAGTTGAGC

CAGGCCACAGGCGTGACCGGAATTGGAGCGCCAGGGGACAGCGGCCGCCCGCGCCAGGCCACGCCGATGGGGGCGTTCTGCCCAGGCGTCGCGCAATG

GGCGGACCGGTCTACGCGACACCGCCCGCCCGGCCTGGAACTCCCCCCGAGCACATTCGATATGGTCCGCATCAAGGCGGGAGGCATCGCCGGGGGCC

GCGCGGACCACCACCTTCGCGGGGGACGGTGCGGGTCGCGACGAGGCGGCGGCATGCGCGCGGGTGCCGTGAGCCCTAGAGTCAGCGGTTGCTGGTAT

GGGCGGACGGGCGCCCCCGACCTGCCGTGGACGCAGGCTCGCGGACTAGTCGACCCGCCATGGGTGCTGTGCGTGACGTTGGCCTTGGTCCAGGGCGCG

ACGCGCAATACCGCGCGGGCTGAGTATCGCGTCCACGGACAGGCGGCTGAGCCGCCCGGCGTAGCTGGTACACGGTCGGGCCGAAGTGGCAAAGAGCC

GGCTTGCGCCCCCAGCGCCGTTGCAGCAGGTCTGGACAGGCAGTACGGAGCGAACGCGGCGAGACACCCGGCTCCGGGCGTAGTCGGATCCGCCCGCC

AGCGGGCGCGCAGCGGTGTCGCGGGCTTGATGCGCCGGTCAGGGAGGTCGTCGCGAATGAGCCGGGTGGAGGGTGCAAGAAGCCAGACGATGGGAGC

ACCGAGTTGTCCGCGTGCGATTGCCGTCCCGGCGAAAGCGCCAGGCCGCGGCGCGTGTGGCAACGGCAAGTACCCGGAAACACGGCACGGGTCTCGTG

GCGCCGCGAGGCGGCGGGTGGCGCGCTCCGGGGGGCTGCGCAGCCCCTCAGCGCCGCTGCTAGTGCCAGACGCTTGCCCTTCTTGAGCGCGGCGGCGGG

AACTCGGTCGCGTGCGTTCAGGGCCCTCATCCGCAGACGTGAGGGGTGCGGGCCAGGGGGCGCAGAGGCGCCTTCCGAAACCCGTAAGGGATGTTCTGG

CGTTGGGGGGCCGCCGTCTTAGGGGGGTAGCGCCGCCTCAACTCCAGTATCGCCGCAAGGCCGGTGCCAGGTTCGATCCGGGGGCGGCGGCGTACCGCG

TCCACGCGTCTCCCAGAGGAGGGTCAAGGTGCATACGGGAGGGATCATACGGCAATCGGCGGCCGGGTTCCGCCATCTAACGTGGCGATCGCAGCGTAC

CAGAGCGAGGCCTATGGGCCATATCCCTGGCGGGAACGGGCTGCTTGCGCCCCGTCCTCGCCGACCCTCCGTACCGGTACGGGGCGAGGATCCCGACAC

ACAGCACGGGGGCGGCGCGCAGCACCCACCCCGTCCTCGGCCCAACAGACAAAGACCCCGCGGCGGGCTGGGGCACCCCCTTTCCGGCTACGTCCACC
GATTCTGTACAGGACAGCCGTTGCGATGGGCACCGGCGATCGGAGAGAAGGACGCCACAGATCAGCAGACGACTCGGGGAAACCACGGGACCGCGCG
TCGCGTCAACTGCCGCGGGAGTGAGAAGGGAGCGACAGGAGCGCGGAGGTAAGGCCAATGGGGCCAGTTGAAGCCGCCCACAGAGGCTGTGCGGGGC
CCCGCCGGGTTTAGGAGCTGTTCTTGGAGCCTGTCGTTAGGGGCGGGGGGGGGCTTACACGGCGCTGGGGGACCCACGAGGCGCAATCCGCCGCGGACT
GCTGGAGGGCTACCTGGGGCAGCACCCCAGGGGCGGCCATGGCGCTTGTTGACGGGGGCGCTAGGACCTTGTCCTAGGGCGCGCCGCTTTCGCCCACAG
GGCTTCGGTGGGCAGTCTATCTGTGCGGTTTCAGTCTCGTACGCACGTGATGCGGTGTAGCCTGAGCCCGGCAGTTGTCTAGAGTAACTAGCGCGCGGTTA
TGGCTTCGTCTGGAAACGGGCTGCAATGTTCCGGGTGTTCATCACACCACGTTCCGCTTTGCAGCTCACCCCCCGACCACACCCGGATTCGGACCGGTGGT
CCTTCCTCTGAGGTACTCGCCGCTCATACAACCGCGGTTCCCGCTGTGGTCGGCACGGCCCGGGTTGCAGGGGCGTGCCTTCCCGGGTAGTGTGCCTGCGC
GGCGTGCGGCTTCCGCAGTCCACGGACCGTTCCGGGGGTGAACGATGGAGGTGAGGGGACGAGTCCCTCCGAGATTAATGCCCCGCGGCAAGCCCGATC
AGCGCTGCGGTCCTGGGCTGTCAGCGCGCGCGGAGGAGCGCCCGATCGGGCGCGGGGGAATGAGAGTGTGGAGGATCATAATAGAGAGATCGCCCCGG
AGACTGCGAGGGGCATGGACGCGGAGGGACGGGGCGGGGGGTCGGCACGCCCGTGCGGCCTGTCGTCATATCCCACGCTGTCACTCGGCTGCGTCCGGC
ACTCCGGGCGGCCGGCGAGCAAAGCCGCCCTGCGCGGCCCGATCGAGGGTGCGGCGGCGCGACGTCGTGGCGTGGGAAATGCGGCGAGGGGCGGGGT
GGGGGACGTCATTGAACCTGCAGCGCGCACGGTGAGTACCGCGGCCCGTGGCGGGGCTGTACTGCGGGACCCCGGAGCGGTAGCGGGGGTTAGACTGG
GTGAGGGTAACCTGCTGCCCCGCGCACCACGTTCTTGGCCAGAGGCCCTGTGGGTGTTGGAGCAGGGTCGAGTATCCCTCCGTGCTTGCCTGTGTGGAGT
GGTTCGCCATGCAGTTGGACTCTTGGCGTCAGCTGGGGCCAGGCGGCGCCTCCGAGGTCGTAAAGGTCAACAGCGCTGTGAGATGGGCGCAGGAAGAGC
GCCAAAGACCTCCTGCGCCCCGATGTCCGCCTCGGGCGGACGTTCGGCCGCCTCGGGACTAGTTAAAGTGCGGCTTGGCTGGGGGCGCGGGACTCGGAA
GTCCGGGTGGAAGATGCGGGGAAGCTGCCCGTCGCCGGTACTGGGGGACTGTAGCTGTGATCCGGGACCACGAGGCATGCACCCGTCGTGGTCCAGCCG
GCCTGGCGCGGGGTCACAGAGCGAAACGGCGGACGCTAGGAACATTCGCTACCACCCCCCGGCGGCACCTTGCAGGGTCTACCCCAGTAAGGACCGGC
CCCAGCAGTGAGTGCGCCCGGGGGCTGGCAGCGGTAGATTGGGTCGCACGAGCTCACGTGGGCCGTTGACCGGAACCGGCAACGCCTGGGACTGGAGC
GCAAACTGGGGTACACTCTTCACGCCCGCTGCTCGTCATCGACCCCGGCGGGCGCCCGGGACCGGACCGGGGGCGACCCACCGGGGGACGGCGCGGCC
CCTGGATGGCCCTGCCCTCGGTCACGAAGCGGCCGAATGGTTTGGGGGGCCTCGGGACGCGGCCGTGGAGGTGCGGGGGGCCTGAAGTCCCTGCCTGGC
CCTCCTCCTAACGGCTTCACGCCCCTCAGGGCAGCTAGGGATTAACGTGGCCGGGTCACACGACCCGCCCAGCGCGGTACGCTGGGCCGCAGTGATTGC
GCGCGCCTGGCGGGTCCTCACCCCCCCACACACATCCGGGTCGGGGCGGAACTGGGTCCCGCCGTTCCCTCAGATCTGCCCCCTGACTTGAACATGCCCA
AAAACCGAAACCGGGCCGGCGCCCAGACCCCCGTGCTAAGGTTCCCACCGCCGACCCTAGCCCCCCCGGAGGTCCCCTGTACTCCGCACGCGACGTCGT
ATCAGGTCCCAGAGCCCGACAATGCATGCGCGTCCCGCTCTGCGCCATGCCCATTGCCCGACCAGCCCCGCCCAGCGCAGAGGCCCCGACTGCTGGCGC
CCTCTGTGGCGCTCGGCCGCTCGAGAGCACCTGCCGCCCGACCGCAGTCTGGCTACCTGCTCCACGGGGCGGCGCTCGTGGTCTCCCGTACTCGGAACGC

CACGCCCCGGGCTCGCGCGTCTCGCGGTCCGACGCATCGTGGTCTTACCTCCGACCCTTTCCTGGGGTCGAGCGCGGGGGGGCAGAGCGCGCTCATCCAG
CCCCGCGCGGCAGCACCCGCCCGCCCAGGGTGGTACCGCTCTCGAGAGTCCGGACGGCCGAACGCCAGGGCAACGGGCCGTGTGAAGACTCACCGTTT
CGGCCGACCGCCATCCTAGCTGGGGCGAGCCCCCGCGCGCGGCCGGATGCGTATGACGTGCGCGCGCTCGGTGCGATGGCGCACGTCGACTGGAATAAC
CCCTTGCCAACCCCAGCGCCGAGAGTCCCTCTTCGGGTCTCCCGTCTGCCTCCGATGGGTGGCCCCTGCCGGTACTTGTTAGGTCCTGGCTCGCCGGGCGC
CTCGTCACCTCGGCAGCCTGGATCGCTTGTCGCCGCCGCAAACGTGCGCGCGTTTCGCCACGGGCCGCCCCGCGAAAAGCGGCGGGAACGTGGACCACG
CGCGCGCCCGCCATCGGCTCGCCGGGATCCCCACCGCGGCAGCGCCCCGCCACAGCTCAGGACGCGGCCGATCGACAAGCACATCTCCCGACTACCGG
GCCCCGCGGCGTGAACGTGGTCATGGAAAACCCGAGCGTGTGCGTTCCTGTCACGGGATCCCGCCGTGGGCAGTTCGCGGCAGGGGCCTACCCGTCGAG
GGGCTACACCGGACCGCCCCTCCAGCTGCATCGTGCAGCCCTAGTGCTGCAGCGAACTTGCCCCGGCCATCCCGTCCGCCCGCAAGCGGCTGGGGACAG
GTTCTCCGCCCATGCGCAGCCAGTGGCCTCATTCATGAGGGCGTGGCGGGCCGGGCCGCGTTGGGCCATCGCCGGTTGGGGGCCCCCACGCCGTCTACCT
GAGTGGACGCGCCGGGCGAGGCTCCCACCACCCCAGTCCTGGGGTCCCAGACGGCCTCCTGGGCGGAGTGTTCTACCCCGCGGCCACCCCCAGCCCGTC
TGCGGCCGAGGCCCCACTGGAGACTGCATTCCCCGCGGCCGGGGAACCGGCCACACCGGCAACGGCGCTCCTGACCCCACCCGCCTGCCACACTCCCCG
CGGCGCGGAGCCCCACCCATACTAAGGCCCCTACGCGCGGCCGACGACATGTTGATGGCTTCGCTGGGGGGGCGGTGACACGTCCACGAGGCGCCTCGC
CGCCGTCGGCCCTGCCCCAGCACGTTCCCCCGTCCCCCGGGGGTGCGGCGTCCTGCTCGCCCCCCGTGTAGGAACGCCGACCGGGCTGCGTCGTCGATGA
GGCAGTGGCCCGCTACGCGGCTCCTCTTCGCACGTGCTTTTGGCTGAAGAACGGGCACCCCCACCATTACGCAGTTGGGACGGCGCGCGGCTGCGAGGG
CCCAAATCTCTCCCATGCGAGTCGTGGTCGCGCCCGTGGACCTCTGAGGGGCAAGGCCGCTCGTCTGCTGAGCGGGGATGTTGCGCCCAGCAAATCGAG
GAAGGTTTGCTCGTAACTTACGGGTACCGAGACTCCTCGCTTGGCTCACCCATTGCGGCAGCCGCGGCCGCCGCCGGGACCAGTAACGGCGTGTCGACC
GCCCCTCGACAACGCCTGGTAGGACGCTCCCCTCCCGGCCCGGACACTAACCCCCGCAACAGCGAATTCCAGGGGCGTCTTCCGCCCAACAAAGACCCA
TCGTGCACGAATTGGGCTGGCGGGCAAGCATGTGCATCACGGACTAGGGTACCGCCTGCCTCCCCGCTGCCTCCGGGGCCCTGGCGCTCCGTCCGATTTT
ATCCCGCTCTGCTGCGATACTCAACCAACCGTTTCGCTTTATTCAGGCCGCCTCTGGGGCCCGGCCGAACACTTTCTCCCGACAAGTGGCGTGACGGACCT
GCCTTCGCGCCGCCCCGAAACAGGCGGGCCGGCCTCGCACCCCTGGGGAGCGGTCCGCCGCAGGCGGGCCTCCTGAGCGCGCGGGTGGCCGGCCAGCG
CGCATACCCCCTCGTCGCTGCCGCCCCCCCCGCCCCGATCCCCGGCCCCGCTCGGCAAGACAGTGGAACCCGCCGCGCCCTTCACTCGCCCGGCTGGCCG
TATTTCGTGGGTCCTCACCTCAGTCGCGTCTGACAGATTAGACCTCGCCGCTGATGCCTAGGGTGGGACACCACCTTCCCGCCCGCCACCACCCTGTGGA
GCGTGAGCGCACTTAAGTCTCCCTACCTCCCCAACTCAGCAGCGGCCCCAGGGCATCCGCGAGGCTCCGTTGACGCACGTGCCGTCGTCCCGCGCCCACT
GTGGCACCTCGGCGGCGACCCTCCGCTGCGGGATGCCTGGAGGAGCGGTCCAGCCTGGGCGCTCTGCCGGTCACCAACGGCGGCCGGGCGGGGGCCCTC
GCGGTCGGACCGAACTTCGGAGGGGTCCCCGGCAGCTGAGTCGAGCGCTGCTAGTGACCTCCACCTCGGCCTAGTCACACTTGCCCGGCTCCCAACCGCT
AAGACATAGGGGGCGTCGCGACGAGGGCAATGCGGGGGCAACCTTGCCACCCCCAAGCGACGTCGCCTCATGTAGGTTGGAGGGGCTGTCTTGTACTCC

ACCGAGGGCGACACGTAGCGTGCGGCGGCTCGACTCGAATCTTGCGCCTCGGTACGCGCCACGCGACCGATGACCTTTGCAGGCGACTCTGCGAGAATG
GGGCCCGCGTCCTGGGGCGTGCTAAATCACGACCGCCGCCGCTGGCCTCCAGGAGGTCCCAGGGGGCAGCACACGCCATTCGCGTCGGTGGTCTAGCCT
CGCGGCACCTCGACGAGTCCCAGGACGCGTCGGGCCCGGTTCTTCCTCTTCCTGTGTGGCAGTCCGGCCGGGTGACGCTTCCGCCGCTGGCTAATTCAGA
CCGCCGTCAGTCGACTGGGCTGCCCTAACCAGCCAGGGCACTCGTGGACTTCCGGCGCCGTCCGACTCCACGCGGCGCGCACAGTCGCCTCCCTCCTTCG
TACCATTCGGCCCTCCTCACCCCAGGGGGGGCTTGCCGCCATTCCCTACGCTGCCATTCGACGTCCCGCGCTACTCACCCATAGTGCCAATTGCGGCGTCG
CGGACACCGGCCTGGCGCAGCGCCAGAGCGCCACCTGTGACGTCGCTCACCGCGGGGGGAGCCCAGCCTCGACGCCCCAGATGGCTCGCGGGGGGGA
GGTCCCGAGGCCACCCTTCCAGGTGGCACAGATGCCCCTCCCTGGAGGGGGTGGAAAGCGCGCGGCGGGCCCAACACCGTGGGCGGCAGCTGGGCATG
CGCTCGGATAAGAGCGCCAGGATGGCAGGAGGGCCGATCTGGCGGGTGGTGGCCAGTGCGGGGCGGGGGGCGAAACTGTCTGGGGGTACCTGGAGCCC
AGGGTTCAGGGTCCGTGCCATGAGGCTGTCAGGCAAGCCAAGCCGTTAACGGCGGCCAGCGAGTGGGCAGGGAAGGTGACTAGCGCGAGCTACATATT
GCCGGCCGCCGCGCGGGGCGGCCAGCCGTGAGGAGCAATCTGAGGGACGGCCCACCGCCGGAGGGCCTGCACCATGAGGTGAGGCGGCGGGTCCCCG
CCACGCCGCGAGACGGTACGACCCGTTCCGGATGTGCACCACCGATGAAGAAGGCGCGCACATGCCGTTCGCTTTCGGCGTATGGTCTTCGCAGCCTCCG
GTGGGAGGCGCAGCCCGGAGGGTGCCCGCGCCACACGGATCAATGGGTTGGAGGGGCGGACTCGTGGTTTCGACCATTGGTGCAGCTAGAAACCGTCCC
AAGGCTCCACTCGATCGCACTGCCGTCGATCTCGGTTCTGACTCCCACCTATGCCAACACGGGAGTGCGCTATGAAACGCGGTTTTATAGTCGGTAATCG
CGACCGAACGCGGAGCGCCGTCTCGAGGCTTGTAACGCTCCGCGGTCGTTGTGGAGCGGACCGGGTAGTCAGCGTGACGTATTTCTAGCCCCCAGTGTGC
CGCCCGGCGAGAAAGTCCTCGCGGGAGCCCGTCGGCTGCCGCTGCGTACGCGGGGAAAGCGGCCGAGGTACGTCTTCCTGGTGGCTTGCTGCCGCCAA
CCACCGATCGGCCCGGCTCGATGACAGCCGGCGAGAATGGGTTGGGGCCTACTTGGCCGGGTTGGCTGATCGTACGCAAGCAGCGTGCTGCGGGCCCAC
GCAAAGGGGAATGGCGGGGGAGCGCCGGCTGCCAATTCCCCCCGGCTGCACAGGCATGATCCGGTGTCAAGAACGGTCTCCGGCCCACGCGGCCGCGC
CTTGGCCCGAGTACGGCTGACTCAGAAGCCGTTGGGTGCGAGGGCGTGGGGTGGTGGCGCATAGGGAGCCAGACCCGACGGAGCCAGGATGGTTCGAG
CAGTGTCACGGCCCGGGGTCATGTGGGGCCCGCCCGCACGGATGACCCGCGTCTCGATAATCGGTTGCAGGCGTGCCGTTCCCACGGGGCGCGCCGGTT
ATCGAACGGGTGCGCGCGTAGACTCGCTTCCGGGCCCCGGACCTGGGCCAGGCAGAAGCGGGGATTACCCGTCTTGATCGCAGACAGCCCGGTGCCCCC
GGGGGCCGACGTACAGCTGGCAAGCACCGGTCCGGCGATTACCCGACCTGGGTTGGCAGGTACACGTTACAGGGGTGGCGTGCCCCCCGGCGCTTGGCG
CCGGCTTGGAGGGACGGGGGGGGATCTAGCTCAGATGGCCAGCGAGCGTAGCTTCGACGGTGGCAAGAGCGAGCGGCGGCGTGCTTGGGGAGGCCGCG
GGCGGGTGCGACGTCCAGTTCGTCACACGCATGAGGGGTTTCCGGGGAGGAATCACCGCCTCGCGCAGGCGTGTCCGACACGACCCGTGTAGCGGGTGT
GAGACATTTAGGCGGAGGCCAGAACCCCTTGCCGGAGGGCTTGTAGTATATTGTCAATGGGGCCGACACCACGTGCGGATCGGGACTGAGAGCGCGTAG
AGCCTTGGCCCTTGCTATATTTGTGGAGCGGACACTGTGCTTCCCACACGTCACGCTGGCGTAAGCCGATGCCCGTCACCGCCCGCGCCCCAGGCCGAGT
GCGGGGGGCCGCAAGGTCCTGAAGCCGATCTACGATGGGCTGGGTGGGCGACGTCGTGGGCTAAGCTCGGCGGCCCCACCGTGAGGCGGACGTGGGGG

CCTGTACTAGAGATGGAGCGACGGTCGTCGATACAGTTCTTGCCAGGAGACCGAGCATTTTGCTAGGCACGGGCTGCATGGGCAATTCATTAACGGCGG

CGGGCCCACCGCGGCCAGCGGTGGCGGGGCGTAGTACCGGTCGGCAGGGATACGACCCCGGGCCGAAATTCGGGGCCGGCGGCCAGGGGGCGGCGCG

CCTGCGCGCCGTGACGCTGACGCCCTCCCGGGGTGCCGCGGGCTGGCCTGAGGCCCAAGGAGGCGCCTGGGGGTCGAGCGCGGGCCCGGGCGCACGCT

GATCCCGAGCCGACGAGCTTACGGATTGACCAAGAGCACCGCGAGCGTCCGAGCGCTGCGGGCGTGGTAACAACTACATCCCGGGTCGAGTTTGCGGCT

GGGACGTGGGATTGGCCGTGCAGACGCGCTGGGGGGGGAGCGGGGCTGGCCCCTCGCTCCGTATTCCCCACTCCACATGGTCCCCGGGCGTACGGGCGT

CCCCGACGGTTCGGGACGCGGGAATCGCGCAGGTGCCCCTCGGTGCGTGGCGGGCTGCGTGTAAGGCGCTTTCGCTGCATGCGGCCGTCTCGCGGACAT

GGCGTACGGGGGCGACCAGGGCGAATCCAAGGTGCGCCCAACGGGCCGAGGCGCTACAGATGGGGAGCCCCGGGCGGCCGTACGCAGGAGTCCAGTG

GCGTATGGAGGGAGGCCTGGGGCTCCGCAACGGCCGAGGCTATGCCCAGACCCCATGCCATTGTAGGCCGAACGCGATAGGTCACGCCGGTGATCCTGC

CGGGGTCGCGGGCCGGATCCACAAGGCCAGACCCATAGCGTAGTCTATAGGCGTCCGGGAAGGGTCGTGGGGGGGCTAA

>2017.TE.25009.1.3

CACGCCCCCCTCCCGGCCAGCCCGCCCCCCGCCCCAGCGTCGTACCCGGCGTCGCGACTCCCCGCCGTCGAGAGGCCTTTGCCGGGGCCCCGGCTAGCC

CGGGGCGAGCGGACCGCCGCGCGCGGGGGGTGTCACCCGGGGCACACCGCGCCAGCCTCCCGGGCATGCCACGCCGGGTTCCGCCCCCGCTGCTCCAA

TTTGGGGGGCCCCTAAGATCCCGTTCCCCAGGCCAGCCGCGCCCGCGGGCCAACATTGCCACACCGTGTCGCCCCCCCGCCTGCCGCACTGACCGTCTTG

CGGGCCCTGCTCCCTGGCCCGAGCACACCTCCGGGGACCCCCCTCGCTCGGCCGGGGGAGCCTTAAGCGCGGCACCGAGTAGGGGGTCGCGTGTTGCCG

CCGCGCTGACGCCCCGGCTGGGACCCCTTTCAGGTCGAGACGCCGGGGTGGCCATGCGGGTCCAGTCGCACGATGGCGCGGCGATGTGGCGCCGACTGC

CCCGCCTGTCTTCTCCGTGCCAGCCCCTCGCCACCATGCCGGTCCGGCCCGCACGGGGGTAGGAGGTGCTGCCGCGGCGGGAGGCGTAGGACGCCATAT

CCGAACCGGCGGGTCCCCCCGGCCACCGAGTCGACAGGACCATAGGCACCGCGTCCGGGCGGGCGCCCTTAACGCCGCCCCTTACTGGAGAAGCAGAG

CTCTGCCGAGGCACCCTGCCTAGCTGGCTGCGTCCCGCACCCCGCGATCCCCCTGGTCCACTTACCCAGCGGGATGCGGGGATGTTCGCAGCTATCACAC

GGTCGTGGCCTGCCTCCCTGGGCCGGCGAGAGCACCGCCACCTTCGTGTTCCTTGCCGCCGGGCGACGTGTCCGGGAAAGGCTTCGCCGCGCGCCCGCGC

GGAGCGCCGGGGAGTCCCGTGAGCGGCCGCAGGAACGTCACGGACCCCGGCTTGGTGCGGTCCCCCTCAGTGGTGCCCGACCAGAGACCGCGGAAAGA

CTCCAGGAAACGCCTCGGGGGGAAAGCAGGGCCGCGCACTTCCGCTCCACGCTAGCTGGGGGGACCACGACGGCAATACTGAACACCTGGCTCTTCGG

CAGCCCGGCCTATGAGGGGCCCTCTCGTCCCGGCCAAAGGCTATTTGCCTTCCTGTCCGGGCACCCTCGACCGCACCCGGGCGGGCGCAGGCTTCATTGC

GGCGCGACTCGCCAAAAGTCCCCGCGATCTCAGCGAGTACCCTGAGCGCTGGCCCTAGTCCGCCTGCCCCCTTAATGTCGCTCAGGTCTGCAATCTCTTA

CGACCGGCTCAGGTAGTGGCCGCACGGTGCGTAGGTCCGCTGCGGGCCGGGCCACTCGCGCCGATCGTGCCGTGCGGCGGCCCTGACACCGTATGCGTG

CGCGCTCCGCAGGCTCGCGAAAATCCTTCGCAGGCACTTGGCCCCGTTGCTAGACGTGGTGTCTCCTAACTGTGGCTCACGCACGGCGTTTAGGCCACGG
TAGCCCGCTGTTCGGTCCTTACGACTCGGCACACGCCCCCGTGCACGCCCTCGGGTTCCGCAGGCCAACGCCTGGCCCTAGGGTTGCGTGCCGGCCTGCC
CGCGATGCCACTTGCCCCACGTCCCCCACCTGGCCGGCGCAGACGGGTTCAGTGGGTGTCGAACGCCATTCAGTCCCCTGGGACTCCCCCGACCCCTCCC
ATGGCACCCGCCGCCTGGCCTTGGAGCTCCTGCGCGTTGGACCTTACTGCGGTCGTCAGCTTCTCGGCGCGCCCCGGACTAGTCCTGGGCGGCCAGCTCG
CGGGACCCTAGCGCGCACGCGTGCCCGATTGCCGTATCCCCTCGACGGCGCCCGGGCATAGGGTTCGATCCCGCTGCTATTGCGGGTGCGTCCAGACCTG
CCGCCGTTTGGCGATCCGAGTGTGCGAAGGGAGCCAGCCAGCGGGCCGCCTGGCCGCACTCCAGGATCCTGGCGCCAAGGGAGAGCCCTGCCAGCGCT
ACGCGAATACTCGACCTGTCCGCGCCCCGCCTCGGGCTTGCGAGATGGCCACTAGACGCTATCGGGTCGTCACCCCGTCGGCCCCACAGGGGCCCGGCTT
CGCGTGGCTTTCTTGTCGAAGGCCCCGAAATCCGGCAGGCCCCGACACCTTCACCGTGAGCGAGGACGCGCGGTGCGGGATGTCCAGTTCATGCACGCC
CCTCCACGGCCGCCTTCGCGCGGACAGCGTCGGGCGTGCCCAGGACGAGAACAGACCCTGGGCCAGCCTCAGGGCCCGGCCTAGGCGACCCTGGTGAC
CAGTTTCCACTCGGCACCCTCGGGCTTCGTGGCAGTGCAGTCATGCCCCCGGGTGCTCCCGCGCGACCGCGCACCTCCCACCCAAGGTTCTTGGTCAACG
CGCCGGATCTGACGTCGCGGTCCGGCCCGCGCCGTAGGCAAAAATGGGTTCGGCTCCCGCCACTCCGCCAGTTCGAGGGCGGTAAAGATCTCAGCCGCC
GTGTTTCGGGCTATCTCCCCGCGATCCCGGGCCACAATCGCAGGTGGCTGGGGCGTCGGCCAGACCCGTCTCGTCGGCCTTTCTGCGCACATTACCTCCGA
GCAAGGCAACCGCTCCGCCCCATTACAGGGGGTTGTCTGGTACCTCCGTCACGTACCCCCGCATCGCTGGCGGGCTGACCCAAGCCCCCTCCCATCCCGA
CCCTCACGCGCTCACGCCCCTTGCCGGGCGGGGTCGTGGCTGCCCAGGGTCCAGGGCCCGCCCCGGGGGCCGGTTGGTTCCAGGAACTGAGGGGGGGTC
GCCCAGCTGACCGCTCCTCTCGCGGGGTCTATTCGGCGTGGCCATCCGCAGGTGCGACAATGCCGGCTCCCATTGCCGGGCCGACCCTGGTCCCTGGCGC
TTCGGACTCCCGGGGAGCGCCTCCCTTCATCGGCGGCCCCTGCCCCCGTCATGACCCCCCTTACGCGCCGGTGGTAGCCGCGCGTGGTGGCGTGCCCCTCT
CCATCATTACGCTCCACAGGAGTGGCCACCGGTATGGGCGCGGTGCAGTCGTACATGTGTGCGGCCCTGTTTTGCGAACCCTTCCCGCGGTGACCTCCGC
CCCAGCGGCCTTCTTCGACCGTTAACGCCGGCCAGCACCCGAAGGGGGTCACACAAAGCCCTGCAAGGATGCGACGCTGCGTAGCCGCAGGTTAGCCGC
TTCCTTCCCAAAACAGTGTCAGAGCTGTCCGGCGGAACGTTAACGCCGCCGGGAGGATCTGCGGCTACCCTCAGGCCCGTCCCGTGGGAGTGGCCGGCG
TCGATGGGGGTACGCACCCCACTAGACGCGGATTTTGGCCAACGGGCACGGCCGCTTCCGAGTTCCCCTGCGGTCTGAGTGTTGGTACGTTCAGCCGGCC
GTTCCGTGGGGCAGAGCGCCGCACGCATGTCGCAGGAGCGCGCCCTCCGCGTCTTGCGGGCGGGGTCCTAACGAAGCCCCCTGGGCTTGGAGGTAGGGG
GCGCGGGGGAGCGATGGGAGCGCAAGCGTGCCATCGGGTCCGGCCCATAAGTGTGTATGGCGCACCGCGCTGCCACGATGTCCGGTGCGCGGGCGCCG
CTGGCTAGCTCACTCTCCCCCGACTCCGCCGCCGCGCCCCGAGACGTATGGAACAGCTGGCGCGCGTCCCCCGCCGCGGGCGGCAACGAACTAGATCCA
CCGCCCTCACGAGGAAGCGCGCCGCCACCACGTCTCCCCGGCCGACGCGGTCCCACCATCCGCTGTGCCGGCGCGCGAGCAGCCTGGCCCTCGGGGCGG
TCGCGCCAGCGCAGACACCCTGTCACGCGCACGGGAGCCCTGGCCACCGCGTCGACGCCCGGGCTAGCCCCGACTATCAGTACTGACCAGAGGGGCGC
CGGTTGTCCCGACTGGTTCCTCCCGAAACGCTGGCGGTGGGCCCGCACGATTGGTGCAGGCCCCCAAGAGGATACGGCTATTCGCGGCTGCCCGAGGCG

AGTCGGGCCAGACCGGTCGCGCGTGTGTGTGTTAGCCCTCCGTCGCGGCCAAGCCGGCGGCACCGGACTGGGCGACAGTAGCGTTCCTCTGTCACTAGTC
ACTCTAACGGACAAATGGAAAAATGGCACCTATCGCTACCGACGGGGTTTTCCTCCAGGATAAGCCTCCGCGCCCCCCACTGGACGCCAGCCATCCTTCC
CCGAACGGCTCGCCTGTGCCGGCTCCAACCCCGCGGGACCTCCCTTCGCGGCCCCGGCGGGGATCGGTGGATCGTCGCCGCCGGCGCCTACCGATCGCG
CCGCCACGGTCGTGCACCAACCAAGCACTGCTACACGCCTGTGCGGCCGAGGCCGCCCCATCCAGCAACGGGGCACCGTTGACCCGTCCCCCATGATGC
CGGACATGGTAGCCCCCACACATGCATCTTCGGGTAACCCGCGCCAGGTCCCCCCCCGCGCCGCCGCCGGCTTTCCCCCCGCTGGTGCTAAGCGGCAGAT
GCACCACTGACGGGACACACTCGCCCCCGATCCAGTGCGCTGCCGCCTGCGGCCCCCCACCGGACTGGTGCCTGGCGCTACACGGAGCCGGCCGCGCAC
TGTGACGTATGGATGCCCGAGGGGACATCCGCCCCGGACGCGTGCGCAAAAGACCACTCGCTCGACGCCACGACGTGTGCGGCCGGGGTGATGAACGT
CGGCCGGTGTGCCAGTGAGGCTTGCACCTACACTCCCTGGTCCACCGGGCCGTTCGAGCGGCACCGGGCGAAGAACCCGCAGCCTCCCGGCTTACGCAC
CGGCCTCGCGTCAACAGGCAACCACGACCGCAGGGCCGGCAGTGTGTGTCGCTGTGGAAAGGTGCGCCGCAGGCGACCCCCACGTCAGCCCACTACAA
AGCGTGGCAGCATGCTGCGCAGCGACCGCACGCCCCGACACAGCCCAATGCACAGCATCGGGGGGGCGGAATCGCCCCACGCCCGTAACATCCCGCGG
GTCGACCGCCTTACACGTCGACTGCCACACCCTGGTGCCGCAGTGGTATCCCGAGACCGCATGACCCGCCGCGAGCCCGCTGACCACCTGTCCTTGATCG
CAGTCGCCACGGGGCCGGAAGGTGTACCGCATACCGCACGTGCTGCGCGTGTGCAGCACGGGGGCACCCTGCATGTATGGATCCCTCTAAGCCGACCTG
CCACCCCAGGCCAGAGGCTAGAGTCTGCCGCCTAGGCCCCCCCCCGTGGTCGCCAGCCCCGGTGGCGGCCCACCCTCGGGGCGACTGACCGGCCACCAT
CCAGGGTAGAGAGGTTTGGCCCTAAATACGGGCGTGCCGAGGAGGTGTTTGGGGCGAGCGGCAGCAGGTTCGGCGGATCCTTGAGGGTGGGGCCTGGG
GTGGGACGGGGGCGGCCGAAACGCGAGCATAGGCCCCTGCCTGGCCCCTTTGCAGTCTGTACTTTTATTGACATGATACTAATACCGTGTTCCGCCCTGA
GATCGCACGCGGACCTACCGTGCCTCTCTCTGCATAGTGCTTCAAGAGCCCAGCGCGACTGGGGACCGGCTCCCGCAGCCGGCTCGGGGCGTAACGCTC
GGTGGAAGGGTCACCCGGATAGCTCCCGGTTAGTTTCGTACAGGGTGTCGGGGGCGCCCCCAGCGAGTCTCCTGGAGGTGCAGGGACGGCGGTGGTCCT
CGAGGGGGCGGACGTTGGCCGCATAACGGGTAGTCTCCAGGGTCACCTATGGTCTGGCTCGCCGTCAGGGTCTGTGAGCGTCGTGCTCACGCCTCCGCAA
CAGCCGGCGACCCCGGCTCGCAACGGCCAGCCGTGGGCGGGGGGGCGACCGGACGCAGTTCCGAGTCTAGGCATGGTGCCACCGCCCTCTCGTTGGGTT
AGTCTCGAATGGCTGTTTCGGGGGTCAGCGCGGCGGGGCCGCCGCTGGGAGCCCGCATAACCGTGGATACACTTGCTGGGTGCGCGCTCGAGGGCGGCC
CCCGAGGGCTGGAAAACCATTTCGGGATCGCCCCCGCGCTCCCGTCCGCACTGCGGGGTCCAGCCCGACGGGGGCGGGTTTCGTTGGGGCAGTACTCAC
GCCCACGGCGAGGCCCATCCAGAGAGTGAGGGTGTCGCTGGGGGGGCCTACCGGGCGGAACGCGCCCCGACTTAGGGCGCCGCAATGCACGACCAGTC
ACCGTCTGGGCGTCGCCGCGCCGTTGAGAACCCCAACACAGAAAGAGCTTGCCGCCTCTGGGGGAGCCAGGCCGAGAAGCGTGGGCGAGCGGCCACGC
TGCGGGCCCTCCGCGGACCGTGCGGCGCTCTCGGGTGACAAACCTAGTCCCCGTCCCAGGCCATGGGCGAGCAGCTGCGCCGGGACTGTGCGACGCCTA
TCCGGCACATTCGGTATTATTGGCCTGGGACGACAGCCGGCGACAGGGCGGACGAGGCGGCGTGGACTCGGGGTGCAGGGACGACGTCCACCCGTACT
GGTCAGTAGCCCAACGCGGAAGCCTGTCTGGGGCTTTGGCCCCTAGACATCGGTCCGGGGCGGGGCGCGTCATCGCTACGTTAGTGAGGGGGAGTCTGC

ACCGATAGGGTGTGGTGGTGTTGCTTAGATCGTTGACGGGCCGTGAGGTGGCGCTGCTGTTTTCCGGCTTACGGCTTACGACCTACCGCCCGCCGCGCCGG

CCCGAAGGCGGCGCCCCCGCACATCGCCCTTGGGTGGTAAAACCCGCCCTGCCGCCGCTACCCCCCCCCCAGGGGTGGCCGGAACGGCGAGGGCAGGA

CACGGTCGACGCACGCAGCGCCGTGACGCCCGGCCCGCGCTAGGGTTGGCACCCGTGGGACCTCGACAAACTTTCCGTACGATTGGGGGGCGCATACAG

CGGACGGGGAACCCGCCCGACGCACGCGCGGTCCCGAGGGGTGGGGGACCTGCAGCTGCGTCGCGCGGTCCGCGAGGGCGTCTTGCTAGACCATGGGC

GTTGCAATAGTGGGAAGGTGCCTCCCAGGCTGCGGGGCGCCGGACGCCCGACACCAAAGCTGTGCGAGGACAGAGAGGCTAGGCACGGATGGGTACAA

GCGAGCTGACAGCCTCCATCCTTGAGTGAGCACGCCGCGGCGAAATGCCGGCCGAAATGGGAGGGGCGCGACACCGGATCGTGATCGAGCGCGTGCGC

TTCGCCCACACGCCCTGACCCGCTGAGTGTCTCGGGTTTGAACCCCGTGCCCGGGGGCGCAGTGGGATAGGCACGACGGGCAGAGTTAGGGGCCTGGGC

ATAACGGCCCTGTCCGCGGCCGAGCGGGCCCGGGTCTTCTCGACCAGGGTGCCGGCCCCGGCGGGCCCCCCGCCCACGGACGGGGACCTGGAGTTGAGC

CAGGCCACAGGCGTGACCGGAATTGGAGCGCCAGGGGACAGCGGCCGCCCGCGCCAGGCCACGCCGATGGGGGCGTTCTGCCCAGGCGTCGCGCAATG

GGCGGACCGGTCTACGCGACACCGCCCGCCCGGCCTGGAACTCCCCCCGAGCACATTCGATATGGTCCGCATCAAGGCGGGAGGCATCGCCGGGGGCC

GCGCGGACCACCACCTTCGCGGGGGACGGTGCGGGTCGCGACGAGGCGGCGGCATGCGCGCGGGTGCCGTGAGCCCTAGAGTCAGCGGTTGCTGGTAT

GGGCGGACGGGCGCCCCCGACCTGCCGTGGACGCAGGCTCGCGGACTAGTCGACCCGCCATGGGTGCTGTGCGTGACGTTGGCCTTGGTCCAGGGCGCG

ACGCGCAATACCGCGCGGGCTGAGTATCGCGTCCACGGACAGGCGGCTGAGCCGCCCGGCGTAGCTGGTACACGGTCGGGCCGAAGTGGCAAAGAGCC

GGCTTGCGCCCCCAGCGCCGTTGCAGCAGGTCTGGACAGGCAGTACGGAGCGAACGCGGCGAGACACCCGGCTCCGGGCGTAGTCGGATCCGCCCGCC

AGCGGGCGCGCAGCGGTGTCGCGGGCTTGATGCGCCGGTCAGGGAGGTCGTCGCGAATGAGCCGGGTGGAGGGTGCAAGAAGCCAGACGATGGGAGC

ACCGAGTTGTCCGCGTGCGATTGCCGTCCCGGCGAAAGCGCCAGGCCGCGGCGCGTGTGGCAACGGCAAGTACCCGGAAACACGGCACGGGTCTCGTG

GCGCCGCGAGGCGGCGGGTGGCGCGCTCCGGGGGGCTGCGCAGCCCCTCAGCGCCGCTGCTAGTGCCAGACGCTTGCCCTTCTTGAGCGCGGCGGCGGG

AACTCGGTCGCGTGCGTTCAGGGCCCTCATCCGCAGACGTGAGGGGTGCGGGCCAGGGGGCGCAGAGGCGCCTTCCGAAACCCGTAAGGGATGTTCTGG

CGTTGGGGGGCCGCCGTCTTAGGGGGGTAGCGCCGCCTCAACTCCAGTATCGCCGCAAGGCCGGTGCCAGGTTCGATCCGGGGGCGGCGGCGTACCGCG

TCCACGCGTCTCCCAGAGGAGGGTCAAGGTGCATACGGGAGGGATCATACGGCAATCGGCGGCCGGGTTCCGCCATCTAACGTGGCGATCGCAGCGTAC

CAGAGCGAGGCCTATGGGCCATATCCCTGGCGGGAACGGGCTGCTTGCGCCCCGTCCTCGCCGACCCTCCGTACCGGTACGGGGCGAGGATCCCGACAC

ACAGCACGGGGGCGGCGCGCAGCACCCACCCCGTCCTCGGCCCAACAGACAAAGACCCCGCGGCGGGCTGGGGCACCCCCTTTCCGGCTACGTCCACC

GATTCTGTACAGGACAGCCGTTGCGATGGGCACCGGCGATCGGAGAGAAGGACGCCACAGATCAGCAGACGACTCGGGGAAACCACGGGACCGCGCG

TCGCGTCAACTGCCGCGGGAGTGAGAAGGGAGCGACAGGAGCGCGGAGGTAAGGCCAATGGGGCCAGTTGAAGCCGCCCACAGAGGCTGTGCGGGGC

CCCGCCGGGTTTAGGAGCTGTTCTTGGAGCCTGTCGTTAGGGGCGGGGGGGGGCTTACACGGCGCTGGGGGACCCACGAGGCGCAATCCGCCGCGGACT

GCTGGAGGGCTACCTGGGGCAGCACCCCAGGGGCGGCCATGGCGCTTGTTGACGGGGGCGCTAGGACCTTGTCCTAGGGCGCGCCGCTTTCGCCCACAG

GGCTTCGGTGGGCAGTCTATCTGTGCGGTTTCAGTCTCGTACGCACGTGATGCGGTGTAGCCTGAGCCCGGCAGTTGTCTAGAGTAACTAGCGCGCGGTTA
TGGCTTCGTCTGGAAACGGGCTGCAATGTTCCGGGTGTTCATCACACCACGTTCCGCTTTGCAGCTCACCCCCCGACCACACCCGGATTCGGACCGGTGGT
CCTTCCTCTGAGGTACTCGCCGCTCATACAACCGCGGTTCCCGCTGTGGTCGGCACGGCCCGGGTTGCAGGGGCGTGCCTTCCCGGGTAGTGTGCCTGCGC
GGCGTGCGGCTTCCGCAGTCCACGGACCGTTCCGGGGGTGAACGATGGAGGTGAGGGGACGAGTCCCTCCGAGATTAATGCCCCGCGGCAAGCCCGATC
AGCGCTGCGGTCCTGGGCTGTCAGCGCGCGCGGAGGAGCGCCCGATCGGGCGCGGGGGAATGAGAGTGTGGAGGATCATAATAGAGAGATCGCCCCGG
AGACTGCGAGGGGCATGGACGCGGAGGGACGGGGCGGGGGGTCGGCACGCCCGTGCGGCCTGTCGTCATATCCCACGCTGTCACTCGGCTGCGTCCGGC
ACTCCGGGCGGCCGGCGAGCAAAGCCGCCCTGCGCGGCCCGATCGAGGGTGCGGCGGCGCGACGTCGTGGCGTGGGAAATGCGGCGAGGGGCGGGGT
GGGGGACGTCATTGAACCTGCAGCGCGCACGGTGAGTACCGCGGCCCGTGGCGGGGCTGTACTGCGGGACCCCGGAGCGGTAGCGGGGGTTAGACTGG
GTGAGGGTAACCTGCTGCCCCGCGCACCACGTTCTTGGCCAGAGGCCCTGTGGGTGTTGGAGCAGGGTCGAGTATCCCTCCGTGCTTGCCTGTGTGGAGT
GGTTCGCCATGCAGTTGGACTCTTGGCGTCAGCTGGGGCCAGGCGGCGCCTCCGAGGTCGTAAAGGTCAACAGCGCTGTGAGATGGGCGCAGGAAGAGC
GCCAAAGACCTCCTGCGCCCCGATGTCCGCCTCGGGCGGACGTTCGGCCGCCTCGGGACTAGTTAAAGTGCGGCTTGGCTGGGGGCGCGGGACTCGGAA
GTCCGGGTGGAAGATGCGGGGAAGCTGCCCGTCGCCGGTACTGGGGGACTGTAGCTGTGATCCGGGACCACGAGGCATGCACCCGTCGTGGTCCAGCCG
GCCTGGCGCGGGGTCACAGAGCGAAACGGCGGACGCTAGGAACATTCGCTACCACCCCCCGGCGGCACCTTGCAGGGTCTACCCCAGTAAGGACCGGC
CCCAGCAGTGAGTGCGCCCGGGGGCTGGCAGCGGTAGATTGGGTCGCACGAGCTCACGTGGGCCGTTGACCGGAACCGGCAACGCCTGGGACTGGAGC
GCAAACTGGGGTACACTCTTCACGCCCGCTGCTCGTCATCGACCCCGGCGGGCGCCCGGGACCGGACCGGGGGCGACCCACCGGGGGACGGCGCGGCC
CCTGGATGGCCCTGCCCTCGGTCACGAAGCGGCCGAATGGTTTGGGGGGCCTCGGGACGCGGCCGTGGAGGTGCGGGGGGCCTGAAGTCCCTGCCTGGC
CCTCCTCCTAACGGCTTCACGCCCCTCAGGGCAGCTAGGGATTAACGTGGCCGGGTCACACGACCCGCCCAGCGCGGTACGCTGGGCCGCAGTGATTGC
GCGCGCCTGGCGGGTCCTCACCCCCCCACACACATCCGGGTCGGGGCGGAACTGGGTCCCGCCGTTCCCTCAGATCTGCCCCCTGACTTGAACATGCCCA
AAAACCGAAACCGGGCCGGCGCCCAGACCCCCGTGCTAAGGTTCCCACCGCCGACCCTAGCCCCCCCGGAGGTCCCCTGTACTCCGCACGCGACGTCGT
ATCAGGTCCCAGAGCCCGACAATGCATGCGCGTCCCGCTCTGCGCCATGCCCATTGCCCGACCAGCCCCGCCCAGCGCAGAGGCCCCGACTGCTGGCGC
CCTCTGTGGCGCTCGGCCGCTCGAGAGCACCTGCCGCCCGACCGCAGTCTGGCTACCTGCTCCACGGGGCGGCGCTCGTGGTCTCCCGTACTCGGAACGC
CACGCCCCGGGCTCGCGCGTCTCGCGGTCCGACGCATCGTGGTCTTACCTCCGACCCTTTCCTGGGGTCGAGCGCGGGGGGGCAGAGCGCGCTCATCCAG
CCCCGCGCGGCAGCACCCGCCCGCCCAGGGTGGTACCGCTCTCGAGAGTCCGGACGGCCGAACGCCAGGGCAACGGGCCGTGTGAAGACTCACCGTTT
CGGCCGACCGCCATCCTAGCTGGGGCGAGCCCCCGCGCGCGGCCGGATGCGTATGACGTGCGCGCGCTCGGTGCGATGGCGCACGTCGACTGGAATAAC
CCCTTGCCAACCCCAGCGCCGAGAGTCCCTCTTCGGGTCTCCCGTCTGCCTCCGATGGGTGGCCCCTGCCGGTACTTGTTAGGTCCTGGCTCGCCGGGCGC
CTCGTCACCTCGGCAGCCTGGATCGCTTGTCGCCGCCGCAAACGTGCGCGCGTTTCGCCACGGGCCGCCCCGCGAAAAGCGGCGGGAACGTGGACCACG

CGCGCGCCCGCCATCGGCTCGCCGGGATCCCCACCGCGGCAGCGCCCCGCCACAGCTCAGGACGCGGCCGATCGACAAGCACATCTCCCGACTACCGG
GCCCCGCGGCGTGAACGTGGTCATGGAAAACCCGAGCGTGTGCGTTCCTGTCACGGGATCCCGCCGTGGGCAGTTCGCGGCAGGGGCCTACCCGTCGAG
GGGCTACACCGGACCGCCCCTCCAGCTGCATCGTGCAGCCCTAGTGCTGCAGCGAACTTGCCCCGGCCATCCCGTCCGCCCGCAAGCGGCTGGGGACAG
GTTCTCCGCCCATGCGCAGCCAGTGGCCTCATTCATGAGGGCGTGGCGGGCCGGGCCGCGTTGGGCCATCGCCGGTTGGGGGCCCCCACGCCGTCTACCT
GAGTGGACGCGCCGGGCGAGGCTCCCACCACCCCAGTCCTGGGGTCCCAGACGGCCTCCTGGGCGGAGTGTTCTACCCCGCGGCCACCCCCAGCCCGTC
TGCGGCCGAGGCCCCACTGGAGACTGCATTCCCCGCGGCCGGGGAACCGGCCACACCGGCAACGGCGCTCCTGACCCCACCCGCCTGCCACACTCCCCG
CGGCGCGGAGCCCCACCCATACTAAGGCCCCTACGCGCGGCCGACGACATGTTGATGGCTTCGCTGGGGGGGCGGTGACACGTCCACGAGGCGCCTCGC
CGCCGTCGGCCCTGCCCCAGCACGTTCCCCCGTCCCCCGGGGGTGCGGCGTCCTGCTCGCCCCCCGTGTAGGAACGCCGACCGGGCTGCGTCGTCGATGA
GGCAGTGGCCCGCTACGCGGCTCCTCTTCGCACGTGCTTTTGGCTGAAGAACGGGCACCCCCACCATTACGCAGTTGGGACGGCGCGCGGCTGCGAGGG
CCCAAATCTCTCCCATGCGAGTCGTGGTCGCGCCCGTGGACCTCTGAGGGGCAAGGCCGCTCGTCTGCTGAGCGGGGATGTTGCGCCCAGCAAATCGAG
GAAGGTTTGCTCGTAACTTACGGGTACCGAGACTCCTCGCTTGGCTCACCCATTGCGGCAGCCGCGGCCGCCGCCGGGACCAGTAACGGCGTGTCGACC
GCCCCTCGACAACGCCTGGTAGGACGCTCCCCTCCCGGCCCGGACACTAACCCCCGCAACAGCGAATTCCAGGGGCGTCTTCCGCCCAACAAAGACCCA
TCGTGCACGAATTGGGCTGGCGGGCAAGCATGTGCATCACGGACTAGGGTACCGCCTGCCTCCCCGCTGCCTCCGGGGCCCTGGCGCTCCGTCCGATTTT
ATCCCGCTCTGCTGCGATACTCAACCAACCGTTTCGCTTTATTCAGGCCGCCTCTGGGGCCCGGCCGAACACTTTCTCCCGACAAGTGGCGTGACGGACCT
GCCTTCGCGCCGCCCCGAAACAGGCGGGCCGGCCTCGCACCCCTGGGGAGCGGTCCGCCGCAGGCGGGCCTCCTGAGCGCGCGGGTGGCCGGCCAGCG
CGCATACCCCCTCGTCGCTGCCGCCCCCCCCGCCCCGATCCCCGGCCCCGCTCGGCAAGACAGTGGAACCCGCCGCGCCCTTCACTCGCCCGGCTGGCCG
TATTTCGTGGGTCCTCACCTCAGTCGCGTCTGACAGATTAGACCTCGCCGCTGATGCCTAGGGTGGGACACCACCTTCCCGCCCGCCACCACCCTGTGGA
GCGTGAGCGCACTTAAGTCTCCCTACCTCCCCAACTCAGCAGCGGCCCCAGGGCATCCGCGAGGCTCCGTTGACGCACGTGCCGTCGTCCCGCGCCCACT
GTGGCACCTCGGCGGCGACCCTCCGCTGCGGGATGCCTGGAGGAGCGGTCCAGCCTGGGCGCTCTGCCGGTCACCAACGGCGGCCGGGCGGGGGCCCTC
GCGGTCGGACCGAACTTCGGAGGGGTCCCCGGCAGCTGAGTCGAGCGCTGCTAGTGACCTCCACCTCGGCCTAGTCACACTTGCCCGGCTCCCAACCGCT
AAGACATAGGGGGCGTCGCGACGAGGGCAATGCGGGGGCAACCTTGCCACCCCCAAGCGACGTCGCCTCATGTAGGTTGGAGGGGCTGTCTTGTACTCC
ACCGAGGGCGACACGTAGCGTGCGGCGGCTCGACTCGAATCTTGCGCCTCGGTACGCGCCACGCGACCGATGACCTTTGCAGGCGACTCTGCGAGAATG
GGGCCCGCGTCCTGGGGCGTGCTAAATCACGACCGCCGCCGCTGGCCTCCAGGAGGTCCCAGGGGGCAGCACACGCCATTCGCGTCGGTGGTCTAGCCT
CGCGGCACCTCGACGAGTCCCAGGACGCGTCGGGCCCGGTTCTTCCTCTTCCTGTGTGGCAGTCCGGCCGGGTGACGCTTCCGCCGCTGGCTAATTCAGA
CCGCCGTCAGTCGACTGGGCTGCCCTAACCAGCCAGGGCACTCGTGGACTTCCGGCGCCGTCCGACTCCACGCGGCGCGCACAGTCGCCTCCCTCCTTCG
TACCATTCGGCCCTCCTCACCCCAGGGGGGGCTTGCCGCCATTCCCTACGCTGCCATTCGACGTCCCGCGCTACTCACCCATAGTGCCAATTGCGGCGTCG

CGGACACCGGCCTGGCGCAGCGCCAGAGCGCCACCTGTGACGTCGCTCACCGCGGGGGGAGCCCAGCCTCGACGCCCCAGATGGCTCGCGGGGGGGGA
GGTCCCGAGGCCACCCTTCCAGGTGGCACAGATGCCCCTCCCTGGAGGGGGTGGAAAGCGCGCGGCGGGCCCAACACCGTGGGCGGCAGCTGGGCATG
CGCTCGGATAAGAGCGCCAGGATGGCAGGAGGGCCGATCTGGCGGGTGGTGGCCAGTGCGGGGCGGGGGGCGAAACTGTCTGGGGGTACCTGGAGCCC
AGGGTTCAGGGTCCGTGCCATGAGGCTGTCAGGCAAGCCAAGCCGTTAACGGCGGCCAGCGAGTGGGCAGGGAAGGTGACTAGCGCGAGCTACATATT
GCCGGCCGCCGCGCGGGGCGGCCAGCCGTGAGGAGCAATCTGAGGGACGGCCCACCGCCGGAGGGCCTGCACCATGAGGTGAGGCGGCGGGTCCCCG
CCACGCCGCGAGACGGTACGACCCGTTCCGGATGTGCACCACCGATGAAGAAGGCGCGCACATGCCGTTCGCTTTCGGCGTATGGTCTTCGCAGCCTCCG
GTGGGAGGCGCAGCCCGGAGGGTGCCCGCGCCACACGGATCAATGGGTTGGAGGGGCGGACTCGTGGTTTCGACCATTGGTGCAGCTAGAAACCGTCCC
AAGGCTCCACTCGATCGCACTGCCGTCGATCTCGGTTCTGACTCCCACCTATGCCAACACGGGAGTGCGCTATGAAACGCGGTTTTATAGTCGGTAATCG
CGACCGAACGCGGAGCGCCGTCTCGAGGCTTGTAACGCTCCGCGGTCGTTGTGGAGCGGACCGGGTAGTCAGCGTGACGTATTTCTAGCCCCCAGTGTGC
CGCCCGGCGAGAAAGTCCTCGCGGGAGCCCGTCGGCTGCCGCTGCGTACGCGGGGGAAAGCGGCCGAGGTACGTCTTCCTGGTGGCTTGCTGCCGCCAA
CCACCGATCGGCCCGGCTCGATGACAGCCGGCGAGAATGGGTTGGGGCCTACTTGGCCGGGTTGGCTGATCGTACGCAAGCAGCGTGCTGCGGGCCCAC
GCAAAGGGGAATGGCGGGGGAGCGCCGGCTGCCAATTCCCCCCGGCTGCACAGGCATGATCCGGTGTCAAGAACGGTCTCCGGCCCACGCGGCCGCGC
CTTGGCCCGAGTACGGCTGACTCAGAAGCCGTTGGGTGCGAGGGCGTGGGGTGGTGGCGCATAGGGAGCCAGACCCGACGGAGCCAGGATGGTTCGAG
CAGTGTCACGGCCCGGGGTCATGTGGGGCCCGCCCGCACGGATGACCCGCGTCTCGATAATCGGTTGCAGGCGTGCCGTTCCCACGGGGCGCGCCGGTT
ATCGAACGGGTGCGCGCGTAGACTCGCTTCCGGGCCCCGGACCTGGGCCAGGCAGAAGCGGGGATTACCCGTCTTGATCGCAGACAGCCCGGTGCCCCC
GGGGGCCGACGTACAGCTGGCAAGCACCGGTCCGGCGATTACCCGACCTGGGTTGGCAGGTACACGTTACAGGGGTGGCGTGCCCCCCGGCGCTTGGCG
CCGGCTTGGAGGGACGGGGGGGGATCTAGCTCAGATGGCCAGCGAGCGTAGCTTCGACGGTGGCAAGAGCGAGCGGCGGCGTGCTTGGGGAGGCCGCG
GGCGGGTGCGACGTCCAGTTCGTCACACGCATGAGGGGTTTCCGGGGAGGAATCACCGCCTCGCGCAGGCGTGTCCGACACGACCCGTGTAGCGGGTGT
GAGACATTTAGGCGGAGGCCAGAACCCCTTGCCGGAGGGCTTGTAGTATATTGTCAATGGGGCCGACACCACGTGCGGATCGGGACTGAGAGCGCGTAG
AGCCTTGGCCCTTGCTATATTTGTGGAGCGGACACTGTGCTTCCCACACGTCACGCTGGCGTAAGCCGATGCCCGTCACCGCCCGCGCCCCAGGCCGAGT
GCGGGGGGCCGCAAGGTCCTGAAGCCGATCTACGATGGGCTGGGTGGGCGACGTCGTGGGCTAAGCTCGGCGGCCCCACCGTGAGGCGGACGTGGGGG
CCTGTACTAGAGATGGAGCGACGGTCGTCGATACAGTTCTTGCCAGGAGACCGAGCATTTTGCTAGGCACGGGCTGCATGGGCAATTCATTAACGGCGG
CGGGCCCACCGCGGCCAGCGGTGGCGGGGCGTAGTACCGGTCGGCAGGGATACGACCCCGGGCCGAAATTCGGGGCCGGCGGCCAGGGGGCGGCGCG
CCTGCGCGCCGTGACGCTGACGCCCTCCCGGGGTGCCGCGGGCTGGCCTGAGGCCCAAGGAGGCGCCTGGGGGTCGAGCGCGGGCCCGGGCGCACGCT
GATCCCGAGCCGACGAGCTTACGGATTGACCAAGAGCACCGCGAGCGTCCGAGCGCTGCGGGCGTGGTAACAACTACATCCCGGGTCGAGTTTGCGGCT
GGGACGTGGGATTGGCCGTGCAGACGCGCTGGGGGGGGAGCGGGGCTGGCCCCTCGCTCCGTATTCCCCACTCCACATGGTCCCCGGGCGTACGGGCGT

CCCCGACGGTTCGGGACGCGGGAATCGCGCAGGTGCCCCTCGGTGCGTGGCGGGCTGCGTGTAAGGCGCTTTCGCTGCATGCGGCCGTCTCGCGGACAT

GGCGTACGGGGGCGACCAGGGCGAATCCAAGGTGCGCCCAACGGGCCGAGGCGCTACAGATGGGGAGCCCCGGGCGGCCGTACGCAGGAGTCCAGTG

GCGTATGGAGGGAGGCCTGGGGCTCCGCAACGGCCGAGGCTATGCCCAGACCCCATGCCATTGTAGGCCGAACGCGATAGGTCACGCCGGTGATCCTGC

CGGGGTCGCGGGCCGGATCCACAAGGCCAGACCCATAGCGTAGTCTATAGGCGTCCGGGAAGGGTCGTGGGGGGGCTAA

>2017.TE.25009.1.2

CACGCCCCCCTCCCGGCCAGCCCGCCCCCCGCCCCAGCGTCGTACCCGGCGTCGCGACTCCCCGCCGTCGAGAGGCCTTTGCCGGGGCCCCGGCTAGCC

CGGGGCGAGCGGACCGCCGCGCGCGGGGGGTGTCACCCGGGGCACACCGCGCCAGCCTCCCGGGCATGCCACGCCGGGTTCCGCCCCCGCTGCTCCAA

TTTGGGGGGCCCCTAAGATCCCGTTCCCCAGGCCAGCCGCGCCCGCGGGCCAACATTGCCACACCGTGTCGCCCCCCCGCCTGCCGCACTGACCGTCTTG

CGGGCCCTGCTCCCTGGCCCGAGCACACCTCCGGGGACCCCCCTCGCTCGGCCGGGGGAGCCTTAAGCGCGGCACCGAGTAGGGGGTCGCGTGTTGCCG

CCGCGCTGACGCCCCGGCTGGGACCCCTTTCAGGTCGAGACGCCGGGGTGGCCATGCGGGTCCAGTCGCACGATGGCGCGGCGATGTGGCGCCGACTGC

CCCGCCTGTCTTCTCCGTGCCAGCCCCTCGCCACCATGCCGGTCCGGCCCGCACGGGGGTAGGAGGTGCTGCCGCGGCGGGAGGCGTAGGACGCCATAT

CCGAACCGGCGGGTCCCCCCGGCCACCGAGTCGACAGGACCATAGGCACCGCGTCCGGGCGGGCGCCCTTAACGCCGCCCCTTACTGGAGAAGCAGAG

CTCTGCCGAGGCACCCTGCCTAGCTGGCTGCGTCCCGCACCCCGCGATCCCCCTGGTCCACTTACCCAGCGGGATGCGGGGATGTTCGCAGCTATCACAC

GGTCGTGGCCTGCCTCCCTGGGCCGGCGAGAGCACCGCCACCTTCGTGTTCCTTGCCGCCGGGCGACGTGTCCGGGAAAGGCTTCGCCGCGCGCCCGCGC

GGAGCGCCGGGGAGTCCCGTGAGCGGCCGCAGGAACGTCACGGACCCCGGCTTGGTGCGGTCCCCCTCAGTGGTGCCCGACCAGAGACCGCGGAAAGA

CTCCAGGAAACGCCTCGGGGGGAAAGCAGGGCCGCGCACTTCCGCTCCACGCTAGCTGGGGGGACCACGACGGCAATACTGAACACCTGGCTCTTCGG

CAGCCCGGCCTATGAGGGGCCCTCTCGTCCCGGCCAAAGGCTATTTGCCTTCCTGTCCGGGCACCCTCGACCGCACCCGGGCGGGCGCAGGCTTCATTGC

GGCGCGACTCGCCAAAAGTCCCCGCGATCTCAGCGAGTACCCTGAGCGCTGGCCCTAGTCCGCCTGCCCCCTTAATGTCGCTCAGGTCTGCAATCTCTTA

CGACCGGCTCAGGTAGTGGCCGCACGGTGCGTAGGTCCGCTGCGGGCCGGGCCACTCGCGCCGATCGTGCCGTGCGGCGGCCCTGACACCGTATGCGTG

CGCGCTCCGCAGGCTCGCGAAAATCCTTCGCAGGCACTTGGCCCCGTTGCTAGACGTGGTGTCTCCTAACTGTGGCTCACGCACGGCGTTTAGGCCACGG

TAGCCCGCTGTTCGGTCCTTACGACTCGGCACACGCCCCCGTGCACGCCCTCGGGTTCCGCAGGCCAACGCCTGGCCCTAGGGTTGCGTGCCGGCCTGCC

CGCGATGCCACTTGCCCCACGTCCCCCACCTGGCCGGCGCAGACGGGTTCAGTGGGTGTCGAACGCCATTCAGTCCCCTGGGACTCCCCCGACCCCTCCC

ATGGCACCCGCCGCCTGGCCTTGGAGCTCCTGCGCGTTGGACCTTACTGCGGTCGTCAGCTTCTCGGCGCGCCCCGGACTAGTCCTGGGCGGCCAGCTCG

CGGGACCCTAGCGCGCACGCGTGCCCGATTGCCGTATCCCCTCGACGGCGCCCGGGCATAGGGTTCGATCCCGCTGCTATTGCGGGTGCGTCCAGACCTG

CCGCCGTTTGGCGATCCGAGTGTGCGAAGGGAGCCAGCCAGCGGGCCGCCTGGCCGCACTCCAGGATCCTGGCGCCAAGGGAGAGCCCTGCCAGCGCT
ACGCGAATACTCGACCTGTCCGCGCCCCGCCTCGGGCTTGCGAGATGGCCACTAGACGCTATCGGGTCGTCACCCCGTCGGCCCCACAGGGGCCCGGCTT
CGCGTGGCTTTCTTGTCGAAGGCCCCGAAATCCGGCAGGCCCCGACACCTTCACCGTGAGCGAGGACGCGCGGTGCGGGATGTCCAGTTCATGCACGCC
CCTCCACGGCCGCCTTCGCGCGGACAGCGTCGGGCGTGCCCAGGACGAGAACAGACCCTGGGCCAGCCTCAGGGCCCGGCCTAGGCGACCCTGGTGAC
CAGTTTCCACTCGGCACCCTCGGGCTTCGTGGCAGTGCAGTCATGCCCCCGGGTGCTCCCGCGCGACCGCGCACCTCCCACCCAAGGTTCTTGGTCAACG
CGCCGGATCTGACGTCGCGGTCCGGCCCGCGCCGTAGGCAAAAATGGGTTCGGCTCCCGCCACTCCGCCAGTTCGAGGGCGGTAAAGATCTCAGCCGCC
GTGTTTCGGGCTATCTCCCCGCGATCCCGGGCCACAATCGCAGGTGGCTGGGGCGTCGGCCAGACCCGTCTCGTCGGCCTTTCTGCGCACATTACCTCCGA
GCAAGGCAACCGCTCCGCCCCATTACAGGGGGTTGTCTGGTACCTCCGTCACGTACCCCCGCATCGCTGGCGGGCTGACCCAAGCCCCCTCCCATCCCGA
CCCTCACGCGCTCACGCCCCTTGCCGGGCGGGGTCGTGGCTGCCCAGGGTCCAGGGCCCGCCCCGGGGGCCGGTTGGTTCCAGGAACTGAGGGGGGGTC
GCCCAGCTGACCGCTCCTCTCGCGGGGTCTATTCGGCGTGGCCATCCGCAGGTGCGACAATGCCGGCTCCCATTGCCGGGCCGACCCTGGTCCCTGGCGC
TTCGGACTCCCGGGGAGCGCCTCCCTTCATCGGCGGCCCCTGCCCCCGTCATGACCCCCCTTACGCGCCGGTGGTAGCCGCGCGTGGTGGCGTGCCCCTCT
CCATCATTACGCTCCACAGGAGTGGCCACCGGTATGGGCGCGGTGCAGTCGTACATGTGTGCGGCCCTGTTTTGCGAACCCTTCCCGCGGTGACCTCCGC
CCCAGCGGCCTTCTTCGACCGTTAACGCCGGCCAGCACCCGAAGGGGGTCACACAAAGCCCTGCAAGGATGCGACGCTGCGTAGCCGCAGGTTAGCCGC
TTCCTTCCCAAAACAGTGTCAGAGCTGTCCGGCGGAACGTTAACGCCGCCGGGAGGATCTGCGGCTACCCTCAGGCCCGTCCCGTGGGAGTGGCCGGCG
TCGATGGGGGTACGCACCCCACTAGACGCGGATTTTGGCCAACGGGCACGGCCGCTTCCGAGTTCCCCTGCGGTCTGAGTGTTGGTACGTTCAGCCGGCC
GTTCCGTGGGGCAGAGCGCCGCACGCATGTCGCAGGAGCGCGCCCTCCGCGTCTTGCGGGCGGGGTCCTAACGAAGCCCCCTGGGCTTGGAGGTAGGGG
GCGCGGGGGAGCGATGGGAGCGCAAGCGTGCCATCGGGTCCGGCCCATAAGTGTGTATGGCGCACCGCGCTGCCACGATGTCCGGTGCGCGGGCGCCG
CTGGCTAGCTCACTCTCCCCCGACTCCGCCGCCGCGCCCCGAGACGTATGGAACAGCTGGCGCGCGTCCCCCGCCGCGGGCGGCAACGAACTAGATCCA
CCGCCCTCACGAGGAAGCGCGCCGCCACCACGTCTCCCCGGCCGACGCGGTCCCACCATCCGCTGTGCCGGCGCGCGAGCAGCCTGGCCCTCGGGGCGG
TCGCGCCAGCGCAGACACCCTGTCACGCGCACGGGAGCCCTGGCCACCGCGTCGACGCCCGGGCTAGCCCCGACTATCAGTACTGACCAGAGGGGCGC
CGGTTGTCCCGACTGGTTCCTCCCGAAACGCTGGCGGTGGGCCCGCACGATTGGTGCAGGCCCCCAAGAGGATACGGCTATTCGCGGCTGCCCGAGGCG
AGTCGGGCCAGACCGGTCGCGCGTGTGTGTGTTAGCCCTCCGTCGCGGCCAAGCCGGCGGCACCGGACTGGGCGACAGTAGCGTTCCTCTGTCACTAGTC
ACTCTAACGGACAAATGGAAAAATGGCACCTATCGCTACCGACGGGGTTTTCCTCCAGGATAAGCCTCCGCGCCCCCCACTGGACGCCAGCCATCCTTCC
CCGAACGGCTCGCCTGTGCCGGCTCCAACCCCGCGGGACCTCCCTTCGCGGCCCCGGCGGGGATCGGTGGATCGTCGCCGCCGGCGCCTACCGATCGCG
CCGCCACGGTCGTGCACCAACCAAGCACTGCTACACGCCTGTGCGGCCGAGGCCGCCCCATCCAGCAACGGGGCACCGTTGACCCGTCCCCCATGATGC
CGGACATGGTAGCCCCCACACATGCATCTTCGGGTAACCCGCGCCAGGTCCCCCCCCGCGCCGCCGCCGGCTTTCCCCCCGCTGGTGCTAAGCGGCAGAT

GCACCACTGACGGGACACACTCGCCCCCGATCCAGTGCGCTGCCGCCTGCGGCCCCCCACCGGACTGGTGCCTGGCGCTACACGGAGCCGGCCGCGCAC
TGTGACGTATGGATGCCCGAGGGGACATCCGCCCCGGACGCGTGCGCAAAAGACCACTCGCTCGACGCCACGACGTGTGCGGCCGGGGTGATGAACGT
CGGCCGGTGTGCCAGTGAGGCTTGCACCTACACTCCCTGGTCCACCGGGCCGTTCGAGCGGCACCGGGCGAAGAACCCGCAGCCTCCCGGCTTACGCAC
CGGCCTCGCGTCAACAGGCAACCACGACCGCAGGGCCGGCAGTGTGTGTCGCTGTGGAAAGGTGCGCCGCAGGCGACCCCCACGTCAGCCCACTACAA
AGCGTGGCAGCATGCTGCGCAGCGACCGCACGCCCCGACACAGCCCAATGCACAGCATCGGGGGGGCGGAATCGCCCCACGCCCGTAACATCCCGCGG
GTCGACCGCCTTACACGTCGACTGCCACACCCTGGTGCCGCAGTGGTATCCCGAGACCGCATGACCCGCCGCGAGCCCGCTGACCACCTGTCCTTGATCG
CAGTCGCCACGGGGCCGGAAGGTGTACCGCATACCGCACGTGCTGCGCGTGTGCAGCACGGGGGCACCCTGCATGTATGGATCCCTCTAAGCCGACCTG
CCACCCCAGGCCAGAGGCTAGAGTCTGCCGCCTAGGCCCCCCCCCGTGGTCGCCAGCCCCGGTGGCGGCCCACCCTCGGGGCGACTGACCGGCCACCAT
CCAGGGTAGAGAGGTTTGGCCCTAAATACGGGCGTGCCGAGGAGGTGTTTGGGGCGAGCGGCAGCAGGTTCGGCGGATCCTTGAGGGTGGGGCCTGGG
GTGGGACGGGGGCGGCCGAAACGCGAGCATAGGCCCCTGCCTGGCCCCTTTGCAGTCTGTACTTTTATTGACATGATACTAATACCGTGTTCCGCCCTGA
GATCGCACGCGGACCTACCGTGCCTCTCTCTGCATAGTGCTTCAAGAGCCCAGCGCGACTGGGGACCGGCTCCCGCAGCCGGCTCGGGGCGTAACGCTC
GGTGGAAGGGTCACCCGGATAGCTCCCGGTTAGTTTCGTACAGGGTGTCGGGGGCGCCCCCAGCGAGTCTCCTGGAGGTGCAGGGACGGCGGTGGTCCT
CGAGGGGGCGGACGTTGGCCGCATAACGGGTAGTCTCCAGGGTCACCTATGGTCTGGCTCGCCGTCAGGGTCTGTGAGCGTCGTGCTCACGCCTCCGCAA
CAGCCGGCGACCCCGGCTCGCAACGGCCAGCCGTGGGCGGGGGGGCGACCGGACGCAGTTCCGAGTCTAGGCATGGTGCCACCGCCCTCTCGTTGGGTT
AGTCTCGAATGGCTGTTTCGGGGGTCAGCGCGGCGGGGCCGCCGCTGGGAGCCCGCATAACCGTGGATACACTTGCTGGGTGCGCGCTCGAGGGCGGCC
CCCGAGGGCTGGAAAACCATTTCGGGATCGCCCCCGCGCTCCCGTCCGCACTGCGGGGTCCAGCCCGACGGGGGCGGGTTTCGTTGGGGCAGTACTCAC
GCCCACGGCGAGGCCCATCCAGAGAGTGAGGGTGTCGCTGGGGGGGCCTACCGGGCGGAACGCGCCCCGACTTAGGGCGCCGCAATGCACGACCAGTC
ACCGTCTGGGCGTCGCCGCGCCGTTGAGAACCCCAACACAGAAAGAGCTTGCCGCCTCTGGGGGAGCCAGGCCGAGAAGCGTGGGCGAGCGGCCACGC
TGCGGGCCCTCCGCGGACCGTGCGGCGCTCTCGGGTGACAAACCTAGTCCCCGTCCCAGGCCATGGGCGAGCAGCTGCGCCGGGACTGTGCGACGCCTA
TCCGGCACATTCGGTATTATTGGCCTGGGACGACAGCCGGCGACAGGGCGGACGAGGCGGCGTGGACTCGGGGTGCAGGGACGACGTCCACCCGTACT
GGTCAGTAGCCCAACGCGGAAGCCTGTCTGGGGCTTTGGCCCCTAGACATCGGTCCGGGGCGGGGCGCGTCATCGCTACGTTAGTGAGGGGGAGTCTGC
ACCGATAGGGTGTGGTGGTGTTGCTTAGATCGTTGACGGGCCGTGAGGTGGCGCTGCTGTTTTCCGGCTTACGGCTTACGACCTACCGCCCGCCGCGCCGG
CCCGAAGGCGGCGCCCCCGCACATCGCCCTTGGGTGGTAAAACCCGCCCTGCCGCCGCTACCCCCCCCCAGGGGTGGCCGGAACGGCGAGGGCAGGA
CACGGTCGACGCACGCAGCGCCGTGACGCCCGGCCCGCGCTAGGGTTGGCACCCGTGGGACCTCGACAAACTTTCCGTACGATTGGGGGGCGCATACAG
CGGACGGGGAACCCGCCCGACGCACGCGCGGTCCCGAGGGGTGGGGGACCTGCAGCTGCGTCGCGCGGTCCGCGAGGGCGTCTTGCTAGACCATGGGC
GTTGCAATAGTGGGAAGGTGCCTCCCAGGCTGCGGGGCGCCGGACGCCCGACACCAAAGCTGTGCGAGGACAGAGAGGCTAGGCACGGATGGGTACAA

GCGAGCTGACAGCCTCCATCCTTGAGTGAGCACGCCGCGGCGAAATGCCGGCCGAAATGGGAGGGGCGCGACACCGGATCGTGATCGAGCGCGTGCGC
TTCGCCCACACGCCCTGACCCGCTGAGTGTCTCGGGTTTGAACCCCGTGCCCGGGGGCGCAGTGGGATAGGCACGACGGGCAGAGTTAGGGGCCTGGGC
ATAACGGCCCTGTCCGCGGCCGAGCGGGCCCGGGTCTTCTCGACCAGGGTGCCGGCCCCGGCGGGCCCCCCGCCCACGGACGGGGACCTGGAGTTGAGC
CAGGCCACAGGCGTGACCGGAATTGGAGCGCCAGGGGACAGCGGCCGCCCGCGCCAGGCCACGCCGATGGGGGCGTTCTGCCCAGGCGTCGCGCAATG
GGCGGACCGGTCTACGCGACACCGCCCGCCCGGCCTGGAACTCCCCCCGAGCACATTCGATATGGTCCGCATCAAGGCGGGAGGCATCGCCGGGGGCC
GCGCGGACCACCACCTTCGCGGGGGACGGTGCGGGTCGCGACGAGGCGGCGGCATGCGCGCGGGTGCCGTGAGCCCTAGAGTCAGCGGTTGCTGGTAT
GGGCGGACGGGCGCCCCCGACCTGCCGTGGACGCAGGCTCGCGGACTAGTCGACCCGCCATGGGTGCTGTGCGTGACGTTGGCCTTGGTCCAGGGCGCG
ACGCGCAATACCGCGCGGGCTGAGTATCGCGTCCACGGACAGGCGGCTGAGCCGCCCGGCGTAGCTGGTACACGGTCGGGCCGAAGTGGCAAAGAGCC
GGCTTGCGCCCCCAGCGCCGTTGCAGCAGGTCTGGACAGGCAGTACGGAGCGAACGCGGCGAGACACCCGGCTCCGGGCGTAGTCGGATCCGCCCGCC
AGCGGGCGCGCAGCGGTGTCGCGGGCTTGATGCGCCGGTCAGGGAGGTCGTCGCGAATGAGCCGGGTGGAGGGTGCAAGAAGCCAGACGATGGGAGC
ACCGAGTTGTCCGCGTGCGATTGCCGTCCCGGCGAAAGCGCCAGGCCGCGGCGCGTGTGGCAACGGCAAGTACCCGGAAACACGGCACGGGTCTCGTG
GCGCCGCGAGGCGGCGGGTGGCGCGCTCCGGGGGGCTGCGCAGCCCCTCAGCGCCGCTGCTAGTGCCAGACGCTTGCCCTTCTTGAGCGCGGCGGCGGG
AACTCGGTCGCGTGCGTTCAGGGCCCTCATCCGCAGACGTGAGGGGTGCGGGCCAGGGGGCGCAGAGGCGCCTTCCGAAACCCGTAAGGGATGTTCTGG
CGTTGGGGGGCCGCCGTCTTAGGGGGGTAGCGCCGCCTCAACTCCAGTATCGCCGCAAGGCCGGTGCCAGGTTCGATCCGGGGGCGGCGGCGTACCGCG
TCCACGCGTCTCCCAGAGGAGGGTCAAGGTGCATACGGGAGGGATCATACGGCAATCGGCGGCCGGGTTCCGCCATCTAACGTGGCGATCGCAGCGTAC
CAGAGCGAGGCCTATGGGCCATATCCCTGGCGGGAACGGGCTGCTTGCGCCCCGTCCTCGCCGACCCTCCGTACCGGTACGGGGCGAGGATCCCGACAC
ACAGCACGGGGGCGGCGCGCAGCACCCACCCCGTCCTCGGCCCAACAGACAAAGACCCCGCGGCGGGCTGGGGCACCCCCTTTCCGGCTACGTCCACC
GATTCTGTACAGGACAGCCGTTGCGATGGGCACCGGCGATCGGAGAGAAGGACGCCACAGATCAGCAGACGACTCGGGGAAACCACGGGACCGCGCG
TCGCGTCAACTGCCGCGGGAGTGAGAAGGGAGCGACAGGAGCGCGGAGGTAAGGCCAATGGGGCCAGTTGAAGCCGCCCACAGAGGCTGTGCGGGGC
CCCGCCGGGTTTAGGAGCTGTTCTTGGAGCCTGTCGTTAGGGGCGGGGGGGGGCTTACACGGCGCTGGGGGACCCACGAGGCGCAATCCGCCGCGGACT
GCTGGAGGGCTACCTGGGGCAGCACCCCAGGGGCGGCCATGGCGCTTGTTGACGGGGGCGCTAGGACCTTGTCCTAGGGCGCGCCGCTTTCGCCCACAG
GGCTTCGGTGGGCAGTCTATCTGTGCGGTTTCAGTCTCGTACGCACGTGATGCGGTGTAGCCTGAGCCCGGCAGTTGTCTAGAGTAACTAGCGCGCGGTTA
TGGCTTCGTCTGGAAACGGGCTGCAATGTTCCGGGTGTTCATCACACCACGTTCCGCTTTGCAGCTCACCCCCCGACCACACCCGGATTCGGACCGGTGGT
CCTTCCTCTGAGGTACTCGCCGCTCATACAACCGCGGTTCCCGCTGTGGTCGGCACGGCCCGGGTTGCAGGGGCGTGCCTTCCCGGGTAGTGTGCCTGCGC
GGCGTGCGGCTTCCGCAGTCCACGGACCGTTCCGGGGGTGAACGATGGAGGTGAGGGGACGAGTCCCTCCGAGATTAATGCCCCGCGGCAAGCCCGATC
AGCGCTGCGGTCCTGGGCTGTCAGCGCGCGCGGAGGAGCGCCCGATCGGGCGCGGGGGAATGAGAGTGTGGAGGATCATAATAGAGAGATCGCCCCGG

AGACTGCGAGGGGCATGGACGCGGAGGGACGGGGCGGGGGGTCGGCACGCCCGTGCGGCCTGTCGTCATATCCCACGCTGTCACTCGGCTGCGTCCGGC
ACTCCGGGCGGCCGGCGAGCAAAGCCGCCCTGCGCGGCCCGATCGAGGGTGCGGCGGCGCGACGTCGTGGCGTGGGAAATGCGGCGAGGGGCGGGGT
GGGGGACGTCATTGAACCTGCAGCGCGCACGGTGAGTACCGCGGCCCGTGGCGGGGCTGTACTGCGGGACCCCGGAGCGGTAGCGGGGGTTAGACTGG
GTGAGGGTAACCTGCTGCCCCGCGCACCACGTTCTTGGCCAGAGGCCCTGTGGGTGTTGGAGCAGGGTCGAGTATCCCTCCGTGCTTGCCTGTGTGGAGT
GGTTCGCCATGCAGTTGGACTCTTGGCGTCAGCTGGGGCCAGGCGGCGCCTCCGAGGTCGTAAAGGTCAACAGCGCTGTGAGATGGGCGCAGGAAGAGC
GCCAAAGACCTCCTGCGCCCCGATGTCCGCCTCGGGCGGACGTTCGGCCGCCTCGGGACTAGTTAAAGTGCGGCTTGGCTGGGGGCGCGGGACTCGGAA
GTCCGGGTGGAAGATGCGGGGAAGCTGCCCGTCGCCGGTACTGGGGGACTGTAGCTGTGATCCGGGACCACGAGGCATGCACCCGTCGTGGTCCAGCCG
GCCTGGCGCGGGGTCACAGAGCGAAACGGCGGACGCTAGGAACATTCGCTACCACCCCCCGGCGGCACCTTGCAGGGTCTACCCCAGTAAGGACCGGC
CCCAGCAGTGAGTGCGCCCGGGGGCTGGCAGCGGTAGATTGGGTCGCACGAGCTCACGTGGGCCGTTGACCGGAACCGGCAACGCCTGGGACTGGAGC
GCAAACTGGGGTACACTCTTCACGCCCGCTGCTCGTCATCGACCCCGGCGGGCGCCCGGGACCGGACCGGGGGCGACCCACCGGGGGACGGCGCGGCC
CCTGGATGGCCCTGCCCTCGGTCACGAAGCGGCCGAATGGTTTGGGGGGCCTCGGGACGCGGCCGTGGAGGTGCGGGGGGCCTGAAGTCCCTGCCTGGC
CCTCCTCCTAACGGCTTCACGCCCCTCAGGGCAGCTAGGGATTAACGTGGCCGGGTCACACGACCCGCCCAGCGCGGTACGCTGGGCCGCAGTGATTGC
GCGCGCCTGGCGGGTCCTCACCCCCCCACACACATCCGGGTCGGGGCGGAACTGGGTCCCGCCGTTCCCTCAGATCTGCCCCCTGACTTGAACATGCCCA
AAAACCGAAACCGGGCCGGCGCCCAGACCCCCGTGCTAAGGTTCCCACCGCCGACCCTAGCCCCCCCGGAGGTCCCCTGTACTCCGCACGCGACGTCGT
ATCAGGTCCCAGAGCCCGACAATGCATGCGCGTCCCGCTCTGCGCCATGCCCATTGCCCGACCAGCCCCGCCCAGCGCAGAGGCCCCGACTGCTGGCGC
CCTCTGTGGCGCTCGGCCGCTCGAGAGCACCTGCCGCCCGACCGCAGTCTGGCTACCTGCTCCACGGGGCGGCGCTCGTGGTCTCCCGTACTCGGAACGC
CACGCCCCGGGCTCGCGCGTCTCGCGGTCCGACGCATCGTGGTCTTACCTCCGACCCTTTCCTGGGGTCGAGCGCGGGGGGGCAGAGCGCGCTCATCCAG
CCCCGCGCGGCAGCACCCGCCCGCCCAGGGTGGTACCGCTCTCGAGAGTCCGGACGGCCGAACGCCAGGGCAACGGGCCGTGTGAAGACTCACCGTTT
CGGCCGACCGCCATCCTAGCTGGGGCGAGCCCCCGCGCGCGGCCGGATGCGTATGACGTGCGCGCGCTCGGTGCGATGGCGCACGTCGACTGGAATAAC
CCCTTGCCAACCCCAGCGCCGAGAGTCCCTCTTCGGGTCTCCCGTCTGCCTCCGATGGGTGGCCCCTGCCGGTACTTGTTAGGTCCTGGCTCGCCGGGCGC
CTCGTCACCTCGGCAGCCTGGATCGCTTGTCGCCGCCGCAAACGTGCGCGCGTTTCGCCACGGGCCGCCCCGCGAAAAGCGGCGGGAACGTGGACCACG
CGCGCGCCCGCCATCGGCTCGCCGGGATCCCCACCGCGGCAGCGCCCCGCCACAGCTCAGGACGCGGCCGATCGACAAGCACATCTCCCGACTACCGG
GCCCCGCGGCGTGAACGTGGTCATGGAAAACCCGAGCGTGTGCGTTCCTGTCACGGGATCCCGCCGTGGGCAGTTCGCGGCAGGGGCCTACCCGTCGAG
GGGCTACACCGGACCGCCCCTCCAGCTGCATCGTGCAGCCCTAGTGCTGCAGCGAACTTGCCCCGGCCATCCCGTCCGCCCGCAAGCGGCTGGGGACAG
GTTCTCCGCCCATGCGCAGCCAGTGGCCTCATTCATGAGGGCGTGGCGGGCCGGGCCGCGTTGGGCCATCGCCGGTTGGGGGCCCCCACGCCGTCTACCT
GAGTGGACGCGCCGGGCGAGGCTCCCACCACCCCAGTCCTGGGGTCCCAGACGGCCTCCTGGGCGGAGTGTTCTACCCCGCGGCCACCCCCAGCCCGTC

TGCGGCCGAGGCCCCACTGGAGACTGCATTCCCCGCGGCCGGGGAACCGGCCACACCGGCAACGGCGCTCCTGACCCCACCCGCCTGCCACACTCCCCG
CGGCGCGGAGCCCCACCCATACTAAGGCCCCTACGCGCGGCCGACGACATGTTGATGGCTTCGCTGGGGGGGCGGTGACACGTCCACGAGGCGCCTCGC
CGCCGTCGGCCCTGCCCCAGCACGTTCCCCCGTCCCCCGGGGGTGCGGCGTCCTGCTCGCCCCCCGTGTAGGAACGCCGACCGGGCTGCGTCGTCGATGA
GGCAGTGGCCCGCTACGCGGCTCCTCTTCGCACGTGCTTTTGGCTGAAGAACGGGCACCCCCACCATTACGCAGTTGGGACGGCGCGCGGCTGCGAGGG
CCCAAATCTCTCCCATGCGAGTCGTGGTCGCGCCCGTGGACCTCTGAGGGGCAAGGCCGCTCGTCTGCTGAGCGGGGATGTTGCGCCCAGCAAATCGAG
GAAGGTTTGCTCGTAACTTACGGGTACCGAGACTCCTCGCTTGGCTCACCCATTGCGGCAGCCGCGGCCGCCGCCGGGACCAGTAACGGCGTGTCGACC
GCCCCTCGACAACGCCTGGTAGGACGCTCCCCTCCCGGCCCGGACACTAACCCCCGCAACAGCGAATTCCAGGGGCGTCTTCCGCCCAACAAAGACCCA
TCGTGCACGAATTGGGCTGGCGGGCAAGCATGTGCATCACGGACTAGGGTACCGCCTGCCTCCCCGCTGCCTCCGGGGCCCTGGCGCTCCGTCCGATTTT
ATCCCGCTCTGCTGCGATACTCAACCAACCGTTTCGCTTTATTCAGGCCGCCTCTGGGGCCCGGCCGAACACTTTCTCCCGACAAGTGGCGTGACGGACCT
GCCTTCGCGCCGCCCCGAAACAGGCGGGCCGGCCTCGCACCCCTGGGGAGCGGTCCGCCGCAGGCGGGCCTCCTGAGCGCGCGGGTGGCCGGCCAGCG
CGCATACCCCCTCGTCGCTGCCGCCCCCCCCGCCCCGATCCCCGGCCCCGCTCGGCAAGACAGTGGAACCCGCCGCGCCCTTCACTCGCCCGGCTGGCCG
TATTTCGTGGGTCCTCACCTCAGTCGCGTCTGACAGATTAGACCTCGCCGCTGATGCCTAGGGTGGGACACCACCTTCCCGCCCGCCACCACCCTGTGGA
GCGTGAGCGCACTTAAGTCTCCCTACCTCCCCAACTCAGCAGCGGCCCCAGGGCATCCGCGAGGCTCCGTTGACGCACGTGCCGTCGTCCCGCGCCCACT
GTGGCACCTCGGCGGCGACCCTCCGCTGCGGGATGCCTGGAGGAGCGGTCCAGCCTGGGCGCTCTGCCGGTCACCAACGGCGGCCGGGCGGGGGCCCTC
GCGGTCGGACCGAACTTCGGAGGGGTCCCCGGCAGCTGAGTCGAGCGCTGCTAGTGACCTCCACCTCGGCCTAGTCACACTTGCCCGGCTCCCAACCGCT
AAGACATAGGGGGCGTCGCGACGAGGGCAATGCGGGGGCAACCTTGCCACCCCCAAGCGACGTCGCCTCATGTAGGTTGGAGGGGCTGTCTTGTACTCC
ACCGAGGGCGACACGTAGCGTGCGGCGGCTCGACTCGAATCTTGCGCCTCGGTACGCGCCACGCGACCGATGACCTTTGCAGGCGACTCTGCGAGAATG
GGGCCCGCGTCCTGGGGCGTGCTAAATCACGACCGCCGCCGCTGGCCTCCAGGAGGTCCCAGGGGGCAGCACACGCCATTCGCGTCGGTGGTCTAGCCT
CGCGGCACCTCGACGAGTCCCAGGACGCGTCGGGCCCGGTTCTTCCTCTTCCTGTGTGGCAGTCCGGCCGGGTGACGCTTCCGCCGCTGGCTAATTCAGA
CCGCCGTCAGTCGACTGGGCTGCCCTAACCAGCCAGGGCACTCGTGGACTTCCGGCGCCGTCCGACTCCACGCGGCGCGCACAGTCGCCTCCCTCCTTCG
TACCATTCGGCCCTCCTCACCCCAGGGGGGGCTTGCCGCCATTCCCTACGCTGCCATTCGACGTCCCGCGCTACTCACCCATAGTGCCAATTGCGGCGTCG
CGGACACCGGCCTGGCGCAGCGCCAGAGCGCCACCTGTGACGTCGCTCACCGCGGGGGGAGCCCAGCCTCGACGCCCCAGATGGCTCGCGGGGGGGA
GGTCCCGAGGCCACCCTTCCAGGTGGCACAGATGCCCCTCCCTGGAGGGGGTGGAAAGCGCGCGGCGGGCCCAACACCGTGGGCGGCAGCTGGGCATG
CGCTCGGATAAGAGCGCCAGGATGGCAGGAGGGCCGATCTGGCGGGTGGTGGCCAGTGCGGGGCGGGGGGCGAAACTGTCTGGGGGTACCTGGAGCCC
AGGGTTCAGGGTCCGTGCCATGAGGCTGTCAGGCAAGCCAAGCCGTTAACGGCGGCCAGCGAGTGGGCAGGGAAGGTGACTAGCGCGAGCTACATATT
GCCGGCCGCCGCGCGGGGCGGCCAGCCGTGAGGAGCAATCTGAGGGACGGCCCACCGCCGGAGGGCCTGCACCATGAGGTGAGGCGGCGGGTCCCCG

CCACGCCGCGAGACGGTACGACCCGTTCCGGATGTGCACCACCGATGAAGAAGGCGCGCACATGCCGTTCGCTTTCGGCGTATGGTCTTCGCAGCCTCCG
GTGGGAGGCGCAGCCCGGAGGGTGCCCGCGCCACACGGATCAATGGGTTGGAGGGGCGGACTCGTGGTTTCGACCATTGGTGCAGCTAGAAACCGTCCC
AAGGCTCCACTCGATCGCACTGCCGTCGATCTCGGTTCTGACTCCCACCTATGCCAACACGGGAGTGCGCTATGAAACGCGGTTTTATAGTCGGTAATCG
CGACCGAACGCGGAGCGCCGTCTCGAGGCTTGTAACGCTCCGCGGTCGTTGTGGAGCGGACCGGGTAGTCAGCGTGACGTATTTCTAGCCCCCAGTGTGC
CGCCCGGCGAGAAAGTCCTCGCGGGAGCCCGTCGGCTGCCGCTGCGTACGCGGGGGAAAGCGGCCGAGGTACGTCTTCCTGGTGGCTTGCTGCCGCCAA
CCACCGATCGGCCCGGCTCGATGACAGCCGGCGAGAATGGGTTGGGGCCTACTTGGCCGGGTTGGCTGATCGTACGCAAGCAGCGTGCTGCGGGCCCAC
GCAAAGGGGAATGGCGGGGGAGCGCCGGCTGCCAATTCCCCCCGGCTGCACAGGCATGATCCGGTGTCAAGAACGGTCTCCGGCCCACGCGGCCGCGC
CTTGGCCCGAGTACGGCTGACTCAGAAGCCGTTGGGTGCGAGGGCGTGGGGTGGTGGCGCATAGGGAGCCAGACCCGACGGAGCCAGGATGGTTCGAG
CAGTGTCACGGCCCGGGGTCATGTGGGGCCCGCCCGCACGGATGACCCGCGTCTCGATAATCGGTTGCAGGCGTGCCGTTCCCACGGGGCGCGCCGGTT
ATCGAACGGGTGCGCGCGTAGACTCGCTTCCGGGCCCCGGACCTGGGCCAGGCAGAAGCGGGGATTACCCGTCTTGATCGCAGACAGCCCGGTGCCCCC
GGGGGCCGACGTACAGCTGGCAAGCACCGGTCCGGCGATTACCCGACCTGGGTTGGCAGGTACACGTTACAGGGGTGGCGTGCCCCCCGGCGCTTGGCG
CCGGCTTGGAGGGACGGGGGGGGATCTAGCTCAGATGGCCAGCGAGCGTAGCTTCGACGGTGGCAAGAGCGAGCGGCGGCGTGCTTGGGGAGGCCGCG
GGCGGGTGCGACGTCCAGTTCGTCACACGCATGAGGGGTTTCCGGGGAGGAATCACCGCCTCGCGCAGGCGTGTCCGACACGACCCGTGTAGCGGGTGT
GAGACATTTAGGCGGAGGCCAGAACCCCTTGCCGGAGGGCTTGTAGTATATTGTCAATGGGGCCGACACCACGTGCGGATCGGGACTGAGAGCGCGTAG
AGCCTTGGCCCTTGCTATATTTGTGGAGCGGACACTGTGCTTCCCACACGTCACGCTGGCGTAAGCCGATGCCCGTCACCGCCCGCGCCCCAGGCCGAGT
GCGGGGGGCCGCAAGGTCCTGAAGCCGATCTACGATGGGCTGGGTGGGCGACGTCGTGGGCTAAGCTCGGCGGCCCCACCGTGAGGCGGACGTGGGGG
CCTGTACTAGAGATGGAGCGACGGTCGTCGATACAGTTCTTGCCAGGAGACCGAGCATTTTGCTAGGCACGGGCTGCATGGGCAATTCATTAACGGCGG
CGGGCCCACCGCGGCCAGCGGTGGCGGGGCGTAGTACCGGTCGGCAGGGATACGACCCCGGGCCGAAATTCGGGGCCGGCGGCCAGGGGGCGGCGCG
CCTGCGCGCCGTGACGCTGACGCCCTCCCGGGGTGCCGCGGGCTGGCCTGAGGCCCAAGGAGGCGCCTGGGGGTCGAGCGCGGGCCCGGGCGCACGCT
GATCCCGAGCCGACGAGCTTACGGATTGACCAAGAGCACCGCGAGCGTCCGAGCGCTGCGGGCGTGGTAACAACTACATCCCGGGTCGAGTTTGCGGCT
GGGACGTGGGATTGGCCGTGCAGACGCGCTGGGGGGGGAGCGGGGCTGGCCCCTCGCTCCGTATTCCCCACTCCACATGGTCCCCGGGCGTACGGGCGT
CCCCGACGGTTCGGGACGCGGGAATCGCGCAGGTGCCCCTCGGTGCGTGGCGGGCTGCGTGTAAGGCGCTTTCGCTGCATGCGGCCGTCTCGCGGACAT
GGCGTACGGGGGCGACCAGGGCGAATCCAAGGTGCGCCCAACGGGCCGAGGCGCTACAGATGGGGAGCCCCGGGCGGCCGTACGCAGGAGTCCAGTG
GCGTATGGAGGGAGGCCTGGGGCTCCGCAACGGCCGAGGCTATGCCCAGACCCCATGCCATTGTAGGCCGAACGCGATAGGTCACGCCGGTGATCCTGC
CGGGGTCGCGGGCCGGATCCACAAGGCCAGACCCATAGCGTAGTCTATAGGCGTCCGGGAAGGGTCGTGGGGGGGCTAA

>2017.TE.25009.1.8

CACGCCCCCCTCCCGGCCAGCCCGCCCCCCGCCCCAGCGTCGTACCCGGCGTCGCGACTCCCCGCCGTCGAGAGGCCTTTGCCGGGGCCCCGGCTAGCC
CGGGGCGAGCGGACCGCCGCGCGCGGGGGGTGTCACCCGGGGCACACCGCGCCAGCCTCCCGGGCATGCCACGCCGGGTTCCGCCCCCGCTGCTCCAA
TTTGGGGGGCCCCTAAGATCCCGTTCCCCAGGCCAGCCGCGCCCGCGGGCCAACATTGCCACACCGTGTCGCCCCCCCGCCTGCCGCACTGACCGTCTTG
CGGGCCCTGCTCCCTGGCCCGAGCACACCTCCGGGGACCCCCCTCGCTCGGCCGGGGGAGCCTTAAGCGCGGCACCGAGTAGGGGGTCGCGTGTTGCCG
CCGCGCTGACGCCCCGGCTGGGACCCCTTTCAGGTCGAGACGCCGGGGTGGCCATGCGGGTCCAGTCGCACGATGGCGCGGCGATGTGGCGCCGACTGC
CCCGCCTGTCTTCTCCGTGCCAGCCCCTCGCCACCATGCCGGTCCGGCCCGCACGGGGGTAGGAGGTGCTGCCGCGGCGGGAGGCGTAGGACGCCATAT
CCGAACCGGCGGGTCCCCCCGGCCACCGAGTCGACAGGACCATAGGCACCGCGTCCGGGCGGGCGCCCTTAACGCCGCCCCTTACTGGAGAAGCAGAG
CTCTGCCGAGGCACCCTGCCTAGCTGGCTGCGTCCCGCACCCCGCGATCCCCCTGGTCCACTTACCCAGCGGGATGCGGGGATGTTCGCAGCTATCACAC
GGTCGTGGCCTGCCTCCCTGGGCCGGCGAGAGCACCGCCACCTTCGTGTTCCTTGCCGCCGGGCGACGTGTCCGGGAAAGGCTTCGCCGCGCGCCCGCGC
GGAGCGCCGGGGAGTCCCGTGAGCGGCCGCAGGAACGTCACGGACCCCGGCTTGGTGCGGTCCCCCTCAGTGGTGCCCGACCAGAGACCGCGGAAAGA
CTCCAGGAAACGCCTCGGGGGGAAAGCAGGGCCGCGCACTTCCGCTCCACGCTAGCTGGGGGGACCACGACGGCAATACTGAACACCTGGCTCTTCGG
CAGCCCGGCCTATGAGGGGCCCTCTCGTCCCGGCCAAAGGCTATTTGCCTTCCTGTCCGGGCACCCTCGACCGCACCCGGGCGGGCGCAGGCTTCATTGC
GGCGCGACTCGCCAAAAGTCCCCGCGATCTCAGCGAGTACCCTGAGCGCTGGCCCTAGTCCGCCTGCCCCCTTAATGTCGCTCAGGTCTGCAATCTCTTA
CGACCGGCTCAGGTAGTGGCCGCACGGTGCGTAGGTCCGCTGCGGGCCGGGCCACTCGCGCCGATCGTGCCGTGCGGCGGCCCTGACACCGTATGCGTG
CGCGCTCCGCAGGCTCGCGAAAATCCTTCGCAGGCACTTGGCCCCGTTGCTAGACGTGGTGTCTCCTAACTGTGGCTCACGCACGGCGTTTAGGCCACGG
TAGCCCGCTGTTCGGTCCTTACGACTCGGCACACGCCCCCGTGCACGCCCTCGGGTTCCGCAGGCCAACGCCTGGCCCTAGGGTTGCGTGCCGGCCTGCC
CGCGATGCCACTTGCCCCACGTCCCCCACCTGGCCGGCGCAGACGGGTTCAGTGGGTGTCGAACGCCATTCAGTCCCCTGGGACTCCCCCGACCCCTCCC
ATGGCACCCGCCGCCTGGCCTTGGAGCTCCTGCGCGTTGGACCTTACTGCGGTCGTCAGCTTCTCGGCGCGCCCCGGACTAGTCCTGGGCGGCCAGCTCG
CGGGACCCTAGCGCGCACGCGTGCCCGATTGCCGTATCCCCTCGACGGCGCCCGGGCATAGGGTTCGATCCCGCTGCTATTGCGGGTGCGTCCAGACCTG
CCGCCGTTTGGCGATCCGAGTGTGCGAAGGGAGCCAGCCAGCGGGCCGCCTGGCCGCACTCCAGGATCCTGGCGCCAAGGGAGAGCCCTGCCAGCGCT
ACGCGAATACTCGACCTGTCCGCGCCCCGCCTCGGGCTTGCGAGATGGCCACTAGACGCTATCGGGTCGTCACCCCGTCGGCCCCACAGGGGCCCGGCTT
CGCGTGGCTTTCTTGTCGAAGGCCCCGAAATCCGGCAGGCCCCGACACCTTCACCGTGAGCGAGGACGCGCGGTGCGGGATGTCCAGTTCATGCACGCC
CCTCCACGGCCGCCTTCGCGCGGACAGCGTCGGGCGTGCCCAGGACGAGAACAGACCCTGGGCCAGCCTCAGGGCCCGGCCTAGGCGACCCTGGTGAC
CAGTTTCCACTCGGCACCCTCGGGCTTCGTGGCAGTGCAGTCATGCCCCCGGGTGCTCCCGCGCGACCGCGCACCTCCCACCCAAGGTTCTTGGTCAACG

CGCCGGATCTGACGTCGCGGTCCGGCCCGCGCCGTAGGCAAAAATGGGTTCGGCTCCCGCCACTCCGCCAGTTCGAGGGCGGTAAAGATCTCAGCCGCC
GTGTTTCGGGCTATCTCCCCGCGATCCCGGGCCACAATCGCAGGTGGCTGGGGCGTCGGCCAGACCCGTCTCGTCGGCCTTTCTGCGCACATTACCTCCGA
GCAAGGCAACCGCTCCGCCCCATTACAGGGGGTTGTCTGGTACCTCCGTCACGTACCCCCGCATCGCTGGCGGGCTGACCCAAGCCCCCTCCCATCCCGA
CCCTCACGCGCTCACGCCCCTTGCCGGGCGGGGTCGTGGCTGCCCAGGGTCCAGGGCCCGCCCCGGGGGCCGGTTGGTTCCAGGAACTGAGGGGGGGTC
GCCCAGCTGACCGCTCCTCTCGCGGGGTTTATTCGGCGTGGCCATCCGCAGGTGCGACAATGCCGGCTCCCATTGCCGGGCCGACCCTGGTCCCTGGCGC
TTCGGACTCCCGGGGAGCGCCTCCCTTCATCGGCGGCCCCTGCCCCCGTCATGACCCCCCTTACGCGCCGGTGGTAGCCGCGCGTGGTGGCGTGCCCCTCT
CCATCATTACGCTCCACAGGAGTGGCCACCGGTATGGGCGCGGTGCAGTCGTACATGTGTGCGGCCCTGTTTTGCGAACCCTTCCCGCGGTGACCTCCGC
CCCAGCGGCCTTCTTCGACCGTTAACGCCGGCCAGCACCCGAAGGGGGTCACACAAAGCCCTGCAAGGATGCGACGCTGCGTAGCCGCAGGTTAGCCGC
TTCCTTCCCAAAACAGTGTCAGAGCTGTCCGGCGGAACGTTAACGCCGCCGGGAGGATCTGCGGCTACCCTCAGGCCCGTCCCGTGGGAGTGGCCGGCG
TCGATGGGGGTACGCACCCCACTAGACGCGGATTTTGGCCAACGGGCACGGCCGCTTCCGAGTTCCCCTGCGGTCTGAGTGTTGGTACGTTCAGCCGGCC
GTTCCGTGGGGCAGAGCGCCGCACGCATGTCGCAGGAGCGCGCCCTCCGCGTCTTGCGGGCGGGGTCCTAACGAAGCCCCCTGGGCTTGGAGGTAGGGG
GCGCGGGGGAGCGATGGGAGCGCAAGCGTGCCATCGGGTCCGGCCCATAAGTGTGTATGGCGCACCGCGCTGCCACGATGTCCGGTGCGCGGGCGCCG
CTGGCTAGCTCACTCTCCCCCGACTCCGCCGCCGCGCCCCGAGACGTATGGAACAGCTGGCGCGCGTCCCCCGCCGCGGGCGGCAACGAACTAGATCCA
CCGCCCTCACGAGGAAGCGCGCCGCCACCACGTCTCCCCGGCCGACGCGGTCCCACCATCCGCTGTGCCGGCGCGCGAGCAGCCTGGCCCTCGGGGCGG
TCGCGCCAGCGCAGACACCCTGTCACGCGCACGGGAGCCCTGGCCACCGCGTCGACGCCCGGGCTAGCCCCGACTATCAGTACTGACCAGAGGGGCGC
CGGTTGTCCCGACTGGTTCCTCCCGAAACGCTGGCGGTGGGCCCGCACGATTGGTGCAGGCCCCCAAGAGGATACGGCTATTCGCGGCTGCCCGAGGCG
AGTCGGGCCAGACCGGTCGCGCGTGTGTGTGTTAGCCCTCCGTCGCGGCCAAGCCGGCGGCACCGGACTGGGCGACAGTAGCGTTCCTCTGTCACTAGTC
ACTCTAACGGACAAATGGAAAAATGGCACCTATCGCTACCGACGGGGTTTTCCTCCAGGATAAGCCTCCGCGCCCCCCACTGGACGCCAGCCATCCTTCC
CCGAACGGCTCGCCTGTGCCGGCTCCAACCCCGCGGGACCTCCCTTCGCGGCCCCGGCGGGGATCGGTGGATCGTCGCCGCCGGCGCCTACCGATCGCG
CCGCCACGGTCGTGCACCAACCAAGCACTGCTACACGCCTGTGCGGCCGAGGCCGCCCCATCCAGCAACGGGGCACCGTTGACCCGTCCCCCATGATGC
CGGACATGGTAGCCCCCACACATGCATCTTCGGGTAACCCGCGCCAGGTCCCCCCCCGCGCCGCCGCCGGCTTTCCCCCCGCTGGTGCTAAGCGGCAGAT
GCACCACTGACGGGACACACTCGCCCCCGATCCAGTGCGCTGCCGCCTGCGGCCCCCCACCGGACTGGTGCCTGGCGCTACACGGAGCCGGCCGCGCAC
TGTGACGTATGGATGCCCGAGGGGACATCCGCCCCGGACGCGTGCGCAAAAGACCACTCGCTCGACGCCACGACGTGTGCGGCCGGGGTGATGAACGT
CGGCCGGTGTGCCAGTGAGGCTTGCACCTACACTCCCTGGTCCACCGGGCCGTTCGAGCGGCACCGGGCGAAGAACCCGCAGCCTCCCGGCTTACGCAC
CGGCCTCGCGTCAACAGGCAACCACGACCGCAGGGCCGGCAGTGTGTGTCGCTGTGGAAAGGTGCGCCGCAGGCGACCCCCACGTCAGCCCACTACAA
AGCGTGGCAGCATGCTGCGCAGCGACCGCACGCCCCGACACAGCCCAATGCACAGCATCGGGGGGGCGGAATCGCCCCACGCCCGTAACATCCCGCGG

GTCGACCGCCTTACACGTCGACTGCCACACCCTGGTGCCGCAGTGGTATCCCGAGACCGCATGACCCGCCGCGAGCCCGCTGACCACCTGTCCTTGATCG
CAGTCGCCACGGGGCCGGAAGGTGTACCGCATACCGCACGTGCTGCGCGTGTGCAGCACGGGGGCACCCTGCATGTATGGATCCCTCTAAGCCGACCTG
CCACCCCAGGCCAGAGGCTAGAGTCTGCCGCCTAGGCCCCCCCCGTGGTCGCCAGCCCCGGTGGCGGCCCACCCTCGGGGCGACTGACCGGCCACCAT
CCAGGGTAGAGAGGTTTGGCCCTAAATACGGGCGTGCCGAGGAGGTGTTTGGGGCGAGCGGCAGCAGGTTCGGCGGATCCTTGAGGGTGGGGCCTGGG
GTGGGACGGGGGCGGCCGAAACGCGAGCATAGGCCCCTGCCTGGCCCCTTTGCAGTCTGTACTTTTATTGACATGATACTAATACCGTGTTCCGCCCTGA
GATCGCACGCGGACCTACCGTGCCTCTCTCTGCATAGTGCTTCAAGAGCCCAGCGCGACTGGGGACCGGCTCCCGCAGCCGGCTCGGGGCGTAACGCTC
GGTGGAAGGGTCACCCGGATAGCTCCCGGTTAGTTTCGTACAGGGTGTCGGGGGCGCCCCCAGCGAGTCTCCTGGAGGTGCAGGGACGGCGGTGGTCCT
CGAGGGGGCGGACGTTGGCCGCATAACGGGTAGTCTCCAGGGTCACCTATGGTCTGGCTCGCCGTCAGGGTCTGTGAGCGTCGTGCTCACGCCTCCGCAA
CAGCCGGCGACCCCGGCTCGCAACGGCCAGCCGTGGGCGGGGGGGCGACCGGACGCAGTTCCGAGTCTAGGCATGGTGCCACCGCCCTCTCGTTGGGTT
AGTCTCGAATGGCTGTTTCGGGGGTCAGCGCGGCGGGGCCGCCGCTGGGAGCCCGCATAACCGTGGATACACTTGCTGGGTGCGCGCTCGAGGGCGGCC
CCCGAGGGCTGGAAAACCATTTCGGGATCGCCCCCGCGCTCCCGTCCGCACTGCGGGGTCCAGCCCGACGGGGGCGGGTTTCGTTGGGGCAGTACTCAC
GCCCACGGCGAGGCCCATCCAGAGAGTGAGGGTGTCGCTGGGGGGGCCTACCGGGCGGAACGCGCCCCGACTTAGGGCGCCGCAATGCACGACCAGTC
ACCGTCTGGGCGTCGCCGCGCCGTTGAGAACCCCAACACAGAAAGAGCTTGCCGCCTCTGGGGGAGCCAGGCCGAGAAGCGTGGGCGAGCGGCCACGC
TGCGGGCCCTCCGCGGACCGTGCGGCGCTCTCGGGTGACAAACCTAGTCCCCGTCCCAGGCCATGGGCGAGCAGCTGCGCCGGGACTGTGCGACGCCTA
TCCGGCACATTCGGTATTATTGGCCTGGGACGACAGCCGGCGACAGGGCGGACGAGGCGGCGTGGACTCGGGGTGCAGGGACGACGTCCACCCGTACT
GGTCAGTAGCCCAACGCGGAAGCCTGTCTGGGGCTTTGGCCCCTAGACATCGGTCCGGGGCGGGGCGCGTCATCGCTACGTTAGTGAGGGGGAGTCTGC
ACCGATAGGGTGTGGTGGTGTTGCTTAGATCGTTGACGGGCCGTGAGGTGGCGCTGCTATTTTCCGGCTTACGGCTTACGACCTACCGCCCGCCGCGCCGG
CCCGAAGGCGGCGCCCCCGCACATCGCCCTTGGGTGGTAAAACCCGCCCTGCCGCCGCTACCCCCCCCCCAGGGGTGGCCGGAACGGCGAGGGCAGGA
CACGGTCGACGCACGCAGCGCCGTGACGCCCGGCCCGCGCTAGGGTTGGCACCCGTGGGACCTCGACAAACTTTCCGTACGATTGGGGGGCGCATACAG
CGGACGGGGAACCCGCCCGACGCACGCGCGGTCCCGAGGGGTGGGGGACCTGCAGCTGCGTCGCGCGGTCCGCGAGGGCGTCTTGCTAGACCATGGGC
GTTGCAATAGTGGGAAGGTGCCTCCCAGGCTGCGGGGCGCCGGACGCCCGACACCAAAGCTGTGCGAGGACAGAGAGGCTAGGCACGGATGGGTACAA
GCGAGCTGACAGCCTCCATCCTTGAGTGAGCACGCCGCGGCGAAATGCCGGCCGAAATGGGAGGGGCGCGACACCGGATCGTGATCGAGCGCGTGCGC
TTCGCCCACACGCCCTGACCCGCTGAGTGTCTCGGGTTTGAACCCCGTGCCCGGGGGCGCAGTGGGATAGGCACGACGGGCAGAGTTAGGGGCCTGGGC
ATAACGGCCCTGTCCGCGGCCGAGCGGGCCCGGGTCTTCTCGACCAGGGTGCCGGCCCCGGCGGGCCCCCCGCCCACGGACGGGGACCTGGAGTTGAGC
CAGGCCACAGGCGTGACCGGAATTGGAGCGCCAGGGGACAGCGGCCGCCCGCGCCAGGCCACGCCGATGGGGGCGTTCTGCCCAGGCGTCGCGCAATG
GGCGGACCGGTCTACGCGACACCGCCCGCCCGGCCTGGAACTCCCCCCGAGCACATTCGATATGGTCCGCATCAAGGCGGGAGGCATCGCCGGGGGCC

GCGCAGACCACCACCTTCGCGGGGGACGGTGCGGGTCGCGACGAGGCGGCGGCATGCGCGCGGGTGCCGTGAGCCCTAGAGTCAGCGGTTGCTGGTAT
GGGCGGACGGGCGCCCCCGACCTGCCGTGGACGCAGGCTCGCGGACTAGTCGACCCGCCATGGGTGCTGTGCGTGACGTTGGCCTTGGTCCAGGGCGCG
ACGCGCAATACCGCGCGGGCTGAGTATCGCGTCCACGGACAGGCGGCTGAGCCGCCCGGCGTAGCTGGTACACGGTCGGGCCGAAGTGGCAAAGAGCC
GGCTTGCGCCCCCAGCGCCGTTGCAGCAGGTCTGGACAGGCAGTACGGAGCGAACGCGGCGAGACACCCGGCTCCGGGCGTAGTCGGATCCGCCCGCC
AGCGGGCGCGCAGCGGTGTCGCGGGCTTGATGCGCCGGTCAGGGAGGTCGTCGCGAATGAGCCGGGTGGAGGGTGCAAGAAGCCAGACGATGGGAGC
ACCGAGTTGTCCGCGTGCGATTGCCGTCCCGGCGAAAGCGCCAGGCCGCGGCGCGTGTGGCAACGGCAAGTACCCGGAAACACGGCACGGGTCTCGTG
GCGCCGCGAGGCGGCGGGTGGCGCGCTCCGGGGGGCTGCGCAGCCCCTCAGCGCCGCTGCTAGTGCCAGACGCTTGCCCTTCTTGAGCGCGGCGGCGGG
AACTCGGTCGCGTGCGTTCAGGGCCCTCATCCGCAGACGTGAGGGGTGCGGGCCAGGGGGCGCAGAGGCGCCTTCCGAAACCCGTAAGGGATGTTCTGG
CGTTGGGGGGCCGCCGTCTTAGGGGGGTAGCGCCGCCTCAACTCCAGTATCGCCGCAAGGCCGGTGCCAGGTTCGATCCGGGGGCGGCGGCGTACCGCG
TCCACGCGTCTCCCAGAGGAGGGTCAAGGTGCATACGGGAGGGATCATACGGCAATCGGCGGCCGGGTTCCGCCATCTAACGTGGCGATCGCAGCGTAC
CAGAGCGAGGCCTATGGGCCATATCCCTGGCGGGAACGGGCTGCTTGCGCCCCGTCCTCGCCGACCCTCCGTACCGGTACGGGGCGAGGATCCCGACAC
ACAGCACGGGGGCGGCGCGCAGCACCCACCCCGTCCTCGGCCCAACAGACAAAGACCCCGCGGCGGGCTGGGGCACCCCCTTTCCGGCTACGTCCACC
GATTCTGTACAGGACAGCCGTTGCGATGGGCACCGGCGATCGGAGAGAAGGACGCCACAGATCAGCAGACGACTCGGGGAAACCACGGGACCGCGCG
TCGCGTCAACTGCCGCGGGAGTGAGAAGGGAGCGACAGGAGCGCGGAGGTAAGGCCAATGGGGCCAGTTGAAGCCGCCCACAGAGGCTGTGCGGGGC
CCCGCCGGGTTTAGGAGCTGTTCTTGGAGCCTGTCGTTAGGGGCGGGGGGGGGCTTACACGGCGCTGGGGGACCCACGAGGCGCAATCCGCCGCGGACT
GCTGGAGGGCTACCTGGGGCAGCACCCCAGGGGCGGCCATGGCGCTTGTTGACGGGGGCGCTAGGACCTTGTCCTAGGGCGCGCCGCTTTCGCCCACAG
GGCTTCGGTGGGCAGTCTATCTGTGCGGTTTCAGTCTCGTACGCACGTGATGCGGTGTAGCCTGAGCCCGGCAGTTGTCTAGAGTAACTAGCGCGCGGTTA
TGGCTTCGTCTGGAAACGGGCTGCAATGTTCCGGGTGTTCATCACACCACGTTCCGCTTTGCAGCTCACCCCCCGACCACACCCGGATTCGGACCGGTGGT
CCTTCCTCTGAGGTACTCGCCGCTCATACAACCGCGGTTCCCGCTGTGGTCGGCACGGCCCGGGTTGCAGGGGCGTGCCTTCCCGGGTAGTGTGCCTGCGC
GGCGTGCGGCTTCCGCAGTCCACGGACCGTTCCGGGGGTGAACGATGGAGGTGAGGGGACGAGTCCCTCCGAGATTAATGCCCCGCGGCAAGCCCGATC
AGCGCTGCGGTCCTGGGCTGTCAGCGCGCGCGGAGGAGCGCCCGATCGGGCGCGGGGGAATGAGAGTGTGGAGGATCATAATAGAGAGATCGCCCCGG
AGACTGCGAGGGGCATGGACGCGGAGGGACGGGGCGGGGGGTCGGCACGCCCGTGCGGCCTGTCGTCATATCCCACGCTGTCACTCGGCTGCGTCCGGC
ACTCCGGGCGGCCGGCGAGCAAAGCCGCCCTGCGCGGCCCGATCGAGGGTGCGGCGGCGCGACGTCGTGGCGTGGGAAATGCGGCGAGGGGCGGGGT
GGGGGACGTCATTGAACCTGCAGCGCGCACGGTGAGTACCGCGGCCCGTGGCGGGGCTGTACTGCGGGACCCCGGAGCGGTAGCGGGGGTTAGACTGG
GTGAGGGTAACCTGCTGCCCCGCGCACCACGTTCTTGGCCAGAGGCCCTGTGGGTGTTGGAGCAGGGTCGAGTATCCCTCCGTGCTTGCCTGTGTGGAGT
GGTTCGCCATGCAGTTGGACTCTTGGCGTCAGCTGGGGCCAGGCGGCGCCTCCGAGGTCGTAAAGGTCAACAGCGCTGTGAGATGGGCGCAGGAAGAGC

GCCAAAGACCTCCTGCGCCCCGATGTCCGCCTCGGGCGGACGTTCGGCCGCCTCGGGACTAGTTAAAGTGCGGCTTGGCTGGGGGCGCGGGACTCGGAA
GTCCGGGTGGAAGATGCGGGGAAGCTGCCCGTCGCCGGTACTGGGGGACTGTAGCTGTGATCCGGGACCACGAGGCATGCACCCGTCGTGGTCCAGCCG
GCCTGGCGCGGGGTCACAGAGCGAAACGGCGGACGCTAGGAACATTCGCTACCACCCCCCGGCGGCACCTTGCAGGGTCTACCCCAGTAAGGACCGGC
CCCAGCAGTGAGTGCGCCCGGGGGCTGGCAGCGGTAGATTGGGTCGCACGAGCTCACGTGGGCCGTTGACCGGAACCGGCAACGCCTGGGACTGGAGC
GCAAACTGGGGTACACTCTTCACGCCCGCTGCTCGTCATCGACCCCGGCGGGCGCCCGGGACCGGACCGGGGGCGACCCACCGGGGGACGGCGCGGCC
CCTGGATGGCCCTGCCCTCGGTCACGAAGCGGCCGAATGGTTTGGGGGGCCTCGGGACGCGGCCGTGGAGGTGCGGGGGGCCTGAAGTCCCTGCCTGGC
CCTCCTCCTAACGGCTTCACGCCCCTCAGGGCAGCTAGGGATTAACGTGGCCGGGTCACACGACCCGCCCAGCGCGGTACGCTGGGCCGCAGTGATTGC
GCGCGCCTGGCGGGTCCTCACCCCCCCACACACATCCGGGTCGGGGCGGAACTGGGTCCCGCCGTTCCCTCAGATCTGCCCCCTGACTTGAACATGCCCA
AAAACCGAAACCGGGCCGGCGCCCAGACCCCCGTGCTAAGGTTCCCACCGCCGACCCTAGCCCCCCCGGAGGTCCCCTGTACTCCGCACGCGACGTCGT
ATCAGGTCCCAGAGCCCGACAATGCATGCGCGTCCCGCTCTGCGCCATGCCCATTGCCCGACCAGCCCCGCCCAGCGCAGAGGCCCCGACTGCTGGCGC
CCTCTGTGGCGCTCGGCCGCTCGAGAGCACCTGCCGCCCGACCGCAGTCTGGCTACCTGCTCCACGGGGCGGCGCTCGTGGTCTCCCGTACTCGGAACGC
CACGCCCCGGGCTCGCGCGTCTCGCGGTCCGACGCATCGTGGTCTTACCTCCGACCCTTTCCTGGGGTCGAGCGCGGGGGGGCAGAGCGCGCTCATCCAG
CCCCGCGCGGCAGCACCCGCCCGCCCAGGGTGGTACCGCTCTCGAGAGTCCGGACGGCCGAACGCCAGGGCAACGGGCCGTGTGAAGACTCACCGTTT
CGGCCGACCGCCATCCTAGCTGGGGCGAGCCCCCGCGCGCGGCCGGATGCGTATGACGTGCGCGCGCTCGGTGCGATGGCGCACGTCGACTGGAATAAC
CCCTTGCCAACCCCAGCGCCGAGAGTCCCTCTTCGGGTCTCCCGTCTGCCTCCGATGGGTGGCCCCTGCCGGTACTTGTTAGGTCCTGGCTCGCCGGGCGC
CTCGTCACCTCGGCAGCCTGGATCGCTTGTCGCCGCCGCAAACGTGCGCGCGTTTCGCCACGGGCCGCCCCGCGAAAAGCGGCGGGAACGTGGACCACG
CGCGCGCCCGCCATCGGCTCGCCGGGATCCCCACCGCGGCAGCGCCCCGCCACAGCTCAGGACGCGGCCGATCGACAAGCACATCTCCCGACTACCGG
GCCCCGCGGCGTGAACGTGGTCATGGAAAACCCGAGCGTGTGCGTTCCTGTCACGGGATCCCGCCGTGGGCAGTTCGCGGCAGGGGCCTACCCGTCGAG
GGGCTACACCGGACCGCCCCTCCAGCTGCATCGTGCAGCCCTAGTGCTGCAGCGAACTTGCCCCGGCCATCCCGTCCGCCCGCAAGCGGCTGGGGACAG
GTTCTCCGCCCATGCGCAGCCAGTGGCCTCATTCATGAGGGCGTGGCGGGCCGGGCCGCGTTGGGCCATCGCCGGTTGGGGGCCCCCACGCCGTCTACCT
GAGTGGACGCGCCGGGCGAGGCTCCCACCACCCCAGTCCTGGGGTCCCAGACGGCCTCCTGGGCGGAGTGTTCTACCCCGCGGCCACCCCCAGCCCGTC
TGCGGCCGAGGCCCCACTGGAGACTGCATTCCCCGCGGCCGGGGAACCGGCCACACCGGCAACGGCGCTCCTGACCCCACCCGCCTGCCACACTCCCCG
CGGCGCGGAGCCCCACCCATACTAAGGCCCCTACGCGCGGCCGACGACATGTTGATGGCTTCGCTGGGGGGGCGGTGACACGTCCACGAGGCGCCTCGC
CGCCGTCGGCCCTGCCCCAGCACGTTCCCCCGTCCCCCGGGGGTGCGGCGTCCTGCTCGCCCCCCGTGTAGGAACGCCGACCGGGCTGCGTCGTCGATGA
GGCAGTGGCCCGCTACGCGGCTCCTCTTCGCACGTGCTTTTGGCTGAAGAACGGGCACCCCCACCATTACGCAGTTGGGACGGCGCGCGGCTGCGAGGG
CCCAAATCTCTCCCATGCGAGTCGTGGTCGCGCCCGTGGACCTCTGAGGGGCAAGGCCGCTCGTCTGCTGAGCGGGGATGTTGCGCCCAGCAAATCGAG

GAAGGTTTGCTCGTAACTTACGGGTACCGAGACTCCTCGCTTGGCTCACCCATTGCGGCAGCCGCGGCCGCCGCCGGGACCAGTAACGGCGTGTCGACC
GCCCCTCGACAACGCCTGGTAGGACGCTCCCCTCCCGGCCCGGACACTAACCCCCGCAACAGCGAATTCCAGGGGCGTCTTCCGCCCAACAAAGACCCA
TCGTGCACGAATTGGGCTGGCGGGCAAGCATGTGCATCACGGACTAGGGTACCGCCTGCCTCCCCGCTGCCTCCGGGGCCCTGGCGCTCCGTCCGATTTT
ATCCCGCTCTGCTGCGATACTCAACCAACCGTTTCGCTTTATTCAGGCCGCCTCTGGGGCCCGGCCGAACACTTTCTCCCGACAAGTGGCGTGACGGACCT
GCCTTCGCGCCGCCCCGAAACAGGCGGGCCGGCCTCGCACCCCTGGGGAGCGGTCCGCCGCAGGCGGGCCTCCTGAGCGCGCGGGTGGCCGGCCAGCG
CGCATACCCCCTCGTCGCTGCCGCCCCCCCCGCCCCGATCCCCGGCCCCGCTCGGCAAGACAGTGGAACCCGCCGCGCCCTTCACTCGCCCGGCTGGCCG
TATTTCGTGGGTCCTCACCTCAGTCGCGTCTGACAGATTAGACCTCGCCGCTGATGCCTAGGGTGGGACACCACCTTCCCGCCCGCCACCACCCTGTGGA
GCGTGAGCGCACTTAAGTCTCCCTACCTCCCCAACTCAGCAGCGGCCCCAGGGCATCCGCGAGGCTCCGTTGACGCACGTGCCGTCGTCCCGCGCCCACT
GTGGCACCTCGGCGGCGACCCTCCGCTGCGGGATGCCTGGAGGAGCGGTCCAGCCTGGGCGCTCTGCCGGTCACCAACGGCGGCCGGGCGGGGGCCCTC
GCGGTCGGACCGAACTTCGGAGGGGTCCCCGGCAGCTGAGTCGAGCGCTGCTAGTGACCTCCACCTCGGCCTAGTCACACTTGCCCGGCTCCCAACCGCT
AAGACATAGGGGGCGTCGCGACGAGGGCAATGCGGGGGCAACCTTGCCACCCCCAAGCGACGTCGCCTCATGTAGGTTGGAGGGGCTGTCTTGTACTCC
ACCGAGGGCGACACGTAGCGTGCGGCGGCTCGACTCGAATCTTGCGCCTCGGTACGCGCCACGCGACCGATGACCTTTGCAGGCGACTCTGCGAGAATG
GGGCCCGCGTCCTGGGGCGTGCTAAATCACGACCGCCGCCGCTGGCCTCCAGGAGGTCCCAGGGGGCAGCACACGCCATTCGCGTCGGTGGTCTAGCCT
CGCGGCACCTCGACGAGTCCCAGGACGCGTCGGGCCCGGTTCTTCCTCTTCCTGTGTGGCAGTCCGGCCGGGTGACGCTTCCGCCGCTGGCTAATTCAGA
CCGCCGTCAGTCGACTGGGCTGCCCTAACCAGCCAGGGCACTCGTGGACTTCCGGCGCCGTCCGACTCCACGCGGCGCGCACAGTCGCCTCCCTCCTTCG
TACCATTCGGCCCTCCTCACCCCAGGGGGGGCTTGCCGCCATTCCCTACGCTGCCATTCGACGTCCCGCGCTACTCACCCATAGTGCCAATTGCGGCGTCG
CGGACACCGGCCTGGCGCAGCGCCAGAGCGCCACCTGTGACGTCGCTCACCGCGGGGGGAGCCCAGCCTCGACGCCCCAGATGGCTCGCGGGGGGGGA
GGTCCCGAGGCCACCCTTCCAGGTGGCACAGATGCCCCTCCCTGGAGGGGGTGGAAAGCGCGCGGCGGGCCCAACACCGTGGGCGGCAGCTGGGCATG
CGCTCGGATAAGAGCGCCAGGATGGCAGGAGGGCCGATCTGGCGGGTGGTGGCCAGTGCGGGGCGGGGGGCGAAACTGTCTGGGGGTACCTGGAGCCC
AGGGTTCAGGGTCCGTGCCATGAGGCTGTCAGGCAAGCCAAGCCGTTAACGGCGGCCAGCGAGTGGGCAGGGAAGGTGACTAGCGCGAGCTACATATT
GCCGGCCGCCGCGCGGGGCGGCCAGCCGTGAGGAGCAATCTGAGGGACGGCCCACCGCCGGAGGGCCTGCACCATGAGGTGAGGCGGCGGGTCCCCG
CCACGCCGCGAGACGGTACGACCCGTTCCGGATGTGCACCACCGATGAAGAAGGCGCGCACATGCCGTTCGCTTTCGGCGTATGGTCTTCGCAGCCTCCG
GTGGGAGGCGCAGCCCGGAGGGTGCCCGCGCCACACGGATCAATGGGTTGGAGGGGCGGACTCGTGGTTTCGACCATTGGTGCAGCTAGAAACCGTCCC
AAGGCTCCACTCGATCGCACTGCCGTCGATCTCGGTTCTGACTCCCACCTATGCCAACACGGGAGTGCGCTATGAAACGCGGTTTTATAGTCGGTAATCG
CGACCGAACGCGGAGCGCCGTCTCGAGGCTTGTAACGCTCCGCGGTCGTTGTGGAGCGGACCGGGTAGTCAGCGTGACGTATTTCTAGCCCCCAGTGTGC
CGCCCGGCGAGAAAGTCCTCGCGGGAGCCCGTCGGCTGCCGCTGCGTACGCGGGGGAAAGCGGCCGAGGTACGTCTTCCTGGTGGCTTGCTGCCGCCAA

CCACCGATCGGCCCGGCTCGATGACAGCCGGCGAGAATGGGTTGGGGCCTACTTGGCCGGGTTGGCTGATCGTACGCAAGCAGCGTGCTGCGGGCCCAC
GCAAAGGGGAATGGCGGGGGAGCGCCGGCTGCCAATTCCCCCCGGCTGCACAGGCATGATCCGGTGTCAAGAACGGTCTCCGGCCCACGCGGCCGCGC
CTTGGCCCGAGTACGGCTGACTCAGAAGCCGTTGGGTGCGAGGGCGTGGGGTGGTGGCGCATAGGGAGCCAGACCCGACGGAGCCAGGATGGTTCGAG
CAGTGTCACGGCCCGGGGTCATGTGGGGCCCGCCCGCACGGATGACCCGCGTCTCGATAATCGGTTGCAGGCGTGCCGTTCCCACGGGGCGCGCCGGTT
ATCGAACGGGTGCGCGCGTAGACTCGCTTCCGGGCCCCGGACCTGGGCCAGGCAGAAGCGGGGATTACCCGTCTTGATCGTAGACAGCCCGGTGCCCCC
GGGGGCCGACGTACAGCTGGCAAGCACCGGTCCGGCGATTACCCGACCTGGGTTGGCAGGTACACGTTACAGGGGTGGCGTGCCCCCCGGCGCTTGGCG
CCGGCTTGGAGGGACGGGGGGGGATCTAGCTCAGATGGCCAGCGAGCGTAGCTTCGACGGTGGCAAGAGCGAGCGGCGGCGTGCTTGGGGAGGCCGCG
GGCGGGTGCGACGTCCAGTTCGTCACACGCATGAGGGGTTTCCGGGGAGGAATCACCGCCTCGCGCAGGCGTGTCCGACACGACCCGTGTAGCGGGTGT
GAGACATTTAGGCGGAGGCCAGAACCCCTTGCCGGAGGGCTTGTAGTATATTGTCAATGGGGCCGACACCACGTGCGGATCGGGACTGAGAGCGCGTAG
AGCCTTGGCCCTTGCTATATTTGTGGAGCGGACACTGTGCTTCCCACACGTCACGCTGGCGTAAGCCGATGCCCGTCACCGCCCGCGCCCCAGGCCGAGT
GCGGGGGGCCGCAAGGTCCTGAAGCCGATCTACGATGGGCTGGGTGGGCGACGTCGTGGGCTAAGCTCGGCGGCCCCACCGTGAGGCGGACGTGGGGG
CCTGTACTAGAGATGGAGCGACGGTCGTCGATACAGTTCTTGCCAGGAGACCGAGCATTTTGCTAGGCACGGGCTGCATGGGCAATTCATTAACGGCGG
CGGGCCCACCGCGGCCAGCGGTGGCGGGGCGTAGTACCGGTCGGCAGGGATACGACCCCGGGCCGAAATTCGGGGCCGGCGGCCAGGGGGCGGCGCG
CCTGCGCGCCGTGACGCTGACGCCCTCCCGGGGTGCCGCGGGCTGGCCTGAGGCCCAAGGAGGCGCCTGGGGGTCGAGCGCGGGCCCGGGCGCACGCT
GATCCCGAGCCGACGAGCTTACGGATTGACCAAGAGCACCGCGAGCGTCCGAGCGCTGCGGGCGTGGTAACAACTACATCCCGGGTCGAGTTTGCGGCT
GGGACGTGGGATTGGCCGTGCAGACGCGCTGGGGGGGGAGCGGGGCTGGCCCCTCGCTCCGTATTCCCCACTCCACATGGTCCCCGGGCGTACGGGCGT
CCCCGACGGTTCGGGACGCGGGAATCGCGCAGGTGCCCCTCGGTGCGTGGCGGGCTGCGTGTAAGGCGCTTTCGCTGCATGCGGCCGTCTCGCGGACAT
GGCGTACGGGGGCGACCAGGGCGAATCCAAGGTGCGCCCAACGGGCCGAGGCGCTACAGATGGGGAGCCCCGGGCGGCCGTACGCAGGAGTCCAGTG
GCGTATGGAGGGAGGCCTGGGGCTCCGCAACGGCCGAGGCTATGCCCAGACCCCATGCCATTGTAGGCCGAACGCGATAGGTCACGCCGGTGATCCTGC
CGGGGTCGCGGGCCGGATCCACAAGGCCAGACCCATAGCGTAGTCTATAGGCGTCCGGGAAGGGTCGTGGGGGGGCTAA

>2017.TE.25009.1.9

CACGCCCCCCTCCCGGCCAGCCCGCCCCCCGCCCCAGCGTCGTACCCGGCGTCACGACTCCCCGCCGTCGAGAGGCCTTTGCCGGGGCCCCGGCTAGCC
CGGGGCGAGCGGACCGCTGCTCGCGGGGGGTGTCACCCGGGGCACACCGCGCCAGCCTCCCGGGCATGCCACGCCGGGTTCCGCCCCCGCTGCTCCAAT
TTGGGGGGCCCCTAAGATCCCGTTCCCCAGGCCAGACGCGCCCGCGGGCCAACATGGCCACACCGTGTCGCCCCCCCGCCTGCCGCACTGACCGTCTTGC

GGGCCCTGCTCCCTGGCCCGAGCACACCTCCGGGGACCCCCCTCGCTCGGCCGGGGGAGCCTTAAGCGCGGCACCGAGTAGGGGGTCGCGTGTTGCCGC
CGCGCTGACGCCCCGGCTGGGACCACTTTCAGGTCGAGACGCCGGGGTGGCCATGCGGGTCCAGCCGCACGATGGCGCGGCGATGTGGCGCCGACTGCC
CCGTCTGTCTTCTCCGTGCCAGCCCCTCGCCACCATGCCGGTCCGGCCCGCACGGGGGTAGGAGGTTCTGCCGCGGCGGGAGGCGTAGGACGCCATATCC
GAACCGGCGGGTCCCCCCGGCCACCGAGTCGACAGGACCATAGGCACCGCGTCCGGGCGGGCGCCCTTAACGCCGCCCCTTACTGGAGAAGCAGAGCT
CTGCCGAGGCACCCTGCCTAGCTGGCTGCGTCCCGCACCCCGCGATCCCCCTGGTCCACTTACCCAGCGGGATGCGGGGATGTTCGCAGCTATCACACGG
TCGGGGCCTGCCTCCCTGGGCCGGCGAGAGCACCGCCACCTTCGTGTTCCTTGCCGCCGGGCGACGTGTCCGGGAAAGGCTTCGCCGCGCGCCCGCGCA
GAGCGCCGGGGAGTCCCGTGAGCGGCCGCAGGAACGTCACGGACCCCGGCTTGGTGCGGTCCCCCTCAGTGGTGCCCGACCAGAGACCGCGGAAAGAC
TCCAGGAAACGCCTCGGGGGGAAAGCAGGGCCGCGCACTTCCGCTCCACGCTAGCTGGGGGGACCACGACGGCAATACTGAACACCTGGCTCTTCGGC
AGCCCGGGCTATGAGGGGCCCTCTCGTCCCAGCCAAAGGCTGTTTGCCTTCCTGTCCGGGCACCCTCGACCGCACCCGGGCGGGCGCAGGCTTCATTGCG
GCGCGACTCGCCAAAAGTCCCCGCGATCTCAGCGAGTACCCTGAGTGCTGGCCCTAGTCCGCCTGCCCCCTTAATGTCGCTCAGGTCTGCAATCTCTTACG
ACCGGCTCGGGTAGTGGCCGCACGGTGCGTAGGTCCGATGCGGGCCGGGCCGCTCGCGCCGATCGTGCCGTGCGGCGGCCCTGACACCGTATGCGTGCG
CGCTCCGCAGGCTCGCGAAAATCCTTCGCAGGCACTTGGCCCCTCTGCTAGACGTGGTGTCTCCTAACTGTGGCTCACGCACGGCGGTTAGGCCACGGTA
GCCCGCTGTTCGGTCCTTACGACTCGGCACACGCCCCCGTGCACGCCCTCGGGTTCCGCAGGCCAACGCCTGGCCCTAGGGTTGCGTGCCGGCCTGCCCG
CGATGCCACTTGCCCCACGTCCCCCACCTGGCCGGCGCAGACGGGTTCAGTGGGTGTCGAACGCCATTCAGTCCCCTGGGACTCCCCCGACCCCTCCCAT
GGCACCCGCCGCCTGGCCTTGGAGCTCCTGCGCGTTGGACCTTACTGCGGTCGTCAGCTTCTCGGCGCGCCCCGGACTAGTCCTGGGCGGCCAGCTCGCG
GGACCCTAGCGCGCACGCGTGCCCGATTGCCGTATCCCCTCGACGGCGCCCGGGCATAGGGTTCGATCCCGCTGCTATTGCGGGTGCGTCCAGACCTGCC
GCCGTTTGGCGATCCGAGTGTGCGAAGGGAGCCAGCCAGCGGGCCGCCTGGCCGCACTCCAGGATCCTGGCGCCAAGGGAGAGCCCTGCCAGCGCTAC
GCGAATACTCGACCTGTCCGCGCCCCGCCTCGGGCTTGCGAGATGGCCACTAGACGCTACCGGGTCGTCACCCCGTCGGCCCCACAGGGGCCCGGCTTC
GCGTGGCTTTCTTGTCGAAGGCCCCGAAATCCGGCAGGCCCCGACACCTTCACCATGAGCGAGGACGCGCGGTGCGGGATGTTCAGTTCATGCACGCCC
CTCCACGGCCGCCTTCGCGCGGACAGCGTCGGGCGCGCCCAGGACGAGAACAGACCCTGGGCCAGCCTCAGGGCCCGGCCTAGGCGACCCTGGTGACC
AGTTTCCACTCGGCACCCTCGGGCTTCGTGGCAGTGCAGTCATGCCCCCGGGTGCTCCCGCGCGACCGCGCACGCCCCACCCAAGGTTCTTGGTCAACGC
GCCGGATCTGACGTCGCGGTCCGGCCCGCGCCGTAGGCAAAAATGGGTTCGGCTCCCGCCACTCCGCCAGTTCGAGGGCGGTAAAGATCTCAGCCGCCG
TGTTTCGGGCTATCTCCCCGCGATCCCGGGCCACAATCGCAGGTGGCTGGGGCGTCGGCCAGACCCGTCTCGCCGGCCTTTCTGCGCACATTACCTCCGA
GCAAGGCAACCGCTCCGCCCCATTACAGGGGGTTGTCTGGTACCTCCGTCACGTACCCCCGCATCGCTGGCGGGCTGACCCAAGCCCCCTCCCATCCCGA
CCCTCACGCGCTCACGCCCCTTGCCGGGCGGGGTCGTGGCTGCCCAGGGTCCAGGGCCCGCCCCGGGGGCCGGTTGGTTCCAGGAACTGAGGGAGGGTC
GCCCAGCTGACCGCTCCTCTCGCGGGGTCTATTCGGCGTGGCCATCCGCAGGTGCGACAATGCCGGCTCCCATTGCCGGGCCGACCTTGGTCCCTGGCGC

TTCGGACTCCCGGGGAGCGCCTCCCTTCATCGGCGGCCCCTGCCCCCGTCATGACCCCCCTTACGCGCCGGTGGTAGCCGCGCGTGGTGGCGTGCCCCTCT
CCATCATTACGCTCCACAGGAGTGGCCACCGGTATGGGCGCGGTGCAGTCGTACATGTGTGCGGCCCTGTTTTGCGAACCCTTCCCGCGGTGACCTCCGC
CCCAGCGGCCTTCTTCGACCGTTAACGCCGGCCAGCACCCGAAGGGGGTCACACAAAGCCCTGCAAGGATGCGACGCTGCGTAGCCGCAGGTTAGCCGC
TTCCTTCCCAAAACCGTGTCAGAGCTGTCCGGCGGAACGTTAACGCCGCCGGGAGGATCTGCGGCTACCCTCAGGCCCGTCCGGTGGGAGTGGCCGGCG
TCGATGGGGGTACGCACCCCACTAGACGCGGATTTTGGCCAACGGGCACGGCCGCTTCCGAGTTCCCCTGCGGTCTGAGTGTTGGTACGTTCAGCCGGCT
GTTCCGTGGGGCAGAGCGCCGCACGCATGCCGCAGGAGCGCGCCCTCCGCGTCTTGCGGGCGGGGTCCTAACGAAGCCCCCTGGGCTTGGAGGTAGGGG
GCGCGGGGGAGCGATGGGAGCGCAAGCGTGCCATCGGGTCCGGCCCATAAGTGTGTATGGCGCACCGCGCTGCCGCGATGTCCGGTGCGCGGGCGCCG
CTGGCTAGCTCACTCTCCCCCGACTCCGCCGCCGCGCCCCGAGACGTATGGAACAGCTGGCGCGCGTCCCCCGCCGCGGGCGGCAACGAACTAGATCCA
CCGCCCTCACGAGGAAGCGCGCCGCCACCACGTCTCCCCGGCCGACGCGGTCCCACCATCCGCTGTGCCGGCGCGCGAGCAGCCTGGCCCTCGGGGCGG
TCGCGCCAGCGCAGACACCCTGTCACGCGCACGGGAGCCCTGGCCACCGCGTCGACGCCCGGGCTAGCCCCGACTATCAGTACTGACCAGAGGGGCGC
CGGTCGTCCCGACTGGTTCCTCCCGAAACGCTGGCGGTGGGCCCGCACGATTGGTGCAGGCCCCCAAGAGGATACGGCTATTCGCGGCTGCCCGAGGCG
AGTCGGGCCAGACCGGTCGCGCGTGTGTGTGTTAGCCCTCCGTCGCGGCCAAGCCGGCGGCACCGGACTGGGCGACAGTAGCGTTCCTCTGTCACTAGTC
ACTCTAACGGACAAATGGAAAAATGGCACCTATCGCTACCGACGGGGTTTTCCTCCAGGATAAGCCTCCGCGCCCCCCACTGGACGCCAGCCATCCTTCC
CCGAACGGCTCGCCTGTGCCGGCTCCAACCCCGCGGGACCTCCCTTCGCGGCCCCGGCGGGGATCGGTGGACCGTCGCCGCCGGCGCCTACCGATCGCG
CCGCCACGGTCGTGCACCAACCAAGCACTGCTACACGCCTGTGCGGCCGAGGCCGCCCCATCCAGCAACGGGGCACCGTTGACCCGTCCCCCATGATGC
CGGACATGGTAGCCCCCACACATGCATCTTCGGGTAACCCGCGCCAGGTCCCCCCCCGCGCCGCCGCCGGCTTTCCCCCCGCTGGTGCTAAGCGGCAGAT
GCACCACTGACGGGACACACTCGCCTCCGATCCAGTGCGCTGCCGCCTGCGGCCCCCCACCGGACTGGTGCCTGGCGCTACACGGAGCCGGCCGCGCAC
TGTGACGTATGGATGCCCGAGGAGACATCCGCCCCGGACGCATGCGCAAAAGACCACTCGCTCGACGCCACGACGTGTGCGGCCGGGGTGATGAACGT
CGGCCGGTGTGCCAGTGAGGCTTGCACCTACACTCCCTGGTCCACCGGGCCGTTCGAGCGGCACCGGGCGAAGAACCCGCAGCCTCCCGGCTTACGCAC
CGGCCTCGCGTCAACAGGCAACCACGACCGCAAGGCCGGCAGTGTGTGTCGCTGTGGAAAGGTGCGCCGCAGGCGACCCCCACGTCAGCCCACTACAA
AGCGTGGCAGCATGCTGCGCAGCGACCGCACGCCCCGACACAGCCCAATGCACAGCATCGGGGGGGCGGAATCGCCCCACGCCCGTAACATCCCGCGG
GTCGACCGCCTTACACGTCGACTGCCACACCCTGGTGCCGCAGTGGTATCCCGAGACCGCATGACCCGCCGCGAGCCCGCTGACCACCTGTCCTTGATCG
CAGTCGCCACGGGGCCGGAAGGTGTACCGCATACCGCACGTGCTGCGCGTGTGCAGCACGGGGGCACCCTGCATGTATGGATCCCTCTAAGCCGACCTG
CCGCCCCAGGCCAGAGGCTAGAGTCTGCCGCCTAGGCCCCCCCCCGTGGTCGCCAGCCCCGGTGGCGGCCCACCCTCGGGGCGACTGACCGGCCACCAT
CCAGGGTAGAGAGGTTTGGCCCTAAATACGGGCGTGCCGAGGAGGTGTTTGGGGCGAGCCGCAGCAGGTTCGGCGGATCCTTGAGGGTGGGGCCTGGGG
TGGGACGGGGGCGGCCGAAACGCGAGCATAGGCCCCTGCCTGGCCCCTTTGCAGTCTGTACTTTTATTGACATGATACTAATACCGTGTTCCGCCCTGAG

ATCGCACGCGGACCTACCGTGCCTCTCTCTGCATAGTGCTTCAAGAGCCCAGCGCGACTGGGGACCGGCTCCCGCAGCCGGCTCGGGGCGTAACGCTCG
GTGGAAGGGTCACCCGGATAGCTCCCGGTTAGTTTCGTACAGGGTGTCGGGAGCGCCCCCAGCGAGTCTCCTGGAGGTGCAGGGACGGCGGTGGTCCTC
GAGGGGGCGGACGTTGGCCGCATAACGGGTAGTCTCCAGGGTCACCTATAGTCTGGCTCGCCGTCAGGGTCTGTGAGCGTCGTGCTCACGCCTCCGCAAC
AGCCGGCGACCCCGGCTCGCAACGGCCAGCCGTGGGCGGGGGGGCGACCGGACGCAGTTCCGAGTCTAGGCATGGTGCCACCGCCCTCTCGTTGGGTTA
GTCTCGAATGGCTGTTGCGGGGGTCAGCGCGGCGGGGCCGCTGCTGGGAGCCCGCATAACCGTGGATACACTTGCTGGGTGCGCGCTCGAGGGCGGCCC
CCGAGGGCTGGAAAACCATTTCGGGATCGCCCCCGCGCACCCGTCCGCACTGCGGGGTCCAGCCTGGCGGGGGCGGGTTTCGTTGGGGCAGTACTCACG
CCCACGGCGAGGCCCATCCAGAGAGTGAGGGTGTCGCTGGGGGGGCCTACCGGGCGGAACGCGCCCCGACTTAGGGCGGCGCAGTGCACGACCAGTCA
CCGGCTGGGCGTCGCCGCGCCGTTGAGAACCCCAACACAGAAAGAGCTTGCCGCCTCTGGGGGAGCCAGGCCGAGAAGCGTGGGCGAGCGGCCACGCT
GCGGGGCCTCCGCGGACCGTGCGGCGCTCTCGGGTGACAAACCTAGTCCCCGTCCCAGGCCATGGGCGAGCAGCTACGCCGGGACTGTGCGACGCCTAT
TCGGCACATTCGGTATCATTGGCCTGGGACGACAGCCGGCGACAGGGCTGACGAGGCGGCGTGGACTCGGGGTGCAGGGACGACGTCCACCCGTACTG
GTCAGTAGCCCAACGCGGAAGCCTGTCTGGGGCTTTGGCCCCTAGACATCGGTCCGGGGCGGGGCGCGTCATCGCTACGTTGGTGAGGGGGAGTCTGCA
CCGATAGGGTGTGGTGGTGTTGCTTAGATCGTTGACGGGCCGTGAGGTGGCGCTGCTGTTTTCCGGCTTACGGCTTACGACCTACCGCCCGCCGCGCCGGC
CCGAAGGCGGCGCCCCCGCACATCGCCCTTGGGTGGTAAAACCCGCTCTGCCGCCGCTACCCCCCCCCCAGGGGTGGCCGGAACGGCGAGGGCAGGAC
ACGGTAGACGCACGCAGCGCCGCGACGCCCGGCCCGCGCTAGGGTTGGCACCCGTGGGACCTCGGCAAACTTTCCGTACGATTGGGGGGCGCATACAG
CGGACGGGGAACCCGCCCGACGCACGCGCGGTCCCGAGGGGTGGGGGACCTGCAGCTGCGTCGCGCGGTCCGCGATGGCGTCTTGCTAGACCATGGGC
GTTGCAATAGTGGGAAGGTGCCTCCCAGGCTGCGGGGCGCCGGACGCCCGACACCAAAGCTGTGCGAGGACAGAGAGGCTAGGCACGGATGGGTCCAA
GCGAGCTGACAGCCTCCATCCTTGAGTGAGCACGCCGCGGCGAAATGCCGGCCGAAATGGGAGGGGCGCGACACCGGATCGTGATCGAGCGCGTGCGC
TTCGCCCACACGCCCTGACCCGCTGAGTGTCTCGGGTTTGAACCCCGTGCCCGGGGGCGCAGTGGGATAGGCACGACGGGCAGAGTTAGGGGCCTGGGC
ATAACGGCCCTGTCCGCGGCCGAGCGGGCCCGGGTCTTCTCGACCAGGGTGCCGGCCCCGGCGGGCCCCCCACCCACGGACGGGGACCTGGAGTTGAGC
CAGGCCACAGGCGTGACCGGAATTGGAGCGCCAGGGGACAGCGGCCGCCCGCGCCAGGCCACGCCGGTGGGGGCGTTCTGCCCAGGCGTCGCGCAATG
GGCGGACCGGTCTACGCGACACCGCCCGCCCGGCCTGGAACTCCCCCCGAGCACATTCGATATGGTCCGCATCAAGGCGGGAGGCATCGCCGGGGGCC
GCGCGGACCACGACCTTCGAGGGGGACGGTGCGGGTCGCGACGAGACGGCGGCATGTGCGCGGGTGCCGTGAGCCCTAGAGTCAGCGGTTGCTGGTAT
GGGCGGACGGGCGCCCCCGACCTGCCGTGGACGCCGGCTCGCGGACTAGTCGACCCGCTATGGGTGCTGTGCGTGACGTTGGCCTTGGTCCAGGGCGCG
GCGCGCAATACCGCGCGGGCTGAGTATCGCGTCCACGGACAGGCGGCTGAGCCGCCCGGCGTAGCTGGTACACGGTCGGGCCGAAGTGGCAAAGAGCC
GGCTTGCGCCCCCAGCGCCGTTGCAGCAGGTCTGGACAGGCAGTACGGAGCGAACGCGGCGAGACACCCGGCTCCGGGCGTAGTCGGATCCGCCCGCC
AGCGGGCGCGCAGCGGTGTCGCGGTCTTGATGCGCCGGTCAGGGAGGTCGTCGCGAATGAGCCGGGTGGAGGGTGCAAGAAGCCAGACGATGGGAGCA

CCGAGTTGTCCGCGTGCGATTGCCGTCCCGGCGAAAGCGCCAGGCCGCGGCGCGTGTGGCAACGGCAAGTACCCGGAAACACGGCACGGGTCTCGTGG
CGCCGCGAGGCGGCGGGTGGCGCGCTCCGGGGGGCTGCGCAGCCCCTCAGCGCCGCTGCTAGTGCCAGACGCTTGCCCTTCTTGGGCGCGGCGGCGGGA
ACTCGGTCGCGTGCGTTCAGGGCCCTCATCCGCAGACGTGAGGGGTGCGGGCCAGGGGGCGCAGAGGCGCCTTCCGAAACCCGTAAGGGATGTTCTGGC
GTTGGGGGGCCGCCGTCTTAGGGGGGTAGCGCCGCCTCAACTCCAGTATCGCCGCAAGGCCGGGGCCAAGTTCGATCCGGGGGCGGCGGCGTACCGCGT
CCACGCGTCTCCCAGAGGAGGGTCAAGGTGCATACGGGAGGGATCATACGGCAATCGGCGGCCGGGTTCCGCCATCTAACGTGGCGATCGCAGCGTACC
AGAGCGAGGCCTATGGGCCATATCCCTGGCGGGAACGGGCTGCTTGCGCCCCGTCCTCGCCGACCCTCCGTACCGGTACGGGGCGAGGATCCCGACACA
CAGCACGGGGGCGGCGCGCAGCACCCACCCCGTCCTCGGCCCAACAGACAAAGACCCCGCGGCGGGCTTGGGCACCCCCTTTCCGGCTACGTCCACCG
ATTCCGTACAGGACAGCCGTTGCGATGGGCACCGGCGATCGGAGAGAAGGACGCCACAGATCAGCAGACGACTCGGGGAAACCACGGGACCGCGCGT
CGCGTCAACTGCCGCGGGAGTGAGAAGGGAGCGACAGGAGCGCGGAGGTAAGGCCAATGGGGCCAGTTGAAGCCGCCCACAGAGGCTGTGAGGGGCC
CCGCCAGGTTTAGGAGCTGTTCTTGGAGCCTGTCGTTAGGGGCGGGGGGGGGCTTACACGGCGCTGGGGGACCCACGGGGCGCAATCCGCCGCGGACTG
CTGGAGGGCTGCCTGGGGCAGCACCCCAGGGGCGGCCATGGCGCTTGTTGACGGGGGCGCTAGGACCTTGTCCTAGGGCGCGCCGCTTTCGCCCACAGG
GCTTCGGTGGGCAGTCTATCTGTGCGGTTTCAGTCTCGTACGCACGTGATGCGGTGTAACCTGAGCCCGGCAGTTGTCTAGAGTAACTAGCGCGCGGTTAT
GGCTTCGTCTAGAAACGGGCTGCAATGTTCCGGGTGTTCATCACACCACGTTCCGCTTTGCAGCTCACCCCCCGACCACACCCGGATTCGGACCGGTGGT
CCTTCCTCTGAGGTACTCGCCGCTCATACAACCGCGGTTCCCGCTGTGGTCGGCACGGCCCGGGCTGCAGGGGCGTGCCTTCACGGGTAGTGTGCCTGCG
CGGCGTGCGGCTTCCGCAGTCCACGGACCGTTCCGGGGGTGAACGATGGAGGTGAGGGGACGAGTCCCTCCGAGATTAATGCCCCGCGGCAAGCCCGAT
CAGCGCTGCGGTCCTGGGCTGTCAGCGCGCGCGGAGGAGCGCCCGATCGGGCGCGGGGGAATGAGAGTGTGGAGGATCATAATAGAGAGATCGCCCCG
GAGACTGCGAGGGGCATGGACGCGGAGGGACGGGGCGGGGGGTCGGCACGCCCGTGCGGCCTGTCGTCATATCCCACGCTGTCACTCGGCTGCGTCCGG
CACTCCGGGCGGCCGGCGAGCAAAGCCGCCCTGCGCGGCCCGATCGAGGGTGCGGCGGCGCGACGTCGTGGCGTGGGAAATGCGGCGAGGGGCGGGGT
GGGGGACGTCATTGAACCTGCAGCGCGCACGGTGAGTACCGCGGCCCGTGGCGGGGCTGTACTGCGGGACCCCGGAGCGGTAGCGGGGGTTAGACTGG
ATGAGGGTAACCTGCTGCCCCGCGCACCACGTTCTTGGCCAGAGGCCCTGTGGGTGTTGGAGCAGGGTCGAGTATCCCTCCGTGCTTGCCTGCGTGGAGT
GGTTCGCCATGCAGTTGGACTCTTGGCGTCAGCTGGGGCCAGGCGGCGCCTCCGAGGTCGTAAAGGCCAACAGCGCTGTGAGATGGGCGCAGGAAGAG
CGCCAAAGACCTCCTGCTCCCCGATGTCCGCCTCGGGCGGACGTTCGGCCGCCTCGGGACTAGTTGAAGTGCGGCTTGGCTGGGGGCGCGGGACTCGGA
AGTCCGGGTGGAGGATGCGGGGAAGCTGCCCGTCGCCGGTACTGAGGGACTGTAGCTGTGATCCGGGACCACGAGGCATGCACCCGTCGTGGTCCAGCC
GGCCTGGCGCGGGGTCACAGAGCGAAACGGCGGACGCTAGGAACATTCGCTACCACCCCCCGGCGGCACCTTGCAGGGTCTACCCCAGTAAGGACCGG
CCCCAGCAGTGAGTGCGTCCGGGGGCTGGCAGCGGTAGATTGGGTCGCACGAGCTCACGTGGGCCGTTGACCGGAACCGGCAACGCCTGGGACTGGAG
CGCAAACTGGGGTACACTCTTCACGCCCGCTGCTCGTCATCGACCCCGGCGGGCGCCCGGGACCGGACCGGGGGCGACCCACCGGGGGACGGCGCGGC

CCCTGGATGGCCCTGCCCTCGGTCACGAAGCGGCCGAATGGTTTAGGGGGCCTCGGGACGCGGCCGTGGAGGTGCGGGGGGCCTGAAGTCCCTGCCTGG
CCCTCCTCCTAACGGCTTCACGCCCCTCAGGGCAGCTAGGGATTAACGTGGCCGGGTCACACGACCCGCCCAGCGCGGTACGCTGGGCCGAAGTGATTG
CGCGCGCCTGGCGGGTCCTCACCCCCCCACACACATCCGGGTCGGGGCGGAACTGGGTCCCGCCGTTCCCTCAGATCTGCCCCCCGACTTGAACATGCCC
AAAAACCGAAACCGGGCCGGCGCCCAGACCCCCGTGCTAAGGTTCCCACCGCCGACCCTAGCCCCCCCGGAGGTCCCCTGTACTCCGCACGCGACGTCG
TATCAGGTCCCAGAGCCCGACAATGCATGCGCGTCCCGCTCTGCGCCATGCCCATTGCCCGACCAGCCCCGCCCAGCGCAGAGGCCCCGACTGCTGGCG
CCCTCTGTGGCGCTCGGCCGCTCGAGAGCACCTGCCGCCCGACCGCAGTCTGGCTACCTGCTCCACGGGGCGGCGCTCGTGGTCTCCCGTACTCGGAACG
CCACGCCCCGGGCTCGCGCGTCTCGCGGCCCGATGCATCGTGGTCTTACCTCCGACCCTTTCCTGGGGTCGAGCGCGGGGGGGCAGAGCGCGCTCATCCA
GCCCCGCGCGGCAGCACCCGCCCGCCCAGGGTGGTACCGCTCTCGAGAGTCCGGACGGCCGAACGCCAGGGCAACGGGCCGTGTGAAGACTCACCGTT
TCGGCCGACCGCCATCCTAGCTGGGGCGAGCCCCCGCGCGCGGCCGGATGCGTATGACGTGCGCGCGCTCGGTGCGATGGCGCACGTCGACTGGAATAA
CCCCTTGCCAACCCCAGCGCCGAGAGTCCCTCTTCGGGTCTCCCGTCTGCCTCCGATGGGTGGCCCCTGCCGGTACTTGTTACGTCCTGGCTCGCCGGGCG
CCTCGTCACCTCGGCAGCCTGGATCGCTTGTCGCCGCCGCAAACGTGCGCACGTTTCGCCACGGGCCGCCCCGCGAAAAGCGGCGGGAACGTGGACCAC
GCGCGCGCCCGCCATCGGCTCGCCGGGATCCCCACCGCGGCAGCGCCCCGCCACAGCTCAGGACGCGGCCGATCGACAAGCACATCTCCCGACTACCG
GGCCCCGCGGCGTGAGCGTGGTCATGGAAAACCCGAGCGTGTGCGTTCCTGTCACGGGATCCCGCCGTGGGCAGTTCGCGGCAGGGGCCTACCCGTCGA
GGGGCTACACCGGACCGCCCCTCCAGCTGCATCGTGCAGCCCTAGTGCTGCAGCGAACTTGCCCCGGCCATCCCGTCCGCCCGCAAGCGGCTGGGGACA
GGTTCTCCGCCCATGCGCAGCCAGTGGCCTCATTCATGAGGGCGTGGCGGGCCGGGCCGCGTTGGGCCATCGCCGGTTGGGGGCCCCCACGCCGTCTACC
TGAGTGGACGCGCCGGGCGAGGCTCCCACCACCCCAGTCCTGGGGTCCCAGACGGCCTCCTGGGCGGAGTGTTCTACCCCGCGGCCACCCCCAGCCCGT
CTGCGGCCGAGGCCCCGCCGGAGACTGCATTCCCCGCGGCCGGGGAACCGGCCACACCGGCAACGGCGCTCCTGACCCCACCCGCCTGCCACACTCCCC
GCGGCGCGGAGCCCCACCCATACTAAGGCCCCTACGCGCGGCCGACGACATGGTGATGGCTTCGCTGGGGGGGCGGTGACACGTCCACGAGGCGCCTC
GCCGCCGTAGGCCCTGCCCCAGCACGTTCCCCCGTCCCCCGGGGGTGCGGCGTCCTGCTCGCCCCCCGTGTAGGAACGCCGACCGGGCTGCGTCGTCGGT
GAGGCAGTGGCCCGCTACGCGGCTCCTCTTCGCACGTGCTTTTGGCTGAAGAACGGGCACCCCCACCATTGCGCGGTTGGGACGGCGCGCGGCTGCGAG
GGCCCAAATCTCTCCCATGCGAGTCGTGGTCGCGCCCGTGGACCTCTGATGGGCAAGGCCGCTCGTCTGCTGAGCGGGGATGTTGCGCCCAGCAAATCGA
GGAAGGTTTGCTCGTAATTTACGGGTACCGAGACTCCTCGCTTGGCTCACCCATTGCGGCAGCCGAGGCCGCCGCCGGGACCAGTAACGGCGTGTCGACC
GCCCCTCGACAACGCCTGGTAGGACGCTCCCCTCCCGGCCCGGACACTAACCCCCGCAACAGCGAATTCCAGGGGCGTCTTCCGCCCAACAAAGACCCA
TCGTGCACGAATTGGGCTGGCGGGCAAGCATGTGCATCACGGACTAGGGTACCGCCTGCCTCCCCGCTGCCTCCGGGGCCCTGGCGCTCCGTCCGATTTT
ATCCCGCTCTGCTGCGATACTCAACCAACCGTTTCGCTTTATTCAGGCCGCCTCTGGGGCACGGCCGAACACTTTCTCCCGACAAGTGGCGTGACGGACCT
GCCTTCGCGCCGCCCCGAAACAGGCGGGCCGGCCTCGCACCCCTGGGGAGCGGTCCGCCGCAGGCGGGCCTCCTGAGCGCGCGGGTGGCCGGCCAGCG

CGCATACCCCCTCGTCGCTGCCGCCCCCCCCGCCCCGATCCCCGGCCCCGCTCGGCAAGACAGTGGAACCCGCCGCGCCCTTCACTCGCCCGGCTGGCCG
TATTTCGTGGGTCCTCACCTCAGTCGCGTCTGACAGATTAGACCTCGCCGCTGATGCCTAGGGTGGGACACCACCTTCCCGCCCGCCACCACCCTGTGGA
GCGTGAGCGCACTTAAGTCTCCCTACCCCCCCAACTCAGCAGCGGCCCCAGGGCATCCGCGAGGCTCCGTTGACGCACGTGCCGTCGTCCCGCGCCCACT
GTGGCATCTCGGCGGCGACCCTCCGCTGCGGGATGCCTGGAGGAGCGGTCCAGCCTGGGCGCTCTGCCGGTCACCAACGGCGGCCGGGCGGGGGCCCTC
GCGGTCGGACCGAACTTCGGAGGGGTCCCCGGCAGCAGAGTCGAGCGCTGCTAGTGACCTCCACCTCGGCCTAGTCACACTTGCCCGGCTCCCAACCGC
TAAGACATAGGGGGGCGTCGCGACGAGGGCAATGCGGGGGCAACCTTGCCACCCCCAAGCGACGTCGCCTCATATAGGTTGGAGGGGCTGTCTTGTACTC
CACCGAGGGCGACACGTAGCGTGCGGCGGCTCGACTCGAATCTTGCGCCTTGGTACGCGCCACGCGACCGATGACCTTTGCAGGCGACTCTGCGAGAAT
GGGGCCCGCGTCCTGGGGCGTGCTAAATCACGACCGCCGCCGCTGGCCTCCAGGAGGTCCCAGGGGGCAGCACACGCCATTCGCGTCGGTGGTCTAGCC
TCGCGGCACTTCGACGAGTCCCAGGACGCGTCGGGCCCGGTTCTTCCTCTTCCTGTGTAGCAGTCCGGCCGGGTGACGCTTCCGCCGCTGGCTAATTCAGA
CCGCCGTCAGTCGACTGGGCTGCCCTAACCAGCCAGGGCACTCGTGGACTTCCGGCGCCGTCCGACTCCACGCGGCGCGCACAGTCGCCTCCCTCCTTCG
TACCATTCGGCCCTGCTCACCCCAGGGGGGGCTTGCCGCCATTCCCTACGCTGCCATTCGACGTCCCGCGCTACTCACCCATAGTGCCAATTGCGGCGTCG
CGGACACCGGCCTGGCGCAGCGCCAGAGCGCCACCTGTGACGTCGCTCACCGCGGGGGGAGCCCAGCCTCGACGCCCCAGATGGCTCGCGGGGGGGA
GGTCCCGAGGCCACCCTTCCAGGTGGCACAGATGCCCCTCCCTGGAGGGGGTGGAAAGCGCGCGGCGGGCCCAACACCGTGGGCGGCAGCTGGGCATG
CGCTCGGATAAGAGCGCCAGGATGGCAGGAGGGCCGATCTGGCGGGTGGTGGCCAGTGCGGGGCGGAGGGCGAAACTGTCTGGGGGTACCTGGAGCCC
AGGATTCAGGGTCCGTGCCATGAGGCTGTCAGGCAAGCCAAGCCGTTAACAGCGGCCAGCGAGTGGGCAGGGAAGGTGACTGGCGCGAACTACATATT
GCCGGCCGCCGCGCGGGGCGGCCAGCCGTGAGGAGCAATCTGAGGGACGGCCCACCGCCGGATGGCCTGCACCATGAGGTGAGGCGGCGGGTCCCCGC
CACGCCGCGAGACGGTACGACCCGTTCCGGATGTGCACCACCGATGAAGAAGGCGCGCACATGCCGTTCGCTTTCGGCGTATGGTCTTCGCAGTCTCCGG
TGGGAGGCGCAGCCCGGAGGGTGCCCGCGCCACACGGATCAATGGGTTGGAGGGGCGGACCCGTGGTTTCGACCATTGGTGCAGCTAGAAACCGTCCC
AAGGCTCCACTCGATCGCACTGCCGTCGATCTCGGTTCTGACTCCCACCTATGCCAACACGGGAGTGCGCTATGAAACGCGGTTTTATAGTCGGTAATCG
CGACCGAACGCGGAGCGCCGTCTCGAGGCTTGTAACGCTCCGCGGTCGTTGTGGAGCGGACCGGGTAGTCAGCGTGACGTATTTCTAGCCCCCAGTGTGC
CGCCCGGCGAGAAAGTCCTCGCGGGAGCCCGTCGGCTGCCGCTGCGTACGCGGGGGAAAGCGGCCGAGGTACGTCTTCCTGGTGGCTTGCTGCCGCCAA
CCACCGATCGGCCCGGCTCGATGACAGCCGGCGAGAATGGGTTGGGGCCTACTTGGCCGGGTTGGCTGATCGTACGCAAGCAGCGTGCTGCGGGCCCAC
GCAAAGGGGAATGGCGGGGGAGCGCCGGCTGCCAATTCCCCCCGGCTGCACAGGCATGATCCGGTGTCAAGAACGGTCTCCGGCCCACGCGGCCGCGC
CTTGGCCCGAGTACGGCTGACTCAGAAGCCGTTGGGTGCGAGGGCGTGGAGTAGTGGCGCATAGGGAGCCAGACCCGACGGAGCCGGGATGGTTCGAT
CAGTGTCACGGCCCGGGGTCATGTGGGGCCCGCCCGCACGGATGACCCGCGTCTCGATAATCGGTTGCAGGCGTGCCGTTCCCACGGGGCGCGCCGGTT
ATCGAACGGGTGCGCGCGTAGACTCGCTTCCGGGCCCCGGACCTGGGCCAGGCAGAAGCGGGGAATACCCGTCTTGATCGCAGACAGCCCGGTGCCCCC

GGGGGCCGACGTACAGCTGGCAAGCACCGGTCCGGCGATTACCCGACCTGGGTTGGCAGGTACACGTTACAGGGGTGGCGTGCCCCCCGGCGCTTGGCG

CCGGCTTGGAGGGACGGGGGGGGATCTAGCTCAGATGGCCAGCGAGCGTAGCTTCGACGGTGGCAAGAGTGAGCGGCGGCGTGCTTGGGGAGGCCGCG

GGCGGGTGCGACGTCCAGTTCGTCACACGCATGAGGGGTTTCCGGGGAGGAATCACCGCCTCGCGCAGGCGTGTCCGACACGACCCGTGTAGCGGGTGT

GAGACATTTAGGCGGAGGCCAGAACGCCTTGCCGGAGGGCTTGTAGTATATTGTCAATGGGGCCGACACCACGTGCGGATCGGGACTGAGAGCGCGTAG

AGCCTTGGCCCTTGCTATATTTGTGGAGCGGACACTGTGCTTCCCACACGTCACGCTGGCGTAAGCCGATGCCCGTCACCGCCCGCGCCCCAGGCCGAGT

GCGGGGGGCCGCAAGGTCCTGAAGCCGATCTACGATGGGCTGGGTGGGCGACGTCGTGGGCTAAGCTCGGCGGCCCCACCGTGAGGCGGACGTGGGGG

CCTGTATTAGAGATGGAGCGACGGTCGTCGATACAGTTCTTGCCAGGAGACCGAGCATTTTGCTAGGCACGGGCTGCATGGGCAATTCATTAACGGCGGC

GGGCCCACCGCGGCCAGCGGTGGCGGGGCGTAGTACCGGTCGGCAGGGATACGACCCCGGACCGAAATTCGGGGCCGGCGGCCAGGGGGCGGCGCGC

CTGCACGCCGTGACGCTGACGCCCTCCCGGGGTGCCGCGGGCTGGCCTGAGGCCCAAGGAGGCGCCTGGGGGTCGAGCGCGGGCCCGGGCGCACGCTG

ATCCCGAGCCGACGAGCTTACGGATTGACCAAGAGCACCACGAGCGTCCGAGCGCTGCGGGCGTGGTAACAACTACATCCCGGGTCGAGTTTGCGGCTG

GGACGTGGGCTTGGCCGTGCAGACGCGCTGGGGGGGGAGCGGGGCTGGCCCCTCGCTCCGTATTCCCCACTCCACATGGTCCCCGGGCGTACGGGCGTC

CCCGACGGTTCGGGACGCGGGAATCGCGCAGGTGCCCCTCGGTGCGTGGCGGGCTGCGTGTAAGGCGCTTTCGCTGCATGCGGCCGTCTCGCGGACATG

GCGTACGGGGGCGACCGGGGCGGATCCAAGGTGCGCCCAACGGGCCGAGGCGCTACAGATGGGGAGCCCCGGGCGGCCGCACGCAGGAGTCCAGTGG

CGTATGGAGGAAGGCCTGGGGCTCCGCAACGGCCGAGGCTATGCCCAGACCCCATGCCATTGTAGGCCGAACGCGATAGGTCACGCCGGTGATCCTGCC

GGGGTCGCGGGCCGGATCCACAAGGCCAGACCCATAGCGCAGTCTATAGGCGTCCGGGAAGGGTCGTGGGGGGGCTAA

>2017.TE.25009.1.13

CACGCCCCCCTCCCGGCCAGCCCGCCCCCCGCCCCAGCGTCGTACCCGTCGTCGCGACTCCCCGCCGTCGAGAGGCCTTTGCCGGGGCCCCGGCTAGCCC

GGGGCGAGCGGACCGCCGCGCGCGGGGGGTGTCACCCGGGGCACACCGCGCCAGCCTCCCGGGCATGCCACGCCGGGTTCCGCCCCCGCTGCTCCAATT

TGGGGGGCCCCTAAGATCCCGTTCCCCAGGCCAGCCGCGCCCGCGGGCCAACATTGCCACACCGTGTCGCCCCCCCGCCTGCCGCACTGACCGTCTTGCG

GGCCCTGCTCCCTGGCCCGAGCACACCTCCGGGGACCCCCCTCGCTCGGCCGGGGGAGCCTTAAGCGCGGCACCGAGTAGGGGGTCGCGTGTTGCCGCC

GCGCTGACGCCCCGGCTGGGACCCCTTTCAGGTCGAGACGCCGGGGTGGCCATGCGGGTCCAGTCGCACGATGGCGCGGCGATGTGGCGCCGACTGCCC

CGCCTGTCTTCTCCGTGCCAGCCCCTCGCCACCATGCCGGTCCGGCCCGCACGGGGGTAGGAGGTGCTGCCGCGGCGGGAGGCGTAGGACGCCATATCC

GAACCGGCGGGTCCCCCCGGCCACCGAGTCGACAGGACCATAGGCACCGCGTCCGGGCGGGCGCCCTTAACGCCGCCCCTTACTGGAGAAGCAGAGCT

CTGCCGAGGCACCCTGCCTAGCTGGCTGCGTCCCGCACCCCGCGATCCCCCTGGTCCACTTACCCAGCGGGATGCGGGGATGTTCGCAGCTATCACACGG

TCGTGGCCTGCCTCCCTGGGCCGGCGAGAGCACCGCCACCTTCGTGTTCCTTGCCGCCGGGCGACGTGTCCGGGAAAGGCTTCGCCGCGCGCCCGCGCGG
AGCGCCGGGGAGTCCCGTGAGCGGCCGCAGGAACGTCACGGGCCCCGGCTTGGTGCGGTCCCCCTCAGTGGTGCCCGACCAGAGACCGCGGAAAGACT
CCAGGAAACGCCTCGGGGGGAAAGCAGGGCCGCGCACTTCCGCTCCACGCTAGCTGGGGGGACCACGACGGCAATACTGAACACCTGGCTCTTCGGCA
GCCCGGCCTATGAGGGGCCCTCTCGTCCCGGCCAAAGGCTATTTGCCTTCCTGTCCGGGCACCCTCGACCGCACCCGGGCGGGCGCAGGCTTCATTGCGG
CGCGACTCGCCAAAAGTCCCCGCGATCTCAGCGAGTACCCTGAGCGCTGGCCCTAGTCCGCCTGCCCCCTTAATGTCGCTCAGGTCTGCAATCTCTTACG
ACCGGCTCAGGTAGTGGCCGCACGGTGCGTAAATCCGCTGCGGGCCGGGCCACTCGCGCCGATCGTGCCGTGCGGCGGCCCTGACACCGTATGCGTGCG
CGCTCCGCAGGCTCGCGAAAATCCTTCGCAGGCACTTGGCCCCGTTGCTAGACGTGGTGTCTCCTAACTGTGGCTCACGCACGGCGTTTAGGCCACGGTA
GCCCGCTGTTCGGTCCTTACGACTCGGCACACGCCCCCGTGCACGCCCTCGGGTTCCGCAGGCCAACGCCTGGCCCTAGGGTTGCGTGCCGGCCTGCCCG
CGATGCCACTTGCCCCACGTCCCCCACCTGGCCGGCGCAGACGGGTTCAGTGGGTGTCGAACGCCATTCAGTCCCCTGGGACTCCCCCGACCCCTCCCAT
GGCACCCGCCGCCTGGCCTTGGAGCTCCTGCGCGTTGGACCTTACTGCGGTCGTCAGCTTCTCGGCGCGCCCCGGACTAGTCCTGGGCGGCCAGCTCGCG
GGACCCTAGCGCGCACGCGTGCCCGATTGCCGTATCCCCTCGACGGCGCCCGGGCATAGGGTTCGATCCCGCTGCTATTGCGGGTGCGTCCAGACCTGCC
GCCGTTTGGCGATCCGAGTGTGCGAAGGGAGCCAGCCAGCGGGCCGCCTGGCCGCACTCCAGGATCCTGGCGCCAAGGGAGAGCCCTGCCAGCGCTAC
GCGAATACTCGACCTGTCCGCGCCCCGCCTCGGGCTTGCGAGATGGCCACTAGACGCTATCGGGTCGTCACCCCGTCGGCCCCACAGGGGCCCGGCTTCG
CGTGGCTTTCTTGTCGAAGGCCCCGAAATCCGGCAGGCCCCGACACCTTCACCGTGAGCGAGGACGCGCGGTGCGGGATGTCCAGTTCATGCACGCCCCT
CCACGGCCGCCTTCGCGCGGACAGCGTCGGGCGTGCCCAGGACGAGAACAGACCCTGGGCCAGCCTCAGGGCCCGGCCTAGGCGACCCTGGTGACCAG
TTTCCACTCGGCACCCTCGGGCTTCGTGGCAGTGCAGTCATGCCCCCGGGTGCTCCCGCGCGACCGCGCACCTCCCACCCAAGGTTCTTGGTCAACGCGC
CGGATCTGACGTCGCGGTCCGGCCCGCGCCGTAGGCAAAAATGGGTTCGGCTCCCGCCACTCCGCCAGTTCGAGGGCGGTAAAGATCTCAGCCGCCGTG
TTTCGGGCTATCTCCCCGCGATCCCGGGCCACAATCGCAGGTGGCTGGGGCGTCGGCCAGACCCGTCTCGTCGGCCTTTCTGCGCACATTACCTCCGAGC
AAGGCAACCGCTCCGCCCCATTACAGGGGGTTGTCTGGTACCTCCGTCACGTACCCCCGCATCGCTGGCGGGCTGACCCAAGCCCCCTCCCATCCCGACC
CTCACGCGCTCACGCCCCTTGCCGGGCGGGGTCGTGGCTGCCCAGGGTCCAGGGCCCGCCCCGGGGGCCGGTTGGTTCCAGGAACTGAGGGGGGGTCGC
CCAGCTGACCGCTCCTCTCGCGGGGTCTATTCGGCGTGGCCATCCGCAGGTGCGACAATGCCGGCTCCCATTGCCGGGCCGACCCTGGTCCCTGGCGCTT
CGGACTCCCGGGGAGCGCCTCCCTTCATCGGCGGCCCCTGCCCCCGTCATGACCCCCCTTACGCGCCGGTGGTAGCCGCGCGTGGTGGCGTGCCCCTCTC
CATCATTACGCTCCACAGGAGTGGCCACCGGTATGGGCGCGGTGCAGTCGTACATGTGTGCGGCCCTGTTTTGCGAACCCTTCCCGCGGTGACCTCCGCC
CCAGCGGCCTTCTTCGACCGTTAACGCCGGCCAGCACCCGAAGGGGGTCACACAAAGCCCTGCAAGGATGCGACGCTGCGTAGCCGCAGGTTAGCCGCT
TCCTTCCCAAAACAGTGTCAGAGCTGTCCGGCGGAACGTTAACGCCGCCGGGAGGATCTGCGGCTACCCTCAGGCCCGTCCCGTGGGAGTGGCCGGCGT
CGATGGGGGTACGCACCCCACTAGACGCGGATTTTGGCCAACGGGCACGGCCGCTTCCGAGTTCCCCTGCGGTCTGAGTGTTGGTACGTTCAGCCGGCCG

TTCCGTGGGGCAGAGCACCGCACGCATGTCGCAGGAGCGCGCCCTCCGCGTCTTGCGGGCGGGGTCCTAACGAAGCCCCCTGGGCTTGGAGGTAGGGGG
CGCGGGGGAGCGATGGGAGCGCAAGCGTGCCATCGGGTCCGGCCCATAAGTGTGTATGGCGCACCGCGCTGCCACGATGTCCGGTGCGCGGGCGCCGCT
GGCTAGCTCACTCTCCCCCGACTCCGCCGCCGCGCCCCGAGACGTATGGAACAGCTGGCGCGCGTCCCCCGCCGCGGGCGGCAACGAACTAGATCCACC
GCCCTCACGAGGAAGCGCGCCGCCACCACGTCTCCCCGGCCGACGCGGTCCCACCATCCGCTATGCCGGCGCGCGAGCAGCCTGGCCCTCGGGGCGGTC
GCGCCAGCGCAGACACCCTGTCACGCGCACGGGAGCCCTGGCCACCGCGTCGACGCCCGGGCTAGCCCCGACTATCAGTACTGACCAGAGGGGCGCCG
GTTGTCCCGACTGGTTCCTCCCGAAACGCTGGCGGTGGGCCCGCACGATTGGTGCAGGCCCCCAAGAGGATACGGCTATTCGCGGCTGCCCGAGGCGAG
TCGGGCCAGACCGGTCGCGCGTGTGTGTGTTAGCCCTCCGTCGCGGCCAAGCCGGCGGCACCGGACTGGGCGACAGTAGCGTTCCTCTGTCACTAGTCAC
TCTAACGGACAAATGGAAAAATGGCACCTATCGCTACCGACGGGGTTTTCCTCCAGGATAAGCCTCCGCGCCCCCCACTGGACGCCAGCCATCCTTCCCC
GAACGGCTCGCCTGTGCTGGCTCCAACCCCGCGGGACCTCCCTTCGCGGCCCCGGCGGGGATCGGTGGATCGTCGCCGCCGGCGCCTACCGATCGCGCC
GCCACGGTCGTGCACCAACCAAGCACTGCTACACGCCTGTGCGGCCGAGGCCGCCCCATCCAGCAACGGGGCACCGTTGACCCGTCCCCCATGATGCCG
GACATGGTAGCCCCCACACATGCATCTTCGGGTAACCCGCGCCAGGTCCCCCCCCGCGCCGCCGCCGGCTTTCCCCCCGCTGGTGCTAAGCGGCAGATGC
ACCACTGACGGGACACACTCGCCCCCGATCCAGTGCGCTGCCGCCTGCGGCCCCCCACCGGACTGGTGCCTGGCGCTACACGGAGCCGGCCGCGCACTG
TGACGTATGGATGCCCGAGGGGACATCCGCCCCGGACGCGTGCGCAAAAGACCACTCGCTCGACGCCACGACGTGTGCGGCCGGGGTGATGAACGTCG
GCCGGTGTGCCAGTGAGGCTTGCACCTACACTCCCTGGTCCACCGGGCCGTTCGAGCGGCACCGGGCGAAGAACCCGCAGCCTCCCGGCTTACGCACCG
GCCTCGCGTCAACAGGCAACCACGACCGCAGGGCCGGCAGTGTGTGTCGCTGTGGAAAGGTGCGCCGCAGGCGACCCCCACGTCAGCCCACTACAAAG
CGTGGCAGCATGCTGCGCAGCGACCGCACGCCCCGACACAGCCCAATGCACAGCATCGGGGGGGCGGAATCGCCCCACGCCCGTAACATCCCGCGGGT
CGACCGCCTTACACGTCGACTGCCACACCCTGGTGCCGCAGTGGTATCCCGAGACCGCATGACCCGCCGCGAGCCCGCTGACCACCTGTCCTTGATCGCA
GTCGCCACGGGGCCGGAAGGTGTACCGCATACCGCACGTGCTGCGCGTGTGCAGCACGGGGGCACCCTGCATGTATGGATCCCTCTAAGCCGACCTGCC
ACCCCAGGCCAGAGGCTAGAGTCTGCCGCCTAGGCCCCCCCCCGTGGTCGCCAGCCCCGGTGGCGGCCCACCCTCGGGGCGACTGACCGGCCACCATCC
AGGGTAGAGAGGTTTGGCCCTAAATACGGGCGTGCCGAGGAGGTGTTTGGGGCGAGCGGCAGCAGGTTCGGCGGATCCTTGAGGGTGGGGCCTGGGGTG
GGACGGGGGCGGCCGAAACGCGAGCATAGGCCCCTGCCTGGCCCCTTTGCAGTCTGTACTTTTATTGACATGATACTAATACCGTGTTCCGCCCTGAGAT
CGCACGCGGACCTACCGTGCCTCTCTCTGCATAGTGCTTCAAGAGCCCAGCGCGACTGGGGACCGGCTCCCGCAGCCGGCTCGGGGCGTAACGCTCGGC
GGAAGGGTCACCCGGATAGCTCCCGGTTAGTTTCGTACAGGGTGTCGGGGGCGCCCCCAGCGAGTCTCCTGGAGGTGCAGGGACGGCGGTGGTCCTCGA
GGGGGCGGACGTTGGCCGCATAACGGGTAGTCTCCAGGGTCACCTATGGTCTGGCTCGCCGTCAGGGTCTGTGAGCGTCGTGCTCACGCCTCCGCAACAG
CCGGCGACCCCGGCTCGCAACGGCCAGCCGTGGGCGGGGGGGCGACCGGACGCAGTTCCGAGTCTAGGCATGGTGCCACCGCCCTCTCGTTGGGTTAGT
CTCGAATGGCTGTTTCGGGGGTCAGCGCGGCGGGGCCGCCGCTGGGAGCCCGCATAACCGTGGATACACTTGCTGGGTGCGCGCTCGAGGGCGGCCCCC

GAGGGCTGGAAAACCATTTCGGGATCGCCCCCGCGCTCCCGTCCGCACTGCGGGGTCCAGCCCGACGGGGGCGGGTTTCGTTGGGGCAGTACTCACGCC
CACGGCGAGGCCCATCCAGAGAGTGAGGGTGTCGCTGGGGGGGCCTACCGGGCGGAACGCGCCCCGACTTAGGGCGCCGCAATGCACGACCAGTCACC
GTCTGGGCGTCGCCGCGCCGTTGAGAACCCCAACACAGAAAGAGCTTGCCGCCTCTGGGGGAGCCAGGCCGAGAAGCGTGGGCGAGCGGCCACGCTGC
GGGCCCTCCGCGGACCGTGCGGCGCTCTCGGGTGACAAACCTAGTCCCCGTCCCAGGCCATGGGCGAGCAGCTGCGCCGGGACTGTGCGACGCCTATCC
GGCACATTCGGTATTATTGGCCTGGGACGACAGCCGGCGACAGGGCGGACGAGGCGGCGTGGACTCGGGGTGCAGGGACGACGTCCACCCGTACTGGT
CAGTAGCCCAACGCGGAAGCCTGTCTGGGGCTTTGGCCCCTAGACATCGGTCCGGGGCGGGGCGCGTCATCGCTACGTTAGTGAGGGGGAGTCTGCACC
GATAGGGTGTGGTGGTGTTGCTTAGATCGTTGACGGGCCGTGAGGTGGCGCTGCTGTTTTCCGGCTTACGGCTTACGACCTACCGCCCGCCGCGCCGGCCC
GAAGGCGGCGCCCCCGCACATCGCCCTTGGGTGGTAAAACCCGCCCTGCCGCCGCTACCCCCCCCCCAGGGGTGGCCGGAACGGCGAGGGCAGGACAC
GGTCGACGCACGCAGCGCCGTGACGCCCGGCCCGCGCTAGGGTTGGCACCCGTGGGACCTCGACAAACTTTCCGTACGATTGGGGGGCGCATACAGCGG
ACGGGGAACCCGCCCGACGCACGCGCGGTCCCGAGGGGTGGGGGACCTGCAGCTGCGTCGCGCGGTCCGCGAGGGCGTCTTGCTAGACCATGGGCGTT
GCAATAGTGGGAAGGTGCCTCCCAGGCTGCGGGGCGCCGGACGCCCGACACCAAAGCTGTGCGAGGACAGAGAGGCTAGGCACGGATGGGTACAAGC
GAGCTGACAGCCTCCATCCTTGAGTGAGAACGCCGCGGCGAAATGCCGGCCGAAATGGGAGGGGCGCGACACCGGATCGTGATCGAGCGCGTGCGCTT
CGCCCACACGCCCTGACCCGCTGAGTGTCTCGGGTTTGAACCCCGTGCCCGGGGGCGCAGTGGGATAGGCACGACGGGCAGAGTTAGGGGCCTGGGCAT
AACGGCCCTGTCCGCGGCCGAGCGGGCCCGGGTCTTCTCGACCAGGGTGCCGGCCCCGGCGGGCCCCCCGCCCACGGACGGGGACCTGGAGTTGAGCC
AGGCCACAGGCGTGACCGGAATTGGAGCGCCAGGGGACAGCGGCCGCCCGCGCCAGGCCACGCCGATGGGGGCGTTCTGCCCAGGCGTCGCGCAATGG
GCGGACCGGTCTACGCGACACCGCCCGCCCGGCCTGGAACTCCCCCCGAGCACATTCGATATGGTCCGCATCAAGGCGGGAGGCATCGCCGGGGGCCG
CGCGGACCACCACCTTCGCGGGGGACGGTGCGGGTCGCGACGAGGCGGCGGCATGCGCGCGGGTGCCGTGAGCCCTAGAGTCAGCGGTTGCTGGTATG
GGCGGACGGGCGCCCCCGACCTGCCGTGGACGCAGGCTCGCGGACTAGTCGACCCGCCATGGGTGCTGTGCGTGACGTTGGCCTTGGTCCAGGGCGCGA
CGCGCAATACCGCGCGGGCTGAGTATCGCGTCCACGGACAGGCGGCTGAGCCGCCCGGCGTAGCTGGTACACGGTCGGGCCGAAGTGGCAAAGAGCCG
GCTTGCGCCCCCAGCGCCGTTGCAGCAGGTCTGGACAGGCAGTACGGAGCGAACGCGGCGAGACACCCGGCTCCGGGCGTAGTCGGATCCGCCCGCCA
GCGGGCGCGCAGCGGTGTCGCGGGCTTGATGCGCCGGTCAGGGAGGTCGTCGCGAATGAGCCGGGTGGAGGGTGCAAGAAGCCAGACGATGGGAGCAC
CGAGTTGTCCGCGTGCGATTGCCGTCCCGGCGAAAGCGCCAGGCCGCGGCGCGTGTGGCAACGGCAAGTACCCGGAAACACGGCACGGGTCTCGTGGC
GCCGCGAGGCGGCGGGTGGCGCGCTCCGGGGGGCTGCGCAGCCCCTCAGCGCCGCTGCTAGTGCCAGACGCTTGCCCTTCTTGAGCGCGGCGGCGGGAA
CTCGGTCGCGTGCGTTCAGGGCCCTCATCCGCAGACGTGAGGGGTGCGGGCCAGGGGGCGCAGAGGCGCCTTCCGAAACCCGTAAGGGATGTTCTGGCG
TTGGGGGGCCGCCGTCTTAGGGGGGTAGCGCCGCCTCAACTCCAGTATCGCCGCAAGGCCGGTGCCAGGTTCGATCCGGGGGCGGCGGCGTACCGCGTC
CACGCGTCTCCCAGAGGAGGGTCAAGGTGCATACGGGAGGGATCATACGGCAATCGGCGGCCGGGTTCCGCCATCTAACGTGGCGATCGCAGCGTACC

AGAGCGAGGCCTATGGGCCATATCCCTGGCGGGAACGGGCTGCTTGCGCCCCGTCCTCGCCGACCCTCCGTACCGGTACGGGGCGAGGATCCCGACACA
CAGCACGGGGGCGGCGCGCAGCACCCACCCCGTCCTCGGCCCAACAGACAAAGACCCCGCGGCGGGCTGGGGCACCCCCTTTCCGGCTACGTCCACCG
ATTCTGTACAGGACAGCCGTTGCGATGGGCACCGGCGATCGGAGAGAAGGACGCCACAGATCAGCAGACGACTCGGGGAAACCACGGGACCGCGCGTC
GCGTCAACTGCCGCGGGAGTGAGAAGGGAGCGACAGGAGCGCGGAGGTAAGGCCAATGGGGCCAGTTGAAGCCGCCCACAGAGGCTGTGCGGGGCCC
TGCCGGGTTTAGGAGCTGTTCTTGGAGCCTGTCGTTAGGGGCGGGGGGGGGCTTACACGGCGCTGGGGGACCCACGAGGCGCAATCCGCCGCGGACTGC
TGGAGGGCTACCTGGGGCAGCACCCCAGGGGCGGCCATGGCGCTTGTTGACGGGGGCGCTAGGACCTTGTCCTAGGGCGCGCCGCTTTCGCCCACAGGG
CTTCGGTGGGCAGTCTATCTGTGCGGTTTCAGTCTCGTACGCACGTGATGCGGTGTAGCCTGAGCCCGGCAGTTGTCTAGAGTAACTAGCGCGCGGTTATG
GCTTCGTCTGGAAACGGGCTGCAATGTTCCGGGTGTTCATCACACCACGTTCCGCTTTGCAGCTCACCCCCCGACCACACCCGGATTCGGACCGGTGGTC
CTTCCTCTGAGGTACTCGCCGCTCATACAACCGCGGTTCCCGCTGTGGTCGGCACGGCCCGGGTTGCAGGGGCGTGCCTTCCCGGGTAGTGTGCCTGCGC
GGCGTGCGGCTTCCGCAGTCCACGGACCGTTCCGGGGGTGAACGATGGAGGTGAGGGGACGAGTCCCTCCGAGATTAATGCCCCGCGGCAAGCCCGATC
AGCGCTGCGGTCATGGGCTGTCAGCGCGCGCGGAGGAGCGCCCGATCGGGCGCGGGGGAATGAGAGTGTGGAGGATCATAATAGAGAGATCGCCCCGG
AGACTGCGAGGGGCATGGACGCGGAGGGACGGGGCGGGGGGTCGGCACGCCCGTGCGGCCTGTCGTCATATCCCACGCTGTCACTCGGCTGCGTCCGGC
ACTCCGGGCGGCCGGCGAGCAAAGCCGCCCTGCGCGGCCCGATCGAGGGTGCGGCGGCGCGACGTCGTGGCGTGGGAAATGCGGCGAGGGGCGGGGT
GGGGGACGTCATTGAACCTGCAGCGCGCACGGTGAGTACCGCGGCCCGTGGCGGGGCTGTACTGCGGGACCCCGGAGCGGTAGCGGGGGTTAGACTGG
GTGAGGGTAACCTGCTGCCCCGCGCACCACGTTCTTGGCCAGAGGCCCTGTGGGTGTTGGAGCAGGGTCGAGTATCCCTCCGTGCTTGCCTGTGTGGAGT
GGTTCGCCATGCAGTTGGACTCTTGGCGTCAGCTGGGGCCAGGCGGCGCCTCCGAGGTCGTAAAGGTCAACAGCGCTGTGAGATGGGCGCAGGAAGAGC
GCCAAAGACCTCCTGCGCCCCGATGTCCGCCTCGGGCGGACGTTCGGCCGCCTCGGGACTAGTTAAAGTGCGGCTTGGCTGGGGGCGCGGGACTCGGAA
GTCCGGGTGGAAGATGCGGGGAAGCTGCCCGTCGCCGGTACTGGGGGACTGTAGCTGTGATCCGGGACCACGAGGCATGCACCCGTCGTGGTCCAGCCG
GCCTGGCGCGGGGTCACAGAGCGAAACGGCGGACGCTAGGAACATTCTCTACCACCCCCCGGCGGCACCTTGCAGGGTCTACCCCAGTAAGGACCGGC
CCCAGCAGTGAGTGCGCCCGGGGGCTGGCAGCGGTAGATTGGGTCGCACGAGCTCACGTGGGCCGTTGACCGGAACCGGCAACGCCTGGGACTGGAGC
GCAAACTGGGGTACACTCTTCACGCCCGCTGCTCGTCATCGACCCCGGCGGGCGCCCGGGACCGGACCGGGGGCGACCCACCGGGGGACGGCGCGGCC
CCTGGATGGCCCTGCCCTCGGTCACGAAGCGGCCGAATGGTTTGGGGGGCCTCGGGACGCGGCCGTGGAGGTGCGGGGGGCCTGAAGTTCCTGCCTGGC
CCTCCTCCTAACGGCTTCACGCCCCTCAGGGCAGCTAGGGATTAACGTGGCCGGGTCACACGACCCGCCCAGCGCGGTACGCTGGGCCGCAGTGATTGC
GCGCGCCTGGCGGGTCCTCACCCCCCCACACACATCCGGGTCGGGGCGGAACTGGGTCCCGCCGTTCCCTCAGATCTGCCCCCTGACTTGAACATGCCCA
AAAACCGAAACCGGGCCGGCGCCCAGACCCCCGTGCTAAGGTTCCCACCGCCGACCCTAGCCCCCCCGGAGGTCCCCTGTACTCCGCACGCGACGTCGT
ATCAGGTCCCAGAGCCCGACAATGCATGCGCGTCCCGCTCTGCGCCATGCCCATTGCCCGACCAGCCCCGCCCAGCGCAGAGGCCCCGACTGCTGGCGC

CCTCTGTGGCGCTCGGCCGCTCGAGAGCACCTGCCGCCCGACCGCAGTCTGGCTACCTGCTCCACGGGGCGGCGCTCGTGGTCTCCCGTACTCGGAACGC
CACGCCCCGGGCTCGCGCGTCTCGCGGTCCGACGCATCGTGGTCTTACCTCCGACCCTTTCCTGGGGTCGAGCGCGGGGGGGCAGAGCGCGCTCATCCAG
CCCCGCGCGGCAGCACCCGCCCGCCCAGGGTGGTACCGCTCTCGAGAGTCCGGACGGCCGAACGCCAGGGCAACGGGCCGTGTGAAGACTCACCGTTT
CGGCCGACCGCCATCCTAGCTGGGGCGAGCCCCCGCGCGCGGCCGGATGCGTATGACGTGCGCGCGCTCGGTGCGATGGCGCACGTCGACTGGAATAAC
CCCTTGCCAACCCCAGCGCCGAGAGTCCCTCTTCGGGTCTCCCGTCTGCCTCCGATGGGTGGCCCCTGCCGGTACTTGTTAGGTCCTGGCTCGCCGGGCGC
CTCGTCACCTCGGCAGCCTGGATCGCTTGTCGCCGCCGCAAACGTGCGCGCGTTTCGCCACGGGCCGCCCCGCGAAAAGCGGCGGGAACGTGGACCACG
CGCGCGCCCGCCATCGGCTCGCCGGGATCCCCACCGCGGCAGCGCCCCGCCACAGCTCAGGACGCGGCCGATCGACAAGCACATCTCCCGACTACCGG
GTCCCGCGGCGTGAACGTGGTCATGGAAAACCCGAGCGTGTGCGTTCCTGTCACGGGATCCCGCCGTGGGCAGTTCGCGGCAGGGGCCTACCCGTCGAG
GGGCTACACCGGACCGCCCCTCCAGCTGCATCGTGCAGCCCTAGTGCTGCAGCGAACTTGCCCCGGCCATCCCGTCCGCCCGCAAGCGGCTGGGGACAG
GTTCTCCGCCCATGCGCAGCCAGTGGCCTCATTCATGAGGGCGTGGCGGGCCGGGCCGCGTTGGGCCATCGCCGGTTGGGGGCCCCCACGCCGTCTACCT
GAGTGGACGCGCCGGGCGAGGCTCCCACCACCCCAGTCCTGGGGTCCCAGACGGCCTCCTGGGCGGAGTGTTCTACCCCGCGGCCACCCCCAGCCCGTC
TGCGGCCGAGGCCCCACTGGAGACTGCATTCCCCGCGGCCGGGGAACCGGCCACACCGGCAACGGCGCTCCTGACCCCACCCGCCTGCCACACTCCCCG
CGGCGCGGAGCCCCACCCATACTAAGGCCCCTACGCGCGGCCGACGACATGTTGATGGCTTCGCTGGGGGGGCGGTGACACGTCCACGAGGCGCCTCGC
CGCCGTCGGCCCTGTCCCAGCACGTTCCCCCGTCCCCCGGGGGTGCGGCGTCCTGCTCGCCCCACGTGTAGGAACGCCGACCGGGCTGCGTCGTCGATGA
GGCAGTGGCCCGCTACGCGGCTCCTCTTCGCACGTGCTTTTGGCTGAAGAACGGGCACCCCCACCATTACGCAGTTGGGACGGCGCGCGGCTGCGAGGG
CCCAAATCTCTCCCATGCGAGTCGTGGTCGCGCCCGTGGACCTCTGAGGGGCAAGGCCGCTCGTCTGCTGAGCGGGGATGTTGCGCCCAGCAAATCGAG
GAAGGTTTGCTCGTAACTTACGGGTACCGAGACTCCTCGCTTGGCTCACCCATTGCGGCAGCCGCGGCCGCCGCCGGGACCAGTAACGGCGTGTCGACC
GCCCCTCGACAACGCCTGGTAGGACGCTCCCCTCCCGGCCCGGACACTAGCCCCCGCAACAGCGAATTCCAGGGGCGTCTTCCGCCCAACAAAGACCCA
TCGTGCACGAATTGGGCTGGCGGGCAAGCATGTGCATCACGGACTAGGGTACCGCCTGCCTCCCCGCTGCCTCCGGGGCCCTGGCGCTCCGTCCGATTTT
ATCCCGCTCTGCTGCGATACTCAACCAACCGTTTCGCTTTATTCAGGCCGCCTCTGGGGCCCGGCCGAACACTTTCTCCCGACAAGTGGCGTGACGGACCT
GCCTTCGCGCCGCCCCGAAACAGGCGGGCCGGCCTCGCACCCCTGGGGAGCGGTCCGCCGCAGGCGGGCCTCCTGAGCGCGCGGGTGGCCGGCCAGCG
CGCATACCCCCTCGTCGCTGCCGCCCCCCCCGCCCCGATCCCCGGCCCCGCTCGGCAAGACAGTGGAACCCGCCGCGCCCTTCACTCGCTCGGCTGGCCG
TATTTCGTGGGTCCTCACCTCAGTCGCGTCTGACAGATTAGACCTCGCCGCTGATGCCTAGGGTGGGACACCACCTTCCCGCCCGCCACCACCCTGTGGA
GCGTGAGCGCACTTAAGTCTCCCTACCTCCCCAACTCAGCAGCGGCCCCAGGGCATCCGCGAGGCTCCGTTGACGCACGTGCCGTCGTCCCGCGCCCACT
GTGGCACCTCGGCGGCGACCCTCCGCTGCGGGATGCCTGGAGGAGCGGTCCAGCCTGGGCGCTCTGCCGGTCACCAACGGCGGCCGGGCGGGGGCCCTC
GCGGTCGGACCGAACTTCGGAGGGGTCCCCGGCAGCTGAGTCGAGCGCTGCTAGTGACCTCCACCTCGGCCTAGTCACACTTGCCCGGCTCCCAACCGCT

AAGACATAGGGGGCGTCGCGACGAGGGCAATGCGGGGGCAACCTTGCCACCCCCAAGCGACGTCGCCTCATGTAGGTTGGAGGGGCTGTCTTGTACTCC
ACCGAGGGCGACACGTAGCGTGCGGCGGCTCGACTCGAATCTTGCGCCTCGGTACGCGCCACGCGACCGATGACCTTTGCAGGCGACTCTGCGAGAATG
GGGCCCGCGTCCTGGGGCGTGCTAAATCACGACCGCCGCCGCTGGCCTCCAGGAGGTCCCAGGGGGCAGCACACGCCATTCGCGTCGGTGGTCTAGCCT
CGCGGCACCTCGACGAGTCCCAGGACGCGTCGGGCCCGGTTCTTCCTCTTCCTGTGTGGCAGTCCGGCCGGGTGACGCTTCCGCCGCTGGCTAATTCAGA
CCGCCGTCAGTCGACTGGGCTGCCCTAACCAGCCAGGGCACTCGTGGACTTCCGGCGCCGTCCGACTCCACGCGGCGCGCACAGTCGCCTCCCTCCTTCG
TACCATTCGGCCCTCCTCACCCCAGGGGGGGCTTGCCGCCATTCCCAACGCTGCCATTCGACGTCCCGCGCTACTCACCCATAGTGCCAATTGCGGCGTC
GCGGACACCGGCCTGGCGCAGCGCCAGAGCGCCACCTGTGACGTCGCTCACCGCGGGGGGAGCCCAGCCTCGACGCCCCAGATGGCTCGCGGGGGGGG
AGGTCCCGAGGCCACCCTTCCAGGTGGCACAGATGCCCCTCCCTGGAGGGGGTGGAAAGCGCGCGGCGGGCCCAACACCGTGGGCGGCAGCTGGGCAT
GCGCTCGGATAAGAGCGCCAGGATGGCAGGAGGGCCGATCTGGCGGGTGGTGGCCAGTGCGGGGCGGGGGGCGAAACTGTCTGGGGGTACCTGGAGCC
CAGGGTTCAGGGTCCGTGCCATGAGGCTGTCAGGCAAGCCAAGCCGTTAACGGCGGCCAGCGAGTGGGCAGGGAAGGTGACTAGCGCGAGCTACATAT
TGCCGGCCGCCGCGCGGGGCGGCCAGCCGTGAGGAGCAATCTGAGGGACGGCCCACCGCCGGAGGGCCTGCACCATGAGGTGAGGCGGCGGGTCCCCG
CCACGCCGCGAGACGGTACGACCCGTTCCGGATGTGCACCACCGATGAAGAAGGCGCGCACATGCCGTTCGCTTTCGGCGTATGGTCTTCGCAGCCTCCG
GTGGGAGGCGCAGCCCGGAGGGTGCCCGCGCCACACGGATCAATGGGTTGGAGGGGCGGACTCGTGGTTTCGACCATTGGTGCAGCTAGAAACCGTCCC
AAGGCTCCACTCGATCGCACTGCCGTCGATCTCGGTTCTGACTCCCACCTATGCCAACACGGGAGTGCGCTATGAAACGCGGTTTTATAGTCGGTAATCG
CGACCGAACGCGGAGCGCCGTCTCGAGGCTTGTAACGCTCCGCGGTCGTTGTGGAGCGGACCGGGTAGTCAGCGTGACGTATTTCTAGCCCCCAGTGTGC
CGCCCGGCGAGAAAGTCCTCGCGGGAGCCCGTCGGCTGCCGCTGCGTACGCGGGGGAAAGCGGCCGAGGTACGTCTTCCTGGTGGCTTGCTGCCGCCAA
CCACCGATCGGCCCGGCTCGATGACAGCCGGCGAGAATGGGTTGGGGCCTACTTGGCCGGGTTGGCTGATCGTACGCAAGCAGCGTGCTGCGGGCCCAC
GCAAAGGGGAATGGCGGGGGAGCGCCGGCTGCCAATTCCCCCCGGCTGCACAGGCATGATCCGGTGTCAAGAACGGTCTCCGGCCCACGCGGCCGCGC
CTTGGCCCGAGTACGGCTGACTCAGAAGCCGTTGGGTGCGAGGGCGTGGGGTGGTGGCGCATAGGGAGCCAGACCCGACGGAGCCAGGATGGTTCGAG
CAGTGTCACGGCCCGGGGTCATGTGGGGCCCGCCCGCACGGATGACCCGCGTCTCGATAATCGGTTGCAGGCGTGCCGTTCCCACGGGGCGCGCCGGTT
ATCGAACGGGTGCGCGCGTAGACTCGCTTCCGGGCCCCGGACCTGGGCCAGGCAGAAGCGGGGATTACCCGTCTTGATCGTAGACAGCCCGGTGCCCCC
GGGGGCCGACGTACAGCTGGCAAGCACCGGTCCGGCGATTACCCGACCTGGGTTGGCAGGTACACGTTACAGGGGTGGCGTGCCCCCCGGCGCTTGGCG
CCGGCTTGGAGGGACGGGGGGGGATCTAGCTCAGATGGCCAGCGAGCGTAGCTTCGACGGTGGCAAGAGCGAGCGGCGGCGTGCTTGGGGAGGCCGCG
GGCGGGTGCGACGTCCAGTTCGTCACACGCATGAGGGGTTTCCGGGGAGGAATCACCGCCTCGCGCAGGCGTGTCCGACACGACCCGTGTAGCGGGTGT
GAGACATTTAGGCGGAGGCCAGAACCCCTTGCCGGAGGGCTTGTAGTATATTGTCAATGGGGCCGACACCACGTGCGGATCGGGACTGAGAGCGCGTAG
AGCCTTGGCCCTTGCTATATTTGTGGAGCGGACACTGTGCTTCCCACACGTCACGCTGGCGTAAGCCGATGCCCGTCACCGCCCGCGCCCCAGGCCGAGT

GCGGGGGGCCGCAAGGTCCTGAAGCCGATCTACGATGGGCTGGGTGGGCGACGTCGTGGGCTAAGCTCGGCGGCCCCACCGTGAGGCGGACGTGGGGG
CCTGTACTAGAGATGGAGCGACGGTCGTCGATACAGTTCTTGCCAGGAGACCGAGCATTTTGCTAGGCACGGGCTGCATGGGCAATTCATTAACGGCGG
CGGGCCCACCGCGGCCAGCGGTGGCGGGGCGTAGTACCGGTCGGCAGGGATACGACCCCGGGCCGAAATTCGGGGCCGGCGGCCAGGGGGCGGCGCG
CCTGCGCGCCGTGACGCTGACGCCCTCCCGGGGTGCCGCGGGCTGGCCTGAGGCCCAAGGAGGCGCCTGGGGGTCGAGCGCGGGCCCGGGCGCACGCT
GATCCCGAGCCGACGAGCTTACGGATTGACCAAGAGCACCGCGAGCGTCCGAGCGCTGCGGGCGTGGTAACAACTACATCCCGGGTCGAGTTTGCGGCT
GGGACGTGGGATTGGCCGTGCAGACGCGCTGGGGGGGGAGCGGGGCTGGCCCCTCGCTCCGTATTCCCCACTCCACATGGTCCCCGGGCGTACGGGCGT
CCCCGACGGTTCGGGACGCGGGAATCGCGCAGGTGCCCCTCGGTGCGTGGCGGGCTGCGTGTAAGGCGCTTTCGCTGCATGCGGCCGTCTCGCGGACAT
GGCGTACGGGGGCGACCAGGGCGAATCCAAGGTGCGCCCAACGGGCCGAGGCGCTACAGATGGGGAGCCCCGGGCGGCCGTACGCAGGAGTCCAGTG
GCGTATGGAGGGAGGCCTGGGGCTCCGCAACGGCCGAGGCTATGCCCAGACCCCATGCCATTGTAGGCCGAACGCGATAGGTCACGCCGGTGATCCTGC
CGGGGTCGCGGGCCGGATCCACAAGGCCAGACCCATAGCGTAGTCTATAGGCGTCCGGGAAGGGTCGTGGGGGGGCTAA

>2017.TE.25009.1.12

CACGCCCCCCTCCCGGCCAGCCCGCCCCCCGCCCCAGCGTCGTACCCGGCGTCACGACTCCCCGCCGTCGAGAGGCCTTTGCCGGGGCCCCGGCTAGCC
CGGGGCGAGCGGACCGCTGCTCGCGGGGGGTGTCACCCGGGGCACACCGCGCCAGCCTCCCGGGCATGCCACGCCGGGTTCCGCCCCCGCTGCTCCAAT
TTGGGGGGCCCCTAAGATCCCGTTCCCCAGGCCAGACGCGCCCGCGGGCCAACATGGCCACACCGTGTCGCCCCCCCGCCTGCCGCACTGACCGTCTTGC
GGGCCCTGCTCCCTGGCCCGAGCACACCTCCGGGGACCCCCCTCGCTCGGCCGGGGGAGCCTTAAGCGCGGCACCGAGTAGGGGGTCGCGTGTTGCCGC
CGCGCTGACGCCCCGGCTGGGACCACTTTCAGGTCGAGACGCCGGGGTGGCCATGCGGGTCCAGCCGCACGATGGCGCGGCGATGTGGCGCCGACTGCC
CCGTCTGTCTTCTCCGTGCCAGCCCCTCGCCACCATGCCGGTCCGGCCCGCACGGGGGTAGGAGGTTCTGCCGCGGCGGGAGGCGTAGGACGCCATATCC
GAACCGGCGGGTCCCCCCGGCCACCGAGTCGACAGGACCATAGGCACCGCGTCCGGGCGGGCGCCCTTAACGCCGCCCCTTACTGGAGAAGCAGAGCT
CTGCCGAGGCACCCTGCCTAGCTGGCTGCGTCCCGCACCCCGCGATCCCCCTGGTCCACTTACCCAGCGGGATGCGGGGATGTTCGCAGCTATCACACGG
TCGGGGCCTGCCTCCCTGGGCCGGCGAGAGCACCGCCACCTTCGTGTTCCTTGCCGCCGGGCGACGTGTCCGGGAAAGGCTTCGCCGCGCGCCCGCGCA
GAGCGCCGGGGAGTCCCGTGAGCGGCCGCAGGAACGTCACGGACCCCGGCTTGGTGCGGTCCCCCTCAGTGGTGCCCGACCAGAGACCGCGGAAAGAC
TCCAGGAAACGCCTCGGGGGGAAAGCAGGGCCGCGCACTTCCGCTCCACGCTAGCTGGGGGGACCACGACGGCAATACTGAACACCTGGCTCTTCGGC
AGCCCGGGCTATGAGGGGCCCTCTCGTCCCAGCCAAAGGCTGTTTGCCTTCCTGTCCGGGCACCCTCGACCGCACCCGGGCGGGCGCAGGCTTCATTGCG
GCGCGACTCGCCAAAAGTCCCCGCGATCTCAGCGAGTACCCTGAGTGCTGGCCCTAGTCCGCCTGCCCCCTTAATGTCGCTCAGGTCTGCAATCTCTTACG

ACCGGCTCGGGTAGTGGCCGCACGGTGCGTAGGTCCGATGCGGGCCGGGCCGCTCGCGCCGATCGTGCCGTGCGGCGGCCCTGACACCGTATGCGTGCG
CGCTCCGCAGGCTCGCGAAAATCCTTCGCAGGCACTTGGCCCCTCTGCTAGACGTGGTGTCTCCTAACTGTGGCTCACGCACGGCGGTTAGGCCACGGTA
GCCCGCTGTTCGGTCCTTACGACTCGGCACACGCCCCCGTGCACGCCCTCGGGTTCCGCAGGCCAACGCCTGGCCCTAGGGTTGCGTGCCGGCCTGCCCG
CGATGCCACTTGCCCCACGTCCCCCACCTGGCCGGCGCAGACGGGTTCAGTGGGTGTCGAACGCCATTCAGTCCCCTGGGACTCCCCCGACCCCTCCCAT
GGCACCCGCCGCCTGGCCTTGGAGCTCCTGCGCGTTGGACCTTACTGCGGTCGTCAGCTTCTCGGCGCGCCCCGGACTAGTCCTGGGCGGCCAGCTCGCG
GGACCCTAGCGCGCACGCGTGCCCGATTGCCGTATCCCCTCGACGGCGCCCGGGCATAGGGTTCGATCCCGCTGCTATTGCGGGTGCGTCCAGACCTGCC
GCCGTTTGGCGATCCGAGTGTGCGAAGGGAGCCAGCCAGCGGGCCGCCTGGCCGCACTCCAGGATCCTGGCGCCAAGGGAGAGCCCTGCCAGCGCTAC
GCGAATACTCGACCTGTCCGCGCCCCGCCTCGGGCTTGCGAGATGGCCACTAGACGCTACCGGGTCGTCACCCCGTCGGCCCCACAGGGGCCCGGCTTC
GCGTGGCTTTCTTGTCGAAGGCCCCGAAATCCGGCAGGCCCCGACACCTTCACCATGAGCGAGGACGCGCGGTGCGGGATGTTCAGTTCATGCACGCCC
CTCCACGGCCGCCTTCGCGCGGACAGCGTCGGGCGCGCCCAGGACGAGAACAGACCCTGGGCCAGCCTCAGGGCCCGGCCTAGGCGACCCTGGTGACC
AGTTTCCACTCGGCACCCTCGGGCTTCGTGGCAGTGCAGTCATGCCCCCGGGTGCTCCCGCGCGACCGCGCACGCCCCACCCAAGGTTCTTGGTCAACGC
GCCGGATCTGACGTCGCGGTCCGGCCCGCGCCGTAGGCAAAAATGGGTTCGGCTCCCGCCACTCCGCCAGTTCGAGGGCGGTAAAGATCTCAGCCGCCG
TGTTTCGGGCTATCTCCCCGCGATCCCGGGCCACAATCGCAGGTGGCTGGGGCGTCGGCCAGACCCGTCTCGCCGGCCTTTCTGCGCACATTACCTCCGA
GCAAGGCAACCGCTCCGCCCCATTACAGGGGGTTGTCTGGTACCTCCGTCACGTACCCCCGCATCGCTGGCGGGCTGACCCAAGCCCCCTCCCATCCCGA
CCCTCACGCGCTCACGCCCCTTGCCGGGCGGGGTCGTGGCTGCCCAGGGTCCAGGGCCCGCCCCGGGGGCCGGTTGGTTCCAGGAACTGAGGGAGGGTC
GCCCAGCTGACCGCTCCTCTCGCGGGGTCTATTCGGCGTGGCCATCCGCAGGTGCGACAATGCCGGCTCCCATTGCCGGGCCGACCTTGGTCCCTGGCGC
TTCGGACTCCCGGGGAGCGCCTCCCTTCATCGGCGGCCCCTGCCCCCGTCATGACCCCCCTTACGCGCCGGTGGTAGCCGCGCGTGGTGGCGTGCCCCTCT
CCATCATTACGCTCCACAGGAGTGGCCACCGGTATGGGCGCGGTGCAGTCGTACATGTGTGCGGCCCTGTTTTGCGAACCCTTCCCGCGGTGACCTCCGC
CCCAGCGGCCTTCTTCGACCGTTAACGCCGGCCAGCACCCGAAGGGGGTCACACAAAGCCCTGCAAGGATGCGACGCTGCGTAGCCGCAGGTTAGCCGC
TTCCTTCCCAAAACCGTGTCAGAGCTGTCCGGCGGAACGTTAACGCCGCCGGGAGGATCTGCGGCTACCCTCAGGCCCGTCCGGTGGGAGTGGCCGGCG
TCGATGGGGGTACGCACCCCACTAGACGCGGATTTTGGCCAACGGGCACGGCCGCTTCCGAGTTCCCCTGCGGTCTGAGTGTTGGTACGTTCAGCCGGCT
GTTCCGTGGGGCAGAGCGCCGCACGCATGCCGCAGGAGCGCGCCCTCCGCGTCTTGCGGGCGGGGTCCTAACGAAGCCCCCTGGGCTTGGAGGTAGGGG
GCGCGGGGGAGCGATGGGAGCGCAAGCGTGCCATCGGGTCCGGCCCATAAGTGTGTATGGCGCACCGCGCTGCCGCGATGTCCGGTGCGCGGGCGCCG
CTGGCTAGCTCACTCTCCCCCGACTCCGCCGCCGCGCCCCGAGACGTATGGAACAGCTGGCGCGCGTCCCCCGCCGCGGGCGGCAACGAACTAGATCCA
CCGCCCTCACGAGGAAGCGCGCCGCCACCACGTCTCCCCGGCCGACGCGGTCCCACCATCCGCTGTGCCGGCGCGCGAGCAGCCTGGCCCTCGGGGCGG
TCGCGCCAGCGCAGACACCCTGTCACGCGCACGGGAGCCCTGGCCACCGCGTCGACGCCCGGGCTAGCCCCGACTATCAGTACTGACCAGAGGGGCGC

CGGTCGTCCCGACTGGTTCCTCCCGAAACGCTGGCGGTGGGCCCGCACGATTGGTGCAGGCCCCCAAGAGGATACGGCTATTCGCGGCTGCCCGAGGCG
AGTCGGGCCAGACCGGTCGCGCGTGTGTGTGTTAGCCCTCCGTCGCGGCCAAGCCGGCGGCACCGGACTGGGCGACAGTAGCGTTCCTCTGTCACTAGTC
ACTCTAACGGACAAATGGAAAAATGGCACCTATCGCTACCGACGGGGTTTTCCTCCAGGATAAGCCTCCGCGCCCCCCACTGGACGCCAGCCATCCTTCC
CCGAACGGCTCGCCTGTGCCGGCTCCAACCCCGCGGGACCTCCCTTCGCGGCCCCGGCGGGGATCGGTGGACCGTCGCCGCCGGCGCCTACCGATCGCG
CCGCCACGGTCGTGCACCAACCAAGCACTGCTACACGCCTGTGCGGCCGAGGCCGCCCCATCCAGCAACGGGGCACCGTTGACCCGTCCCCCATGATGC
CGGACATGGTAGCCCCCACACATGCATCTTCGGGTAACCCGCGCCAGGTCCCCCCCCGCGCCGCCGCCGGCTTTCCCCCCGCTGGTGCTAAGCGGCAGAT
GCACCACTGACGGGACACACTCGCCTCCGATCCAGTGCGCTGCCGCCTGCGGCCCCCCACCGGACTGGTGCCTGGCGCTACACGGAGCCGGCCGCGCAC
TGTGACGTATGGATGCCCGAGGAGACATCCGCCCCGGACGCATGCGCAAAAGACCACTCGCTCGACGCCACGACGTGTGCGGCCGGGGTGATGAACGT
CGGCCGGTGTGCCAGTGAGGCTTGCACCTACACTCCCTGGTCCACCGGGCCGTTCGAGCGGCACCGGGCGAAGAACCCGCAGCCTCCCGGCTTACGCAC
CGGCCTCGCGTCAACAGGCAACCACGACCGCAAGGCCGGCAGTGTGTGTCGCTGTGGAAAGGTGCGCCGCAGGCGACCCCCACGTCAGCCCACTACAA
AGCGTGGCAGCATGCTGCGCAGCGACCGCACGCCCCGACACAGCCCAATGCACAGCATCGGGGGGGCGGAATCGCCCCACGCCCGTAACATCCCGCGG
GTCGACCGCCTTACACGTCGACTGCCACACCCTGGTGCCGCAGTGGTATCCCGAGACCGCATGACCCGCCGCGAGCCCGCTGACCACCTGTCCTTGATCG
CAGTCGCCACGGGGCCGGAAGGTGTACCGCATACCGCACGTGCTGCGCGTGTGCAGCACGGGGGCACCCTGCATGTATGGATCCCTCTAAGCCGACCTG
CCGCCCCAGGCCAGAGGCTAGAGTCTGCCGCCTAGGCCCCCCCCGTGGTCGCCAGCCCCGGTGGCGGCCCACCCTCGGGGCGACTGACCGGCCACCAT
CCAGGGTAGAGAGGTTTGGCCCTAAATACGGGCGTGCCGAGGAGGTGTTTGGGGCGAGCCGCAGCAGGTTCGGCGGATCCTTGAGGGTGGGGCCTGGGG
TGGGACGGGGGCGGCCGAAACGCGAGCATAGGCCCCTGCCTGGCCCCTTTGCAGTCTGTACTTTTATTGACATGATACTAATACCGTGTTCCGCCCTGAG
ATCGCACGCGGACCTACCGTGCCTCTCTCTGCATAGTGCTTCAAGAGCCCAGCGCGACTGGGGACCGGCTCCCGCAGCCGGCTCGGGGCGTAACGCTCG
GTGGAAGGGTCACCCGGATAGCTCCCGGTTAGTTTCGTACAGGGTGTCGGGAGCGCCCCCAGCGAGTCTCCTGGAGGTGCAGGGACGGCGGTGGTCCTC
GAGGGGGCGGACGTTGGCCGCATAACGGGTAGTCTCCAGGGTCACCTATAGTCTGGCTCGCCGTCAGGGTCTGTGAGCGTCGTGCTCACGCCTCCGCAAC
AGCCGGCGACCCCGGCTCGCAACGGCCAGCCGTGGGCGGGGGGGCGACCGGACGCAGTTCCGAGTCTAGGCATGGTGCCACCGCCCTCTCGTTGGGTTA
GTCTCGAATGGCTGTTGCGGGGGTCAGCGCGGCGGGGCCGCTGCTGGGAGCCCGCATAACCGTGGATACACTTGCTGGGTGCGCGCTCGAGGGCGGCCC
CCGAGGGCTGGAAAACCATTTCGGGATCGCCCCCGCGCACCCGTCCGCACTGCGGGGTCCAGCCTGGCGGGGGCGGGTTTCGTTGGGGCAGTACTCACG
CCCACGGCGAGGCCCATCCAGAGAGTGAGGGTGTCGCTGGGGGGGCCTACCGGGCGGAACGCGCCCCGACTTAGGGCGGCGCAGTGCACGACCAGTCA
CCGGCTGGGCGTCGCCGCGCCGTTGAGAACCCCAACACAGAAAGAGCTTGCCGCCTCTGGGGGAGCCAGGCCGAGAAGCGTGGGCGAGCGGCCACGCT
GCGGGGCCTCCGCGGACCGTGCGGCGCTCTCGGGTGACAAACCTAGTCCCCGTCCCAGGCCATGGGCGAGCAGCTACGCCGGGACTGTGCGACGCCTAT
TCGGCACATTCGGTATCATTGGCCTGGGACGACAGCCGGCGACAGGGCTGACGAGGCGGCGTGGACTCGGGGTGCAGGGACGACGTCCACCCGTACTG

GTCAGTAGCCCAACGCGGAAGCCTGTCTGGGGCTTTGGCCCCTAGACATCGGTCCGGGGCGGGGCGCGTCATCGCTACGTTGGTGAGGGGGAGTCTGCA
CCGATAGGGTGTGGTGGTGTTGCTTAGATCGTTGACGGGCCGTGAGGTGGCGCTGCTGTTTTCCGGCTTACGGCTTACGACCTACCGCCCGCCGCGCCGGC
CCGAAGGCGGCGCCCCCGCACATCGCCCTTGGGTGGTAAAACCCGCTCTGCCGCCGCTACCCCCCCCCCAGGGGTGGCCGGAACGGCGAGGGCAGGAC
ACGGTAGACGCACGCAGCGCCGCGACGCCCGGCCCGCGCTAGGGTTGGCACCCGTGGGACCTCGGCAAACTTTCCGTACGATTGGGGGGCGCATACAG
CGGACGGGGAACCCGCCCGACGCACGCGCGGTCCCGAGGGGTGGGGGACCTGCAGCTGCGTCGCGCGGTCCGCGATGGCGTCTTGCTAGACCATGGGC
GTTGCAATAGTGGGAAGGTGCCTCCCAGGCTGCGGGGCGCCGGACGCCCGACACCAAAGCTGTGCGAGGACAGAGAGGCTAGGCACGGATGGGTCCAA
GCGAGCTGACAGCCTCCATCCTTGAGTGAGCACGCCGCGGCGAAATGCCGGCCGAAATGGGAGGGGCGCGACACCGGATCGTGATCGAGCGCGTGCGC
TTCGCCCACACGCCCTGACCCGCTGAGTGTCTCGGGTTTGAACCCCGTGCCCGGGGGCGCAGTGGGATAGGCACGACGGGCAGAGTTAGGGGCCTGGGC
ATAACGGCCCTGTCCGCGGCCGAGCGGGCCCGGGTCTTCTCGACCAGGGTGCCGGCCCCGGCGGGCCCCCCACCCACGGACGGGGACCTGGAGTTGAGC
CAGGCCACAGGCGTGACCGGAATTGGAGCGCCAGGGGACAGCGGCCGCCCGCGCCAGGCCACGCCGGTGGGGGCGTTCTGCCCAGGCGTCGCGCAATG
GGCGGACCGGTCTACGCGACACCGCCCGCCCGGCCTGGAACTCCCCCCGAGCACATTCGATATGGTCCGCATCAAGGCGGGAGGCATCGCCGGGGGCC
GCGCGGACCACGACCTTCGAGGGGGACGGTGCGGGTCGCGACGAGACGGCGGCATGTGCGCGGGTGCCGTGAGCCCTAGAGTCAGCGGTTGCTGGTAT
GGGCGGACGGGCGCCCCCGACCTGCCGTGGACGCCGGCTCGCGGACTAGTCGACCCGCTATGGGTGCTGTGCGTGACGTTGGCCTTGGTCCAGGGCGCG
GCGCGCAATACCGCGCGGGCTGAGTATCGCGTCCACGGACAGGCGGCTGAGCCGCCCGGCGTAGCTGGTACACGGTCGGGCCGAAGTGGCAAAGAGCC
GGCTTGCGCCCCCAGCGCCGTTGCAGCAGGTCTGGACAGGCAGTACGGAGCGAACGCGGCGAGACACCCGGCTCCGGGCGTAGTCGGATCCGCCCGCC
AGCGGGCGCGCAGCGGTGTCGCGGTCTTGATGCGCCGGTCAGGGAGGTCGTCGCGAATGAGCCGGGTGGAGGGTGCAAGAAGCCAGACGATGGGAGCA
CCGAGTTGTCCGCGTGCGATTGCCGTCCCGGCGAAAGCGCCAGGCCGCGGCGCGTGTGGCAACGGCAAGTACCCGGAAACACGGCACGGGTCTCGTGG
CGCCGCGAGGCGGCGGGTGGCGCGCTCCGGGGGGCTGCGCAGCCCCTCAGCGCCGCTGCTAGTGCCAGACGCTTGCCCTTCTTGGGCGCGGCGGCGGGA
ACTCGGTCGCGTGCGTTCAGGGCCCTCATCCGCAGACGTGAGGGGTGCGGGCCAGGGGGCGCAGAGGCGCCTTCCGAAACCCGTAAGGGATGTTCTGGC
GTTGGGGGGCCGCCGTCTTAGGGGGGTAGCGCCGCCTCAACTCCAGTATCGCCGCAAGGCCGGGGCCAAGTTCGATCCGGGGGCGGCGGCGTACCGCGT
CCACGCGTCTCCCAGAGGAGGGTCAAGGTGCATACGGGAGGGATCATACGGCAATCGGCGGCCGGGTTCCGCCATCTAACGTGGCGATCGCAGCGTACC
AGAGCGAGGCCTATGGGCCATATCCCTGGCGGGAACGGGCTGCTTGCGCCCCGTCCTCGCCGACCCTCCGTACCGGTACGGGGCGAGGATCCCGACACA
CAGCACGGGGGCGGCGCGCAGCACCCACCCCGTCCTCGGCCCAACAGACAAAGACCCCGCGGCGGGCTTGGGCACCCCCTTTCCGGCTACGTCCACCG
ATTCCGTACAGGACAGCCGTTGCGATGGGCACCGGCGATCGGAGAGAAGGACGCCACAGATCAGCAGACGACTCGGGGAAACCACGGGACCGCGCGT
CGCGTCAACTGCCGCGGGAGTGAGAAGGGAGCGACAGGAGCGCGGAGGTAAGGCCAATGGGGCCAGTTGAAGCCGCCCACAGAGGCTGTGAGGGGCC
CCGCCAGGTTTAGGAGCTGTTCTTGGAGCCTGTCGTTAGGGGCGGGGGGGGGCTTACACGGCGCTGGGGGACCCACGGGGCGCAATCCGCCGCGGACTG

CTGGAGGGCTGCCTGGGGCAGCACCCCAGGGGCGGCCATGGCGCTTGTTGACGGGGGCGCTAGGACCTTGTCCTAGGGCGCGCCGCTTTCGCCCACAGG
GCTTCGGTGGGCAGTCTATCTGTGCGGTTTCAGTCTCGTACGCACGTGATGCGGTGTAACCTGAGCCCGGCAGTTGTCTAGAGTAACTAGCGCGCGGTTAT
GGCTTCGTCTAGAAACGGGCTGCAATGTTCCGGGTGTTCATCACACCACGTTCCGCTTTGCAGCTCACCCCCCGACCACACCCGGATTCGGACCGGTGGT
CCTTCCTCTGAGGTACTCGCCGCTCATACAACCGCGGTTCCCGCTGTGGTCGGCACGGCCCGGGCTGCAGGGGCGTGCCTTCACGGGTAGTGTGCCTGCG
CGGCGTGCGGCTTCCGCAGTCCACGGACCGTTCCGGGGGTGAACGATGGAGGTGAGGGGACGAGTCCCTCCGAGATTAATGCCCCGCGGCAAGCCCGAT
CAGCGCTGCGGTCCTGGGCTGTCAGCGCGCGCGGAGGAGCGCCCGATCGGGCGCGGGGGAATGAGAGTGTGGAGGATCATAATAGAGAGATCGCCCCG
GAGACTGCGAGGGGCATGGACGCGGAGGGACGGGGCGGGGGGTCGGCACGCCCGTGCGGCCTGTCGTCATATCCCACGCTGTCACTCGGCTGCGTCCGG
CACTCCGGGCGGCCGGCGAGCAAAGCCGCCCTGCGCGGCCCGATCGAGGGTGCGGCGGCGCGACGTCGTGGCGTGGGAAATGCGGCGAGGGGCGGGGT
GGGGGACGTCATTGAACCTGCAGCGCGCACGGTGAGTACCGCGGCCCGTGGCGGGGCTGTACTGCGGGACCCCGGAGCGGTAGCGGGGGTTAGACTGG
ATGAGGGTAACCTGCTGCCCCGCGCACCACGTTCTTGGCCAGAGGCCCTGTGGGTGTTGGAGCAGGGTCGAGTATCCCTCCGTGCTTGCCTGCGTGGAGT
GGTTCGCCATGCAGTTGGACTCTTGGCGTCAGCTGGGGCCAGGCGGCGCCTCCGAGGTCGTAAAGGCCAACAGCGCTGTGAGATGGGCGCAGGAAGAG
CGCCAAAGACCTCCTGCTCCCCGATGTCCGCCTCGGGCGGACGTTCGGCCGCCTCGGGACTAGTTGAAGTGCGGCTTGGCTGGGGGCGCGGGACTCGGA
AGTCCGGGTGGAGGATGCGGGGAAGCTGCCCGTCGCCGGTACTGAGGGACTGTAGCTGTGATCCGGGACCACGAGGCATGCACCCGTCGTGGTCCAGCC
GGCCTGGCGCGGGGTCACAGAGCGAAACGGCGGACGCTAGGAACATTCGCTACCACCCCCCGGCGGCACCTTGCAGGGTCTACCCCAGTAAGGACCGG
CCCCAGCAGTGAGTGCGTCCGGGGGCTGGCAGCGGTAGATTGGGTCGCACGAGCTCACGTGGGCCGTTGACCGGAACCGGCAACGCCTGGGACTGGAG
CGCAAACTGGGGTACACTCTTCACGCCCGCTGCTCGTCATCGACCCCGGCGGGCGCCCGGGACCGGACCGGGGGCGACCCACCGGGGGACGGCGCGGC
CCCTGGATGGCCCTGCCCTCGGTCACGAAGCGGCCGAATGGTTTAGGGGGCCTCGGGACGCGGCCGTGGAGGTGCGGGGGGCCTGAAGTCCCTGCCTGG
CCCTCCTCCTAACGGCTTCACGCCCCTCAGGGCAGCTAGGGATTAACGTGGCCGGGTCACACGACCCGCCCAGCGCGGTACGCTGGGCCGAAGTGATTG
CGCGCGCCTGGCGGGTCCTCACCCCCCCACACACATCCGGGTCGGGGCGGAACTGGGTCCCGCCGTTCCCTCAGATCTGCCCCCCGACTTGAACATGCCC
AAAAACCGAAACCGGGCCGGCGCCCAGACCCCCGTGCTAAGGTTCCCACCGCCGACCCTAGCCCCCCCGGAGGTCCCCTGTACTCCGCACGCGACGTCG
TATCAGGTCCCAGAGCCCGACAATGCATGCGCGTCCCGCTCTGCGCCATGCCCATTGCCCGACCAGCCCCGCCCAGCGCAGAGGCCCCGACTGCTGGCG
CCCTCTGTGGCGCTCGGCCGCTCGAGAGCACCTGCCGCCCGACCGCAGTCTGGCTACCTGCTCCACGGGGCGGCGCTCGTGGTCTCCCGTACTCGGAACG
CCACGCCCCGGGCTCGCGCGTCTCGCGGCCCGATGCATCGTGGTCTTACCTCCGACCCTTTCCTGGGGTCGAGCGCGGGGGGGCAGAGCGCGCTCATCCA
GCCCCGCGCGGCAGCACCCGCCCGCCCAGGGTGGTACCGCTCTCGAGAGTCCGGACGGCCGAACGCCAGGGCAACGGGCCGTGTGAAGACTCACCGTT
TCGGCCGACCGCCATCCTAGCTGGGGCGAGCCCCCGCGCGCGGCCGGATGCGTATGACGTGCGCGCGCTCGGTGCGATGGCGCACGTCGACTGGAATAA
CCCCTTGCCAACCCCAGCGCCGAGAGTCCCTCTTCGGGTCTCCCGTCTGCCTCCGATGGGTGGCCCCTGCCGGTACTTGTTACGTCCTGGCTCGCCGGGCG

CCTCGTCACCTCGGCAGCCTGGATCGCTTGTCGCCGCCGCAAACGTGCGCACGTTTCGCCACGGGCCGCCCCGCGAAAAGCGGCGGGAACGTGGACCAC

GCGCGCGCCCGCCATCGGCTCGCCGGGATCCCCACCGCGGCAGCGCCCCGCCACAGCTCAGGACGCGGCCGATCGACAAGCACATCTCCCGACTACCG

GGCCCCGCGGCGTGAGCGTGGTCATGGAAAACCCGAGCGTGTGCGTTCCTGTCACGGGATCCCGCCGTGGGCAGTTCGCGGCAGGGGCCTACCCGTCGA

GGGGCTACACCGGACCGCCCCTCCAGCTGCATCGTGCAGCCCTAGTGCTGCAGCGAACTTGCCCCGGCCATCCCGTCCGCCCGCAAGCGGCTGGGGACA

GGTTCTCCGCCCATGCGCAGCCAGTGGCCTCATTCATGAGGGCGTGGCGGGCCGGGCCGCGTTGGGCCATCGCCGGTTGGGGGCCCCCACGCCGTCTACC

TGAGTGGACGCGCCGGGCGAGGCTCCCACCACCCCAGTCCTGGGGTCCCAGACGGCCTCCTGGGCGGAGTGTTCTACCCCGCGGCCACCCCCAGCCCGT

CTGCGGCCGAGGCCCCGCCGGAGACTGCATTCCCCGCGGCCGGGGAACCGGCCACACCGGCAACGGCGCTCCTGACCCCACCCGCCTGCCACACTCCCC

GCGGCGCGGAGCCCCACCCATACTAAGGCCCCTACGCGCGGCCGACGACATGGTGATGGCTTCGCTGGGGGGGCGGTGACACGTCCACGAGGCGCCTC

GCCGCCGTAGGCCCTGCCCCAGCACGTTCCCCCGTCCCCCGGGGGTGCGGCGTCCTGCTCGCCCCCCCGTGTAGGAACGCCGACCGGGCTGCGTCGTCGGT

GAGGCAGTGGCCCGCTACGCGGCTCCTCTTCGCACGTGCTTTTGGCTGAAGAACGGGCACCCCCACCATTGCGCGGTTGGGACGGCGCGCGGCTGCGAG

GGCCCAAATCTCTCCCATGCGAGTCGTGGTCGCGCCCGTGGACCTCTGATGGGCAAGGCCGCTCGTCTGCTGAGCGGGGATGTTGCGCCCAGCAAATCGA

GGAAGGTTTGCTCGTAATTTACGGGTACCGAGACTCCTCGCTTGGCTCACCCATTGCGGCAGCCGAGGCCGCCGCCGGGACCAGTAACGGCGTGTCGACC

GCCCCTCGACAACGCCTGGTAGGACGCTCCCCTCCCGGCCCGGACACTAACCCCCGCAACAGCGAATTCCAGGGGCGTCTTCCGCCCAACAAAGACCCA

TCGTGCACGAATTGGGCTGGCGGGCAAGCATGTGCATCACGGACTAGGGTACCGCCTGCCTCCCCGCTGCCTCCGGGGCCCTGGCGCTCCGTCCGATTTT

ATCCCGCTCTGCTGCGATACTCAACCAACCGTTTCGCTTTATTCAGGCCGCCTCTGGGGCACGGCCGAACACTTTCTCCCGACAAGTGGCGTGACGGACCT

GCCTTCGCGCCGCCCCGAAACAGGCGGGCCGGCCTCGCACCCCTGGGGAGCGGTCCGCCGCAGGCGGGCCTCCTGAGCGCGCGGGTGGCCGGCCAGCG

CGCATACCCCCTCGTCGCTGCCGCCCCCCCCGCCCCGATCCCCGGCCCCGCTCGGCAAGACAGTGGAACCCGCCGCGCCCTTCACTCGCCCGGCTGGCCG

TATTTCGTGGGTCCTCACCTCAGTCGCGTCTGACAGATTAGACCTCGCCGCTGATGCCTAGGGTGGGACACCACCTTCCCGCCCGCCACCACCCTGTGGA

GCGTGAGCGCACTTAAGTCTCCCTACCCCCCCAACTCAGCAGCGGCCCCAGGGCATCCGCGAGGCTCCGTTGACGCACGTGCCGTCGTCCCGCGCCCACT

GTGGCATCTCGGCGGCGACCCTCCGCTGCGGGATGCCTGGAGGAGCGGTCCAGCCTGGGCGCTCTGCCGGTCACCAACGGCGGCCGGGCGGGGGCCCTC

GCGGTCGGACCGAACTTCGGAGGGGTCCCCGGCAGCAGAGTCGAGCGCTGCTAGTGACCTCCACCTCGGCCTAGTCACACTTGCCCGGCTCCCAACCGC

TAAGACATAGGGGGCGTCGCGACGAGGGCAATGCGGGGGCAACCTTGCCACCCCCAAGCGACGTCGCCTCATATAGGTTGGAGGGGCTGTCTTGTACTC

CACCGAGGGCGACACGTAGCGTGCGGCGGCTCGACTCGAATCTTGCGCCTTGGTACGCGCCACGCGACCGATGACCTTTGCAGGCGACTCTGCGAGAAT

GGGGCCCGCGTCCTGGGGCGTGCTAAATCACGACCGCCGCCGCTGGCCTCCAGGAGGTCCCAGGGGGCAGCACACGCCATTCGCGTCGGTGGTCTAGCC

TCGCGGCACTTCGACGAGTCCCAGGACGCGTCGGGCCCGGTTCTTCCTCTTCCTGTGTAGCAGTCCGGCCGGGTGACGCTTCCGCCGCTGGCTAATTCAGA

CCGCCGTCAGTCGACTGGGCTGCCCTAACCAGCCAGGGCACTCGTGGACTTCCGGCGCCGTCCGACTCCACGCGGCGCGCACAGTCGCCTCCCTCCTTCG

TACCATTCGGCCCTGCTCACCCCAGGGGGGGCTTGCCGCCATTCCCTACGCTGCCATTCGACGTCCCGCGCTACTCACCCATAGTGCCAATTGCGGCGTCG
CGGACACCGGCCTGGCGCAGCGCCAGAGCGCCACCTGTGACGTCGCTCACCGCGGGGGGAGCCCAGCCTCGACGCCCCAGATGGCTCGCGGGGGGGGA
GGTCCCGAGGCCACCCTTCCAGGTGGCACAGATGCCCCTCCCTGGAGGGGGTGGAAAGCGCGCGGCGGGCCCAACACCGTGGGCGGCAGCTGGGCATG
CGCTCGGATAAGAGCGCCAGGATGGCAGGAGGGCCGATCTGGCGGGTGGTGGCCAGTGCGGGGCGGAGGGCGAAACTGTCTGGGGGTACCTGGAGCCC
AGGATTCAGGGTCCGTGCCATGAGGCTGTCAGGCAAGCCAAGCCGTTAACAGCGGCCAGCGAGTGGGCAGGGAAGGTGACTGGCGCGAACTACATATT
GCCGGCCGCCGCGCGGGGCGGCCAGCCGTGAGGAGCAATCTGAGGGACGGCCCACCGCCGGATGGCCTGCACCATGAGGTGAGGCGGCGGGTCCCCGC
CACGCCGCGAGACGGTACGACCCGTTCCGGATGTGCACCACCGATGAAGAAGGCGCGCACATGCCGTTCGCTTTCGGCGTATGGTCTTCGCAGTCTCCGG
TGGGAGGCGCAGCCCGGAGGGTGCCCGCGCCACACGGATCAATGGGTTGGAGGGGCGGACCCGTGGTTTCGACCATTGGTGCAGCTAGAAACCGTCCC
AAGGCTCCACTCGATCGCACTGCCGTCGATCTCGGTTCTGACTCCCACCTATGCCAACACGGGAGTGCGCTATGAAACGCGGTTTTATAGTCGGTAATCG
CGACCGAACGCGGAGCGCCGTCTCGAGGCTTGTAACGCTCCGCGGTCGTTGTGGAGCGGACCGGGTAGTCAGCGTGACGTATTTCTAGCCCCCAGTGTGC
CGCCCGGCGAGAAAGTCCTCGCGGGAGCCCGTCGGCTGCCGCTGCGTACGCGGGGGAAAGCGGCCGAGGTACGTCTTCCTGGTGGCTTGCTGCCGCCAA
CCACCGATCGGCCCGGCTCGATGACAGCCGGCGAGAATGGGTTGGGGCCTACTTGGCCGGGTTGGCTGATCGTACGCAAGCAGCGTGCTGCGGGCCCAC
GCAAAGGGGAATGGCGGGGGAGCGCCGGCTGCCAATTCCCCCCGGCTGCACAGGCATGATCCGGTGTCAAGAACGGTCTCCGGCCCACGCGGCCGCGC
CTTGGCCCGAGTACGGCTGACTCAGAAGCCGTTGGGTGCGAGGGCGTGGAGTAGTGGCGCATAGGGAGCCAGACCCGACGGAGCCGGGATGGTTCGAT
CAGTGTCACGGCCCGGGGTCATGTGGGGCCCGCCCGCACGGATGACCCGCGTCTCGATAATCGGTTGCAGGCGTGCCGTTCCCACGGGGCGCGCCGGTT
ATCGAACGGGTGCGCGCGTAGACTCGCTTCCGGGCCCCGGACCTGGGCCAGGCAGAAGCGGGGAATACCCGTCTTGATCGCAGACAGCCCGGTGCCCCC
GGGGGCCGACGTACAGCTGGCAAGCACCGGTCCGGCGATTACCCGACCTGGGTTGGCAGGTACACGTTACAGGGGTGGCGTGCCCCCCGGCGCTTGGCG
CCGGCTTGGAGGGACGGGGGGGGATCTAGCTCAGATGGCCAGCGAGCGTAGCTTCGACGGTGGCAAGAGTGAGCGGCGGCGTGCTTGGGGAGGCCGCG
GGCGGGTGCGACGTCCAGTTCGTCACACGCATGAGGGGTTTCCGGGGAGGAATCACCGCCTCGCGCAGGCGTGTCCGACACGACCCGTGTAGCGGGTGT
GAGACATTTAGGCGGAGGCCAGAACGCCTTGCCGGAGGGCTTGTAGTATATTGTCAATGGGGCCGACACCACGTGCGGATCGGGACTGAGAGCGCGTAG
AGCCTTGGCCCTTGCTATATTTGTGGAGCGGACACTGTGCTTCCCACACGTCACGCTGGCGTAAGCCGATGCCCGTCACCGCCCGCGCCCCAGGCCGAGT
GCGGGGGGCCGCAAGGTCCTGAAGCCGATCTACGATGGGCTGGGTGGGCGACGTCGTGGGCTAAGCTCGGCGGCCCCACCGTGAGGCGGACGTGGGGG
CCTGTATTAGAGATGGAGCGACGGTCGTCGATACAGTTCTTGCCAGGAGACCGAGCATTTTGCTAGGCACGGGCTGCATGGGCAATTCATTAACGGCGGC
GGGCCCACCGCGGCCAGCGGTGGCGGGGCGTAGTACCGGTCGGCAGGGATACGACCCCGGACCGAAATTCGGGGCCGGCGGCCAGGGGGCGGCGCGC
CTGCACGCCGTGACGCTGACGCCCTCCCGGGGTGCCGCGGGCTGGCCTGAGGCCCAAGGAGGCGCCTGGGGGTCGAGCGCGGGCCCGGGCGCACGCTG
ATCCCGAGCCGACGAGCTTACGGATTGACCAAGAGCACCACGAGCGTCCGAGCGCTGCGGGCGTGGTAACAACTACATCCCGGGTCGAGTTTGCGGCTG

GGACGTGGGCTTGGCCGTGCAGACGCGCTGGGGGGGGAGCGGGGCTGGCCCCTCGCTCCGTATTCCCCACTCCACATGGTCCCCGGGCGTACGGGCGTC
CCCGACGGTTCGGGACGCGGGAATCGCGCAGGTGCCCCTCGGTGCGTGGCGGGCTGCGTGTAAGGCGCTTTCGCTGCATGCGGCCGTCTCGCGGACATG
GCGTACGGGGGCGACCGGGGCGGATCCAAGGTGCGCCCAACGGGCCGAGGCGCTACAGATGGGGAGCCCCGGGCGGCCGCACGCAGGAGTCCAGTGG
CGTATGGAGGAAGGCCTGGGGCTCCGCAACGGCCGAGGCTATGCCCAGACCCCATGCCATTGTAGGCCGAACGCGATAGGTCACGCCGGTGATCCTGCC
GGGGTCGCGGGCCGGATCCACAAGGCCAGACCCATAGCGCAGTCTATAGGCGTCCGGGAAGGGTCGTGGGGGGGCTAA

>2017.TE.25009.1.11

CACGCCCCCCTCCCGGCCAGCCCGCCCCCCGCCCCAGCGTCGTACCCGGCGTCGCGACTCCCCGCCGTCGAGAGGCCTTTGCCGGGGCCCCGGCTAGCC
CGGGGCGAGCGGACCGCCGCGCGCGGGGGGTGTCACCCGGGGCACACCGCGCCAGCCTCCCGGGCATGCCACGCCGGGTTCCGCCCCCGCTGCTCAAA
TTTGGGGGGCCCCTAAGATCCCGTTCCCCAGGCCAGCCGCGCCCGCGGGCCAACATTGCCACACCGTGTCGCCCCCCCGCCTGCCGCACTGACCGTCTTG
CGGGCCCTGCTCCCTGGCCCGAGCACACCTCCGGGGACCCCCCTCGCTCGGCCGGGGGAGCCTTAAGCGCGGCACCGAGTAGGGGGTCGCGTGTTGCCG
CCGCGCTGACGCCCCGGCTGGGACCCCTTTCAGGTCGAGACGCCGGGGTGGCCATGCGGGTCCAGTCGCACGATGGCGCGGCGATGTGGCGCCGACTGC
CCCGCCTGTCTTCTCCGTGCCAGCCCCTCGCCACCATGCCGGTCCGGCCCGCACGGGGGTAGGAGGTGCTGCCGCGGCGGGAGGCGTAGGACGCCATAT
CCGAACCGGCGGGTCCCCCCGGCCACCGAGTCGACAGGACCATAGGCACCGCGTCCGGGCGGGCGCCCTTAACGCCGCCCCTTACTGGAGAAGCAGAG
CTCTGCCGAGGCACCCTGCCTAGCTGGCTGCGTCCCGCACCCCGCGATCCCCCTGGTCCACTTACCCAGCGGGATGCGGGGATGTTCGCAGCTATCACAC
GGTCGTGGCCTGCCTCCCTGGGCCGGCGAGAGCACCGCCACCTTCGTGTTCCTTGCCGCCGGGCGACGTGTCCGGGAAAGGCTTCGCCGCGCGCCCGCGC
GGAGCGCCGGGGAGTCCCGTGAGCGGCCGCAGGAACGTCACGGACCCCGGCTTGGTGCGGTCCCCCTCAGTGGTGCCCGACCAGAGACCGCGGAAAGA
CTCCAGGAAACGCCTCGGGGGGAAAGCAGGGCCGCGCACTTCCGCTCCACGCTAGCTGGGGGGACCACGACGGCAATACTGAACACCTGGCTCTTCGG
CAGCCCGGCCTATGAGGGGCCCTCTCGTCCCGGCCAAAGGCTATTTGCCTTCCTGTCCGGGCACCCTCGACCGCACCCGGGCGGGCGCAGGCTTCATTGC
GGCGCGACTCGCCAAAAGTCCCCGCGATCTCAGCGAGTACCCTGAGCGCTGACCCTAGTCCGCCTGCCCCCTTAATGTCGCTCAGGTCTGCAATCTCTTA
CGACCGGCTCAGGTAGTGGCCGCACGGTGCGTAGGTCCGCTGCGGGCCGGGCCACTCGCGCCGATCGTGCCGTGCGGCGGCCCTGACACCGTATGCGTG
CGCGCTCCGCAGGCTCGCGAAAATCCTTCGCAGGCACTTGGCCCCGTTGCTAGACGTGGTGTCTCCTAACTGTGGCTCACGCACGGCGTTTAGGCCACGG
TAGCCCGCTGTTCGGTCCTTACGACTCGGCACACGCCCCCGTGCACGCCCTCGGGTTCCGCAGGCCAACGCCTGGCCCTAGGGTTGCGTGCCGGCCTGCC
CGCGATGCCACTTGCCCCACGTCCCCCACCTGGCCGGCGCAGACGGGTTCAGTGGGTGTCGAACGCCATTCAGTCCCCTGGGACTCCCCCGACCCCTCCC
ATGGCACCCGCCGCCTGGCCTTGGAGCTCCTGCGCGTTGGACCTTACTGCGGTCGTCAGCTTCTCGGCGCGCCCCGGACTAGTCCTGGGCGGCCAGCTCG

CGGGACCCTAGCGCGCACGCGTGCCCGATTGCCGTATCCCCTCGACGGCGCCCGGGCATAGGGTTCGATCCCGCTGCTATTGCGGGTGCGTCCAGACCTG
CCGCCGTTTGGCGATCCGAGTGTGCGAAGGGAGCCAGCCAGCGGGCCGCCTGGCCGCACTCCAGGATCCTGGCGCCAAGGGAGAGCCCTGCCAGCGCT
ACGCGAATACTCGACCTGTCCGCGCCCCGCCTCGGGCTTGCAAGATGGCCACTAGACGCTATCGGGTCGTCACCCCGTCGGTCCCACAGGGGCCCGGCTT
CGCGTGGCTTTCTTGTCGAAGGCCCCGAAATCCGGCAGGCCCCGACACCTTCACCGTGAGCGAGGACGCGCGGTGCGGGATGTCCAGTTCATGCACGCC
CCTCCACGGCCGCCTTCGCGCGGACAGCGTCGGGCGTGCCCAGGACGAGAACAGACCCTGGGCCAGCCTCAGGGCCCGGCCTAGGCGACCCTGGTGAC
CAGTTTCCACTCGGCACCCTCGGGCTTCGTGGCAGTGCAGTCATGCCCCCGGGTGCTCCCGCGCGACCGCGCACCTCCCACCCAAGGTTCTTGGTCAACG
CGCCGGATCTGACGTCGCGATCCGGCCCGCGCCGTAGGCAAAAATGGGTTCGGCTCCCGCCACTCCGCCAGTTCGAGGGCGGTAAAGATCTCAGCCGCC
GTGTTTCGGGCTATCTCCCCGCGATCCCGGGCCACAATCGCAGGTGGCTGGGGCGTCGGCCAGACCCGTCTCGTCGGCCTTTCTGCGCACATTACCTCCGA
GCAAGGCAACCGCTCCGCCCCATTACAGGGGGTTGTCTGGTACCTCCGTCACGTACCCCCGCATCGCTGGCGGGCTGACCCAAGCCCCCTCCCATCCCGA
CCCTCACGCGCTCACGCCCCTTGCCGGGCGGGGTCGTGGCTGCCCAGGGTCCAGGGCCCGCCCCGGGGGCCGGTTGGTTCCAGGAACTGAGGGGGGGTC
GCCCAGCTGACCGCTCCTCTCGCGGGGTCTATTCGGCGTGGCCATCCGCAGGTGCGACAATGCCGGCTCCCATTGCCGGGGCGACCCTGGTCCCTGGCGC
TTCGGACTCCCGGGGAGCGCCTCCCTTCATCGGCGGCCCCTGCCCCCGTCATGACCCCCCTTACGCGCCGGTGGTAGCCGCGCGTGGTGGCGTGCCCCTCT
CCATCATTACGCTCCACAGGAGTGGCCACCGGTATGGGCGCGGTGCAGTCGTACATGTGTGCGGCCCTGTTTTGCGAACCCTTCCCGCGGTGACCTCCGC
CCCAGCGGCCTTCTTCGACCGTTAACGCCGGCCAGCACCCGAAGGGGGTCACACAAAGCCCTGCAAGGATGCGACGCTGCGTAGCCGCAGGTTAGCCGC
TTCCTTCCCAAAACAGTGTCAGAGCTGTCCGGCGGAACGTTAACGCCGCCGGGAGGATCTGCGGCTACCCTCAGGCCCGTCCCGTGGGAGTGGCCGGCG
TCGATGGGGGTACGCACCCCACTAGACGCGGATTTTGGCCAACGGGCACGGCCGCTTCCGAGTTCCCCTGCGGTCTGAGTGTTGGTACGTTCAGCCGGCC
GTTCCGTGGGGCAGAGCGCCGCACGCATGTCGCAGGAGCGCGCCCTCCGCGTCTTGCGGGCGGGGTCCTAACGAAGCCCCCTGGGCTTGGAGGTAGGGG
GCGCGGGGGAGCGATGGGAGCGCAAGCGTGCCATCGGGTCCGGCCCATAAGTGTGTATGGCGCACCGCGCTGCCACGATGTCCGGTGCGCGGGCGCCG
CTGGCTAGCTCACTCTCCCCCGACTCCGCCGCCGCGCCCCGAGACGTATGGAACAGCTGGCGCGCGTCCCCCGCCGCGGGCGGCAACGAACTAGATCCA
CCGCCCTCACGAGGAAGCGCGCCGCCACCACGTCTCCCCGGCCGACGCGGTCCCACCATCCGCTGTGCCGGCGCGCGAGCAGCCTGGCCCTCGGGGCGG
TCGCGCCAGCGCAGACACCCTGTCACGCGCACGGGAGCCCTGGCCACCGCGTCGACGCCCGGGCTAGCCCCGACTATCAGTACTGACCAGAGGGGCGC
CGGTTGTCCCGACTGGTTCCTCCCGAAACGCTGGCGGTGGGCCCGCACGATTGGTGCAGGCCCCCAAGAGGATACGGCTATTCGCGGCTGCCCGAGGCG
AGTCGGGCCAGACCGGTCGCGCGTGTGTGTGTTAGCCCTCCGTCGCGGCCAAGCCGGCGGCACCGGACTGGGCGACAGTAGCGTTCCTCTGTCACTAGTC
ACTCTAACGGACAAATGGAAAAATGGCACCTATCGCTACCGACGGGGTTTTCCTCCAGGATAAGCCTCCGCGCCCCCCACTGGACGCCAGCCATCCTTCC
CCGAACGGCTCGCCTGTGCCGGCTCCAACCCCGCGGGACCTCCCTTCGCGGCCCCGGCGGGGATCGGTGGATCGTCGCCGCCGGCGCCTACCGATCGCG
CCGCCACGGTCGTGCACCAACCAAGCACTGCTACACGCCTGTGCGGCCGAGGCCGCCCCATCCAGCAACGGGGCACCGTTGACCCGTCCCCCATGATGC

CGGACATGGTAGCCCCCACACATGCATCTTCGGGTAACCCGCGCCAGGTCCCCCCCCGCGCCGCCGCCGGCTTTCCCCCCGCTGGTGCTAAGCGGCAGAT
GCACCACTGACGGGACACACTCGCCCCCGATCCAGTGCGCTGCCGCCTGCGGCCCCCCACCGGACTGGTGCCTGGCGCTACACGGAGCCGGCCGCGCAC
TGTGACGTATGGATGCCCGAGGGGACATCCGCCCCGGACGCGTGCGCAAAAGACCACTCGCTCGACGCCACGACGTGTGCGGCCGGGGTGATGAACGT
CGGCCGGTGTGCCAGTGAGGCTTGCACCTACACTCCCTGGTCCACCGGGCCGTTCGAGCGGCACCGGGCGAAGAACCCGCAGCCTCCCGGCTTACGCAC
CGGCCTCGCGTCAACAGGCAACCACGACCGCAGGGCCGGCAGTGTGTGTCGCTGTGGAAAGGTGCGCCGCAGGCGACCCCCACGTCAGCCCACTACAA
AGCGTGGCAGCATGCTGCGCAGCGACCGCACGCCCCGACACAGCCCGATGCACAGCATCGGGGGGGCGGAATCGCCCCACGCCCGTAACATCCCGCGA
GTCGACCGCCTTACACGTCGACTGCCACACCCTGGTGCCGCAGTGGTATCCCGAGACCGCATGACCCGCCGCGAGCCCGCTGACCACCTGTCCTTGATCG
CAGTCGCCACGGGGCCGGAAGGTGTACCGCATACCGCACGTGCTGCGCGTGTGCAGCACGGGGGCACCCTGCATGTATGGATCCCTCTAAGCCGACCTG
CCACCCCAGGCCAGAGGCTAGAGTCTGCCGCCTAGGCCCCCCCCCGTGGTCGCCAGCCCCGGTGGCGGCCCACCCTCGGGGCGACTGACCGGCCACCAT
CCCGGGTAGAGCGGTTTGGCCCTAAATACGGGCGTGCCGAGGAGGTGTTTGGGGCGAGCGGCAGCAGGTTCGGCGGATCCTTGAGGGTGGGGCCTGGGG
TGGGACGGGGGCGGCCGAAACGCGAGCATAGGCCCCTGCCTGGCCCCTTTGCAGTCTGTACTTTTATTGACATGATACTAATACCGTGTTCCGCCCTGAG
ATCGCACGCGGACCTACCGTGCCTCTCTCTGCATAGTGCTTCAAGAGCCCAGCGCGACTGGGGACCGGCTCCCGCAGCCGGCTCGGGGCGTAACGCTCG
GTGGAAGGGTCACCCGGATAGCTCCCGGTTAGTTTCGTACAGGGTGTCGGGGGCGCCCCCAGCGAGTCTCCTGGAGGTGCAGGGACGGCGGTGGTCCTC
GAGGGGGCGGACGTTGTCCGCATAACGGGTAGTCTCCAGGGTCACCTATGGTCTGGCTCGCCGTCAGGGTCTGTGAGCGTCGTGCTCACGCCTCCGCAAC
AGCCGGCGACCCCGGCTCGCAACGGCCAGCCGTGGGCGGGGGGGCGACCGGACGCAGTTCCGAGTCTAGGCATGGTGCCACCGCCCTCTCGTTGGGTTA
GTCTCGAATGGCTGTTTCGGGGGTCAGCGCGGCGGGGCCGCCGCTGGGAGCCCGCATAACCGTGGATACACTTGCTGGGTGCGCGCTCGAGGGCGGCCC
CCGAGGGCTGGAAAACCATTTCGGGATCGCCCCCGCGCTCCCGTCCGCACTGCGGGGTCCAGCCCGACGGGGGCGGGTTTCGTTGGGGCAGTACTCACG
CCCACGGCGAGGCCCATCCAGAGAGTGAGGGTGTCGCTGGGGGGGCCTACCGGGCGGAACGCGCCCCGACTTAGGGCGCCGCAATGCACGACCAGTCA
CCGTCTGGGCGTCGCCGCGCCGTTGAGAACCCCAACACAGAAAGAGCTTGCCGCCTCTGGGGGAGCCAGGCCGAGAAGCGTGGGCGAGCGGCCACGCT
GCGGGCCCTCCGCGGACCGTGCGGCGCTCTCGGGTGACAAACCTAGTCCCCGTCCCAGGCCATGGGCGAGCAGCTGCGCCGGGACTGTGCGACGCCTAT
CCGGCACATTCGGTATTATTGGCCTGGGACGACAGCCGGCGACAGGGCGGACGAGGCGGCGTGGACTCGGGGTGCAGGGACGACGTCCACCCGTACTG
GTCAGTAGCCCAACGCGGAAGCCTGTCTGGGGCTTTGGCCCCTAGACATCGGTCCGGGGCGGGGCGCGTCATCGCTACGTTAGTGAGGGGGAGTCTGCA
CCGATAGGGTGTGGTGGTGTTGCTTAGATCGTTGACGGGCCGTGAGGTGGCGCTGCTGTTTTCCGGCTTACGGCTTACGACCTACCGCCCGCCGCGCCGGC
CCGAAGGCGGCGCCCCCGCACATCGCCCTTGGGTGGTAAAACCCGCCCTGCCGCCGCTACCCCCCCCCCAGGGGTGGCCGGAACGGCGAGGGCAGGAC
ACGGTCGACGCACGCAGCGCCGTGACGCCCGGCCCGCGCTAGGGTTGGCACCCGTGGGACCTCGACAAACTTTCCGTACGATTGGGGGGCGCATACAGC
GGACGGGGAACCCGCCCGACGCACGCGCGGTCCCGAGGGGTGGGGGACCTGCAGCTGCGTCGCGCGGTCCGCGAGGGCGTCTTGCTAGACCATGGGCG

TTGCAATAGTGGGAAGGTGCCTCCCAGGCTGCGGGGCGCCGGACGCCCGACACCAAAGCTGTGCGAGGACAGAGAGGCTAGGCACGGATGGGTACAAG
CGAGCTGACAGCCTCCATCCTTGAGTGAGCACGCCGCGGCGAAATGCCGGCCGAAATGGGAGGGGCGCGACACCGGATCGTGATCGAGCGCGTGCGCT
TCGCCCACACGCCCTGACCCGCTGAGTGTCTCGGGTTTGAACCCCGTGCCCGGGGGCGCAGTGGGATAGGCACGACGGGCAGAGTTAGGGGCCTGGGCA
TAACGGCCCTGTCCGCGGCCGAGCGGGCCCGGGTCTTCTCGACTAGGGTGCCGGCCCCGGCGGGCCCCCCGCCCACGGACGGGGACCTGGAGTTGAGCC
AGGCCACAGGCGTGACCGGAATTGGAGCGCCAGGGGACAGCGGCCGCCCGCGCCAGGCCACGCCGATGGGGGCGTTCTGCCCAGGCGTCGCGCAATGG
GCGGACCGGTCTACGCGACACCGCCCGCCCGGCCTGGAACTCCCCCCGAGCACATTCGATATGGTCCGCATCAAGGCGGGAGGCATCGCCGGGGGCCG
CGCGGACCACCACCTTCGCGGGGGACGGTGCGGGTCGCGACGAGGCGGCGGCATGCGCGCGGGTGCCGTGAGCCCTAGAGTCAGCGGTTGCTGGTATG
GGCGGACGGGCGCCCCCGACCTGCCGTGGACGCAGGCTCGCGGACTAGTCGACACGCCATGGGTGCTGTGCGTGACGTTGGCCTTGGTCCAGGGCGCGA
CGCGCAATACCGCGCGGGCTGAGTATCGCGTCCACGGACAGGCGGCTGAGCCGCCCGGCGTAGCTGGTACACGGTCGGGCCGAAGTGGCAAAGAGCCG
GCTTGCGCCCCCAGCGCCGTTGCAGCAGGTCTGGACAGGCAGTACGGAGCGAACGCGGCGAGACACCCGGCTCCGGGCGTAGTCGGATCCGCCCGCCA
GCGGGCGCGCAGCGGTGTCGCGGGCTTGATGCGCCGGTCAGGGAGGTCGTCGCGAATGAGCCGGGTGGAGGGTGCAAGAAGCCAGACGATGGGAGCAC
CGAGTTGTCCGCGTGCGATTGCCGTCCCGGCGAAAGCGCCAGGCCGCGGCGCGTGTGGCAGCGGCAAGTACCCGGAAACACGGCACGGGTCTCGTGGC
GCCGCGAGGCGGCGGGTGGCGCGCTCCGGGGGGCTGCGCAGCCCCTCAGCGCCGCTGCTAGTGCCAGACGCTTGCCCTTCTTGAGCGCGGCGGCGGGAA
CTCGGTCGCGTGCGTTCAGGGCCCTCATCCGCAGACGTGAGGGGTGCGGGCCAGGGGGCGCAGAGGCGCCTTCCGAAACCCGTAAGGGATGTTCTGGCG
TTGGGGGGCCGCCGTCTTAGGGGGGTAGCGCCGCCTCAACTCCAGTATCGCCGCAAGGCCGGTGCCAGGTTCGATCCGGGGGCGGCGGCGTACCGCGTC
CACGCGTCTCCCAGAGGAGGGTCAAGGTGCATACGGGAGGGATCATACGGCAATCGGCGGCCGGGTTCCGCCATCTAACGTGGCGATCGCAGCGTACC
AGAGCGAGGCCTATGGGCCATATCCCTGGCGGGAACGGGCTGCTTGCGCCCCGTCCTCGCCGACCCTCCGTACTGGTACGGGGCGAGGATCCCGACACA
CAGCACGGGGGCGGCGCGCAGCACCCACCCCGTCCTCGGCCCAACAGACAAAGACCCCGCGGCGGGCTGGGGCACCCCCTTTCCGGCTACGTCCACCG
ATTCTGTACAGGACAGCCGTTGCGATGGGCACCGGCGATCGGAGAGAAGGACGCCACAGATCAGCAGACGACTCGGGGAAACCACGGGACCGCGCGTC
GCGTCAACTGCCGCGGGAGTGAGAAGGGAGCGACAGGAGCGCGGAGGTAAGGCCAATGGGGCCAGTTGAAGCCGCCCACAGAGGCTGTGCGGGGCCC
CGCCGGGTTTAGGAGCTGTTCTTGGAGCCTGTCGTTAGGGGCGGGGGGGGGCTTACACGGCGCTGGGGGACCCACGAGGCGCAATCCGCCGCGGACTGC
TGGAGGGCTACCTGGGGCAGCACCCCAGGGGCGGCCATGGCGCTTGTTGACGGGGGCGCTAGGACCTTGTCCTAGGGCGCGCCGCTTTCGCCCACAGGG
CTTCGGTGGGCAGTCTATCTGTGCGGTTTCAGTCTCGTACGCACGTGATGCGGTGTAGCCTGAGCCCGGCAGTTGTCTAGAGTAACTAGCGCGCGGTTATG
GCTTCGTCTGGAAACGGGCTGCAATGTTCCGGGTGTTCATCACACCACGTTCCGCTTTGCAGCTCACCCCCCGACCACACCCGGATTCGGACCGGTGGTC
CTTCCTCTGAGGTACTCGCCGCTCATACAACCGCGGTTCCCGCTGTGGTCGGCACGGCCCGGGTTGCAGGGGCGTGCCTTCCCGGGTAGTGTGCCTGCGC
GGCGTGCGGCTTCCGCAGTCCACGGACCGTTCCGGGGGTGAACGATGGAGGTGAGGGGACGAGTCCCTCCGAGATTAATGCCCCGCGGCAAGCCCGATC

AGCGCTGCGGTCCTGGGCTGTCAGCGCGCGGAGGAGCGCCCGATCGGGCGCGGGGGAATGAGAGTGTGGAGGATCATAATAGAGAGATCGCCCCGG
AGACTGCGAGGGGCATGGACGCGGAGGGACGGGGCGGGGGGTCGGCACGCCCGTGCGGCCTGTCGTCATATCCCACGCTGTCACTCGGCTGCGTCCGGC
ACTCCGGGCGGCCGGCGAGCAAAGCCACCCTGCGCGGCCCGATCGAGGGTGCGGCGGCGCGACGTCGTGGCGTGGGAAATGCGGCGAGGGGCGGGGT
GGGGGACGTCATTGAACCTGCAGCGCGCACGGTGAGTACCGCGGCCCGTGGCGGGGCTGTACTGCGGGACCCCGGAGCGGTAGCGGGGGTTAGACTGG
GTGAGGGTAACCTGCTGCCCCGCGCACCACGTTCTTGGCCAGAGGCCCTGTGGGTGTTGGAGCAGGGTCGAGTATCCCTCCGTGCTTGCCTGTGTGGAGT
GGTTCGCCATGCAGTTGGACTCTTGGCGTCAGCTGGGGCCAGGCGGCGCCTCCGAGGTCGTAAAGGTCAACAGCGCTGTGAGATGGGCGCAGGAAGAGC
GCCAAAGACCTCCTGCGCCCCGATGTCCGCCTCGGGCGGACGTTCGGCCGCCTCGGGACTAGTTAAAGTGCGGCTTGGCTGGGGGCGCGGGACTCGGAA
GTCCGGGTGGAAGATGCGGGGAAGCTGCCCGTCGCCGGTACTGGGGGACTGTAGCTGTGATCCGGGACCACGAGGCATGCACCCGTCGTGGTCCAGCCG
GCCTGGCGCGGGGTCACAGAGCGAAACGGCGGACGCTAGGAACATTCGCTACCACCCCCCGGCGGCACCTTGCAGGGTCTACCCCAGTAAGGACCGGC
CCCAGCAGTGAGTGCGCCCGGGGGCTGGCAGCGGTAGATTGGGTCGCACGAGCTCACGTGGGCCGTTGACCGGAACCGGCAACGCCTGGGACTGGAGC
GCAAACTGGGGTACACTCTTCACGCCCGCTGCTCGTCATCGACCCCGGCGGGCGCCCGGGACCGGACCGGGGGCGACCCACCGGGGGACGGCGCGGCC
CCTGGATGGCCCTGCCCTCGGTCACGAAGCGGCCGAATGGTTTGGGGGGCCTCGGGACGCGGCCGTGGAGGTGCGGGGGGCCTGAAGTCCCTGCCTGGC
CCTCCTCCTAACGGCTTCACGCCCCTCAGGGCAGCTAGGGATTAACGTGGCCGGGTCACACGACCCGCCCAGCGCGGTACGCTGGGCCGCAGTGATTGC
GCGCGCCTGGCGGGTCCTCACCCCCCCACACACATCCGGGTCGGGGCGGAACTGGGTCCCGCCGTTCCCTCAGATCTGCCCCCTGACTTGAACATGCCCA
AAAACCGAAACCGGGCCGGCGCCCAGACCCCCGTGCTAAGGTTCCCACCGCCGACCCTAGCCCCCCCGGAGGTCCCCTGTACTCCGCACGCGACGTCGT
ATCAGGTCCCAGAGCCCGACAATGCATGCGCGTCCCGCTCTGCGCCATGCCCATTGCCCGACCAGCCCCGCCCAGCGCAGAGGCCCCGACTGCTGGCGC
CCTCTGTGGCGCTCGGCCGCTCGAGAGCACCTGCCGCCCGACCGCAGTCTGGCTACCTGCTCCACGGGGCGGCGCTCGTGGTCTCCCGTACTCGGAACGC
CACGCCCCGGGCTCGCGCGTCTCGCGGTCCGACGCATCGTGGTCTTACCTCCGACCCTTTCCTGGGGTCGAGCGCGGGGGGGCAGAGCGCGCTCATCCAG
CCCCGCGCGGCAGCACCCGCCCGCCCAGGGTGGTACCGCTCTCGAGAGTCCGGACGGCCGAACGCCAGGGCAACGGGCCGTGTGAAGACTCACCGTTT
CGGCCGACCGCCATCCTAGCTGGGGCGAGCCCCCGCGCGCGGCCGGATGCGTATGACGTGCGCGCGCTCGGTGCGATGGCGCACGTCGACTGGAATAAC
CCCTTGCCAACCCCAGCGCCGAGAGTCCCTCTTCGGGTCTCCCGTCTGCCTCCGATGGGTGGCCCCTGCCGGTACTTGTTAGGTCCTGGCTCGCCGGGCGC
CTCGTCACCTCGGCAGCCTGGATCGCTTGTCGCCGCCGCAAACGTGCGCGCGTTTCGCCACGGGCCGCCCCGCGAAAAGCGGCGGGAACGTGGACCACG
CGCGCGCCCGCCATCGGCTCGCCGGGATCCCCACCGCGGCAGCGCCCCGCCACAGCTCAGGACGCGGCCGATCGACAAGCACATCTCCCGACTACCGG
GCCCCGCGGCGTGAACGTGGTCATGGAAAACCCGAGCGTGTGCGTTCCTGTCACGGGATCCCGCCGTGGGCAGTTCGCGGCAGGGGCCTACCCGTCGAG
GGGCTACACCGGACCGCCCCTCCAGCTGCATCGTGCAGCCCTAGTGCTGCAGCGAACTTGCCCCGGCCATCCCGTCCGCCCGCAAGCGGCTGGGGACAG
GTTCTCCGCCCATGCGCAGCCAGTGGCCTCATTCATGAGGGCGTGGCGGGCCGGGCCGCGTTGGGCCATCGCCGGTTGGGGGCCCCCACGCCGTCTACCT

GAGTGGACGCGCCGGGCGAGGCTCCCACCACCCCAGTCCTGGGGTCCCAGACGGCCTCCTGGGCGGAGTGTTCTACCCCGCGGCCACCCCCAGCCCGTC
TGCGGCCGAGGCCCCACTGGAGACTGCATTCCCCGCGGCCGGGGAACCGGCCACACCGGCAACGGCGCTCCTGACCCCACCCGCCTGCCACACTCCCCG
CGGCGCGGAGCCCCACCCATACTAAGGCCCCTACGCGCGGCCGACGACATGTTGATGGCTTCGCTGGGGGGGCGGTGACACGTCCACGAGGCGCCTCGC
CGCCGTCGGCCCTGCCCCAGCACGTTCCCCCGTCCCCCGGGGGTGCGGCGTCCTGCTCGCCCCCCGTGTAGGAACGCCGACCGGGCTGCGTCGTCGATGA
GGCAGTGGCCCGCTACGCGGCTCCTCTTCGCACGTGCTTTTGGCTGAAGAACGGGCACCCCCACCATTACGCAGTTGGGACGGCGCGCGGCTGCGAGGG
CCCAAATCTCTCCCATGCGAGTCGTGGTCGCGCCCGTGGACCTCTGAGGGGCAAGGCCGCTCGTCTGTTGAGCGGGGATGTTGCGCCCAGCAAATCGAG
GAAGGTTTGCTCGTAACTTACGGGTACCGAGACTCCTCGCTTGGCTCACCCATTGCGGCAGCCGCGGCCGCCGCCGGGACCAGTAACGGCGTGTCGACC
GCCCCTCGACAACGCCTGGTAGGACGCTCCCCTCCCGGCCCGGACACTAACCCCCGCAACAGCGAATTCCAGGGGCGTCTTCCGCCCAACAAAGACCCA
TCGTGCACGAATTGGGCTGGCGGGCAAGCATGTGCATCACGGACTAGGGTACCGCCTGCCTCCCCGCTGCCTCCGGGGCCCTGGCGCTCCGTCCGATTTT
ATCCCGCTCTGCTGCGATACTCAACCAACCGTTTCGCTTTATTCAGGCCGCCTCTGGGGCCCGGCCGAACACTTTCTCCCGACAAGTGGCGTGACGGACCT
GCCTTCGCGCCGCCCCGAAACAGGCGGGCCGGCCTCGCACCCCTGGGGAGCGGTCCGCCGCAGGCGGGCCTCCTGAGCGCGCGGGTGGCCGGCCAGCG
CGCATACCCCCTCGTCGCTGCCGCCCCCCCCGCCCCGATCCCCGGCCCCGCTCGGCAAGACAGTGGAACCCGCCGCGCCCTTCACTCGCCCGGCTGGCCG
TATTTCGTGGGTCCTCACCTCAGTCGCGTCTGACAGATTAGACCTCGCCGCTGATGCCTAGGGTGGGACACCACCTTCCCGCCCGCCACCACCCTGTGGA
GCGTGAGCGCACTTAAGTCTCCCTACCTCCCCAACTCAGCAGCGGCCCCAGGGCATCCGCGAGGCTCCGTTGACGCACGTGCCGTCGTCCCGCGCCCACT
GTGGCACCTCGGCGGCGACCCTCCGCTGCGGGATGCCTGGAGGAGCGGTCCAGCCTGGGCGCTCTGCCGGTCACCAACGGCGGCCGGGCGGGGGCCCTC
GCGGTCGGACCGAACTTCGGAGGGGTCCCCGGCAGCTGAGTCGAGCGCTGCTAGTGACCTCCACCTCGGCCTAGTCACACTTGCCCGGCTCCCAACCGCT
AAGACATAGGGGGCGTCGCGACGAGGGCAATGCGGGGGCAACCTTGCCACCCCCAAGCGACGTCGCCTCATGTAGGTTGGAGGGGCTGTCTTGTACTCC
ACCGAGGGCGACACGTAGCGTGCGGCGGCTCGACTCGAATCTTGCGCCTCGGTACGCGCCACGCGACCGATGACCTTTGCAGGCGACTCTGCGAGAATG
GGGCCCGCGTCCTGGGGCGTGCTAAATCACGACCGCCGCCGCTGGCCTCCAGGAGGTCCCAGGGGGCAGCACACGCCATTCGCGTCGGTGGTCTAGCCT
CGCGGCACCTCGACGAGTCCCAGGACGCGTCGGGCCCGGTTCTTCCTCTTCCTGTGTGGCAGTCCGGCCGGGTGACGCTTCCGCCGCTGGCTAATTCAGA
CCGCCGTCAGTCGACTGGGCTGCCCTAACCAGCCAGGGCACTCGTGGACTTCCGGCGCCGTCCGACTCCACGCGGCGCGCACAGTCGCCTCCCTCCTTCG
TACCATTCGGCCCTCCTCACCCCAGGGGGGGCTTGCCGCCATTCCCTACGCTGCCATTCGACGTCCCGCGCTACTCACCCATAGTGCCAATTGCGGCGTCG
CGGACACCGGCCTGGCGCAGCGCCAGAGCGCCACCTGTGACGTCGCTCACCGCGGGGGGAGCCCAGCCTCGACGCCCCAGATGGCTCGCGGGGGGGGA
GGTCCCGAGGCCACCCTTCCAGGTGGCACAGATGCCCCTCCCTGGAGGGGGTGGAAAGCGCGCGGCGGGCCCAACACCGTGGGCGGCAGCTGGGCATG
CGCTCGGATAAGAGCGCCAGGATGGCAGGAGGGCCGATCTGGCGGGTGGTGGCCAGTGCGGGGCGGGGGGCGAAACTGTCTGGGGGTACCTGGAGCCC
AGGGTTCAGGGTCCGTGCCATGAGGCTGTCAGGCAAGCCAAGCCGTTAACGGCGGCCAGCGAGTGGGCAGGGAAGGTGACTAGCGCGAGCTACATATT

GCCGGCCGCCGCGCGGGGCGGCCAGCCGTGAGGAGCAATCTGAGGGACGGCCCACCGCCGGAGGGCCTGCACCATGAGGTGAGGCGGCGGGTCCCCG
CCACGCCGCGAGACGGTACGACCCGTTCCGGATGTGCACCACCGATGAAGAAGGCGCGCACATGCCGTTCGCTTTCGGCGTATGGTCTTCGCAGCCTCCG
GTGGGAGGCGCAGCCCGGAGGGTGCCCGCGCCACACGGATCAATGGGTTGGAGGGGCGGACTCGTGGTTTCGACCATTGGTGCAGCTAGAAACCGTCCC
AAGGCTCCACTCGATCGCACTGCCGTCGATCTCGGTTCTGACTCCCACCTATGCCAACACGGGAGTGCGCTATGAAACGCGGTTTTATAGTCGGTAATCG
CGACCGAACGCGGAGCGCCGTCTCGAGGCTTGTAACGCTCCACGGTCGTTGTGGAGCGGACCGGGTAGTCAGCGTGACGTATTTCTAGCCCCCAGTGTGC
CGCCCGGCGAGAAAGTCCTCGCGGGAGCCCGTCGGCTGCCGCTGCGTACGCGGGGGAAAGCGGCCGAGGTACGTCTTCCTGGTGGCTTGCTGCCGCCAA
CCACCGATCGGCCCGGCTCGATGACAGCCGGCGAGAATGGGTTGGGGCCTACTTGGCCGGGTTGGCTGATCGTACGCAAGCAGCGTGCTGCGGGCCCAC
GCAAAGGGGAATGGCGGGGGAGCGCCGGCTGCCAATTCCCCCCGGCTGCACAGGCATGATCCGGTGTCAAGAACGGTCTCCGGCCCACGCGGCCGCGC
CTTGGCCCGAGTACGGCTGACTCAGAAGCCGTTGGGTGCGAGGGCGTGGGGTGGTGGCGCATAGGGAGCCAGACCCGACGGAGCCAGGATGGTTCGAG
CAGTGTCACGGCCCGGGGTCATGTGGGGCCCGCCCGCACGGATGACCCGCGTCTCGATAATCGGTTGCAGGCGTGCCGTTCCCACTGGGCGCGCCGGTTA
TCGAACGGGTGCGCGCGTAGACTCGCTTCCGGGCCCCGGACCTGGGCCAGGCAGAAGCGGGGATTACCCGTCTTGATCGTAGACAGCCCGGTGCCCCCG
GGGGCCGACGTACAGCTGGCAAGCACCGGTCCGGCGATTACCCGACCTGGGTTGGCAGGTACACGTTACAGGGGTGGCGTGCCCCCCGGCGCTTGGCGC
CGGCTTGGAGGGACGGGGGGGGATCTAGCTCAGATGGCCAGCGAGCGTAGCTTCGACGGTGGCAAGAGCGAGCGGCGGCGTGCTTGGGGAGGCCGCGG
GCGGGTGCGACGTCCAGTTCGTCACACGCATGAGGGGTTTCCGGGGAGGAATCACCGCCTCGCGCAGGCGTGTCCGACACGACCCGTGTAGCGGGTGTG
AGACATTTAGGCGGAGGCCAGAACCCCTTGCCGGAGGGCTTGTAGTATATTGTCAATGGGGCCGACACCACGTGCGGATCGGGACTGAGAGCGCGTAGA
GCCTTGGCCCTTGCTATATTTGTGGAGCGGACACTGCGCTTCCCACACGTCACGCTGGCGTAAGCCGATGCCCGTCACCGCCCGCGCCCCAGGCCGAGTG
CGGGGGGCCGCAAGGTCCTGAAGCCGATCTACGATGGGCTGGGTGGGCGACGTCGTGGGCTAAGCTCGGCGGCCCCACCGTGAGGCGGACGTGGGGGC
CTGTACTAGAGATGGAGCGACGGTCGTCGATACAGTTCTTGCCAGGAGACCGAGCATTTTGCTAGGCACGGGCTGCATGGGCAATTCATTAACGGCGGC
GGGCCCACCGCGGCCAGCGGTGGCGGGGCGTAGTACCGGTCGGCAGGGATACGACCCCGGGCCGAAATTCGGGGCCGGCGGCCAGGGGGCGGCGCGC
CTGCGCGCCGTGACGCTGACGCCCTCCCGGGGTGCCGCGGGCTGGCCTGAGGCCCAAGGAGGCGCCTGGGGGTCGAGCGCGGGCCCGGGCGCACGCTG
ATCCCGAGCCGACGAGCTTACGGATTGACCAAGAGCACCGCGAGCGTCCGAGCGCTGCGGGCGTGGTAACAACTACATCCCGGGTCGAGTTTGCGGCTG
GGACGTGGGATTGGCCGTGCAGACGCGCTGGGGGGGGAGCGGGGCTGGCCCCTCGCTCCGTATTCCCCACTCCACATGGTCCCCGGGCGTACGGGCGCC
CTCGACGGTTCGGGACGCGGGAATTGCGCAGGTGCCCCTCGGTGCGTGGCGGGCTGCGTGTAAGGCGCTTTCGCTGCATGCGGCCGTCTCGCGGACATGG
CGTACGGGGGCGACCAGGGCGAATCCAAGGTGCGCCCAACGGGCCGAGGCGCTACAGATGGGGAGCCCCGGGCGGCCGTACGCAGGAGTCCAGTGGC
GTATGGAGGGAGGCCTGGGGCTCCGCAACGGCCGAGGCTATGCCCAGACCCCATGCCATTGTAGGCCGAACGCGATAGGTCACGCCGGTGATCCTGCCG
GGGTCGCGGGCCGGATCCACAAGGCCAGACCCATAGCGTAGTCTATAGGCGTCCGGGAAGGGTCGTGGGGGGGCTAA

>2017.TE.25009.1.10

CACGCCCCCCTCCCGGCCAGCCCGCCCCCCGCCCCAGCGTCGTACCCGGCGTCGCGACTCCCCGCCGTCGAGAGGCCTTTGCCGGGGCCCCGGCTAGCC
CGGGGCGAGCGGACCGCCGCGCGCGGGGGGTGTCACCCGGGGCACACCGCGCCAGCCTCCCGGGCATGCCACGCCGGGTTCCGCCCCCGCTGCTCCAA
TTTGGGGGGCCCCTAAGATCCCGTTCCCCAGGCCAGCCGCGCCCGCGGGCCAACATTGCCACACCGTGTCGCCCCCCCGCCTGCCGCACTGACCGTCTTG
CGGGCCCTGCTCCCTGGCCCGAGCACACCTCCGGGGACCCCCCTCGCTCGGCCGGGGGAGCCTTAAGCGCGGCACCGAGTAGGGGGTCGCGTGTTGCCG
CCGCGCTGACGCCCCGGCTGGGACCCCTTTCAGGTCGAGACGCCGGGGTGGCCATGCGGGTCCAGTCGCACGATGGCGCGGCGATGTGGCGCCGACTGC
CCCGCCTGTCTTCTCCGTGCCAGCCCCTCGCCACCATGCCGGTCCGGCCCGCACGGGGGTAGGAGGTGCTGCCGCGGCGGGAGGCGTAGGACGCCATAT
CCGAACCGGCGGGTCCCCCCGGCCACCGAGTCGACAGGACCATAGGCACCGCGTCCGGGCGGGCGCCCTTAACGCCGCCCCTTACTGGAGAAGCAGAG
CTCTGCCGAGGCACCCTGCCTAGCTGGCTGCGTCCCGCACCCCGCGATCCCCCTGGTCCACTTACCCAGCGGGATGCGGGGATGTTCGCAGCTATCACAC
GGTCGTGGCCTGCCTCCCTGGGCCGGCGAGAGCACCGCCACCTTCGTGTTCCTTGCCGCCGGGCGACGTGTCCGGGAAAGGCTTCGCCGCGCGCCCGCGC
GGAGCGCCGGGGAGTCCCGTGAGCGGCCGCAAGAACGTCACGGACCCCGGCTTGGTGCGGTCCCCCTCAGTGGTGCCCGACCAGAGACCGCGGAAAGA
CTCCAGGAAACGCCTCGGGGGGAAAGCAGGGCCGCGCACTTCCGCTCCACGCTAGCTGGGGGGACCACGACGGCAATACTGAACACCTGGCTCTTCGG
CAGCCCGGCCTATGAGGGGCCCTCTCGTCCCGGCCAAAGGCTATTTGCCTTCCTGTCCGGGCACCCTCGACCGCACCCGGGCGGGCGCAGGCTTCATTGC
GGCGCGACTCGCCAAAAGTCCCCGCGATCTCAGCGAGTACCCTGAGCGCTGGCCCTAGTCCGCCTGCCCCCTTAATGTCGCTCAGGTCTGCAATCTCTTA
CGACCGGCTCAGGTAGTGGCCGCACGGTGCGTAGGTCCGCTGCGGGCCGGGCCACTCGCGCCGATCGTGCCGTGCGGCGGCCCTGACACCGTATGCGTG
CGCGCTCCGCAGGCTCGCGAAAATCCTTCGCAGGCACTTGGCCCCGTTGCTAGACGTGGTGTCTCCTAACTGTGGCTCACGCACGGCGTTTAGGCCACGG
TAGCCCGCTGTTCGGTCCTTACGACTCGGCACACGCCCCCGTGCACGCCCTCGGGTTCCGCAGGCCAACGCCTGGCCCTAGGGTTGCGTGCCGGCCTGCC
CGCGATGCCACTTGCCCCACGTCCCCCACCTGGCCGGCGCAGACGGGTTCAGTGGGTGTCGAACGCCATTCAGTCCCCTGGGACTCCCCCGACCCCTCCC
ATGGCACCCGCCGCCTGGCCTTGGAGCTCCTGCGCGTTGGACCTTACTGCGGTCGTCAGCTTCTCGGCGCGCCCCGGACTAGTCCTGGGCGGCCAGCTCTC
GGGACCCTAGCGCGCACGCGTGCCCGATTGCCGTATCCCCTCGACGGCGCCCGGGCATAGGGTTCGATCCCGCTGCTATTGCGGGTGCGTCCAGACCTGC
CGCCGTTTGGCGATCCGAGTGTGCGAAGGGAGCCAGCCAGCGGGCCGCCTGGCCGCACTCCAGGATCCTGGCGCCAAGGGAGAGCCCTGCCAGCGCTA
CGCGAATACTCGACCTGTCCGCGCCCCGCCTCGGGCTTGCGAGATGGCCACTAGACGCTATCGGGTCGTCACCCCGTCGGCCCCACAGGGGCCCGGCTTC
GCGTGGCTTTCTTGTCGAGGGCCCCGAAATCCGGCAGGCCCCGACACCTTCACCGTGAGCGAGGACGCGCGGTGCGGGATGTCCAGTTCATGCACGCCC
CTCCACGGCCGCCTTTGCGCGGACAGCGTCGGGCGTGCCCAGGACGAGAACAGACCCTGGGCCAGCCTCAGGGCCCGGCCTAGGCGACCCTGGTGACC
AGTTTCCACTCGGCACCCTCGGGCTTCGTGGCAGTGCAGTCATGCCCCCGGGTGCTCCCGCGCGACCGCGCACCTCCCACCCAAGGTTCTTGGTCAACGC

GCCGGATCTGACGTCGCGGTCCGGCCCGCGCCGTAGGCAAAAATGGGTTCGGCTCCCGCCACTCCGCCAGTTCGAGGGCGGTAAAGATCTCAGCCGCCG
TGTTTCGGGCTATCTCCCCGCGATCCCGGGCCACAATCGCAGGTGGCTGGGGCGTCGGCCAGACCCGTCTCGTCGGCCTTTCTGCGCACATTACCTCCGAG
CAAGGCAACCGCTCCGCCCCATTACAGGGGGTTGTCTGGTACCTCCGTCACGTACCCCCGCATCGCTGGCGGGCTGACCCAAGCCCCCTCCCATCCCGAC
CCTCACGCGCTCACGCCCCTTGCCGGGCGGGGTCGTGGCTGCCCAGGGTCCAGGGCCCGCCCCGGGGGCCGGTTGGTTCCAGGAACTGAGGGGGGGTCG
CCCAGCTGACCGCTCCTCTCGCGGGGTCTATTCGGCGTGGCCATCCGCAGGTGCGACAATGCCGGCTCCCATTGCCGGGCCGACCCTGGTCCCTGGCGCT
TCGGACTCCCGGGGAGCGCCTCCCTTCATCGGCGGCCCCTGCCCCCGTCATGACCCCCCTTACGCGCCGGTGGTAGCCGCGCGTGGTGGCGTGCCCCTCT
CCATCATTACGCTCCACAGGAGTGGCCACCGGTATGGGCGCGGTGCAGTCGTACATGTGTGCGGCCCTGTTTTGCGAACCCTTCCCGCGGTGACCTCCGC
CCCAGCGGCCTTCTTCGACCGTTAACGCCGGCCAGCACCCGAAGGGGGTCACACAAAGCCCTGCAAGGATGCGACGCTGCGTAGCCGCAGGTTAGCCGC
TTCCTTCCCAAAACAGTGTCAGAGCTGTCCGGCGGAACGTTAACGCCGCCGGGAGGATCTGCGGCTACCCTCAGGCCCGTCCCGTGGGAGTGGCCGGCG
TCGATGGGGGTACGCACCCCACTAGACGCGGATTTTGGCCAACGGGCACGGCCGCTTCCGAGTTCCCCTGCGGTCTGAGTGTTGGTACGTTCAGCCGGCC
GTTCCGTGGGGCAGAGCGCCGCACGCATGTCGCAGGAGCGCGCCCTCCGCGTCTTGCGGGCGGGGTCCTAACGAAGCCCCCTGGGCTTGGAGGTAGGGG
GCGCGGGGGAGCGATGGGAGCGCAAGCGTGCCATCGGGTCCGGCCCATAAGTGTGTATGGCGCACCGCGCTGCCACGATGTCCGGTGCGCGGGCGCCG
CTGGCTAGCTCACTCTCCCCCGACTCCGCCGCCGCGCCCCGAGACGTATGGAACAGCTGGCGCGCGTCCCCCGCCGCGGGCGGCAACGAACTAGATCCA
CCGCCCTCACGAGGAAGCGCGCCGCCACCACGTCTCCCCGGCCGACGCGGTCCCACCATCCGTTGTGCCGGCGCGCGAGCAGCCTGGCCCTCGGGGCGG
TCGCGCCAGCGCAGACAGCCTGTCACGCGCACGGGAGCCCTGGCCACCGCGTCGACGCCCGGGCTAGCCCCGACTATCAGTACTGACCAGAGGGGCGC
CGGTTGTCCCGACTGGTTCCTCCCGAAACGCTGGCGGTGGGCCCGCACGATTGGTGCAGGCCCCCAAGAGGATACGGCTATTCGCGGCTGCCCGAGGCG
AGTCGGGCCAGACCGGTCGCGCGTGTGTGTGTTAGCCCTCCGTCGCGGCCAAGCCGGCGGCACCGGACTGGGCGACAGTAGCGTTCCTCTGTCACTAGTC
ACTCTAACGGACAAATGGAAAAATGGCACCTATCGCTACCGACGGGGTTTTCCTCCAGGATAAGCCTCCGCGCCCCCCACTGGACGCCAGCCATCCTTCC
CCGAACGGCTCGCCTGTGCCGGCTCCAACCCCGCGGGACCTCCCTTCGCGGCCCCGGCGGGGATCGGTGGATCGTCGCCGCCGGCGCCTACCGATCGCG
CCGCCACGGTCGTGCACCAACCAAGCACTGCTACACGCCTGTGCGGCCGAGGCCGCCCCATCCAGCAACGGGGCACCGTTGACCCGTCCCCCATGATGC
CGGACATGGTAGCCCCCACACATGCATCTTCGGGTAACCCGCGCCAGGTCCCCCCCCGCGCCGCCGCCGGCTTTCCCCCCGCTGGTGCTAAGCGGCAGAT
GCACCACTGACGGGACACACTCGCCCCCGATCCAGTGCGCTGCCGCCTGCGTCCCCCCACCGGACTGGTGCCTGGCGCTACACGGAGCCGGCCGCGCAC
TGTGACGTATGGATGCCCGAGGGGACATCCGCCCCGGACGCGTGCGCAAAAGACCACTCGCTCGACGCCACGACGTGTGCGGCCGGGGTGATGAACGT
CGGCCGGTGTGCCAGTGAGGCTTGCACCTACACTCCCTGGTCCACCGGGCCGTTCGAGCGGCACCGGGCGAAGAACCCGCAGCCTCCCGGCTTACGCAC
CGGCCTCGCGTCAACAGGCAACCACGACCGCAGGGCCGGCAGTGTGTGTCGCTGTGGAAAGGTGCGCCGCAGGCGACCCCCACGTCAGCCCACTACAA
AGCGTGGCAGCATGCTGCGCAGCGACCGCACGCCCCGACACAGCCCAATGCACAGCATCGGGGGGGCGGAATCGCCCCACGCCCGTAACATCCCGCGG

GTCGACCGCCTTACACGTCGACTGCCACACCCTGGTGCCGCAGTGGTATCCCGAGACCGCATGACCCGCCGCGAGCCCGCTGACCACCTGTCCTTGATCG
CAGTCGCCACGGGGCCGGAAGGTGTACCGCATACCGCACGTGCTGCGCGTGTGCAGCACGGGGGCACCCTGCATGTATGGATCCCTCTAAGCCGACCTG
CCACCCCAGGCCAGAGGCTAGAGTCTGCCGCCTAGGCCCCCCCCGAGGTCGCCAGCCCCGGTGGCGGCCCACCCTCGGGGCGACTGACCGGCCACCAT
CCAAGGTAGAGAGGTTTGGCCCTAAATACGGGCGTGCCGAGGAGGTGTTTGGGGCGAGCGGCAGCAGGTTCGGCGGATCCTTGAGGGTGGGGCCTGGG
GTGGGACGGGGGCGGCCGAAACGCGAGCATAGGCCCCTGCCTGGCCCCTTTGCAGTCTGTACTTTTATTGACATGATACTAATACCGTGTTCCGCCCTGA
GATCGCACGCGGACCTACCGTGCCTCTCTCTGCATAGTGCTTCAAGAGCCCAGCGCGACTGGGGACCGGCTCCCGCAGCCGGCTCGGGGCGTAACGCTC
GGTGGAAGGGTCACCCGGATAGCTCCCGGTTAGTTTCGTACAGGGTGTCGGGGGCGCCCCCAGCGAGTCTCCTGGAGGTGCAGGGACGGCGGTGGTCCT
CGAGGGGGCGGACGTTGGCCGCATAACGGGTAGTCTCCAGGGTCACCTATGGTCTGGCTCGCCGTCAGGGTCTGTGAGCGTCGTGCTCACGCCTCCGCAA
CAGCCGGCGACCCCGGCTCGCAACGGCCAGCCGTGGGCGGGGGGGCGACCGGACGCAGTTCCGAGTCTAGGCATGGTGCCACCGCCCTCTCGTTGGGTT
AGTCTCGAATGGCTGTTTCGGGGGTCAGCGCGGCGGGGCCGCCGCTGGGAGCCCGCATAACCGTGGATACACTTGCTGGGTGCGCGCTCGAGGGCGGCC
CCCGAGGGCTGGAAAACCATTTCGGGATCGCCCCCGCGCTCCCGTCCGCACTGCGGGGTCCAGCCCGACGGGGGCGGGTTTCGTTGGGGCAGTACTCAC
GCCCACGGCGAGGCCCATCCAGAGAGTGAGGGTGTCGCTGGGGGGGCCTACCGGGCGGAACGCGCCCCGACTTAGGGCGCCGCAATGCACGACCAGTC
ACCGTCTGGGCGTCGCCGCGCCGTTGAGAACCCCAACACAGAAAGAGCTTGCCGCCTCTGGGGGAGCCAGGCCGAGAAGCGTGGGCGAGCGGCCACGC
TGCGGGCCCTCCGCGGACCGTGCGGCGCTCTCGGGTGACAAACCTAGTCCCCGTCCCAGGCCATGGGCGAGCAGCTGCGCCGGGACTGTGCGACGCCTA
TCCGGCACATTCGGTATTATTGGCCTGGGACGACAGCCGGCGACAGGGCGGACGAGGCGGCGTGGACTCGGGGTGCAGGGACGACGTCCACCCGTACT
GGTCAGTAGCCCAACGCGGAAGCCTGTCTGGGGCTTTGGCCCCTAGACATCGGTCCGGGGCGGGGCGCGTCATCGCTACGTTAGTGAGGGGGAGTCTGC
ACCGATAGGGTGTGGTGGTGTTGCTTAGATCGTTGACGGGCCGTGAGGTGGCGCTGCTGTTTTCCGGCTTACGGCTTACGACCTACCGCCCGCCGCGCCGG
CCCGAAGGCGGCGCCCCCGCACATCGCCCTTGGGTGGTAAAACCCGCCCTGCCGCCGCTACCCCCCCCCCAGGGGTGGCCGGAACGGCGAGGGCAGGA
CACGGTCGACGCACGCAGCGCCGTGACGCCCGGCCCGCGCTAGGGTTGGCACCCGTGGGACCTCGACAAACTTTCCGTACGATTGGGGGGCGCATACAG
CGGACGGGGAACCCGCCCGACGCACGCGCGGTCCCGAGGGGTGGGGGACCTGCAGCTGCGTCGCGCGGTCCGCGAGGGCGTCTTGCTAGACCATGGGC
GTTGCAATAGTGGGAAGGTGCCTCCCAGGCTGCGGGGCACCGGACGCCCGACACCAAAGCTGTGCGAGGACAGAGAGGCTAGGCACGGATGGGTACAA
GCGAGCTGACAGCCTCCATCCTTGAGTGAGCACGCCGCGGCGAAATGCCGGCCGAAATGGGAGGGGCGCGTCACCGGATCGTGATCGAGCGCGTGCGC
TTCGCCCACACGCCCTGACCCGCTGAGTGTCTCGGGTTTGAACCCCGTGCCCGGGGGCGCAGTGGGATAGGCACGACGGGCAGAGTTAGGGGCCTGGGC
ATAACGGCCCTGTCCGCGGCCGAGCGGGCCCGGGTCTTCTCGACCAGGGTGCCGGCCCCGGCGGGCCCCCCGCCCACGGACGGGGACCTGGAGTTGAGC
CAGGCCACAGGCGTGACCGGAATTGGAGCGCCAGGGGACAGCGGCCGCCCGCGCCAGGCCACGCCGATGGGGGCGTTCTGCCCAGGCGTCGCGCAATG
GGCGGACCGGTCTACGCGACACCGCCCGCCCGGCCTGGAACTCCCCCCGAGCACATTCGATATGGTCCGCATCAAGGCGGGAGGCATCGCCGGGGGCC

GCGCGGACCACCACCTTCGCGGGGGACGGTGCGGGTCGCGACGAGGCGGCGGCATGCGCGCGGGTGCCGTGAGCCCTAGAGTCAGCGGTTGCTGGTAT
GGGCGGACGGGCGCCCCCGACCTGCCGTGGACGCAGGCTCGCGGACTAGTCGACCCGCCATGGGTGCTGTGCGTGACGTTGGCCTTGGTCCAGGGCGCG
ACGCGCAATACCGCGCGGGCTGAGTATCGCGTCCACGGACAGGCGGCTGAGCCGCCCGGCGTAGCTGGTACACGGTCGGGCCGAAGTGGCAAAGAGCC
GGCTTGCGCCCCCAGCGCCGTTGCAGCAGGTCTGGACAGGCAGTACGGAGCGAACGCGGCGAGACACCCGGCTCCGGGCGTAGTCGGATCCGCCCGCC
AGCGGGCGCGCAGCGGTGTCGCGGGCTTGATGCGCCGGTCAGGGAGGTCGTCGCGAATGAGCCGGGTGGAGGGTGCAAGAAGCCAGACGATGGGAGC
ACCGAGTTGTCCGCGTGCGATTGCCGTCCCGGCGAAAGCGCCAGGCCGCGGCGCGTGTGGCAACGGCAAGTACCCGGAAACACGGCACGGGTCTCGTG
GCGCCGCGAGGCGGCGGGTGGCGCGCTCCGGGGGGCTGCGCAGCCCCTCAGCGCCGCTGCTAGTGCCAGACGCTTGCCCTTCTTGAGCGCGGCGGCGGG
AACTCGGTCGCGTGCGTTCAGGGCCCTCATCCGCAGACGTGAGGGGTGCGGGCCAGGGGGCGCAGAGGCGCCTTCCGAAACCCGTAAGGGATGTTCTGG
CGTTGGGGGGCCGCCGTCTTAGGGGGGTAGCGCCGCCTCAACTCCAGTATCGCCGCAAGGCCGGTGCCAGGTTCGATCCGGGGGCGGCGGCGTACCGCG
TCCACGCGTCTCCCAGAGGAGGGTCAAGGTGCATACGGGAGGGATCATACGGCAATCGGCGGCCGGGTTCCGCCATCTAACGTGGCGATCGCAGCGTAC
CAGAGCGAGGCCTATGGGCCATATCCCTGGCGGGAACGGGCTGCTTGCGCCCCGTCCTCGCCGACCCTCCGTACCGGTACGGGGCGAGGATCCCGACAC
ACAGCACGGGGGCGGCGCGCAGCACCCACCCCGTCCTCGGCCCAACAGACAAAGACCCCGCGGCGGGCTGGGGCACCCCCTTTCCGGCTACGTCCACC
GATTCTGTACAGGACAGCCGTTGCGATGGGCACCGGCGATCGGAGAGAAGGACGCCACAGATCAGCAGACGACTCGGGGAAACCACGGGACCGCGCG
TCGCGTCAACTGCCGCGGGAGTGAGAAGGGAGCGACAGGAGCGCGGAGGTAAGGCCAATGGGGCCAGTTGAAGCCGCCCACAGAGGCTGTGCGGGGC
CCCGCCGGGTTTAGGAGCTGTTCTTGGAGCCTGTCGTTAGGGGCGGGGGGGGGCTTACACGGCGCTGGGGGACCCACGAGGCGCAATCCGCCGCGGACT
GCTGGAGGGCTACCTGGGGCAGCACCCCAGGGGCGGCCATGGCGCTTGTTGACGGGGGCGCTAGGACCTTGTCCTAGGGCGCGCCGCTTTCGCCCACAG
GGCTTCGGTGGGCAGTCTATCTGTGCGGTTTCAGTCTCGTACGCACGTGATGCGGTGTAGCCTGAGCCCGGCAGTTGTCTAGAGTAACTAGCGCGCGGTTA
TGGCTTCGTCTGGAAACGGGCTGCAATGTTCCGGGTGTTCATCACACCACGTTCCGCTTTGCAGCTCACCCCCCGACCACACCCGGATTCGGACCGGTGGT
CCTTCCTCTGAGGTACTCGCCGCTCATACAACCGCGGTTCCCGCTGTGGTCGGCACGGCCCGGGTTGCAGGGGCGTGCCTTCCCGGGTAGTGTGCCTGCGC
GGCGTGCGGCTTCCGCAGTCCACGGACCGTTCCGGGGGTGAACGATGGAGGTGAGGGGACGAGTCCCTCCGAGATTAATGCCCCGCGGCAAGCCCGATC
AGCGCTGCGGTCCTGGGCTGTCAGCGCGCGCGGAGGAGCGCCCGATCGGGCGCGGGGGAATGAGAGTGTGGAGGATCATAATAGAGAGATCGCCCCGG
AGACTGCGAGGGGCATGGACGCGGAGGGACGGGGCGGGGGGTCGGCACGCCCGTGCGGCCTGTCGTCATATCCCACGCTGTCACTCGGCTGCGTCCGGC
ACTCCGGGCGGCCGGCGAGCAAAGCCGCCCTGCGCGGCCCGATCGAGGGTGCGGCGGCGCGACGTCGTGGCGTGGGAAATGCGGCGAGGGGCGGGGT
GGGGGACGTCATTGAACCTGCAGCGCGCACGGTGAGTACCGCGGCCCGTGGCGGGGCTGTACTGCGGGACCCCGGAGCGGTAGCGGGGGTTAGACTGG
GTGAGGGTAACTTGCTGCCCCGCGCACCACGTTCTTGGCCAGAGGCCCTGTGGGTGTTGGAGCAGGGTCGAGTATCCCTCCGTGCTTGCCTGTGTGGAGT
GGTTCGCCATGCAGTTGGACTCTTGGCGTCAGCTGGGGCCAGGCGGCGCCTCCGAGGTCGTAAAGGTCAACAGCGCTGTGAGATGGGCGCAGGAAGAGC

GCCAAAGACCTCCTGCGCCCCGATGTCCGCCTCGGGCGGACGTTCGGCCGCCTCGGGACTAGTTAAAGTGCGGCTTGGCTGGGGGCGCGGGACTCGGAA
GTCCGGGTGGAAGATGCGGGGAAGCTGCCCGTCGCCGGTACTGGGGGACTGTAGCTGTGATCCGGGACCACGAGGCATGCACCCGTCGTGGTCCAGCCG
GCCTGGCGCGGGGTCACAGAGCGAAACGGCGGACGCTAGGAACATTCGCTACCACCCCCCGGCGGCACCTTGCAGGGTCTACCCCAGTAAGGACCGGC
CCCAGCAGTGAGTGCGCCCGGGGGCTGGCAGCGGTAGATTGGGTCGCACGAGCTCACGTGGGCCGTTGACCGGAACCGGCAACGCCTGGGACTGGAGC
GCAAACTGGGGTACACTCTTCACGCCCGCTGCTCGTCATCGACCCCGGCGGGCGCCCGGGACCGGACCGGGGGCGACCCACCGGGGGACGGCGCGGCC
CCTGGATGGCCCTGCCCTCGGTCACGAAGCGGCCGAATGGTTTGGGGGGCCTCGGGACGCGGCCGTGGAGGTGCGGGGGGCCTGAAGTCCCTGCCTGGC
CCTCCTCCTAACGGCTTCACGCCCCTCAGGGCAGCTAGGGATTAACGTGGCCGGGTCACACGACCCGCCCAGCGCGGTACGCTGGGCCGCAGTGATTGC
GCGCGCCTGGCGGGTCCTCACCCCCCCACACACATCCGGGTCGGGGCGGAACTTGGTCCCGCCGTTCCCTCAGATCTGCCCCCTGACTTGAACATGCCCA
AAAACCGAAACCGGGCCGGCGCCCAGACCCCCGTGCTAAGGTTCCCACCGCCGACCCTAGCCCCCCCGGAGGTCCCCTGTACTCCGCACGCGACGTCGT
ATCAGGTCCCAGAGCCCGACAATGCATGCGCGTCCCGCTCTGCGCCATGCCCATTGCCCGACCAGCCCCGCCCAGCGCAGAGGCCCCGACTGCTGGCGC
CCTCTGTGGCGCTCGGCCGCTCGAGAGCACCTGCCGCCCGACCGCAGTCTGGCTACCTGCTCCACGGGGCGGCGCTCGTGGTCTCCCGTACTCGGAACGC
CACGCCCCGGGCTCGCGCGTCTCGCGGTCCGACGCATCGTGGTCTTACCTCCGACCCTTTCCTGGGGTCGAGCGCGGGGGGGCAGAGCGCGCTCATCCAG
CCCCGCGCGGCAGCACCCGCCCGCCCAGGGTGGTACCGCTCTCGAGAGTCCGGACGGCCGAACGCCAGGGCAACGGGCCGTGTGAAGACTCACCGTTT
CGGCCGACCGCCATCCTAGCTGGGGCGAGCCCCGCGCGCGGCCGGATGCGTATGACGTGCGCGCGCTCGGTGCGATGGCGCACGTCGACTGGAATAAC
CCCTTGCCAACCCCAGCGCCGAGAGTCCCTCTTCGGGTCTCCCGTCTGCCTCCGATGGGTGGCCCCTGCCGGTACTTGTTAGGTCCTGGCTCGCCGGGCGC
CTCGTCACCTCGGCAGCCTGGATCGCTTGTCGCCGCCGCAAACGTGCGCGCGTTTCGCCACGGGCCGCCCCGCGAAAAGCGGCGGGAACGTGGACCACG
CGCGCGCCCGCCATCGGCTCGCCGGGATCCCCACCGCGGCAGCGCCCCGCCACAGCTCAGGACGCGGCCGATCGACAAGCACATCTCCCGACTACCGG
GCCCCGCGGCGTGAACGTGGTCATGGAAAACCCGAGCGTGTGCGTTCCTGTCACGGGATCCCGCCGTGGGCAGTTCGCGGCAGGGGCCTACCCGTCGAG
GGGCTACACCGGACCGCCCCTCCAGCTGCATCGTGCAGCCCTAGTGCTGCAGCGAACTTGCCCCGGCCATCCCGTCCGCCCGCAAGCGGCTGGGGACAG
GTTCTCCGCCCATGCGCAGCCAGTGGCCTCATTCATGAGGGCGTGGCGGGCCGGGCCGCGTTGGGCCATCGCCGGTTGGGGGCCCCCACGCCGTCTACCT
GAGTGGACGCGCCGGGCGAGGCTCCCACCACCCCAGTCCTGGGGTCCCAGACGGCCTCCTGGGCGGAGTGTTCTACCCCGCGGCCACCCCCAGCCCGTC
TGCGGCCGAGGCCCCACTGGAGACTGCATTCCCCGCGGCCGGGGAACCGGCCACACCGGCAACGGCGCTCCTGACCCCTCCCGCCTGCCACACTCCCCG
CGGCGCGGAGCCCCACCCATACTAAGGCCCCTACGCGCGGCCGACGACATGTTGATGGCTTCGCTGGGGGGGCGGTGACACGTCCACGAGGCGCCTCGC
CGCCGTCGGCCCTGCCCCAGCACGTTCCCCCGTCCCCCGGGGGTGCGGCGTCCTGCTCGCCCCCCGTGTAGGAACGCCGACCGGGCTGCGTCGTCGATGA
GGCAGTGGCCCGCTACGCGGCTCCTCTTCGTACGTGCTTTTGGCTGAAGAACGGGCACCCCCACCATTACGCAGTTGGGACGGCGCGCGGCTGCGAGGG
CCCAAATCTCTCCCATGCGAGTCGTGGTCGCGCCCGTGGACCTCTGAGGGGCAAGGCCGCTCGTCTGCTGAGCGGGGATGTTGCGCCCAGCAAATCGAG

GAAGGTTTGCTCGTAACTTACGGGTACCGAGACTCCTCGCTTGGCTCACCCATTGCGGCAGCCGCGGCCGCCGCCGGGACGAGTAACGGCGTGTCGACC
GCCCCTCGACAACGCCTGGTAGGACGCTCCCCTCCCGGCCCGGACACTAACCCCCGCAACAGCGAATTCCAGGGGCGTCTTCCGCCCAACAAAGACTCA
TCGTGCACGAATTGGGCTGGCGGGCAAGCATGTGCATCACGGACTAGGGTACCGCCTGCCTCCCCGCTGCCTCCGGGGCCCTGGCGCTCCGTCCGATTTT
ATCCCGCTCTGCTGCGATACTCAACCAACCGTTTCGCTTTATTCAGGCCGCCTCTGGGGCCCGGCCGAACACTTTCTCCCGACAAGTGGCGTGACGGACCT
GCCTTCGCGCCGCCCCGAAACAGGCGGGCCGGCCTCGCACCCCTGGGGAGCGGTCCGCCGCAGGCGGGCCTCCTGAGCGCGCGGGTGGCCGGCCAGCG
CGCATACCCCCTCGTCGCTGCCGCCCCCCCCGCCCCGATCCCCGGCCCCGCTCGGCAAGACAGTGGAACCCGCCGCGCCCTTCACTCGCCCGGCTGGCCG
TATTTCGTGGGTCCTCACCTCAGTCGCGTCTGACAGATTAGACCTCGCCGCTGATGCCTAGGGTGGGACACCACCTTCCCGCCCGCCACCACCCTGTGGA
GCGTGAGCGCACTTAAGTCTCCCTACCTCCCCAACTCAGCAGCGGCCCCAGGGCATCCGCGAGGCTCCGTTGACGCACGTGCCGTCGTCCCGCGCCCACT
GTGGCACCTCGGCGGCGACCCTCCGCTGCGGGATGCCTGGAGGAGCGGTCCAGCCTGGGCGCTCTGCCGGTCACCAACGGCGGCCGGGCGGGGGCCCTC
GCGGTCGGACCGAACTTCGGAGGGGTCCCCGGCAGCTGAGTCGAGCGCTGCTAGTGACCTCCACCTCGGCCTAGTCACACTTGCCCGGCTCCCAACCGCT
AAGACATAGGGGGCGTCGCGACGAGGGCAATGCGGGGGCAACCTTGCCACCCCCAAGCGACGTCGCCTCATGTAGGTTGGAGGGGCTGTCTTGTACTCC
ACCGAGGGCGACACGTAGCGTGCGGCGGCTCGACTCGAATCTTGCGCCTCGGTACGCGCCACGCGACCGATGACCTTTGCAGGCGACTCTGCGAGAATG
GGGCCCGCGTCCTGGGGCGTGCTAAATCACGACCGCCGCCGCTGGCCTCCAGGAGGTCCCAAGGGGCAGCACACGCCATTCGCGTCGGTGGTCTAGCCT
CGCGGCACCTCGACGAGTCCCAGGACGCGTCGGGCCCGGTTCTTCCTCTTCCTGTGTGGCAGTCCGGCCGGGTGACGCTTCCGCCGCTGGCTAATTCAGA
CCGCCGTCAGTCGACTGGGCTGCCCTAACCAGCCAGGGCACTCGTGGACTTCCGGCGCCGTCCGACTCCACGCGGCGCGCACAGTCGCCTCCCTCCTTCG
TACCATTCGGCCCTCCTCACCCCAGGGGGGGCTTGCCGCCATTCCCTACGCTGCCATTCGACGTCCCGCGCTACTCACCCATAGTGCCAATTGCGGCGTCG
CGGACACCGGCCTGGCGCAGCGCCAGAGCGCCACCTGTGACGTCGCTCACCGCGGGGGGAGCCCAGCCTCGACGCCCCAGATGGCTCGCGGGGGGGGA
GGTCCCGAGGCCACCCTTCCAGGTGGCACAGATGCCCCTCCCTGGAGGGGGTGGAAAGCGCGCGGCGGGCCCAACACCGTGGGCGGCAGCTGGGCATG
CGCTCGGATAAGAGCGCCAGGATGGCAGGAGGGCCGATCTGGCGGGTGGTGGCCAGTGCGGGGCGGGGGGCGAAACTGTCTGGGGGTACCTGGAGCCC
AGGGTTCAGGGTCCGTGCCATGAGGCTGTCAGGCAAGCCAAGCCGTTAACGGCGGCCAGCGAGTGGGCAGGGAAGGTGACTAGCGCGAGCTACATATT
GCCGGCCGCCGCGCGGGGCGGCCAGCCGTGAGGAGCAATCTGAGGGACGGCCCACCGCCGGAGGGCCTGCACCATGAGGTGAGGCGGCGGGTCCCCG
CCACGCCGCGAGACGGTACGACCCGTTCCGGATGTGCACCACCGATGAAGAAGGCGCGCACATGCCGTTCGCTTTCGGCGTATGGTCTTCGCAGCCTCCG
GTGGGAGGCGCAGCCCGGAGGGTGCCCGCGCCACACGGATCAATGGGTTGGAGGGGCGGACTCGTGGTTTCGACCATTGGTGCAGCTAGAAACCGTCCC
AAGGCTCCACTCGATCGCACTGCCGTCGATCTCGGTTCTGACTCCCACCTATGCCAACACGGGAGTGCGCTATGAAACGCGGTTTTATAGTCGGTAATCG
CGACCGAACGCGGAGCGCCGTCTCGAGGCTTGTAACGCTCCGCGGTCGTTGTGGAGCGGACCGGGTAGTCAGCGTGACGTATTTCTAGCCCCCAGTGTGC
CGCCCGGCGAGAAAGTCCTCGCGGGAGCCCGTCGGCTGCCGCTGCGTACGCGGGGGAAAGCGGCCGAGGTACGTCTTCCTGGTGGCTTGCTGCCGCCAA

CCACCGATCGGCCCGGCTCGATGACAGCCGGCGAGAATGGGTTGGGGCCTACTTGGCCGGGTTGGCTGATCGTACGCAAGCAGCGTGCTGCGGGCCCAC
GCAAAGGGGAATGGCGGGGGAGCGCCGGCTGCCAATTCCCCCCGGCTGCACAGGCATGATCCGGTGTCAAGAACGGTCTCCGGCCCACGCGGCCGCGC
CTTGGCCCGAGTACGGCTGACTCAGAAGCCGTTGGGTGCGAGGGCGTGGGGTGGTGGCGCATAGGGAGCCAGACCCGACGGAGCCAGGATGGTTCGAG
CAGTGTCACGGCCCGGGGTCATGTGGGGCCCGCCCGCACGGATGACCCGCGTCTCGATAATCGGTTGCAGGCGTGCCGTTCCCACGGGGCGCGCCGGTT
ATCGAACGGGTGCGCGCGTAGACTCGCTTCCGGGCCCCGGACCTGGGCCAGGCAGAAGCGGGGATTACCCGTCTTGATCGCAGACAGCCCGGTGCCCCC
GGGGGCCGACGTACAGCTGGCAAGCACCGGTCCGGCGATTACCCGACCTGGGTTGGCAGGTACACGTTACAGGGGTGGCGTGCCCCCCGGCGCTTGGCG
CCGGCTTGGAGGGACGGGGGGGGATCTAGCTCAGATGGCCAGCGAGCGTAGCTTCGACGGTGGCAAGAGCGAGCGGCGGCGTGCTTGGGGAGGCCGCG
GGCGGGTGCGACGTCCAGTTCGTCACACGCATGAGGGGTTTCCGGGGAGGAATCACCGCCTCGCGCAGGCGTGTCCGACACGACCCGTGTAGCGGGTGT
GAGACATTTAGGCGGAGGCCAGAACCCCTTGCCGGAGGGCTTGTAGTATATTGTCAATGGGGCCGACACCACGTGCGGATCGGGACTGAGAGCGCGTAG
AGCCTTGGCCCATGCTATATTTGTGGAGCGGACACTGTGCTTTCCACACGTCACGCTGGCGTAAGCCGATGCCCGTCACCGCCCGCGCCCCAGGCCGAGT
GCGGGGGGCCGCAAGGTCCTGAAGCCGATCTACGATGGGCTGGGTGGGCGACGTCGTGGGCTAAGCTCGGCGGCCCCACCGTGAGGCGGACGTGGGGG
CCTGTACTAGAGATGGAGCGACGGTCGTCGATACAGTTCTTGCCAGGAGACCGAGCATTTTGCTAGGCACGGGCTGCATGGGCAATTCATTAACGGCGG
CGGGCCCACCGCGGCCAGCGGTGGCGGGGCGTAGTACCGGTCGGCAGGGATACGACCCCGGGCCGAAATTCGGGGCCGGCGGCCAGGGGGCGGCGCG
CCTGCGCGCCGTGACGCTGACGCCCTCCCGGGGTGCCGCGGGCTGGCCTGAGGCCCAAGGAGGCGCCTGGGGGTCGAGCGCGGGCCCGGGCGCACGCT
GATCCCGAGCCGACGAGCTTACGGATTGACCAAGAGCACCGCGAGCGTCCGAGCGCTGCGGGCGTGGTAACAACTACATCCCGGGTCGAGTTTGCGGCT
GGGACGTGGGATTGGCCGTGCAGACGCGCTGGGGGGGGAGCGGGGCTGGCCCCTCGCTCCGTATTCCCCACTCCACATGGTCCCCGGGCGTACGGGCGT
CCCCGACGGTTCGGGACGCGGGAATCGCGCAGGTGCCCCTCGGTGCGTGGCGGGCTGCGTGTAAGGCGCTTTCGCTGCATGCGGCCGTCTCGCGGACAT
GGCGTACGGGGGCGACCAGGGCGAATCCAAGGTGCGCCCAACGGGCCGAGGCGCTACAGATGGGGAGCCCCGGGCGGCCGTACGCAGGAGTCCAGTG
GCGTATGGAGGGAGGCCTGGGGCTCCGCAACGGCCGAGGCTATGCCCAGACCCCATGCCATTGTAGGCCGAACGCGATAGGTCACGCCGGTGATCCTGC
CGGGGTCGCGGGCCGGATCCACAAGGCCAGACCCATAGCGTAGTCTATAGGCGTCCGGGAAGGGTCGTGGGGGGGCTAA

>2017.TE.25008.1.10

CACGCCCCCCTCCCGGCCAGCCCGCCCCCCGCCCCAGCGTCGTACCCGGCGTCACGACTCCCCGCCGTCGAGAGGCCTTTGCCGGGGCCCCGGCTAGCC
CGGGGCGAGCGGACCGCTGCTCGCGGGGGGTGTCACCCGGGGCACACCGCGCCAGCCTCCCGGGCATGCCACGCCGGGTTCCGCCCCCGCTGCTCCAAT
TTGGGGGGCCCCTAAGATCCCGTTCCCCAGGCCAGACGCGCCCGCGGGCCAACATGGCCACACCGTGTCGCCCCCCCGCCTGCCGCACTGACCGTCTTGC

GGGCCCTGCTCCCTGGCCCGAGCACACCTCCGGGGACCCCCCTCGCTCGGCCGGGGGAGCCTTAAGCGCGGCACCGAGTAGGGGGTCGCGTGTTGCCGC
CGCGCTGACGCCCCGGCTGGGACCACTTTCAGGTCGAGACGCCGGGGTGGCCATGCGGGTCCAGCCGCACGATGGCGCGGCGATGTGGCGCCGACTGCC
CCGTCTGTCTTCTCCGTGCCAGCCCCTCGCCACCATGCCGGTCCGGCCCGCACGGGGGTAGGAGGTTCTGCCGCGGCGGGAGGCGTAGGACGCCATATCC
GAACCGGCGGGTCCCCCCGGCCACCGAGTCGACAGGACCATAGGCACCGCGTCCGGGCGGGCGCCCTTAACGCCGCCCCTTACTGGAGAAGCAGAGCT
CTGCCGAGGCACCCTGCCTAGCTGGCTGCGTCCCGCACCCCGCGATCCCCCTGGTCCACTTACCCAGCGGGATGCGGGGATGTTCGCAGCTATCACACGG
TCGGGGCCTGCCTCCCTGGCCGGCGAGAGCACCGCCACCTTCGTGTTCCTTGCCGCCGGGCGACGTGTCCGGGAAAGGCTTCGCCGCGCGCCCGCGCA
GAGCGCCGGGGAGTCCCGTGAGCGGCCGCAGGAACGTCACGGACCCCGGCTTGGTGCGGTCCCCCTCAGTGGTGCCCGACCAGAGACCGCGGAAAGAC
TCCAGGAAACGCCTCGGGGGGAAAGCAGGGCCGCGCACTTCCGCTCCACGCTAGCTGGGGGGACCACGACGGCAATACTGAACACCTGGCTCTTCGGC
AGCCCGGGCTATGAGGGGCCCTCTCGTCCCAGCCAAAGGCTGTTTGCCTTCCTGTCCGGGCACCCTCGACCGCACCCGGGCGGGCGCAGGCTTCATTGCG
GCGCGACTCGCCAAAAGTCCCCGCGATCTCAGCGAGTACCCTGAGTGCTGGCCCTAGTCCGCCTGCCCCCTTAATGTCGCTCAGGTCTGCAATCTCTTACG
ACCGGCTCGGGTAGTGGCCGCACGGTGCGTAGGTCCGATGCGGGCCGGGCCGCTCGCGCCGATCGTGCCGTGCGGCGGCCCTGACACCGTATGCGTGCG
CGCTCCGCAGGCTCGCGAAAATCCTTCGCAGGCACTTGGCCCCTCTGCTAGACGTGGTGTCTCCTAACTGTGGCTCACGCACGGCGGTTAGGCCACGGTA
GCCCGCTGTTCGGTCCTTACGACTCGGCACACGCCCCCGTGCACGCCCTCGGGTTCCGCAGGCCAACGCCTGGCCCTAGGGTTGCGTGCCGGCCTGCCCG
CGATGCCACTTGCCCCACGTCCCCCACCTGGCCGGCGCAGACGGGTTCAGTGGGTGTCGAACGCCATTCAGTCCCCTGGGACTCCCCCGACCCCTCCCAT
GGCACCCGCCGCCTGGCCTTGGAGCTCCTGCGCGTTGGACCTTACTGCGGTCGTCAGCTTCTCGGCGCGCCCCGGACTAGTCCTGGGCGGCCAGCTCGCG
GGACCCTAGCGCGCACGCGTGCCCGATTGCCGTATCCCCTCGACGGCGCCCGGGCATAGGGTTCGATCCCGCTGCTATTGCGGGTGCGTCCAGACCTGCC
GCCGTTTGGCGATCCGAGTGTGCGAAGGGAGCCAGCCAGCGGGCCGCCTGGCCGCACTCCAGGATCCTGGCGCCAAGGGAGAGCCCTGCCAGCGCTAC
GCGAATACTCGACCTGTCCGCGCCCCGCCTCGGGCTTGCGAGATGGCCACTAGACGCTACCGGGTCGTCACCCCGTCGGCCCCACAGGGGCCCGGCTTC
GCGTGGCTTTCTTGTCGAAGGCCCCGAAATCCGGCAGGCCCCGACACCTTCACCATGAGCGAGGACGCGCGGTGCGGGATGTTCAGTTCATGCACGCCC
CTCCACGGCCGCCTTCGCGCGGACAGCGTCGGGCGCGCCCAGGACGAGAACAGACCCTGGGCCAGCCTCAGGGCCCGGCCTAGGCGACCCTGGTGACC
AGTTTCCACTCGGCACCCTCGGGCTTCGTGGCAGTGCAGTCATGCCCCCGGGTGCTCCCGCGCGACCGCGCACGCCCCACCCAAGGTTCTTGGTCAACGC
GCCGGATCTGACGTCGCGGTCCGGCCCGCGCCGTAGGCAAAAATGGGTTCGGCTCCCGCCACTCCGCCAGTTCGAGGGCGGTAAAGATCTCAGCCGCCG
TGTTTCGGGCTATCTCCCCGCGATCCCGGGCCACAATCGCAGGTGGCTGGGGCGTCGGCCAGACCCGTCTCGCCGGCCTTTCTGCGCACATTACCTCCGA
GCAAGGCAACCGCTCCGCCCCATTACAGGGGGTTGTCTGGTACCTCCGTCACGTACCCCCGCATCGCTGGCGGGCTGACCCAAGCCCCCTCCCATCCCGA
CCCTCACGCGCTCACGCCCCTTGCCGGGCGGGGTCGTGGCTGCCCAGGGTCCAGGGCCCGCCCCGGGGGCCGGTTGGTTCCAGGAACTGAGGGAGGGTC
GCCCAGCTGACCGCTCCTCTCGCGGGGTCTATTCGGCGTGGCCATCCGCAGGTGCGACAATGCCGGCTCCCATTGCCGGGCCGACCTTGGTCCCTGGCGC

TTCGGACTCCCGGGGAGCGCCTCCCTTCATCGGCGGCCCCTGCCCCCGTCATGACCCCCCTTACGCGCCGGTGGTAGCCGCGCGTGGTGGCGTGCCCCTCT

CCATCATTACGCTCCACAGGAGTGGCCACCGGTATGGGCGCGGTGCAGTCGTACATGTGTGCGGCCCTGTTTTGCGAACCCTTCCCGCGGTGACCTCCGC

CCCAGCGGCCTTCTTCGACCGTTAACGCCGGCCAGCACCCGAAGGGGGTCACACAAAGCCCTGCAAGGATGCGACGCTGCGTAGCCGCAGGTTAGCCGC

TTCCTTCCCAAAACCGTGTCAGAGCTGTCCGGCGGAACGTTAACGCCGCCGGGAGGATCTGCGGCTACCCTCAGGCCCGTCCGGTGGGAGTGGCCGGCG

TCGATGGGGGTACGCACCCCACTAGACGCGGATTTTGGCCAACGGGCACGGCCGCTTCCGAGTTCCCCTGCGGTCTGAGTGTTGGTACGTTCAGCCGGCT

GTTCCGTGGGGCAGAGCGCCGCACGCATGCCGCAGGAGCGCGCCCTCCGCGTCTTGCGGGCGGGGTCCTAACGAAGCCCCCTGGGCTTGGAGGTAGGGG

GCGCGGGGGAGCGATGGGAGCGCAAGCGTGCCATCGGGTCCGGCCCATAAGTGTGTATGGCGCACCGCGCTGCCGCGATGTCCGGTGCGCGGGCGCCG

CTGGCTAGCTCACTCTCCCCCGACTCCGCCGCCGCGCCCCGAGACGTATGGAACAGCTGGCGCGCGTCCCCCGCCGCGGGCGGCAACGAACTAGATCCA

CCGCCCTCACGAGGAAGCGCGCCGCCACCACGTCTCCCCGGCCGACGCGGTCCCACCATCCGCTGTGCCGGCGCGCGAGCAGCCTGGCCCTCGGGGCGG

TCGCGCCAGCGCAGACACCCTGTCACGCGCACGGGAGCCCTGGCCACCGCGTCGACGCCCGGGCTAGCCCCGACTATCAGTACTGACCAGAGGGGCGC

CGGTCGTCCCGACTGGTTCCTCCCGAAACGCTGGCGGTGGGCCCGCACGATTGGTGCAGGCCCCCAAGAGGATACGGCTATTCGCGGCTGCCCGAGGCG

AGTCGGGCCAGACCGGTCGCGCGTGTGTGTGTTAGCCCTCCGTCGCGGCCAAGCCGGCGGCACCGGACTGGGCGACAGTAGCGTTCCTCTGTCACTAGTC

ACTCTAACGGACAAATGGAAAAATGGCACCTATCGCTACCGACGGGGTTTTCCTCCAGGATAAGCCTCCGCGCCCCCCACTGGACGCCAGCCATCCTTCC

CCGAACGGCTCGCCTGTGCCGGCTCCAACCCCGCGGGACCTCCCTTCGCGGCCCCGGCGGGGATCGGTGGACCGTCGCCGCCGGCGCCTACCGATCGCG

CCGCCACGGTCGTGCACCAACCAAGCACTGCTACACGCCTGTGCGGCCGAGGCCGCCCCATCCAGCAACGGGGCACCGTTGACCCGTCCCCCATGATGC

CGGACATGGTAGCCCCCACACATGCATCTTCGGGTAACCCGCGCCAGGTCCCCCCCCGCGCCGCCGCCGGCTTTCCCCCCGCTGGTGCTAAGCGGCAGAT

GCACCACTGACGGGACACACTCGCCTCCGATCCAGTGCGCTGCCGCCTGCGGCCCCCCACCGGACTGGTGCCTGGCGCTACACGGAGCCGGCCGCGCAC

TGTGACGTATGGATGCCCGAGGAGACATCCGCCCCGGACGCATGCGCAAAAGACCACTCGCTCGACGCCACGACGTGTGCGGCCGGGGTGATGAACGT

CGGCCGGTGTGCCAGTGAGGCTTGCACCTACACTCCCTGGTCCACCGGGCCGTTCGAGCGGCACCGGGCGAAGAACCCGCAGCCTCCCGGCTTACGCAC

CGGCCTCGCGTCAACAGGCAACCACGACCGCAAGGCCGGCAGTGTGTGTCGCTGTGGAAAGGTGCGCCGCAGGCGACCCCCACGTCAGCCCACTACAA

AGCGTGGCAGCATGCTGCGCAGCGACCGCACGCCCCGACACAGCCCAATGCACAGCATCGGGGGGGCGGAATCGCCCCACGCCCGTAACATCCCGCGG

GTCGACCGCCTTACACGTCGACTGCCACACCCTGGTGCCGCAGTGGTATCCCGAGACCGCATGACCCGCCGCGAGCCCGCTGACCACCTGTCCTTGATCG

CAGTCGCCACGGGGCCGGAAGGTGTACCGCATACCGCACGTGCTGCGCGTGTGCAGCACGGGGGCACCCTGCATGTATGGATCCCTCTAAGCCGACCTG

CCGCCCCAGGCCAGAGGCTAGAGTCTGCCGCCTAGGCCCCCCCCCGTGGTCGCCAGCCCCGGTGGCGGCCCACCCTCGGGGCGACTGACCGGCCACCAT

CCAGGGTAGAGAGGTTTGGCCCTAAATACGGGCGTGCCGAGGAGGTGTTTGGGGCGAGCCGCAGCAGGTTCGGCGGATCCTTGAGGGTGGGGCCTGGGG

TGGGACGGGGGCGGCCGAAACGCGAGCATAGGCCCCTGCCTGGCCCCTTTGCAGTCTGTACTTTTATTGACATGATACTAATACCGTGTTCCGCCCTGAG

ATCGCACGCGGACCTACCGTGCCTCTCTCTGCATAGTGCTTCAAGAGCCCAGCGCGACTGGGGACCGGCTCCCGCAGCCGGCTCGGGGCGTAACGCTCG
GTGGAAGGGTCACCCGGATAGCTCCCGGTTAGTTTCGTACAGGGTGTCGGGAGCGCCCCCAGCGAGTCTCCTGGAGGTGCAGGGACGGCGGTGGTCCTC
GAGGGGGCGGACGTTGGCCGCATAACGGGTAGTCTCCAGGGTCACCTATAGTCTGGCTCGCCGTCAGGGTCTGTGAGCGTCGTGCTCACGCCTCCGCAAC
AGCCGGCGACCCCGGCTCGCAACGGCCAGCCGTGGGCGGGGGGGCGACCGGACGCAGTTCCGAGTCTAGGCATGGTGCCACCGCCCTCTCGTTGGGTTA
GTCTCGAATGGCTGTTGCGGGGGTCAGCGCGGCGGGGCCGCTGCTGGGAGCCCGCATAACCGTGGATACACTTGCTGGGTGCGCGCTCGAGGGCGGCCC
CCGAGGGCTGGAAAACCATTTCGGGATCGCCCCCGCGCACCCGTCCGCACTGCGGGGTCCAGCCTGGCGGGGGCGGGTTTCGTTGGGGCAGTACTCACG
CCCACGGCGAGGCCCATCCAGAGAGTGAGGGTGTCGCTGGGGGGGCCTACCGGGCGGAACGCGCCCCGACTTAGGGCGGCGCAGTGCACGACCAGTCA
CCGGCTGGGCGTCGCCGCGCCGTTGAGAACCCCAACACAGAAAGAGCTTGCCGCCTCTGGGGGAGCCAGGCCGAGAAGCGTGGGCGAGCGGCCACGCT
GCGGGGCCTCCGCGGACCGTGCGGCGCTCTCGGGTGACAAACCTAGTCCCCGTCCCAGGCCATGGGCGAGCAGCTACGCCGGGACTGTGCGACGCCTAT
TCGGCACATTCGGTATCATTGGCCTGGGACGACAGCCGGCGACAGGGCTGACGAGGCGGCGTGGACTCGGGGTGCAGGGACGACGTCCACCCGTACTG
GTCAGTAGCCCAACGCGGAAGCCTGTCTGGGGCTTTGGCCCCTAGACATCGGTCCGGGGCGGGGCGCGTCATCGCTACGTTGGTGAGGGGGAGTCTGCA
CCGATAGGGTGTGGTGGTGTTGCTTAGATCGTTGACGGGCCGTGAGGTGGCGCTGCTGTTTTCCGGCTTACGGCTTACGACCTACCGCCCGCCGCGCCGGC
CCGAAGGCGGCGCCCCCGCACATCGCCCTTGGGTGGTAAAACCCGCTCTGCCGCCGCTACCCCCCCCCCAGGGGTGGCCGGAACGGCGAGGGCAGGAC
ACGGTAGACGCACGCAGCGCCGCGACGCCCGGCCCGCGCTAGGGTTGGCACCCGTGGGACCTCGGCAAACTTTCCGTACGATTGGGGGGCGCATACAG
CGGACGGGGAACCCGCCCGACGCACGCGCGGTCCCGAGGGGTGGGGGACCTGCAGCTGCGTCGCGCGGTCCGCGATGGCGTCTTGCTAGACCATGGGC
GTTGCAATAGTGGGAAGGTGCCTCCCAGGCTGCGGGGCGCCGGACGCCCGACACCAAAGCTGTGCGAGGACAGAGAGGCTAGGCACGGATGGGTCCAA
GCGAGCTGACAGCCTCCATCCTTGAGTGAGCACGCCGCGGCGAAATGCCGGCCGAAATGGGAGGGGCGCGACACCGGATCGTGATCGAGCGCGTGCGC
TTCGCCCACACGCCCTGACCCGCTGAGTGTCTCGGGTTTGAACCCCGTGCCCGGGGGCGCAGTGGGATAGGCACGACGGGCAGAGTTAGGGGCCTGGGC
ATAACGGCCCTGTCCGCGGCCGAGCGGGCCCGGGTCTTCTCGACCAGGGTGCCGGCCCCGGCGGGCCCCCCACCCACGGACGGGGACCTGGAGTTGAGC
CAGGCCACAGGCGTGACCGGAATTGGAGCGCCAGGGGACAGCGGCCGCCCGCGCCAGGCCACGCCGGTGGGGGCGTTCTGCCCAGGCGTCGCGCAATG
GGCGGACCGGTCTACGCGACACCGCCCGCCCGGCCTGGAACTCCCCCCGAGCACATTCGATATGGTCCGCATCAAGGCGGGAGGCATCGCCGGGGGCC
GCGCGGACCACGACCTTCGAGGGGGACGGTGCGGGTCGCGACGAGACGGCGGCATGTGCGCGGGTGCCGTGAGCCCTAGAGTCAGCGGTTGCTGGTAT
GGGCGGACGGGCGCCCCCGACCTGCCGTGGACGCCGGCTCGCGGACTAGTCGACCCGCTATGGGTGCTGTGCGTGACGTTGGCCTTGGTCCAGGGCGCG
GCGCGCAATACCGCGCGGGCTGAGTATCGCGTCCACGGACAGGCGGCTGAGCCGCCCGGCGTAGCTGGTACACGGTCGGGCCGAAGTGGCAAAGAGCC
GGCTTGCGCCCCCAGCGCCGTTGCAGCAGGTCTGGACAGGCAGTACGGAGCGAACGCGGCGAGACACCCGGCTCCGGGCGTAGTCGGATCCGCCCGCC
AGCGGGCGCGCAGCGGTGTCGCGGTCTTGATGCGCCGGTCAGGGAGGTCGTCGCGAATGAGCCGGGTGGAGGGTGCAAGAAGCCAGACGATGGGAGCA

CCGAGTTGTCCGCGTGCGATTGCCGTCCCGGCGAAAGCGCCAGGCCGCGGCGCGTGTGGCAACGGCAAGTACCCGGAAACACGGCACGGGTCTCGTGG

CGCCGCGAGGCGGCGGGTGGCGCGCTCCGGGGGGCTGCGCAGCCCCTCAGCGCCGCTGCTAGTGCCAGACGCTTGCCCTTCTTGGGCGCGGCGGCGGGA

ACTCGGTCGCGTGCGTTCAGGGCCCTCATCCGCAGACGTGAGGGGTGCGGGCCAGGGGGCGCAGAGGCGCCTTCCGAAACCCGTAAGGGATGTTCTGGC

GTTGGGGGGCCGCCGTCTTAGGGGGGTAGCGCCGCCTCAACTCCAGTATCGCCGCAAGGCCGGGGCCAAGTTCGATCCGGGGGCGGCGGCGTACCGCGT

CCACGCGTCTCCCAGAGGAGGGTCAAGGTGCATACGGGAGGGATCATACGGCAATCGGCGGCCGGGTTCCGCCATCTAACGTGGCGATCGCAGCGTACC

AGAGCGAGGCCTATGGGCCATATCCCTGGCGGGAACGGGCTGCTTGCGCCCCGTCCTCGCCGACCCTCCGTACCGGTACGGGGCGAGGATCCCGACACA

CAGCACGGGGGCGGCGCGCAGCACCCACCCCGTCCTCGGCCCAACAGACAAAGACCCCGCGGCGGGCTTGGGCACCCCCTTTCCGGCTACGTCCACCG

ATTCCGTACAGGACAGCCGTTGCGATGGGCACCGGCGATCGGAGAGAAGGACGCCACAGATCAGCAGACGACTCGGGGAAACCACGGGACCGCGCGT

CGCGTCAACTGCCGCGGGAGTGAGAAGGGAGCGACAGGAGCGCGGAGGTAAGGCCAATGGGGCCAGTTGAAGCCGCCCACAGAGGCTGTGAGGGGCC

CCGCCAGGTTTAGGAGCTGTTCTTGGAGCCTGTCGTTAGGGGCGGGGGGGGGCTTACACGGCGCTGGGGGACCCACGGGGCGCAATCCGCCGCGGACTG

CTGGAGGGCTGCCTGGGGCAGCACCCCAGGGGCGGCCATGGCGCTTGTTGACGGGGGCGCTAGGACCTTGTCCTAGGGCGCGCCGCTTTCGCCCACAGG

GCTTCGGTGGGCAGTCTATCTGTGCGGTTTCAGTCTCGTACGCACGTGATGCGGTGTAACCTGAGCCCGGCAGTTGTCTAGAGTAACTAGCGCGCGGTTAT

GGCTTCGTCTAGAAACGGGCTGCAATGTTCCGGGTGTTCATCACACCACGTTCCGCTTTGCAGCTCACCCCCCGACCACACCCGGATTCGGACCGGTGGT

CCTTCCTCTGAGGTACTCGCCGCTCATACAACCGCGGTTCCCGCTGTGGTCGGCACGGCCCGGGCTGCAGGGGCGTGCCTTCACGGGTAGTGTGCCTGCG

CGGCGTGCGGCTTCCGCAGTCCACGGACCGTTCCGGGGGTGAACGATGGAGGTGAGGGGACGAGTCCCTCCGAGATTAATGCCCCGCGGCAAGCCCGAT

CAGCGCTGCGGTCCTGGGCTGTCAGCGCGCGCGGAGGAGCGCCCGATCGGGCGCGGGGGAATGAGAGTGTGGAGGATCATAATAGAGAGATCGCCCCG

GAGACTGCGAGGGGCATGGACGCGGAGGGACGGGGCGGGGGGTCGGCACGCCCGTGCGGCCTGTCGTCATATCCCACGCTGTCACTCGGCTGCGTCCGG

CACTCCGGGCGGCCGGCGAGCAAAGCCGCCCTGCGCGGCCCGATCGAGGGTGCGGCGGCGCGACGTCGTGGCGTGGGAAATGCGGCGAGGGGCGGGGT

GGGGGACGTCATTGAACCTGCAGCGCGCACGGTGAGTACCGCGGCCCGTGGCGGGGCTGTACTGCGGGACCCCGGAGCGGTAGCGGGGGTTAGACTGG

ATGAGGGTAACCTGCTGCCCCGCGCACCACGTTCTTGGCCAGAGGCCCTGTGGGTGTTGGAGCAGGGTCGAGTATCCCTCCGTGCTTGCCTGCGTGGAGT

GGTTCGCCATGCAGTTGGACTCTTGGCGTCAGCTGGGGCCAGGCGGCGCCTCCGAGGTCGTAAAGGCCAACAGCGCTGTGAGATGGGCGCAGGAAGAG

CGCCAAAGACCTCCTGCTCCCCGATGTCCGCCTCGGGCGGACGTTCGGCCGCCTCGGGACTAGTTGAAGTGCGGCTTGGCTGGGGGCGCGGGACTCGGA

AGTCCGGGTGGAGGATGCGGGGAAGCTGCCCGTCGCCGGTACTGAGGGACTGTAGCTGTGATCCGGGACCACGAGGCATGCACCCGTCGTGGTCCAGCC

GGCCTGGCGCGGGGTCACAGAGCGAAACGGCGGACGCTAGGAACATTCGCTACCACCCCCCGGCGGCACCTTGCAGGGTCTACCCCAGTAAGGACCGG

CCCCAGCAGTGAGTGCGTCCGGGGGCTGGCAGCGGTAGATTGGGTCGCACGAGCTCACGTGGGCCGTTGACCGGAACCGGCAACGCCTGGGACTGGAG

CGCAAACTGGGGTACACTCTTCACGCCCGCTGCTCGTCATCGACCCCGGCGGGCGCCCGGGACCGGACCGGGGGCGACCCACCGGGGGACGGCGCGGC

CCCTGGATGGCCCTGCCCTCGGTCACGAAGCGGCCGAATGGTTTAGGGGGCCTCGGGACGCGGCCGTGGAGGTGCGGGGGGCCTGAAGTCCCTGCCTGG
CCCTCCTCCTAACGGCTTCACGCCCCTCAGGGCAGCTAGGGATTAACGTGGCCGGGTCACACGACCCGCCCAGCGCGGTACGCTGGGCCGAAGTGATTG
CGCGCGCCTGGCGGGTCCTCACCCCCCCACACACATCCGGGTCGGGGCGGAACTGGGTCCCGCCGTTCCCTCAGATCTGCCCCCCGACTTGAACATGCCC
AAAAACCGAAACCGGGCCGGCGCCCAGACCCCCGTGCTAAGGTTCCCACCGCCGACCCTAGCCCCCCCGGAGGTCCCCTGTACTCCGCACGCGACGTCG
TATCAGGTCCCAGAGCCCGACAATGCATGCGCGTCCCGCTCTGCGCCATGCCCATTGCCCGACCAGCCCCGCCCAGCGCAGAGGCCCCGACTGCTGGCG
CCCTCTGTGGCGCTCGGCCGCTCGAGAGCACCTGCCGCCCGACCGCAGTCTGGCTACCTGCTCCACGGGGCGGCGCTCGTGGTCTCCCGTACTCGGAACG
CCACGCCCCGGGCTCGCGCGTCTCGCGGCCCGATGCATCGTGGTCTTACCTCCGACCCTTTCCTGGGGTCGAGCGCGGGGGGGCAGAGCGCGCTCATCCA
GCCCCGCGCGGCAGCACCCGCCCGCCCAGGGTGGTACCGCTCTCGAGAGTCCGGACGGCCGAACGCCAGGGCAACGGGCCGTGTGAAGACTCACCGTT
TCGGCCGACCGCCATCCTAGCTGGGGCGAGCCCCCGCGCGCGGCCGGATGCGTATGACGTGCGCGCGCTCGGTGCGATGGCGCACGTCGACTGGAATAA
CCCCTTGCCAACCCCAGCGCCGAGAGTCCCTCTTCGGGTCTCCCGTCTGCCTCCGATGGGTGGCCCCTGCCGGTACTTGTTACGTCCTGGCTCGCCGGGCG
CCTCGTCACCTCGGCAGCCTGGATCGCTTGTCGCCGCCGCAAACGTGCGCACGTTTCGCCACGGGCCGCCCCGCGAAAAGCGGCGGGAACGTGGACCAC
GCGCGCGCCCGCCATCGGCTCGCCGGGATCCCCACCGCGGCAGCGCCCCGCCACAGCTCAGGACGCGGCCGATCGACAAGCACATCTCCCGACTACCG
GGCCCCGCGGCGTGAGCGTGGTCATGGAAAACCCGAGCGTGTGCGTTCCTGTCACGGGATCCCGCCGTGGGCAGTTCGCGGCAGGGGCCTACCCGTCGA
GGGGCTACACCGGACCGCCCCTCCAGCTGCATCGTGCAGCCCTAGTGCTGCAGCGAACTTGCCCCGGCCATCCCGTCCGCCCGCAAGCGGCTGGGGACA
GGTTCTCCGCCCATGCGCAGCCAGTGGCCTCATTCATGAGGGCGTGGCGGGCCGGGCCGCGTTGGGCCATCGCCGGTTGGGGGCCCCCACGCCGTCTACC
TGAGTGGACGCGCCGGGCGAGGCTCCCACCACCCCAGTCCTGGGGTCCCAGACGGCCTCCTGGGCGGAGTGTTCTACCCCGCGGCCACCCCCAGCCCGT
CTGCGGCCGAGGCCCCGCCGGAGACTGCATTCCCCGCGGCCGGGGAACCGGCCACACCGGCAACGGCGCTCCTGACCCCACCCGCCTGCCACACTCCCC
GCGGCGCGGAGCCCCACCCATACTAAGGCCCCTACGCGCGGCCGACGACATGGTGATGGCTTCGCTGGGGGGGCGGTGACACGTCCACGAGGCGCCTC
GCCGCCGTAGGCCCTGCCCCAGCACGTTCCCCCGTCCCCCGGGGGTGCGGCGTCCTGCTCGCCCCCCGTGTAGGAACGCCGACCGGGCTGCGTCGTCGGT
GAGGCAGTGGCCCGCTACGCGGCTCCTCTTCGCACGTGCTTTTGGCTGAAGAACGGGCACCCCCACCATTGCGCGGTTGGGACGGCGCGCGGCTGCGAG
GGCCCAAATCTCTCCCATGCGAGTCGTGGTCGCGCCCGTGGACCTCTGATGGGCAAGGCCGCTCGTCTGCTGAGCGGGGATGTTGCGCCCAGCAAATCGA
GGAAGGTTTGCTCGTAATTTACGGGTACCGAGACTCCTCGCTTGGCTCACCCATTGCGGCAGCCGAGGCCGCCGCCGGGACCAGTAACGGCGTGTCGACC
GCCCCTCGACAACGCCTGGTAGGACGCTCCCCTCCCGGCCCGGACACTAACCCCCGCAACAGCGAATTCCAGGGGCGTCTTCCGCCCAACAAAGACCCA
TCGTGCACGAATTGGGCTGGCGGGCAAGCATGTGCATCACGGACTAGGGTACCGCCTGCCTCCCCGCTGCCTCCGGGGCCCTGGCGCTCCGTCCGATTTT
ATCCCGCTCTGCTGCGATACTCAACCAACCGTTTCGCTTTATTCAGGCCGCCTCTGGGGCACGGCCGAACACTTTCTCCCGACAAGTGGCGTGACGGACCT
GCCTTCGCGCCGCCCCGAAACAGGCGGGCCGGCCTCGCACCCCTGGGGAGCGGTCCGCCGCAGGCGGGCCTCCTGAGCGCGCGGGTGGCCGGCCAGCG

CGCATACCCCCTCGTCGCTGCCGCCCCCCCCGCCCCGATCCCCGGCCCCGCTCGGCAAGACAGTGGAACCCGCCGCGCCCTTCACTCGCCCGGCTGGCCG
TATTTCGTGGGTCCTCACCTCAGTCGCGTCTGACAGATTAGACCTCGCCGCTGATGCCTAGGGTGGGACACCACCTTCCCGCCCGCCACCACCCTGTGGA
GCGTGAGCGCACTTAAGTCTCCCTACCCCCCCAACTCAGCAGCGGCCCCAGGGCATCCGCGAGGCTCCGTTGACGCACGTGCCGTCGTCCCGCGCCCACT
GTGGCATCTCGGCGGCGACCCTCCGCTGCGGGATGCCTGGAGGAGCGGTCCAGCCTGGGCGCTCTGCCGGTCACCAACGGCGGCCGGGCGGGGGCCCTC
GCGGTCGGACCGAACTTCGGAGGGGTCCCCGGCAGCAGAGTCGAGCGCTGCTAGTGACCTCCACCTCGGCCTAGTCACACTTGCCCGGCTCCCAACCGC
TAAGACATAGGGGGCGTCGCGACGAGGGCAATGCGGGGGCAACCTTGCCACCCCCAAGCGACGTCGCCTCATATAGGTTGGAGGGGCTGTCTTGTACTC
CACCGAGGGCGACACGTAGCGTGCGGCGGCTCGACTCGAATCTTGCGCCTTGGTACGCGCCACGCGACCGATGACCTTTGCAGGCGACTCTGCGAGAAT
GGGGCCCGCGTCCTGGGGCGTGCTAAATCACGACCGCCGCCGCTGGCCTCCAGGAGGTCCCAGGGGGCAGCACACGCCATTCGCGTCGGTGGTCTAGCC
TCGCGGCACTTCGACGAGTCCCAGGACGCGTCGGGCCCGGTTCTTCCTCTTCCTGTGTAGCAGTCCGGCCGGGTGACGCTTCCGCCGCTGGCTAATTCAGA
CCGCCGTCAGTCGACTGGGCTGCCCTAACCAGCCAGGGCACTCGTGGACTTCCGGCGCCGTCCGACTCCACGCGGCGCGCACAGTCGCCTCCCTCCTTCG
TACCATTCGGCCCTGCTCACCCCAGGGGGGGCTTGCCGCCATTCCCTACGCTGCCATTCGACGTCCCGCGCTACTCACCCATAGTGCCAATTGCGGCGTCG
CGGACACCGGCCTGGCGCAGCGCCAGAGCGCCACCTGTGACGTCGCTCACCGCGGGGGGAGCCCAGCCTCGACGCCCCAGATGGCTCGCGGGGGGGGA
GGTCCCGAGGCCACCCTTCCAGGTGGCACAGATGCCCCTCCCTGGAGGGGGTGGAAAGCGCGCGGCGGGCCCAACACCGTGGGCGGCAGCTGGGCATG
CGCTCGGATAAGAGCGCCAGGATGGCAGGAGGGCCGATCTGGCGGGTGGTGGCCAGTGCGGGGCGGAGGGCGAAACTGTCTGGGGGTACCTGGAGCCC
AGGATTCAGGGTCCGTGCCATGAGGCTGTCAGGCAAGCCAAGCCGTTAACAGCGGCCAGCGAGTGGGCAGGGAAGGTGACTGGCGCGAACTACATATT
GCCGGCCGCCGCGCGGGGCGGCCAGCCGTGAGGAGCAATCTGAGGGACGGCCCACCGCCGGATGGCCTGCACCATGAGGTGAGGCGGCGGGTCCCCGC
CACGCCGCGAGACGGTACGACCCGTTCCGGATGTGCACCACCGATGAAGAAGGCGCGCACATGCCGTTCGCTTTCGGCGTATGGTCTTCGCAGTCTCCGG
TGGGAGGCGCAGCCCGGAGGGTGCCCGCGCCACACGGATCAATGGGTTGGAGGGGCGGACCCGTGGTTTCGACCATTGGTGCAGCTAGAAACCGTCCC
AAGGCTCCACTCGATCGCACTGCCGTCGATCTCGGTTCTGACTCCCACCTATGCCAACACGGGAGTGCGCTATGAAACGCGGTTTTATAGTCGGTAATCG
CGACCGAACGCGGAGCGCCGTCTCGAGGCTTGTAACGCTCCGCGGTCGTTGTGGAGCGGACCGGGTAGTCAGCGTGACGTATTTCTAGCCCCCAGTGTGC
CGCCCGGCGAGAAAGTCCTCGCGGGAGCCCGTCGGCTGCCGCTGCGTACGCGGGGGAAAGCGGCCGAGGTACGTCTTCCTGGTGGCTTGCTGCCGCCAA
CCACCGATCGGCCCGGCTCGATGACAGCCGGCGAGAATGGGTTGGGGCCTACTTGGCCGGGTTGGCTGATCGTACGCAAGCAGCGTGCTGCGGGCCCAC
GCAAAGGGGAATGGCGGGGGAGCGCCGGCTGCCAATTCCCCCCGGCTGCACAGGCATGATCCGGTGTCAAGAACGGTCTCCGGCCCACGCGGCCGCGC
CTTGGCCCGAGTACGGCTGACTCAGAAGCCGTTGGGTGCGAGGGCGTGGAGTAGTGGCGCATAGGGAGCCAGACCCGACGGAGCCGGGATGGTTCGAT
CAGTGTCACGGCCCGGGGTCATGTGGGGCCCGCCCGCACGGATGACCCGCGTCTCGATAATCGGTTGCAGGCGTGCCGTTCCCACGGGGCGCGCCGGTT
ATCGAACGGGTGCGCGCGTAGACTCGCTTCCGGGCCCCGGACCTGGGCCAGGCAGAAGCGGGGAATACCCGTCTTGATCGCAGACAGCCCGGTGCCCCC

GGGGGCCGACGTACAGCTGGCAAGCACCGGTCCGGCGATTACCCGACCTGGGTTGGCAGGTACACGTTACAGGGGTGGCGTGCCCCCCGGCGCTTGGCG
CCGGCTTGGAGGGACGGGGGGGGATCTAGCTCAGATGGCCAGCGAGCGTAGCTTCGACGGTGGCAAGAGTGAGCGGCGGCGTGCTTGGGGAGGCCGCG
GGCGGGTGCGACGTCCAGTTCGTCACACGCATGAGGGGTTTCCGGGGAGGAATCACCGCCTCGCGCAGGCGTGTCCGACACGACCCGTGTAGCGGGTGT
GAGACATTTAGGCGGAGGCCAGAACGCCTTGCCGGAGGGCTTGTAGTATATTGTCAATGGGGCCGACACCACGTGCGGATCGGGACTGAGAGCGCGTAG
AGCCTTGGCCCTTGCTATATTTGTGGAGCGGACACTGTGCTTCCCACACGTCACGCTGGCGTAAGCCGATGCCCGTCACCGCCCGCGCCCCAGGCCGAGT
GCGGGGGGCCGCAAGGTCCTGAAGCCGATCTACGATGGGCTGGGTGGGCGACGTCGTGGGCTAAGCTCGGCGGCCCCACCGTGAGGCGGACGTGGGGG
CCTGTATTAGAGATGGAGCGACGGTCGTCGATACAGTTCTTGCCAGGAGACCGAGCATTTTGCTAGGCACGGGCTGCATGGGCAATTCATTAACGGCGGC
GGGCCCACCGCGGCCAGCGGTGGCGGGGCGTAGTACCGGTCGGCAGGGATACGACCCCGGACCGAAATTCGGGGCCGGCGGCCAGGGGGCGGCGCGC
CTGCACGCCGTGACGCTGACGCCCTCCCGGGGTGCCGCGGGCTGGCCTGAGGCCCAAGGAGGCGCCTGGGGGTCGAGCGCGGGCCCGGGCGCACGCTG
ATCCCGAGCCGACGAGCTTACGGATTGACCAAGAGCACCACGAGCGTCCGAGCGCTGCGGGCGTGGTAACAACTACATCCCGGGTCGAGTTTGCGGCTG
GGACGTGGGCTTGGCCGTGCAGACGCGCTGGGGGGGGAGCGGGGCTGGCCCCTCGCTCCGTATTCCCCACTCCACATGGTCCCCGGGCGTACGGGCGTC
CCCGACGGTTCGGGACGCGGGAATCGCGCAGGTGCCCCTCGGTGCGTGGCGGGCTGCGTGTAAGGCGCTTTCGCTGCATGCGGCCGTCTCGCGGACATG
GCGTACGGGGGCGACCGGGGCGGATCCAAGGTGCGCCCAACGGGCCGAGGCGCTACAGATGGGGAGCCCCGGGCGGCCGCACGCAGGAGTCCAGTGG
CGTATGGAGGAAGGCCTGGGGCTCCGCAACGGCCGAGGCTATGCCCAGACCCCATGCCATTGTAGGCCGAACGCGATAGGTCACGCCGGTGATCCTGCC
GGGGTCGCGGGCCGGATCCACAAGGCCAGACCCATAGCGCAGTCTATAGGCGTCCGGGAAGGGTCGTGGGGGGGCTAA
